# Posters

**DOI:** 10.1155/1995/73954

**Published:** 1995


### P001

EVALUATION OF THE METAL STENTS IN THE PALLIATION OF OBSTRUCTIVE JAUNDICE IN A HOSPITAL OF 1800 BEDS.

T.Butrón,M.Diaz Tie, M.J.Castillo, A.G.Carranza,O.G.Villar J.G.Borda,G.J. Parga*,E.G.Hidalgo*,M.Lomas. I Department of General and Digestive Surgery. *Department of Radiology. Doce de Octubre Hospital. Madrid. Spain.

Palliation of obstructive jaundice can be done with surgical bypass, endoscopic stent insertion or percutaneous transhepatic stent insertion. Self expanding metal stent have recently been proposed as better alternative for treatment of bile duct obstruction. This study was set up to evaluate follow-up results of the metal stents in our hospital. MATERIAL AND METHODS.33 Patients with obstructive jaundice treated with percutaneous transhepatic metal stent insertion at the Doce de Octubre Hospital, Madrid, Spain were reviewed. There were 12 women and 21 men, aged 37-87 years(mean age 64.4 years). Biliary obstruction was caused by pancreatic carcinoma (n= 7), cholangiocarcinoma (n= 9), gallbladder carcinoma(n=4), metastatic lymphadenectomy (n=5), hepaticojejunostomie strictures(n=4) and others (n=4). The histopathological diagnosis was proven in 21 patients. The indications for stent insertion included 4 hepaticojejunostomie strictures, 16 advanced diseases, 8 unresecable tumors, 3 medical opinion, 2 high ASA. External or internal biliary drainage was established during first session in all patients. Stent insertion was successfull in 32 patients.
RESULTS. Effective biliary decompression was accomplished in 23 patients, but only 6 of them had complete relief of jaundice. Early morbidity was 17.1% (bacteriemia 3, wound infection 1, hemobilia 1) and late complications were 12.1% ( Cholangitis 2, stent oclusion 2). 30-day mortality rate was of 12.1%. The overall mean survival was 58 weeks _ 19.5 standard desviation. The median post stent hospital stay was 9.1 (range 1-33 days).
CONCLUSIONS. Metal stent in biliary tree is useful palliative treatment for those patients with malignant obstructive jaundice when estimated operative risk is high or there is advanced disease. Hospital stay is low and quality of survival is better with relief of jaundice and pruritus.

### P002

**OBSTRUCTIVE JAUNDICE DUE TO NODAL METASTASES ; SHOULD WE PALLIATE BY PERCUTANEOUS STENTING?**

N Doctor, Helen Whiteway, A Salamat, J Dooley, R Dick, B Davidson.
Hepatobiliary Unit, Royal Free Hospital, London

Patients with disseminated malignant disease may ocassionaly present with obstructive jaundice due to extra-hepatic ductal obstruction. Whether useful palliation is obtained by stenting is controversial. We have analysed the outcome of percutaneous stenting in this patient group.
Over a 3 year period 8 patients (5 Male) mean age 56.7 years(range 39-77) with symptomatic obstructive jaundice due to nodal metastatic adenocarcinoma (stomach(4), ovary(1), breast(2), salivary(1) were referred to the Hepatobiliary Unit for percutaneous endoprosthesis insertion. All patients had undergone surgical resection, 1 month to 10 years previously. Three had previously failed an endoscopic stent insertion. In 4 of the 8 patients nodal metastases were the only known site of disease recurrence, the others having liver (2), lung(1) and peritoneal(1) disease. Percutaneous stenting was successful in 7 patients(87%), one being referred for surgical drainage. Acute cholangitis occurred in 4 patients following stent insertion (57%) but responded to antibiotics in all cases. All patients were discharged from hospital. Four patients were re-admitted with stent related complications (blockage+/-cholangitis). One responded to antibiotics alone whilst the other three required stent change with success in one. Follow up (mean 7 months) was available in 5 of the 8 patients, 4 of whom had symptomatic relief of biliary obstruction (80%). We would conclude that useful palliation may be obtained by stenting selected patients with jaundice due to metastatic malignancy.

### P003

**ERCP IN THE DIAGNOSIS AND MANAGEMENT OF BILIARY COMPLICATIONS POST LIVER TRANSPLANTATION**

B.Macfarlane, B.Davidson, J.Dooley, K.Dawson, M.Osborne, K.Rolles, A.Burroughs.
Department of Medicine, Royal Free Hospital, London

Aim: To evaluate the usefulness of ERCP in the diagnosis and management of biliary complications post orthotopic liver transplant.

Methods: Retrospective casenote analysis of 90 patients in whom the biliary anastomosis was end to end choledochocholedochostomy without T tube splintage.

Results: ERCP was performed in 26 patients with suspected biliary complications (29%). The procedure was successful in 22 (85%). ERCP was normal in 7 patients, a bile leak found in 4, a biliary stricture in 10, and in 1 patient a dilated biliary tree with no stricture. PTC showed a biliary stricture in the 4 cases where ERCP failed. In total, 20% of patients had a biliary complication, 4.4% having a bile leak and 15.6% a biliary stricture.The ERCP defined biliary complication was managed endoscopically in 6/14 patients (42%).One patient with a bile leak could be treated with nasobiliary drainage, and remains well. The strictures in 5 patients were balloon dilated, and 3 of these patients remain well. The fourth patient was retransplanted. The final patient developed a biliary stricture 18 months later.

Conclusion: The role of ERCP in the management of bile leaks is well documented. In this report half the biliary strictures defined by ERCP could be managed endoscopically. A prospective controlled trial is needed to clarify which biliary strictures are best suited for endoscopic management alone.

### P004

THE ROLE OF ENDOSCOPIC ULTRASONOGRAPHY IN CHOLEDOCHOLITHIASIS

Ü. Santaş, U. Yılmaz, B. Yıldırım, G. Temuçin, B, Şahin, G. Gürkaynak

Department of Gastroenterology, Yüksek İhtisas Hospital, TURKEY

Forty-nine patients with suspected choledocholithiasis were evaluated the efficiency of endoscopic ultrasonography (EUS) in the diagnosis of choledocholithiasis. The definitive diagnosis was established by ERCP, intraoperative cholangioscopy or cholangiography (IOC) in 38 patients.
ERCP, IOC or intraoperative cholangioscopy demonstrated choledocholithiasis in 24 of 38 patients. Eleven patients were thought to have choledocholithiasis on conventional ultrasonography (45.8%) and 23 patients on EUS (95.83%). The diagnostic accuracy of EUS was found more valuable than conventional ultrasonography and equal to ERCP. These findings suggest that EUS may be as sensitive as ERCP in the detection of choledocholithiasis.

### P005

LONG-TERM OUTCOME OF SPHINCTER OF ODDI DYSFUNCTION - RESULTS OF ENDOSCOPIC SPHINCTEROTOMY.
T. Bozkurt, U. Braun, K.-H. Orth, B. Butsch, G. Lux. Department of Internal Medicine and Gastroenterology, Community Hospital of Solingen, Germany.

In pts with sphincter of Oddi (SO) dysfunction, the benefitial role of endoscopic sphincterotomy (ES) is still a matter of controversial discussion, especially in biliary type II and III groups. The aim of the prospective study was to investigate the value of ES in pts with abnormal resting pressure of SO [ > 40 mm Hg]. The patient material comprised 31 [m:f=3:28, age range:30-72 y.] subjects. All pts who had underwent a cholecystectomy 1 to 45 years before entry to the study, suffered from biliary type of pain. Ultrasound, EGD, colonoscopy and ERCP was performed as a primary diagnostic work-up to exclude other causes of gastrointestinal disease. Sonographic and radiologic measurements of the common bile duct, pain induced by injection of contrast into the common bile duct, delayed drainage of contrast during ERCP [> 45 min] and results of a morphine-neostigmine test were documented. Manometric measurements of ductal pressure, basal pressure of SO and recordings of SO contraction frequencies were performed. All pts with elevated basal SO pressure underwent ES. In seven pts, complications occured after manometry or ES. During the follow-up [4-58 mo.], 83% of pts were improved. The results of our analysis assume that ES is an effective therapeutic modality also in biliary type II and III pts diagnosed by manometry. Morphine-neostigmine test was the most sensitive non-invasive technique in pts with SO dysfunction.

### P006

THE ENDOSCOPIC TREATMENT OF POSTOPERATIVE BILIARY STRICTURES
Z.Zádorová, M.Zavoral, P.Frič
Department of Internal Medicine, Policlinic of Charles University, Czech Republic

Between 1990 - 93 we performed 5 289 ERCP. We found 49 /0,9%/ patients with benign postoperative biliary strictures. They comprised 17 men and 32 women with a mean age of 60,8 years. In all cases were performed ERCP for diagnosis and site of stricture and the presence proximal biliary calculi. The most frequent strictu-re locations were common duct- site of cystic duct stump and distal common duct. 12 patients had calculi proximal to the stricture.
We attempted endoscopic therapy in 28 patients by the placement of one or multiple endoprostheses. The rest patients was reccomended for surgical repair. The endoscopic procedure was succesfull in 20 out of 28 patients. The stents were exchanged usually at 3-monthly intervals to avoid clogging of the stent. Follow up study during a period of 3-36 month's after stent removal of 9 patients showed 3 reccurent stones and 2 restrictures. We prefer multiple /2-3/ stents to avoid restenosis.
Endoscopic treatment of postoperative biliary strictures it should be the initial therapeutic modality owing to the difficulty of reconstructive biliary surgery and its associated morbidity and mortality.

### P007

**FAILED ENDOSCOPIC STENTING FOR DISTAL BILIARY TRACT OBSTRUCTION : IS THE PERCUTANEOUS APPROACH WORTHWHILE ?**
N Doctor, Helen Whiteway, A Salamat, J Dooley, R Dick, B Davidson.
Hepatobiliary Unit, Royal Free Hospital, London

Endoscopic stenting is a satisfactory treatment for patients with distal biliary tract obstruction who are unsuitable for surgery. However, for those in whom an endoscopic approach has failed the optimum management has not been established.
Over a 3 year period 133 patients were referred to the Hepatobiliary Unit for percutaneous endoprosthesis insertion. Of this group 29 patients (16M, 13F, median age 72 years, range 38-87) with distal biliary tract obstruction had previously failed an endoscopic attempt at stent insertion (failed cannulation (n=24), duodenal stenosis (n=2), duodenal diverticulum(n=2) and previous gastric surgery(n=1)). All patients were jaundiced whilst 20(69%) had pruritus and 9(31%) abdominal pain. Two patients (6.8%) had acute cholangitis at time of referral. The aetiology was carcinoma pancreas (n=15), bile duct (n=8) or ampulla (n=3), nodal metastases (n=1) whilst two patients had benign strictures. Percutaneous stent placement succeeded in 25 patients (86%). Of the 4 failures 2 patients with advanced cancer died following the procedure (acute cholangitis(1), bleed from inoperable rectal cancer(1)). In the other two patients (with liver metastases) the guidewire failed to traverse the stricture. There was one in hospital mortality in the group successfully stented (4%) due to pseudomembranous colitis. Five of the 25 patients with successful stent insertion developed acute cholangitis following the procedure (20%). All responded to antibiotics. Of the 26 patients discharged from hospital 8 (31%) were readmitted with stent blockage, 6 with acute cholangitis. All were successfully managed by stent change (endoscopic(6) , percutaneous(2)) without mortality.
Percutaneous stent placement for distal biliary obstruction is successful and effective in patients failing an endoscopic approach.

### P008

ENDOSCOPIC MANAGEMENT OF EXTERNAL HIGH OUTPUT BILIARY FISTULAE AFTER OPERATIONS FOR HYDATID DISEASE OF THE LIVER
A. Romanos, A. Manouras, B. Katergiannakis, B. Paizis, I. Linardakis, N. Apostolidis
A' Propaedeutic Surgical Clinic of Athens University.

The therapeutic ERCP, is an alternative method for the management of the post-operative biliary fistulae, in some patients, apart from the classic surgical procedures involved.
This study includes 13 patients (7M, 6F). All patients were operated during the last 4 years for hydatid disease of the liver, and all suffered post-op chronic cholorrhea due to communication of the residual cavity and a large intrahepatic bile duct. All fistulae, were of high output (>400 cc/24h). Common characteristic of these patients was: 1) type of operation, 2) moderate calcification of the cystic wall, (≥ 1cm), 3) long existance of the cysts and 4) the location (mainly in the Rt lobe-VI, VII, VIII segments).
Diagnostic ERCP was performed in all patients, as to demonstrate the location of the fistula or possible obstruction of the extrahepatic biliary tree. Nasobiliary drainage applied initially in 7 patients, stenting (10 Fr) in 4 pts, whereas in 2 others a combination of both. Definitive stopage of the output observed in 8 pts whithin five days. In 3, this happened in about 10 days and in 1 pt the initial application of the nasobiliary drainage decreased the cholorrhea remarkably, but the stopage achieved finally by stenting. Finally in one patients it was impossible to eliminate the cholorrhea neither by nasobiliary drainage nor with stenting. This patient was operated and an atypical Rt hepatectomy was performed.
The endoprostheses, stayed in position approximately 4 weeks and the nasobiliary drainage 10 days, after the final fistulae's occlusion. We did not observe any complication and the healing was successful in 92,3% of the cases.
In conclusion, the endoscopic management of the post-op high output, biliary fistulae seems to be a safe and effective method before any surgical re-intervention.

### P009

PAPILLOTOMY AND ITS CORRELATION TO TARGETING OF EXTRACORPOREAL SHOCK WAVE LITHOTRIPSY OF COMMON BILE DUCT STONES.

J.Beneš, J.Chmel, V.Dufek, J.Špičák, Č.Štuka

1st Department of Medicine, Charles University, Prague

This study was performed to assess the influence of endoscopic papillosphincterotomy (EP) on the success rate of targeting of common bile duct (CBD) stones at ESWL by ultrasound compared to X-ray. Electrohydraulic lithotriptors Medilit were used. For endoscopic extraction or mechanical lithotripsy failed. EP was performed prior to ESWL in all cases. In a group of 157 patients, X-ray targeting after contrast filling of the CBD was successful in 137 cases, USG targeting in 20. In the latter group, EP had to be extended after ESWL in 11/22 cases to enable endoscopic removal of residual fragments. In patients where USG targeting was unsuccessful and therefore X-ray location of only 3 of cases.

A large EP makes USG targeting of stones more difficult compared to an EP of limited extent, as it often causes aerobilia. It can also cause problems with X-ray targeting due to accelerated evacuation of contrast medium.

USG targeting at ESWL has certain advantages (absence of radiation and of nasobiliary drainage). Therefore, it should be always attempted as the first modality. We believe, that a EP of a limited extent should be performed in cases where the large size of the stone(s) suggests that ESWL should be preferred to an attempts of stone extraction or mechanical lithotripsy. After ESWL, an extended EP can be performed to facilitate extraction of fragments, if necessary.

### P010

LOCALIZATION OF PANCREATIC ENDOCRIN TUMORS WITH ENDOSCOPİC ULTRASONOGRAPHY

U. Yılmaz, B. Yıldırım, Ü. Sarıtaş, KB. Ateş, B. Şahin, G. Temuçin

Department of Gastroenterology, Yüksek İhtisas Hospital, TURKEY

Pancreatic endocrine tumors (PET) are rare but important disease because of it is curable when it was diagnosed accurately. Localization of the tumor is difficult with the imaging procedures used up to now, such as CT, US, scintigraphy, angiography, and venous sampling, and fails in up to 40-60% of cases. Endoscopic ultrasonography (EUS) seems to be more sensitive for preoperative localization of these tumors.

Five cases with PET were diagnosed at our EUS Laboratory in the last one year. We present these five cases. Two of 5 were female and the other 3 were male. Mean age was 29.2 years (range 21-58). Alhought one case had been performed pancreatic resection she had suffered hypoglycemic symptoms after the operation. In all 5 cases CT and US had not determined the tumor. All 5 cases had elevated C-peptide levels. Tumor localization was in the pancreatic tail in 2 cases, in the corpus in 2 cases and in the uncinate process in 1 case on EUS. Tumor diameter was between 9-15 mm. Tumor was hypoechoic in 4 cases and isoechoic in the other one. The surgeon couldn't detected the tumour in one case whose had been operated previously and had tumor in the uncinate process. In this case operative ultrasonography was performed and tumor was enucleated. Tumors were detected by surgeon in the localization as had been defined by EUS. Pancreatic tail resection was performed whose had tumor in the pancreatic tail, in other four cases tumor were enucleated. Post-operative histopathologic examination revealed PET in all cases.

EUS is more sensitive than other conventional diagnostic procedures for determining the PET. EUS bears less complication than other invasive procedures (angiography, venous sampling). EUS also provides more information for determining the lymph nodes, metastasis, venous invasion including the tumor in the gut wall.

### P011

**PERCUTANEOUS VIDEO-CHOLEDOCHOSCOPIC STONE REMOVAL VIA T-TUBE TRACT**

Gamal E.M., Rózsa I., Szüle E., Metzger P., Cserepes É., Szántó I., Kiss J.

Department of Surgery, Urology and Radiology, Postgraduate Medical School, Budapest, Hungary.

**Retained biliary stones remain a common clinical problem in patients following surgery for complicated gallstone disease, especially when Endoscopic Retrograde Cholangiography (ERC) and Endoscopic sphincterotomy (ES) is unsuccessful. During 1994 authors used percutaneous Video-choledochoscopy (PVC) through the T-tube tract in 9 patients suffering from retained biliary stones. These procedures had been performed in the Urology Department by surgeons and urologists under general anaesthesia. The time allowed for T-tube tract maturation ranged from four weeks to six weeks. Following the removal of the T-tube an instant dilatation of the T-tube tract is performed up to 25F-30F leaving a sleeve in the tract, through which the scopes are introduced. With the continuous flushing of saline a diagnostic exploration of the biliary tract is performed with a 9F flexible ureteroscope and the process of stone removal is then performed under direct fluoroscopic vision and TV monitor control using the continuous flushing and Dormia basket. Whenever difficulty of extraction is encountered a wide rigid renoscope is introduced instead of the flexible one. Following the clearance of the biliary tract a tube is re-inserted and fixed in the tract of the T-tube and through which a control percutaneous cholangiography is performed in the 2nd postoperative day. This procedure had been applied 12 times successfully, with no major complications or mortality. The authors conclude that by using VPC the surgeon may safely manage complicated biliary problems and give the chance for the patients to avoid re-operations especially in those who are not suitable for postoperative ERC and EST.**

### P012

CARCINOMA OF THE AMPULLA OF VATER. ENDOSCOPY, TREATMENT AND FOLLOW-UP OF 12 PATIENTS.

I.A. Mouzas, N. Frangiadakis, S. Potamianos, G. Alexandrakis, M. Tzardi, O.N. Manousos.
Gastroenterology Department, University Hospital of Heraklion, Crete, Greece.

The purpose of this study was to evaluate the clinical, imaging, endoscopic and management data, as well as to report the follow up of patients with carcinoma of the ampulla of Vater.

Twelve consecutive cases of ampullary carcinoma, 8 males and 4 females, aged 43-82, were diagnosed in the last 3 years in our Gastroenterology Department. In all patients were performed a duodenoscopy, a US and a CT scan.

Ten out of 12 patients were jaundiced, 4 had pruritus, 5 cholangitis, 9 anemia. Elevated levels of transaminases were observed in 9 and of γ-GT and alkaline phosphatase in 11 patients. The duration of symptoms was from one week to 14 months.

In the US, hepatic petastases were found in 2 patients, as well as in the CT scan. Additionaly, in the CT scan there was a suspicion of a ampullary carcinoma in 2 patients and of a pancreas head cancer in other 2 patients.

In 10 patients the Vater tumor was clearly visible and in 2 the infundibulum was protruding. In 7 patients duodenoscopy was completed with biopsies, in 8 with ERCP, in 5 with a sphincterotomy and in 4 with stent placement. In 3 cases the endoscopic biopsies were inconclusive, compatible with dysplasia or adenoma.

Six patients were submitted to Whipple surgery; one died within 25 days, the other 4 survived with a mean follow-up of 23 months. In 2 patients a local resection of the carcinoma was performed. Both are alive after 20 and 18 months. In 2 other patients a palliative surgical procedure was undertaken; one died within 10 days ant the other after 2 months. In 2 patients a stent was inserted endoscopically. One was alive 12 months, he was afterwards lost to follow-up, the other is still alive at 12 months.

It is concluded that in ampullary carcinoma:
1. US and CT scan are not sensitive methods in detecting it.
2. Endoscopic biopsies are often inconclusive.
3. Endoscopic palliative management is safe and effective.

### P013

SURGICAL TREATMENT OF HEPATIC HYDATID DESEASE

V.Koubishkin, R.Ikramov
A.Vishnevsky Institute of Surgery, Moscow, Russia

The results of the evaluation of early and remote results of various methods of surgical treatments of echinococcosis of the liver in 171 pts permitted us to propose principles of the choise of optimal operative technique. In our practice,we used various methods of echinococcectomy (58), excision of the echinococcal cyst together with its fibrous capsule (36), hepatic resection (25), percutaneous aspiration (8) and combinations of these methods (44) in cases with of multiple cysts. 19.7% of pts also underwent procedures on the biliary tract. In accordance with our observations,any operative technique of echinococcectomy without the excision of the fibrous capsule,even if the small bile ducts are undersewn,carries with it the risk of the development of external biliary fistulae and abscess formation,which we observed in 31% of cases. In our view this explains the advantage of excising the echinococcal cyst along with its fibrous capsule,as following such a procedure in 16% of our patients we observed only abscess formation and there were no biliary fistulae.We consider hepatic resection for echinococcosis an agressive surgery and it was necessary to perform it in the form of hemihepatectomy in 7 of our pts and as resection of 1-2 segments in 18. Percutaneous aspiration was performed in pts with increased operative risks, who had uncomplicated solitary cysts measuring up to 10 cm. The prospects of this method is evidenced by its good early effects. No deaths were observed amongst all the operated patients. During a follow-up period of 2-8 years no recurrences of disease were observed.

### P014

HSP AND ANTI-HSP IN PATIENTS WITH HYDATID DISEASE.

Paparo B.S.,Teggi A.,Teichner A.,Franchi C.,Gammaitoni A.L.,Sinopoli M.T., Leri O.
Department of Infectious Diseases, University "La Sapienza", Rome, Italy

Heat-shock-proteins (HSP) are highly conserved, immunogenic molecules produced (on the cellular surface) in response to a variety of environmental and chemical stimuli (such as bacteria, virus, parasite). The aim of this study was to investigate the stress response of Hydatidosis; in fact recent studies have demonstrated there is a chronic, immunologic stimulation in patients with hydatid cysts. We have measured the circulating antibodies to HSP-70 family (tested by ELISA according to Bahr et al 1988) in 18 patients ( 10 M/8 F range 15-60 years ) affected by liver hydatidosis. 25 healthy subjects, blood donors, age and sex matched, served as controls. The sera positive were expressed as mean±2 standard deviations (SD) of the control group, assuming as cut-off 0.69 Optical Density (OD) for HSP70 antibodies. HSP-70 Antigen (StressGen Biotechnology Corp. (Sidney, British Columbia, Canada) and hydatic antigen (collected from fluid of fertile sheep cysts) have been investigated by isoelectrofocusing (I.E.F.) as described by Righetti. Then we have performed western-blot against the sera of patients that resulted positive to HSP-70 antibodies and the serum of a healthy blood donor. HSP 70 Ab was found in 1 of 18 sera (5.5%). Western-blot showed a prominent protein band at approximately 66 kD, not observed in the control group. Our preliminary data could suggest that the presence, in infected patients, of HSP 70 Ab may be due to hydatic HSPs, which act as triggering factors in the development and persistence of the chronic inflammation.

### P015

GIANT HYDATID CYSTS OF THE LIVER

P. Vrachnos, N. Christoforides, V. Skountzos, L. Papastamatiou
2nd Dpt of Surgery. "Apostle Paul" Hosp.-KAT, ATHENS - HELLAS

Although Hellenic surgery is familiar to echinococcosis, mortality rates remain high (2-3%) depending on various factors. One of the most important factors is the big size of the cyst, causing operative difficulties, intrahepatic rupture of the cyst and intraabdominal recurrence of the parasitosis.

Fifteen patients, 9 men and 6 women aged 22-67, with giant (>20 cm) hydatid cysts of the liver were treated during the last 10 years. Pain was the main symptom, but jaundice, cholangitis, septic fever and deteriorating clinical picture in cases of intrahepatic rupture of the cyst were the causes of emergency admission. In 10 cases a mass was easily visible at the right hypochondrium. Immunologic investigations were positive in all patients and radiology, ultrasonography and CT-scans confirmed the diagnosis and determined the dimensions of the cysts. The greater diameter ranged from 32 to 21 cm. The operative technique included wide exposure of the liver through abdominal or thoracoabdominal incision, aspiration, incision and careful evacuation of the cyst, partial capsectomy or total pericystectomy sacrificing liver parenchyma up to a typical segmentectomy. Cholecystectomy, exploration of the common bile duct, removal of daughter cysts and debriments and choledochoduodenostomy in the cases of intrahepatic rupture of the cysts was added.

Suture of bile communications, drainage and omentoplasty of the liver cavity completed the operation in each case. One elderly patient died because of cardiovascular complications and another two were reoperated 4 and 6 years later because of recurrence of the disease.

It is concluded that total pericystectomy is the best operative procedure to prevent morbidity. Mortality in elderly patients remains high while recurrence is depended on contamination during aspiration and evacuation of the cyst.

### P016

OUR EXPERIENCE FROM THE SURGICAL MANAGEMENT OF MULTIPLE HYDATID LIVER DISEASE

Z.Plaitakis, D.Koumbi, Al.Zogbi, G.Panousis, G.Doulgerakis, A.Koukoulas
Second Surgical Department, General State Hospital of Nikea's "St.Panteleimon", Greece

We report our experience from the surgical treatment of 22 patients with multiple hydatid disease of the liver. Ten men and twelve women (mean age 61 years) among 374 patients who underwent surgery for hydatid liver disease during the last 22 years, were found to have multiple cysts in the liver. These were located in the right lobe in 16 cases (Group A) whereas the disease was bilobar in 6 cases (Group B). From the Group A a minor liver resection was performed in 5 cases, cysts unification and omentoplasty in 4 cases, partial or total pericystectomy of one cyst with omentoplasty of the otehr in 6 cases, and simple drainage of the cysts in one case. From the Group B partial cystectomy of one cyst with omentoplasty of the other was performed in 3 patients, lobectomies in one patient and left lobe resection with omentoplasty or partial pericystectomy with unification of the cysts was performed in 2 cases. In all patients cholecystectomy with cholangiography was performed which revealed a communication between the cysts and the biliary system in 5 cases. Common bile duct exploration was made in all these cases and migrated hydatid material was cleared from the common bile duct through a choledochotomy in 3 of them. One patient died (4.5%) during the immediate postoperative period and the morbidity was 18%. The mean hospitalization time was 30 days. In conclusion, multiple hydatid liver disease constitutes an uncommon clinical entity and the surgical intervention requires experience since cystobilliary communications often exist.

### P017

SURGICAL TREATMENT OF HEPATIC HYDATIDOSIS

M.Stojiljković,M.Jeremić,M.Stojanović,

V.Pejčić,A.Bogićević

Surgical clinic.Clinical center-Niš,Serbia

Yugoslavia

The aim of this study is to analyze our results in the surgical treatment of hepatic hydatidosis.Beetween 1989 and 1993 89 patients with hepatic hydatidosis were operated. Demographic characteristic are:male/female 37(41%)/52(59%),mean age 48,3 yrs.Main clinical symptoms and signs were pain in the right hypochondrium(7o%) and hepatomegaly(33,6) Basic diagnostic methods were:ultrasonography(95,6%)and computer tomography(26,3%).Complicated cysts were noted in 15(16,85%)patients in the form of supuration,rupture in the peritoneal cavity and biliary tract,etc.Surgical techniques performed were partial cystopericystectomy(in 65 patients i.e.73,3%) total cystopericystectomy(in 19 patients,ie 21,34%)and liver resection in 5(5,6%).Other associatedoperations were cholecystectomy, choledochotomy,sphincteroplasty and so on in 32(35,95%).The most frequent complications were: biliary fistulas in 7 cases,infection of the cyst cavity and perihepatic areas (5 cases) and pleural effusions(4 cases).The average time of hospitalization was 12,6 days. There was no mortality.

Our results suggest that every diagnosis,before the appearance of complications,with the use of current surgical techniques allowed successful treatment of the disease.The best results,when it is possible,give total cystopericystectomy.

### P018

THORACIC COMPLICATIONS AFTER SURGERY FOR LIVER HYDATIDOSIS.

G. Chamalakis, S. Kakaris, I. Scotis, B. Daras, A. Takas, E. Salemi
A' Thoracic Surgery Clinic "Sotiria Hospital".

The rupture of the echinococcus cyst of the upper surface of the liver to the hemithorax or the development of an hepato-bronchial fistula are conditions unusual but rare that need immediate diagnosis and surgical restoration.

The last two years we treated two pulmonary surgical complications in our department after rupture of an echinococcus cyst of the upper surface of the liver.

The first case was a 68-years old farmer whose main complaint was a productive cough with billious expectoration. He had had a CT of the upper abdomen which revealed a cystic mass in the upper surface of the right lobe of the liver with peripheral calcification and a small shadow in the adjacent part of the lower lobe of the right lung. Because of the history this caused suspicion of bronchial communication of the echinococcus cyst and the base of the right lung.

The patient was operated on through a right thoracotomy the fistula was resected and the echinococcus cyst was extracted through the diaphragm.

The second case was a 30-years old builder who presented in the outpatient's clinic of our department urgently under shock. The x-ray of the chest revealed collection of pleuritic fluid. The patient was urgently intubated under local anaesthesia of the right hemithorax and a quantity of about 1000cc of purulent liquid was collected. The patient's condition was automatically improved.

The CT of the upper abdomen revealed an echinococcus cyst of the upper surface of the liver that was ruptured to the hemithorax. He had had a right thoracotomy, the echinococcus cyst was resected through the diaphragm and the cystic wall was closed, using a "capitonage" technique. Both cases are doing very well now.

### P019

### P020

*RİSK FACTORS AFFECTING SURGERY FOR HYDATID DISEASE OF THE LIVER*

*Yıldıray YÜZER, Hakan ÇEVİKEL*, Murat KILIÇ*, Ahmet ÇOKER, Ali MENTEŞ, Mustafa KORKUT*

*The HPB Surgery Unit, Department of Surgery*,AegeanUniversity Faculty of Medicine, Izmir, TURKEY*

A retrospective study was planned to identify factors that may be responsible for complications that arising after for the surgery for the hydatid disease of the liver. Data were obtained from the hospital records of 387 patients that were operated at the Aegean University Hospital, for hydatid disease of the liver during the last decade. Twenty-five variables determined during the preoperative period were evaluated in a multivariate analysis. The nature of the cyst content and the type of operation performed were added as operative variables.

Patients having hydatid cysts of the liver were treated with simple drainage in 35 cases, with omentoplasty in 248 cases, with introflexion in 61 cases, and with cystectomy in 43 cases. Mortality was observed in 6 (1.6 %) patients mainly due to coagulation disorders (2 patients), biliary sepsis (2 patients) and coexisting medical diseases such as cardiac and renal failure. Major complications were encountered in 24 ( 6.2 %) patients, (mainly bile fistula, wound disruption, pneumonia) and minor complications were observed in 16 (4.1%) patients (wound infection, pleural fluid collections, urinary tract infection). The overall morbidity was 10.3%. Higher serum bilirubin levels ($p<0.001$), the presence of ascites ($p<0.001$), bilober hydatid disease ($p<0.001$), multipl cysts ($p<0.001$), extraabdominal cysts ($p<0.01$), and coexisting medical diseases($p<0.01$) such as chronic lung disease, and cardiovascular diseases were risk factors likely to lead to complications during the postoperative period. The type of the operation performed and the nature of the content of the hydatid cyst had no predictive effect on morbidity.

The important variables forthcoming from the multivariate analysis suggest that the intrahepatic extension of the disease besides the functional reserve entrapped by the hydatid disease has an important role on the postoperative period. Combined treatment strategies may be reasonable in patients with such advanced hydatid disease states.

### P021

THORACOPRHENOLAPAROTOMY FOR SUPERIOR EDGE OF 10[TH] RIB IN THE TREATMENT OF HEPATIC HYDATIDOSIS

*T.Butrón, M.Diaz Tie*, A. Garcia Carranza*,S. Mallagray**
*I Department of General and Digestive Surgery. 12 de Octubre Hospital. Madrid. Spain.**Department of General Surgery. Cruz Roja Hospital. Madrid. Spain

Hepatic hydatic cyst are located more frecuently in the right lobe, being in more than 60% of the cases in posterosuperior segments. Radical surgery is the most effective. We proposed the thoracophrenolaparotomy for superior edge of 10[th] rib (TPL10) how suitable surgical incision for hydatic cysts of that location.
MATERIAL AND METHODS: Prospective study of 48 patients operated on from 1985 to 1989 with a follow-up of at least 5 years. The cysts were located in VI, VII, VIII segments of the liver. There were uniques or multiples, complicated or not. All were operated on by a TPL10 performing radical techniques as a close or open total pericystectomy, hepatectomy or partial pericystectomy. We show the technique in the pictures.
RESULTS: The mean age was 48.3 years, wiht 56.4% men and 43.5% women. Postoperative mean stay was 18 days (10 - 60 days). A total of 78 cysts from 5 to 30 centimeters of size were present in the 48 patients. We performed total pericystectomy or hepatectomy in 85.3% and partial pericystectomy in 14.5%. Morbidity was 12.5% (6 patients), subphrenic abscess (2 patients), wound infection (1 patient), pleural suffusion (1 patient), residual cavity infection (1 case), pneumonia (1 patient). All patients are fine after 5 to 9 years from surgery, without recidivation. Combined techniques were done in 8 patients (16.7%), cholecystectomy (3 patients), cholecystectomy with T-tube biliary drainage (1 patient), splenectomy (2 patients), pulmonary lobectomy (1 patient), pleurectomy (1 patient), cystectomy of simple renal cyst (1 patient).
CONCLUSIONS: TPL10 allowed perform radical techniques in all cases with both low morbidity and postoperative hospital stay. It was a good incision to perform combined techniques. TPL10 seems to be a suitable incision for posterosuperior hepatic hydatic cysts, especially those of large size.

### P022

INTRAHEPATIC RUPTURE OF HYDATID CYSTS

L. Lapidakis, D. Kalokerinos, D. Xypolytas, L. Papastamatiou
2nd Dpt of Surgery "Apostle Paul" Hosp.-KAT ATHENS-HELLAS

One of the most severe complication of liver echinococcosis is the intrahepatic rupture of the cyst. Six such cases,out of 34 patients with liver hydatidosis are presented. Two patients were subjected to two interventions.

Pain, aphylaxia, jaundice, cholangitis, septic fever and deteriorating clinical picture were the main causes of the emergency treatment. Diagnosis was based on history, laboratory findings and X-Ray examination, including scintigraphy, ultrasonography and computed tomography appearance.

The operative technique is rather standardised:

- Wide exposure of the liver through abdominal or thoracoabdominal incision.
- Recognition, paracentisis, incision and evacuation of the cyst(s).
- Partial capsectomy, sacrificing liver parenchyma or, in lateral location of the cyst, atypical segmentectomy.
- Cholecystectomy.
- Peroperative cholangiography. Exploration of the common bile duct. Removal of daughter cysts and debriments.
- Wide side to side choledochoduodenostomy.
- Suture of bile communications, drainage of the liver cavity and omentoplasty.

No mortality in these series was observed. Morbidity rates were high: 4 complications in 3 patients (66,6%).

It is concluded that hepatic hydatidosis and its complications consists still a surgical problem, with long hospitalisation time mainly due to postoperative complications. In elderly, mortality rates remain high, underlining the severity of this parasitosis.

### P023

ULTRASOUND APPEARANCE OF LIVER HYDATID CYSTS BEFORE AND AFTER CHEMOTHERAPY

G.Mechkov, T.Todorov, T.Todorova
Clinic of Internal Medicine, Fifth Clinical City Hospital, Sofia, Bulgaria

Ultrasonography is considered to be a very useful diagnostic technique in investigation of the liver and allows direct visualization of hydatid cysts in this organ. The purpose of our study was, using this diagnostic method, to describe the morphological appearance of hepatic hydatid cysts and to define the changes following chemotherapy. The sonogaphic images of hydatid cysts in the liver were studied in 67 patient with echinococcosis. Thirty one patients were treated with albendazole and 36 with mebendazole. The hydatid cysts were examined before initiation of chemotherapy to analyse the ultrasound scans and to classify the patterns of cystic lesions. The changes in ultrasound appearance of the cysts undergoing medical treatment were evaluated during therapy and follow-up period. Before therapy four types of cyst appearance were observed. The greatest number of cysts were more or less spherical in shape with fluid collection and completely anechoic. A limited number of cysts were also with fluid collection but with split walls, causing detachment of membrane, other showing multicystic appearance due to development of daughter cysts. The fourth type of cysts, less frequent, was characterized by hyperechoic appearance with thick and calcified walls. Following chemotherapy anechoic cysts became hyperechoic. Separation of endocyst from ectocyst was observed, producing an ultrasound water lily sign. Cyst size reduction and/or deformation occurred later, predominantly in smaller cysts. Some cysts with daughter cyst, with mixed echostructure, later increased their echogenicity due to rupture of daughter cysts. The most important findings was disappearance of the cysts from liver parenchyma. No change in cyst morphology was established in about one fourth of the cysts treated. Ultrasonography is a well established technique for the diagnosis of liver hydatid disease and may be very useful in evaluation the response of liver cysts to chemotherapy.

### P024

THE TREATMENT OF ECHINOCCOCAL CYSTS IN THE EXTRAHEPATIC BILIARY TRACT DIAGNOSED AFTER THE INITIAL OPERATION FOR LIVER HYDATID DISEASE.

Tsantilas D, Giakoustidis E., Frankandreas G., Fouzas I.,Ouzounidis N. and Papazoglou O.
5[th] Surgical Clinic,Hippokration General Hospital, Department of Surgery, Aristotelian Univesity of Thessaloniki Medical School Greece.

The rupture of echinococcal cysts into the biliary tract is a common and severe complication of liver hydatid disease. There is a number of patients in whom the presence of the cysts is not diagnosed during the initial operation. Five such patients were referred to our Clinic and were succefully treated. Three patients, 2-3 years after the initial operation, presented with biliary colic and jaundice The echinococcal cysts were found during the biliary tract exploration and they were treated by T tube placement and continuous infusion of hypertonic saline in the biliary tree or choledochoduedonostomy. The fourth patient was operated for choledocholithiasis and the presence of the primary cyst was diagnosed by T tube cholangiography and was reoperated and submitted to evacuation of the cyst and omentoplasty. The fifth patient was referred for a biliary fistula and the diagnosis was established by ERCP and was treated by sphincterotomy and continuous infusion of hypertonic saline through the fistula. CONCLUSIONS: 1) Undetected rupture of echinococcal cysts is a serious complication, whith many diagnostic problems. 2) Diagnosis is established either by ERCP or intraoperatively 3) Treatment of the residual daughter cysts in the bile ducts is achieved either by ERCP and sphincterotomy, or operatively by T tube placement or choledochoduedonostomy and further continuous infusion of hypertonic saline in the biliary tree.

### P025

**ARE SCOLICIDAL AGENT SOOKED SPONGES EFFECTIVE IN HYDATID CYST SURGERY ?**

**K.Karayalçın, H.Besim, O.Hamamcı, A.Korkmaz**

**Sixth Department of Surgery, Ankara Numune Hospital, Ankara, Turkey.**

Avoidance of intraoperative spillage is fundemental for the successfull management of hydatid disease. Any failure will result in dissemination of the protoscoleces with severe sequale. One of the most commonly recommended and employed measure to prevent dissemination is to pack the operating field with sponges soaked with scolicidal agents. However its effectivity has never been investigated clinically or experimentally.
In an invitro model we tested the efficacy of scolicidal agent soaked sponges. 1x1cm pieces of sponge were cut and soaked with hypertonic saline (3%,10%,20%), hydrogen peroxide, povidone-iodine 10%, ethyl alcohol 95% and normal saline as a control. A drop of scolex rich hydatid fluid obtained from a public slaughterhouse was sprayed on sponge pieces. After 15 minutes they were put into test tubes filled with PBS and shaken vigorously. After sentrifugation, the sediment was placed on a slide and stained with 0.1% Eosin in order to determine protoscolex viability. Living protoscoleces do not take up the dye.
Sponges soaked with hypertonic saline(20%), ethyl alcohol, povidone-iodine and hydrogen peroxide were found to be effective in terms of killing the scoleces. Hypertonic saline(3%,10%) and control group were found ineffective.
The results of this experiment showed that scolicidal agent soaked sponges work not only as a mechanical barrier but also an effective measure to prevent dissemination if the scolicidal agent is chosen correctly.

### P026

CURRENT TREATMENT OF LIVER ECHINOCOCCOSIS

C.Fotiadis, A.Macheras, D.Mandrekas, T.Liakakos, K.Giafis, G.Papastratis, M.N.Sechas.
3rd University Surgical Dpt, Athens University.

The aim of this study is to present the experience of 3rd University Surgical Dpt by 11 cases of echinococcosis (9 granulosis, 2 alveolaris) from March '93 to November '94. We present diagnosis, surgical intervention, complications, postsurgical treatment and follow up. For all cases we made omentoplasty, in one case cystectomy and in one case open evaluation and intubation. Only one patient relapsed one year after. The patients received albendazole per os before and after operation. In conclusion omentoplaty is a safe method for therapy in case of liver echinococcosis.

### P027

*INTRAOPERATIVE ENDOSCOPIC DIAGNOSIS OF CYSTOBILIARY COMMUNICATION*

*Ali MENTES, Yıldıray YÜZER, Ahmet ÇOKER, Mahir AKYILDIZ*, Sinan ERSİN**

*The HPB Surgery Unit, Department of Surgery*, Aegean University Faculty of Medicine, Izmir, TURKEY*

Biliary communication is the most frequently reported complication of hydatid liver cyst. Although routine preoperative work-up reveals frank biliary rupture in most instances, unlocated biliary leaks into the cyst space frequently causes problematic postoperative complications. Intraoperative detection of such leakage points is always based on visual inspection of the cavity after evacuation of the cyst contents. However, in patients harbouring cysts at unsuitable (atypical) locations for direct inspection, decisions on surgical treatment modalities may be blindfolded. We report eight patients with hydatid liver cysts at atypical locations who were evaluated by intraoperative endoscopic evaluation of the hydatid cyst cavity for the diagnosis of cystobiliary communication. Four patients had cysts at posterior diaphragmatic locations at the right lobe, two patients had cysts deeply seated within the liver substance intraparenchymally and one patient had a medium sized cyst under the portal pedicle. Another patient had a deep recess at the wall of cyst, extending into the liver proper which did not allow direct inspection. Five of the patients had infected cysts, one patient had bile stained cyst contents and one patient had a history of anaphylaxis. In none of the patients was a biliary communication demonstrated by preoperative diagnostic studies. Intraoperative endoscopy revealed biliary communication in three patients and suggested biliary leakage in another. The success rate of intraoperative intracystic endoscopy was 100%. There were no complications.

### P028

**The Albendazole role in the treatment of hepatic hydatidosis. A surgical evaluation.**

MUSELLA M*, LOINAZ C, GOMEZ R, PINTO I, ALVARADO AA, CHAMORRO A, PETRELLA G**.
Department of General and Digestive Surgery and Abdominal Organs Tranplantation-University Hospital "12 de Octubre" Madrid-Spain. *CNR-ESTERO. **Naples University-Italy.

From January 1988 to December 1994, 31 patients with hepatic hydatidosis have been treatad in our center, 18 males and 13 females. The mean age was 52.7 years, the range 20-66 years, 70.3% were solitary cysts. A bilateral subcostal incision was preferred, in only three pts. with a single left lobe cyst a mid-line incision was performed. One patient with right lung involvment needed a thoracic extension of the abdominal incision. The most common symptoms were related to the presence of an abdominal mass. In seven patients the cyst(s) were incidentally discovered while undergoing ultrasound for presumed gallbladder pathology. Fever and abdominal pain were often present. All pts. received a preoperative ultrasound. A CT-scan, HIDA and arteriography were used as well. In regards to the preoperative therapy we administred mebendazole in 4 patients, **albendazole in 24** and nothing in three pts. Albendazole treatment consisted of three cycles of 28 days each; there was a medication-free period of 14 days beetween cycles. A CT-scan was performed at the beginning and at the end of the albendazole therapy, an ultrasound evaluation was performed between the cycles. In seven pts. we noticed a direct involvment of the extrahepatic biliary tract via a fistula evaluated intraoperatively with cholangiography. Five of these patients had jaundice caused by the common bile duct being obstructed by hydatid vescicles. In all patients we performed a total cystopericystectomy. In 18 pts. the cyst was removed intact. A hepatic resection was never necessary. One patient died (N° 21) in the postoperative period due to hepatic insufficiency, his old age and a significant intraoperative blood loss. Three pts. developed biliary fistulas (pts. N° 15, 21 e 29) while in another case a large subphrenic abscess forced us to reoperate on the patient. We had no disease recourrences. The mean hospitalization period was 17.2 days. Albendazole gave us two main advantages: a) a preoperative parasiticyde effect b) a complete excision of the cyst wall from the parenchyma was made easier.

### P029

SURGICAL TREATMENT OF ALVEOLAR ECHINOCOCCOSIS OF THE LIVER

O. Bilge, A. Emre, K. Acarlı, A. Alper, Y. Tekant, Y. Çakaloğlu*, A. Ökten*, O. Arıoğul.

Hepatopancreatobiliary Surgery Unit and *Hepatology Division, University of Istanbul Medical Faculty, Istanbul, Turkey.

The data on 37 alveolar echinococcosis patients who attended the Hepatopancreatobiliary Surgery Unit of the University of Istanbul Medical Faculty between January 1979 and December 1994 were reviewed. These cases constitute 7% of all echinococcosis patients treated at the unit during the same period. The operative procedures were radical resection in 8 patients (22%), debulking surgery in 6 (16%) and biliary diversion in another 6 (16%). No intervention beyond exploratory laparotomy was possible in 10 cases (27%). Surgical exploration was not performed in 7 patients (19%) with obviously inoperable lesions. One patient, in whom the lesion had infiltrated the vena cava, the right and middle hepatic veins, died perioperatively following resection due to uncontrollable haemorrhage. There was no recurrence in the other 7 patients who underwent radical resection (follow-up range: 2 months-5 years). Five patients who underwent a biliary diversion were asymptomatic while one was lost to follow-up. Nine patients were lost to follow-up and 7 died during this period. Long-term albendazole treatment was given to all the patients in whom radical resection was not possible.

Radical surgery is the only chance for cure in this lesion which behaves like a slowly progressing malignant tumor. Unfortunately, this is frequently impossible due to delays in diagnosis. Medical therapy should be preferred only in the inoperable cases.

### P030

LAPAROSCOPIC TREATMENT OF HEPATIC HYDATID DISEASE

A.Alper, A. Emre, H. Hazar, İ. Özden, O. Bilge, Y. Tekant, K. Acarlı, O. Arıoğul

Hepatopancreatobiliary Surgery Unit, University of Istanbul Medical Faculty, Istanbul, Turkey

In this report, a laparoscopic method for the treatment of hepatic hydatid disease is described and the results in the first 24 cases (median age 32 years, range: 13-66) are presented. The method involves the use of an aspirator-grinder apparatus designed specifically for laparoscopic surgery. The method achieves effective evacuation of viable cyst contents with the benefits of laparoscopic surgery. Mean hospital stay was 8 days (range 2-16). Cavity infections (5 patients) and external biliary drainage (4 patients) were the main postoperative complications which were treated by percutaneous cavity drainage and endoscopic papillotomy. Two patients required open surgery.

Our experience suggests that laparoscopic surgery of last-stage, thick and calcified walled cysts are prone to serious complications. These patients should be excluded from laparoscpopic treatment . Biliary communications and incomplete collapse of the cavity are the main reasons for complications. The method is particularly suitable for uncomplicated, early-stage cysts located in laparoscopically accessible positions. Early postoperative parameters and follow-up results (up to 25 months) are encouraging in selected patients.

### P031

SURGICAL TREATMENT IN HEPATIC HYDATID DISEASE

PINHEIRO, M.J.;GIRÃO, R; MENDES,M.;SCHIAPPA,J.M.; GIRÃO, J.;

HOSPITAL DE SANTO ANTÓNIO DOS CAPUCHOS
SERVIÇO 6
LISBOA - PORTUGAL

From a series of 60 patients affected by 73 hydatid cysts, 61 of which were liver cysts,the AA evaluate some features dealing with pathology, clinical manifestations and surgical treatment.
The importance of the imagiology of the cysts achieved by CT Scan and ERCP, in order to assess the situation and possible biliary fistulae, is stressed.
The AA discuss the rational for the surgical approach and enhance several technical procedures done in this series.
The analysis is done from a follow up ranging from 3 to 20 years and it is concluded that the treatment of this condition may need , in many instances, the referral to a special HPB unit.

### P032

Surgery of biliocystic communications in hepatic hydatosis.

F. Martin Vieira, I. Moreno, R. Garcia, A. Lopez, A. Calvo, L. Lozano, J. Guadarrama, M. Lomas, T. Butron, F. Botella.
Servico Cirugia Digestivo B.Hospital 12 de Octubre. (Profesor M.R. Vilariño)Madrid. Spain.

The frecuency and morbimortality rate of biliocystic communications(BC) render them of great importance in hepatic hydatosis.
Review of our experience with 216 patients operated by hepatic hydatosis over the last 17 years, with 59 cases of BC(27,3%). The BC were clasified according to the moment of the diagnosis in intraoperatives and postoperatives and were analized the age, the sex and the morbimortality according to the surgical tecnique used over the cyst and over the biliary tree.
The BC were diagnosed during the operation in 44 cases and on the posoperative in 15 cases.
On the intraoperative BC the tecnique over the cyst with most posoperative fistule was the marsupialization over tube (70%) and the tecnique with least fistule was the cystepericystectomie total (9%). The tecnique over the biliary tree with most posoperative biliary fistule was the only suture of BC(24%) and the tecnique with least fistule the sphincterectomy(0%).
On the BC posoperatives, the fistule is presented after: Hepatectomie(0%),Cystepericystectomie total(1%),Cystepericystectomie partial(6%),Marsupialization(25%).
The mortality rate in our serie was of 1.6%.
Conclusions: Over the cyst the best surgical tecnique was the cystepericystectomie total, over the biliary tree the best results were obtained when the sphincterotomy was realized.

### P033

TITLE: UNUSUAL LOCATIONS AND TYPES OF ECHINOCOCCOSIS

RIGHTERS: K.FOTIADIS.N.KAKAVIATOS.K.GIAFIS.D.PAPAIOANNIDES.
D.MANDREKAS.N.DOVAS.M.SECHAS.
3rd and 2nd UNIVERSITY SURGICAL DPT. ATHENS UNIVERSITY SCHOOL OF MEDICINE.

The aim of this study is to Present the experience of 3rd Surgical Dpt on seven cases of Unuscal Locations of exhinococcosis. We present four cases of echinococcosis of the Liver and the spleen one case of multiple echinococcosis of the liver, the spleen and the left femur.
One case with echinococcosis of the liver and thyroid gland and one case of echinococcosis of the liver and the scapyla as well. Also we present two cases of echinococ. alveolaris. Diagnostic methods surgal technique that we used and post operative follow up are presented.

### P034

SURGICAL OPTIONS IN MANAGEMENT OF HYDATID CYST OF THE LIVER

R.Yalın, A.Ö Aktan, R.İnceoğlu, C.Yeğen, M.Ghaemi.
Marmara University School of Medicine, Dept.of General Surgery, İstanbul, Turkey.

Turkey is one of the countries where hydatid cysts are endemic.While surgery is still the primary treatment for hepatic hydatid cysts, a variety of approaches have been described. In this retrospective study, different surgical techniques in management of hepatic hydatid cysts were compared. 106 surgical procedure were carried out in 96 patients with hepatic hydatid cyst. The most common complication of hydatid cyst was biliary rupture (18.3%), followed by infection of the cyst cavity (5.5%).
Omentoplasty was carried out for uncomplicated cysts (37.7 %) with low morbidity (19.5%) and short hospital stay (mean 12 d.) External tube drainage was carried out in 28.4% of patients. The morbidity rate was 74.1% and the mean hospital stay was 19.8 days. Partial cystectomy and introflexion was performed in 20.1% patient. There was no mortality.
Conclusion: Omentoplasty is the procedure of choice for uncomplicated cysts with a low complication rate and relatively short hospital period. External tube drainage is recommended for infected cysts and biliary drainage procedure must be added to external tube drainage for cysts with intrabiliary rupture. Omentoplasty is easy to perform and is a good way of obliterating the cyst cavity.

### P035

ULTRASOUND APPEARANCE OF HYDATID DISEASE OF THE LIVER

I. Spathopoulos, T. Pavlidis, D. Kouvelas, S. M. Janković
Second Department of Surgery, Agios Pavlos Hospital, Thessaloniki, Greece

The hydatid disease of the liver has a characteristic variable ultrasound(US) appearance depending on some well determined factors. The aim of this study was the presentation of various interesting pictures emphasising on differential diagnosis(DD) between hydatid disease and other liver diseases. Our study indicated that echinococcal cyst might be seen on US as: (a) Simple,unilocular cyst. Differentiation from solitary non parasitic cyst is impossible. (b) The usual picture of cystic multiloculated lesion due to contained multiple daughter cysts. DD from cyst adenoma. (c) Impact lesion because of ablation of membranes from the cyst wall. DD from hepatoma, metastasis, adenoma and focal nodular hyperplasia, abscess and hematoma. (d) Partial or complete calcification of the cyst wall. The latter indicates inactive disease. (e) "Crescent sign", when the ablation of the laminated membrane(endocyst) from the adventitia(ectocyst) is partial and local; "floating water-lily sign",when it is more extended; "cyst into cyst" when it is complete. (f) Thickened wall and impact lesion,because of infection and abscess formation. DD as in (c). In conclusion, it seems that US is a simple, safe and inexpensive diagnostic tool,which could evaluate all the variety of the disease giving useful information.

### P036

LAPAROSCOPIC MANAGEMENT OF ABDOMINAL CYSTS

V. Pegan, M. Sever, A. Pleskovič, A. Perovič,
Department of Gastroenterologic Surgery, University Medical Center, Ljubljana, Slovenia

In 1994, 8 patients underwent laparoscopy for cysts in the abdominal cavity; there were 2 males and 6 females (mean age 43 yrs and 57 yrs respectively). Five patients had one or two cysts (congenital) in the liver, one had a parasitic liver cyst, while the remaining two patients presented with a splenic cyst and a diaphragmatic cyst respectively.
All the above mentioned cysts were diagnosed by ultrasonography. Three patients were symptomatic, and two of them were admitted for symptomatic gallstones.
The aim of the study was to determine the advantages and efficacy of laparoscopic treatment. Unroofing of the cyst was performed in 7 patients. In one patient total pericystectomy combined with cholecystectomy was accomplished laparoscopically.
The recovery of all 8 patients was ineventful. They were discharged from hospital on the 4$^{th}$ postoperative day. Follow-up ultrasonography 2-6 months after surgery showed a recurrent cyst in 5 patients while two were without signs of recurrence.
In summary, the recurrence rate in our series of laparoscopic treatments for abdominal cysts was 62%. Therefore, we have to conclude that the indication for laparoscopic treatment is rather questionable even in symptomatic cysts. In comparison with fine-needle aspiration, the laparoscopic method enables us to investigate histologically the excised roof of the cyst. This possibility could be regarded as advantage in suspicious cases.

### P037

CORRECT TIMING FOR LOCAL ANESTHESIA IN LAPAROSCOPIC SURGERY

A.Ö.Aktan, A.M.Saraç, C.Yeğen, R.İnceoğlu, Ö.Günal,R.Yalın

Marmara University School of Medicine Department of General Surgery, İstanbul, Turkey.

Although postoperative pain in laparoscopic cholecystectomy (LC) seen much less than open surgery it increases postoperative morbidity and complications. The aim of the study is to find out whether local anesthetic (LA) infiltration of the trocar sites during LC could decrease postoperative pain and also to find out the correct timing for LA.
Seventy patients undergoing LC were randomized into three groups: The first group (n=25) as the control and 3 cc. 0.9% NaCl was injected around the each of 5 mm.trocar sites and 4 cc around I0 mm. trocar sites subcutaneously.In the second group (n=20) the same volume of LA (Bupivacaine 5%) was injected at the beginning of the operation. In the third group (n=25) LA infiltrated at the end of operation. Visual Analog Scale were given to all patients and asked to record their pain intensity postoperatively. Pain intensities were checked on the I.3.5.7 and I2th hours and Petidine HCl I mg/kg im were done whose pain intensities greater than five. As the results are compared, in the preoperative LA group 50% of patients and 28% of patients in the postoperative LA group required analgesics. This number was 76% in the control group. The mean pain intensities were 5.9, 5.I and 7.6/I0 respectively. There were significantly lower pain intensities and analgesic requirements in postoperative LA group. LA groups had lower pain intensities and Petidine requirements than the control group in the early postoperative period.

### P038

LAPAROSCOPIC VERSUS OPEN CHOLECYSTECTOMY IN CIRRHOTIC PATIENTS

M.A. Yerdel, I. Alacayir, S. Demir, N. Aras, H. Tsuge.
Ankara Univ. Med. School, Dept. of Surgery, Ankara-Turkey and Okayama Univ. Med. School, 1st Dept. of Surgery, Okayama-Japan.

Open cholecystectomy (OC) in cirrhotic patients is associated with high morbidity and mortality owing to the increased rate of infectious, haemorrhagic and incision related complications. In this study we compared the results of laparoscopic cholecystectomy (LC)(n:6) versus OC (n:6) which were performed consecutively in a 3 year period in patients with proven cirrhosis. Groups were well matched for age, sex, and Child's class. LC was performed with Zucker's technique and OC was performed through a right subcostal incision. There were no mortality in either group. In LC group; mean operating time was 133 min., and average amount of operative blood loss was 150 cc. No patient in LC group required blood component therapy during or after the operation. Mean hospital stay in LC group was 6 days and no complication was encountered. In OC group; mean operating time was 100 min., and average amount of operative blood loss was 400 cc. 0.66 U/patient blood transfusion was required in OC group. Mean hospital stay in OC group was 16 days. Wound infections necessitating drainage occurred in 3 patients (50%) in OC group. One of these patients had wound dehiscence which resulted in an incisional hernia. LC seemed to offer serious advantages over OC in cirrhotic patients. Avoiding the incision lowers the amount of blood loss, minimizes if not eliminates the risk of wound related complications such as infection, dehiscence and contamination of the ascites. In conclusion; contrary to previous belief of many authors; we think that cirrhosis per se is definitely not a contraindication to LC if OC is the alternative. LC should be the procedure of choice whenever cholecystectomy is indicated in a patient with cirrhosis.

### P039

FIRST STEP IN LAPAROSCOPIC CHOLECYSTECTOMY CRITICAL APPROACH

D.Niculescu, E.Târcoveanu, St.Georgescu, C.Bradea, D.Vintila

Frist Surgical Clinic

University of Medicine and Pharmacy Iasi

In Mars 1993 the first laparoscopic cholecystectomy has been performed in our clinic and in the next 18 months we performed.
219 such operations. In the same period had been performed 355 classic cholecystectomy.
In 21 observation (10 acute, 11 chronic) the laparoscopic procedure was converted to open cholecystectomy. The comparative study of the 2 groups prove a lower rate of hospital stage after laparoscopic approach but the cost can't be accourately compared (because repaing of investition in equipment for laparoscopic approach).
The higt rate of connversion characterize the begining of laparoscopic cholecystectomy and we have done it because intraoperative incidents: haemorrhagic - 3, GB rupture - 2, cystic lithiasis - 2, gangrenous cholecystitis - 1, plastic pericholecystic peritonitis - 7, section of right hepatic duct - 1, miscellanons - 5.
Thare are 42 cases with subhepatic adherences, 18 cases with a difficult disection of cystic artery due to previous inflamations , 25%, CBD fissure, 20%. Haemorrhagie 10%, GB rupture with intraperitoneal stone loss, 2 CBD lesion, 1 intraperitoneal hematome, 1 ileal fistula due to trocar lesion and 1 death (brain haemorrhagie).
Comparing the resuts of both methods laparoscopic cholecystectomy is more economically, with similar rate of complication but not for the untrained surgeon, in our report the operation time decrease from 100 to 60 minutes and also the number of reconverssion.

### P040

**LAPAROSCOPIC MODIFIED SUBTOTAL CHOLECYSTECTOMY**

Palanivelu C. MS,MCH., Rajan P.S., Mahesh Kumar, *Department of Surgical Gastroenterology, Coimbatore Medical College Hospital, Coimbatore-641 018, INDIA.*

Though with increasing experience in Laparoscopic Cholecystectomy open conversion rate is coming down, still standard technique needs to be improved to treat difficult cases without increasing morbidity and mortality.

From July 1991 through October 1994, 1250 patients with symptomatic cholelithiasis were treated by Laparoscopic method. The standard technique was modified to subtotal cholecystectomy for successful laparoscopic management in 77 patients with following risk factors : (A) Cirrhotic Liver - 31; (B) Portal Hypertention - 14; (C) Inflammatory Phlegmon - 18; (D) Extensive Pericholedochal Fibrosis-11; (E) Malposition of Gall Bladder-3.

Subtotal Cholecystectomy was performed in two ways.
Type-1 : Difficult GB bed - Posterior wall was left intact, mucosa was either peeled off or cauterised.
Type - 2 : Difficult Hilum - Infundibulum was divided and the flap was sutured to cover the neck after mucosal excision or cauterisation.

By this modification undue bleeding or CBD injury was avoided. Post operative morbidity, hospital stay and return to routine work were similar to standard laparoscopic cholecystectomy.

Policy of keeping away from danger zone is safe and modified cholecystectomy makes laparoscopic management of difficult cases practicable without increasing morbidity and mortality.

### P041

### LAPAROSCOPIC CHOLECYSTECTOMY : RISKS OF ASSOCIATED COMMON BILE DUCT STONES

S. Rizos, S. Skaltsas, B. Kekis, E. Pagounis, S. Minokakis, I. Xanas, Th. Megas.

Second Department of Surgery, 1st Hospital IKA, Athens, Greece
Department of Surgery, Agii Anargiri Hospital, Athens, Greece

Patients with sympomatic gallbladder stones may have an associated common bile duct (CBD) stone. The purpose of this study was to define this risk according to the presence or not of preoperative suspected signs. All laparoscopic cholecystectomies were included in the study. Patients with abnormal preoperative liver function tests were considered at high risk of associated CBD stones. From Jan. 1991 to Nov. 1994,1250 consecutive patients had a laparoscopic cholecystectomy. There were 370 males and 880 females.The mean age was 47 years.1090 patients had a biliary colic,111 acute cholecystitis and 4 gallbladder polyps. 63 patients ( 5% ) who had a history of biliary colic or jaundice and abnormal LFTs or dilated CBD on U/S underwent a preoperative ERCP. 11 patients had normal preoperative liver function tests. However, the ERCP detected 5 stones. In all patients with calculi ( n= 40 ) endo scopic stone removal was successfully performed with sphincterotomy. Morbidity was 1.6% after ERCP. Intraoperative cholangiography was attempted in 130/1250 ( 10.4% ) patients and was successful in 112/130 ( 86.2% ). Unsuspected CBD stones were found in 18 patients. Stones were removed intraoperatively using a small choledochoscope through the cystic duct in 1 patient. ERCP and EST were performed in 17 patients with successful stones removal in all. CBD access was possible in 3 patients after a needle-knife papillotomy and nasobiliary tube was used in 7 patients. One patient developed mild pancreatitis which was treated conservatively. 52 patients had abnormal preoperative liver function tests.
It is concluded that normal liver function tests reduce the risk of associated CBD stones (p=0001) without reducing it to nothing. Pre- or post-operative ERCP, EST and CBD clearance combined with Laparoscopic Cholecystectomy is a safe and effective treatment in patients with gallbladder and CBD stones.

### P042

### OPERATIONS FROM MINIMAL ACCESS IN TREATMENT OF PATIENTS WITH ACUTE CALCULOUS CHOLECYSTITIS.

M.I.Proudkov, .S.A Zaikov, A.A.Paderin, Sh.B.Bagmanyan

The Ural State Medical Institute

Ekaterinburg, Russia

For the last years laparoscopic cholecystectomy has become quite popular in treating patients with cholelithiasis. However in treatment of acute cholecystitis this kind of operation is being used quite seldom because of some technical problems. Whereas the first variant of laparoscopy ("open" laparoscopy by D.O. Ott (1901), according to which the examination of abdominal cavity is being performed through a small open wound has been practically paid no attention to.

### P043

### LAPAROSCOPIC CHOLECYSTECTOMY: EXPERIENCE WITH 527 PATIENTS

Boutzouvis S., Niotis M., Vlachos A., Ziakas M., Grigoraki E., Kostoglanis C.
First Department of Surgery, NIMTS Hospital, Athens, Greece.

Laparoscopic cholecystectomy has become the procedure of choise for surgical removal of the gallbladder and it is more popular than the traditional procedure.
Five houndred twenty seven laparoscopic cholecystectomies were done in our department between June 1992 and December 1994. In 16 patients (3 percent) the operation was converted to conventional open cholecystectomy. The most common reason was the inability to identify safely the cystic duct and the cystic artery (11 cases). Other reasons were: injury of the common bile duct (2 cases); injury of the junction between cystic and common bile duct (one case); diverticulum of the common bile duct (one case); cancer of the gallbladder (one case). The mean hospital stay for the patients was 1,1 days.
In conclusion, the results of laparoscopic cholecystectomy compare favorably with those of conventional cholecystectomy with respect to mortality, complications and length of hospital stay.

### P044

### FACTORS INFLUENCING THE DECISION TO CONVERT FROM LAPAROSCOPIC TO CONVENTIONAL CHOLECYSTECTOMY

F.E. Klee[1], B.R. Osswald[2], St. Wysocki[1]. Depts. of Surgery, [1]Salem Medical Center, [2]University of Heidelberg, Heidelberg, F.R.G.

We perform laparoscopic cholecystectomy since 1990 (n>1500). The conversion rate from endoscopical to conventional cholestystectomy remained constant. Our aim was to investigate different factors influencing the decision for the change of the surgical approach.

The decision whether minimal invasive or conventional cholecystectomy is to be performed takes account of age, individual risk factors, etc. However, besides technical difficulties and/or anatomical variations also intraoperative complications (bleeding, bile duct injury etc.) occured in some patients. Although it seems to be impossible to avoid every conversion of the surgical method, exact history and preoperative diagnostic examination may reduce the number of laparotomies.

In our patient group, we observed in tendency, that previously performed abdominal surgery represents the only factor indicating a higher risk of severe adhesions leading to technical problems during laparoscopic surgery. The constant conversion rate during the last four years possibly reflects the increase of „high risk“ patients within the laparoscopic treated patient group. In conclusion, laparoscopic cholecystectomy requires fundamental knowledge in conventional cholecystectomy.

### P045

### LAPAROSCOPIC CHOLECYSTECTOMY IN MAPOSITIONED GALL BLADDER

Palanivelu C. MS.MCH., Rajan P.S., Mahesh Kumar, *Department of Surgical Gastroenterology, Coimbatore Medical College Hospital, Coimbatore-641 018, INDIA.*

Though Laparoscopic Cholecystectomy has become the gold standard treatment for Gallstone disease, Laparoscopic management of malpositioned gallbladder is difficult and the technique needs to be improved for successful cholecystectomy.

Though June 1991 to October 1994, 1250 patients gallstone disease were treated by laparoscopic method. 11 patients had abnormally placed gallbladder. The Mal Position as follows: A.Situs inversus totalis-1, B.Left lobe - 3 C.Quadrate lobe-4 and D.RT Lobe liver-3. The problems: a,Difficult traction of liver b, cystic duct joining the Left Hepatic Duct in 6 patients. Trocars were positioned in different places for good exposure. In left lobe and quardrate lobe gallbladder extra port was made to lift the quadrate lobe and the working port was made in the left mid clavicular level. In situs inversus, trocars were placed as mirror image of the standard technique.

Retrograde cholecystectomy was performed in 5 patients where cystic duct joining the left hepatic duct deep in the hilum in four and liver plastered to the chest wall in one. . Modified subtotal cholecystectomy was performed in 4 patients by dividing the gallbladder at the infundibulum and the neck [illegible] cystic duct was covered by suturing the flap.

In all the 11 patients laparoscopic cholecystectomy was performed successfully. 1 patient had bile leak treated conservatively. Post operative recovery was similar to standard Laparoscopic Cholecystectomy.

Malpositioned gallbladder with stone disease can be effectively managed by Laparoscopic Cholecystectomy.

### P046

### DIFFICULT LAPAROSCOPIC CHOLECYSTECTOMY

S. Rizos, B. Kekis ,S. Skaltsas, M. Azami, B. Primikiris, G. Giannatos, D. Tsatzalos. G. Lakiotis.

Second Department of Surgery, 1st Hospital IKA, Athens, Greece
Department of Surgery, Agii Anargiri Hospital, Athens, Greece

1250 laparoscopic cholecystectomy were performed in our departments from Jan.1991 to Nov. 1994. The applicability was 85%. There were 13 (1.04%) major complications without mortality. 21 cases were converted to open cholecystectomy with a coversion rate of 1.7 %. The reasons for conversion were severe adhesions ( 9 ), CBD stones found by IOC ( 3 ), CBD injuries ( 2 ), Gall bladder cancer ( 4 ), and cholecystoduodenal fistula ( 3 ). Of the remaining 1229 laparoscopic cholecystectomy, approximately 15% were considered difficult. These included chronic contracted gall bladder with acute attack ( 68 ), gangrenous cholecystitis ( 31 ), extra-large stone without free lumen of gall bladder ( 35 ), porcelain gall bladder ( 21 ), embedded gall bladder ( 23 ), short cystic duct ( 19 ), stone impaction at the cystic duct ( 25 ), anatomical variation of cystic duct and/or cystic artery ( 24 ), severe adhesion from previous upper abdominal surgery ( 81 ), liver cirrhosis ( 19 ) and etc. The length of operation for laparoscopic cholecystectomy for acute cholecystitis was 40-90 min ( 60 min ), for gangrenous cholecystitis the laparoscopic approach took longer time 60-150 min ( 80 min). The length of stay for acute cholecystitis was 1-6 Days and 3-8 days for gangrenous cholecystitis. Conversion to open cholecystectomy is advised for difficult laparoscopic cholecystectomy when delineation of the Calot triangle can not be accomplished after 40 mins laparoscopic dissection.
So, we concluse that LC with improvements in laparoscopic instrumentation have become the standard treatment for patients with complicated gallstone disease.

### P047

### LAPAROSCOPIC CHOLECYSTECTOMY

M.Velegrakis, S. David, A. Saroukos, M. Savvidou, E. Gaganis, S. Kandylakis.
First Department of Surgery, Venizelion General Hospital, Iraklion Crete Greece.

Currently the laparoscopic cholecystectomy (LC) has been adopted by many surgical centres as the method of choice for the therapy of cholelithiasis.
Over the past three years LC has been undertaken on 221 chololethiasic patients, which was successful in 208 of those (179 females and 29 males, 17 to 78 years old, average age 47.58 years old). Criteria for selection of this method were the absence of obstructive jaundice in the past history of the patients and previous operations in upper abdomen. In thirteen patients (5.8%) the laparoscopic method was converted to open operation due to the thickness of the gallbladder wall and to the solid adhesions in Calot's triangle. Hasson's procedure was used in 78 patients because of previous sub-umbilical laparotomies, and laparoscopic cholangiography was performed in 9 cases. LC by the fundus was carried out in five cases due to difficulties in preparation of cystic duct and artery, which were ligated after the gallbladder was mobilized. Serious intraoperative complications were not observed and the mean operative time was 2 hours. Postoperative complications occurred in four patients (1.92%). One patient required prolonged exploration due to bile leak after a presumed diathermy injury of the CBD. Bile leak was observed in a second case caused by bad application of clips on cystic duct, DVT in a third case and sub-cutaneous emphysema in another one. In all the patients antibiotics were administered perioperatively, anti-thrombotic agents were also given and sub-hepatic drainage was established in all patients for 24 hours. In the rest of the patients, the post-operative recovery was uneventful, the average time of hospitalization was 2.6 days and the patients returned to their normal activities after one week.
In conclusion, laparoscopic cholecystectomy has now become a method of choice for the treatment of chololethiasis. This method, for its advantages, is embraced with confidence by the patients, because it rids them of their disease avoiding the trouble of open cholecystectomy.

### P048

### COMPLICATIONS OF LAPAROSCOPIC CHOLECYSTECTOMY

S. Rizos, S. Skaltsas, I. Paltoglou, G. Koliopoulos, D. Bisbikis, Maria Blitaki, B. Kekis. G. Lakiotis.

Second Department of Surgery, 1st Hospital IKA, Athens, Greece
Department of Surgery, Agii Anargiri Hospital, Athens, Greece

Between Jan. 1991 and Nov. 1994, 1250 laparoscopic cholecystectomies (LC) were attempted by a team of surgeons. The mean age was 47 years (range 18-91) and 890 ( 71.2% ) were female. The indications for the procedure were : chronic cholecystitis in 1090 cases ( 87.2 %), acute cholecystitis in 111 ( 8.8% ) and gallbladder polyps in 4( 0.3 %). The diagnosis was confirmed in all cases by ultrasound. Because of presumed pathology on ultrasound, suggestive for common bile duct stones or papillary stenosis 63 patients ( 5% ) had preoperative ERCP. In all patients with calculi ( n= 40 ) endo scopic stone removal was successfully performed with sphincterotomy. Morbidity was 1.6% after ERCP. The procedure needed conversion in 21 cases ( 1.7 %) . The reasons for conversion were severe adhesions ( 9 ), CBD stones found by IOC ( 3 ), CBD injuries ( 2 ), Gall bladder cancer ( 4 ), and cholecystoduodenal fistula ( 3 ). Other ntraoperative complications which did not require conversion included : bleeding 11, perforation of the gallbladder 223 , laceration of the cystic duct 3, stones left in the abdomen 45 etc. Morbidity rate varied between o to 7.2%. Mortality rate was 0%. Technical complications in 4 cases. Six patients required reoperation : dislodgement of cys tic duct clips 1, bleeding of liver bed 1, bleeding of trocar place 2, and bile leaks 2. Follow up showed 21 patients with retained stones. Minor complications arose in 3 cases.
LC shows an overhelming impact upon treatment of gallbladder diseases and a comparative morbidity with traditional operative and nonoperative methods.

### P049

### THE SOCIO-ECONOMIC ASPECTS IN LAPAROSCOPIC GALL STONES SURGERY IN OUR EXPERIENCE

B.Łazarkiewicz, J.Rudnicki, W.Kwarciński, W.Kielan, M.Ściebura

Second Department of Surgery, Medical Academy of Wrocław, Poland

The authors present 250 laparoscopic cholecystectomies as compared to a similar number of conventional cholecystectomies. The analysis comprised the length of stay in hospital, the time of convalescence and return to professional activity, the necessity of postoperative antibiotic administration, as well as the cost of the operation ( surgical tools and other equipment, sutures and dressing materials ), anaesthesia, postoperative therapy and accommodation. Also assessed were such subjective effects as the look of the scar, peri- and postoperative stress and the time of return to full vital activity.
It was proved that laparoscopic cholecyscectomies had substantial advantage over the convential ones, i.e., they facilitate shorter hospitalisation time, quicker return to work, much lower costs of medicamental therapy, reduced stress and better cosmetic effects.

### P050

### THE INCIDENTAL GALLBLADDER NEOPLASIAS SEEN DURING LAPAROSCOPIC CHOLECYSTECTOMY: IS IT A DILEMMA ?

**D. GÜZEY, F. ÇELEBİ, N. MEMMİ, M. AKAYDIN, C. ER, R. KAPLAN**

*Vakıf Gureba Hospital, II. Dept. of General Surgery, Istanbul, TÜRKİYE*

The incidental diagnosis of gallbladder neoplasias during cholecystectomy is said to be seen in one percent of symptomatic cholelithiasis. In 15-30 % of these cases diagnosis can only be made during histopathological study postoperatively.

The incidence of such neoplasias seems to have decreased in the published series, following the widespread use of the "gold-standart" laparoscopic cholecsytectomies. The question is whether there is a true decrease in the rate or some neoplasias are being overlooked.

This paper is a prospective study concerning 100 endoscopic surgical interventions. We have seen two cases with incidental neoplasias without any symptoms prior to operation. In one of these cases the pathologist had to confirm the diagnosis only after histopathologic study, where both cases were classified as pT2.

Patients with Stage 2 (pT2) tumor have a good survival rate following simple cholecystectomy, unlike patients with Stage 3 (pT3 or N1) tumor. R0 resection is the preferred operation in cases with Stage 3 (pT3).

We conclude that in any case should there be a suspect of an intramural tumor, shift to open surgery will certainly be beneficial for the patient in diagnosis and treatment.

### P051

### A RANDOMIZED STUDY OF RE-OPERATIONS AFTER CONVENTIONAL CHOLECYSTECTOMY (CC) VERSUS LAPAROSCOPIC CHOLECYSTECTOMY (LC)

Ja.Bereznitsky, I.Bondarenko, B.Shevchenko, S.Shiriaev, A.Semashko
Digestive Organs Surgical Department, Institute of Gastroenterology, Dniepropetrovsk, Ukraine

Laparoscopic techniques have radically changed the procedure of gallbladder and gallstones removing. The aim of our study was to find out the essence of complications, which demanded re-operations after CC and LC.

Patients and Methods: For the last 15 years 4875 patients having been operated on by a routine method, for the period of February-November 1994 - 186 patients have undergone LC.

Results: After CC 85(1,74%) patients needed Relaparotomy due to 4 types of serious complications: 1.peritonitis and small bowel obstruction in 35(0,72%) cases; 2.intraabdominal abscesses-in 31(0,64%); 3.post-operative bleeding-in 12(0,24%); CBD fistula, CBD obstruction and jaundice, pancreonecrosis-in 7(0,14%). After LC 5(2,69%) patients were re-operated: one-openly because of partial CBD injury, four -laparoscopically to drain subphrenic abscesses. Re-operations mortality rate was 29,4% (25/85) at CC group and there were no fatalities among LC patients.

Conclusion: The frequency of re-operation incidents after LC did not exceed re-operation incidents after CC. They were directly connected with operative locus in case of LC, while generalized complications prevailed after CC. LC complications course was more favourable.

### P052

### PORTOSYSTEMIC ENCEPHALOPATHY AFTER MESOCAVAL SHUNTING AND SCLEROTHERAPY

B Isaksson, E. Tideman, F. Bengtsson, I. Rosén, T. Jonung, B. Jeppsson
Department of Surgery, Neurology, Clinical Pharmacology and Neurophysiology, Lund University, Sweden

The influence of shunting or sclerotherapy on the development of hepatic encephalopathy has not been clearly defined. Results from several studies vary considerably. The problem of post shunt encephalopathy has been revived in the 90's with the introduction of TIPSS which has an incidence of encephalopathy between 15 and 25%. We report the incidence of hepatic encephalopathy in a prospective study comparing mesocaval interposition shunt and endoscopic sclerotherapy in the prevention of rebleeding from oesophageal varices.

Material and methods: 24 patients were randomised to shunt and 21 to sclerotherapy. All Child's classes were represented. Encephalopathy was evaluated by EEG with spectral analysis and a battery of psychometric tests.

Results: 9 patients exhibited mild to moderate encephalopathy preoperatively. All these patients remained encephalopatic, two of them detoriated post shunt. In the sclerotherapy group 13 patients were encephalopatic. In both groups the patients were encephalopatic before start of study and remained so through out follow up. The psychometric tests showed that patients in the shunt group performed significantly poor in tests measuring verbal ability, visual performance and logic inductive capacity and intellectual capacity.

Conclusion: We could find that the shunt group had a significantly poorer performance in three psychometric tests during follow up but this did not influence the total score of all psychometric tests in that it did not create any significant difference between the shunt group and the sclerotherapy group.

### P053

USE OF A BIOFRAGMENTABLE RING IN THE MENAGEMENT OF BLEEDING ESOPHAGEAL VARICES.

F. OLIVEIRA MARTINS; J.M. SCHIAPPA; J. SILVA FERNANDES
J. GIRÃO
HOSPITAL SANTO ANTONIO DOS CAPUCHOS-LISBOA - PORTUGAL

INTRODUCTION_ Portal hypertention and its Bleending complications still are a great challange, specially if sclerotherapy fails.
OBJECTIVE - Our objective was an easy and effective surgical approach for menagement of rebleeding esophagel varices after sclerosis.
MATERIAL AND METHODS _ We've performed in 4 cases esophageal transection with a Biofragmentable Ring associated to splenectomy and gastric disconection.

CONCLUSION _ We've achieved control of bleeding situation, with no complications directed related to the procedure. We assess the Biofragmentable Ring with daily X-Ray and contrast esofagogastric X - Ray 10 days after Surgery. Endoscopy was performed two weeks after surgery, we haven't found any leakage or significative stenosis, and we've realise the disruption of the ring by 12 day after surgery. We think in emergent situations it is an easy and efective procedure to performe but only to control bleeding esophageal varices.

### P054

CONSERVATIVE MANAGEMENT OF BLEEDING ESOPHAGEAL VARICES BY OCTREOTIDE AND SOMATOSTATINE

P.Kagmakis, P.Potamousi, Th.Kalogirou, M.Exarchakos, A.Priftis, A.Akizidou, M.Hadjiyannakis
A'DPT of Internal Medicine, Asklipieon General Hospital, Voula, Greece

The bleeding esophageal varices, is a medical emergency. The conservative management, has changed since the introduction of Somatostatin and Octreotide, a new synthetic analogue! This study was performed to assess the possible differences between Octreotide and Somatostatin in bleeding esophageal varices. Thirty four patients with bleeding from esophageal varices, were admitted to our Hospital during the last 3 years. All patients, inittially, were treated by placement of a Sengstaken - Blakemore tube (SBT) for 48 h. At the same time, they randomized to take Somatostatine or Octreotide. Group A (17 patients) was treated with continuous i.v infusion of Somatostatin and Group B (17 patients) with intermittent doses of Octreotide. Somatostatine was given as a bolus 50 μg and then, as a continuous i.v drip at a dose of 250 μg/h for 5 days. Octreotide, was given as s.c. injections 0,1 mg/8 h for 5 days. Activelly bleeding was controlled in 16 (94%) patients in group A and 15 (88%) in group B. There was 1 rebleeding in both groups and 1 patient of group B, needed urgent endoscopic sclerophera PY. Blood transfused (units) for each group was 4,1±2,1 in group A and 5,2±4,2 in group B. We have no death and no side effects from the therapy. We conclude, that Somatostatine and Octreotide are both effective in controlling bleeding from esophageal varices, but the results are better with the continuous infusion of Somatostatin.

### P055

**DISTAL SPLENORENAL SHUNT. LONG TERM RESULTS**

C.Margarit, JL.Lázaro, J.Balsells, R.Charco, E.Murio, I.Bilbao, E.Gifre, C.Ruiz and J. Bonnin.
Hospital General Universitario Vall d'Hebron. Barcelona, Spain.

MATERIAL AND METHODS
From January 1987 to February 1994, 75 patients were operated due to bleeding esophageal varices. Forty-one patients underwent Warren operation with a mean age of 50.6+-19.4 years. Elective surgery was performed in 28 cases (68.3%) and urgent in 13 (31.7%). Ethiology of portal hypertension was: liver cirrhosis in 36 patients, liver fibrosis in 1, portal thrombosis in 1, and rebleeding in patients with previous portal hypertension surgery in 3. Child A was in 29 patients (70.7%), B in 8 (19.5%) and C in 4 (9.8%).
RESULTS
Operative mortality was 7.3% (3 patients) and early rebleeding occurred in four patients (9.7%) but shunt occlusion was found in only one.
Follow-up: thirty-eight patients were discharged from the hospital. Mean follow-up was 32.1±23.4 months (range:2.2 to 77.2). Six patients (15.7%) died in the follow-up (4 from liver failure, 1 from post-transplant sepsis and 1 from hemorrhage post-liver biopsy).
Three patients presented encephalopathy requiring medical treatment and three developed hepatoma. Two underwent liver transplant.
Recurrent hemorrhage occurred in 3 cases (7.8%) and shunt occlusion was found in two by ultrasonography. Several hemorrhage episodes were presented in other patient despite open shunt and no evidence of bleeding lesion was found by colonoscopy and upper endoscopy.
Actuarial survival were: 82.9%, 64.4% and 46% at 3, 4 and 6 years, respectively.
CONCLUSION
Warren operation is a safe procedure with a low rebleeding and encephalopathy rate, and low mortality. We recommend this shunt as elective operation in patients with bleeding esophageal varices.

### P056

**ESOPHAGOGASTRIC DEVASCULARIZATION, SPLENECTOMY AND POSTOPERATIVE ENDOSCOPIC SCLEROTHERAPY FOR THE TREATMENT OF SCHISTOSOMAL PORTAL HYPERTENSION**

V. Pugliese, P. Herman, M.C.C. Machado, H.W. Pinotti
Department of Gastroenterology, University of São Paulo, Brazil

Bleeding esophageal varices is the leading cause of death in patients with schistosomal portal hypertension. The objective of this study was evaluate the effectiveness of a non-shunt operation associated with endoscopic sclerotherapy for the elective treatment of schistosomal portal hypertension.

From August 1988 to August 1993, 84 patients with hepatosplenic schistosomiasis (confirmed by liver biopsy) and history of upper gastrointestinal bleeding were prospectively studied. The surgical procedure was always performed by midline laparotomy and consisted of splenectomy, devascularization of the abdominal esophagus and proximal part of the stomach and esophageal fundoplication. The sclerotherapy was performed by intravariceal injections of ethanolamine, the first session done two months after the operation and continued at three-monthly intervals till obliteration of varices was achieved.

No mortality was observed. Early postoperative complications registred were: portal vein thrombosis (53,2%), ascites (39.3%), acute pancreatitis (3.6%), pancreatic fistula (3.6%), respiratory complications (2.4%) and esophageal fistula (1.2%). 13 patients were lost to follow-up, the 71 remainig patients had a mean follow-up of 30 months. The endoscopic aspect of the esophageal varices was significant improved after treatment as assessed by Paquet's classification (Wilcoxon $p<0.05$). The total rebleeding rate was 7.0% and the variceal rebleeding rate 4.3%, these episodes were successfuly treated by conservative measures.

The authors conclude that esophagogastric devascularization associated with endoscopic sclerotherapy represent a good alternative for the elective treatment of schistosomal portal hypertension.

### P057

### COMBINED SURGICAL PROCEDURE FOR TREATMENT OF PORTAL HYPERTENSION

J.Piecuch, M.Pardela, A.Kozłowski, K.Witkowski, W.Orkisz, J.Puzio, M.Wyleżoł, A.Urbaniak
Dept. of General and Vascular Surgery, Silesian Medical School, Zabrze, Poland

The most dramatic complication of portal hypertension is oesophageal varices haemorrhagia. Surgical treatment tends to control this in spite of hypertension decrease in portal system or inflow portal blood reduction to oesophageal varices. In our surgical department in 1986-92 36 patients with portal hypertension caused by prae- or intrahepatic block were treated by surgical operation. In anamnesis, before operations patients reported one or more cases of haemorrhagia from oesophageal varices. In Child's scale three of them were in C-level, 25 in B and eight in A. Till 1988 in treatment of portal hypertension vascular operations were performed and operations called "non shunt", from which assent oesophageal transsection with following anastomosis with front wall of stomach bottom with devascularization underdiaphragmatic part of oesophageus, bottom and trunk of ventricul, with pyloroplasty and splenectomy by abdominal entrance. In the begining, transsection and oesophago-gastro anastomosis was made by a traditional method. For four years transsection (with ILS appliance) complemented by spleno-renal anastomosis by Linton method have been made both of kinds of operations simultaneously. Combination of both operation techniques leads to the reduction of portal hypertension and also prevents rebleending from oesophageal varices. In 14 cases simultaneous operations were performed. From this group 3 patients died before the 14th day after operation. In 11 cases under observation from 6 to 52 months no broaden of portal or splenic vein were noted in ultrasound examination. In control gastrofiberoscopy in two cases there was ascertaining presence of I oesophageal varices and in nine - variceal changes have completly retracted. In postoperative control biochemistry examinations (albumins level, GOT, GPT, GGTP, bilirubine, time and content of protrombin) show moderate level of hepatic function handicap.

### P058

### HEMODYNAMIC CONSEQUENCES OF SPLENIC ARTERY OCCLUSION WITH ENDOSCOPIC SCLEROTHERAPY IN PATIENTS WITH ADVANCED LIVER CIRRHOSIS

K. Boulanov, N. Kalita, L. Nikishin, A.Bouriy
Department of Liver Surgery and Portal Hypertension, Institute of Clinical and Experimental Surgery, Kiev, Ukraine

Alterations in splanchnic hemodynamics play key role in the development of portal hypertension and it's complications in liver cirrhosis. The aim of the study was to evaluate results of serial determination of hemodynamics in 30 cirrhotic patients (Child class B-18,. C-12), who undergone splenic artery occlusion (SAO) and subsequent endoscopic sclerotherapy (ES). ES began 14 days after SAO and repeated every 6 month. Measurements were performed before and after SAO and ES using duplex Doppler flowmeter system (ml/min) and included the following parameters: splenic arterial flow (SAF), splenic venous flow (SVF), portal venous flow(PVF) and hepatic arterial flow (HAF). P values < 0.05 were considered *significant* (*).

Preoperative indices were the following: SAF-436.1, SVF-776.6, PVF-846.1, HAF-99.5. Postoperative studies (10-12 days after SAO) revealed reduction of SAF-213.1*, SVF-522.0*, and PVF-541.0*, whereas HAF increased to 150.8*. One week after ES PVF increased to 696.2*. Results of remote investigations (12-18 months) were similar to postoperative data, but still showed evidence of significant differences with preoperative values: SAF-189.8*, SVF-499.2*, PVF-706.0, HAF-152.7*. Increasing of PVF and HAF resulted in considerable enhancing of Child-Pugh score in 15 patients.

Thus, SAO accompanied by ES causes favorable changes of portal hemodynamics in patients with liver cirrhosis and poor hepatic reserve.

### P059

### LONG-TERM FIBREOPTIC INJECTION SCLEROTHERAPY FOR BLEEDING OESOPHAGEAL VARICES: A PROSPECTIVE EVALUATION IN 204 PATIENTS

JEJ KRIGE, PC Bornman, J Terblanche. Department of Surgery and MRC Liver Research Centre, University of Cape Town and Groote Schuur Hospital, Cape Town, South Africa.

The efficacy of long-term injection sclerotherapy (IST) in eradicating oesophageal varices after endoscopically proven variceal bleeding was assessed prospectively in 204 patients between 1984 and 1989. Data were analyzed in December 1994 to allow a minimum 60 month follow-up. The 204 patients (127 men, 77 women; mean age 50.3, range 16-82 years) underwent 1022 emergency and elective injection treatments with 5% ethanolamine oleate using a combined intra and paravariceal technique during 1860 endoscopy sessions during the study period. The majority (167;82%) had cirrhosis, mainly due to alcohol (131;64%). The Pugh-Child's risk grades were A:26, B:94, C:84. Seventy-five (37%) of the 204 patients had a total of 130 bleeding episodes after the first hospital admission before eradication of varices (0.03 bleeding episodes per patient month of follow-up). Rebleeding was markedly reduced after eradication of varices. In the 100 (87%) of 114 patients who survived >3 months, varices were eradicated after a mean of 5 injections and remained eradicated in 47 [mean follow-up: 71.5 months; range:5-120 months]. Varices recurred in 53 patients and rebled in 18 of whom only 8 rebled from oesophageal varices. Cumulative survival by life table analysis was 55%, 42%, and 32% at 1,3 and 5 years. 113 patients (55.4%) died during follow-up. Liver failure was the most common cause of death. Of the 236 complications which occurred in 139 (68.1%) patients, mucosal slough (137 patients) was the most common. A localised injection-site leak occurred in 9 patients and oesophageal stenosis developed in 23 patients of whom 14 required dilatation (mean:4; range: 1-7 dilatations). Free oesophageal perforation occurred in 5 patients, 4 of whom died. Repeated fibreoptic IST eradicates oesophageal varices in the majority of patients with a reduction in rebleeding. Complications related to IST were mostly of a minor nature but became cumulative with time.

### P060

### SPLENIC ARTERY EMBOLIZATION (SAE) VERSUS SPLENECTOMY FOR HYPERSPLENISM IN PATIENTS WITH LIVER CIRRHOSIS

A.M.Granov, P.G.Tarazov, A.A.Polykarpov
Department of Surgery and Division of Angio/Interventional, St. Petersburg Research Institute of Roentgenology and Radiation Therapy, Russia

We compared safety and effectiveness of SAE and splenectomy for correction of cirrhotic hypersplenism. We treated 52 pts with Child-Pugh class A (35) or B (17) portal cirrhosis. Group I of 33 pts underwent proximal SAE with coils. Splenectomy (mostly with omentohepatopexy) was performed in 19 pts of Group II. Both groups were fully comparable. The mortality was 6% in Group I (variceal bleeding and sepsis each 1) and 5% in Group II(sepsis - 1). The complication rates were 24% (splenic abscess - 2, left-sided pneumonia - 3, increase of ascites - 3) vs 10% ( subphrenic abscess and operative bleeding each 1 ). In Group I, the platelet count (PC) increased significantly 1 mo later but then returned to the pre-SAE level. In Group II, signigicant improvement of PC during 3yr period of follow-up. The 5yr survival rates in Groups I and II were 35% and 80% (.05 P .1). These results showed that surgical splenectomy is more effective than proximal SAE in correction of hypersplenism in patients with nonadvanced hepatic cirrhosis. Moreover, splenectomy has at least equal or even less morbidity and mortality.

### P061

EMBOLIZATION-SCLEROTHERAPY (ES) AND FERROMAGNETIC-ASSISTED HYPERTHERMIA (FAH) IN CAVERNOUS HEMANGIOMA OF THE LIVER (CHL)

A.M.Granov, V.N.Polysalov, K.V.Prozorovskij, P.G.Tarazov
Department of Surgery, Department of Radiology, Division of Angio/Interventional, St.Petersburg Research Institute of Roentgenology and Radiation Therapy, Russia

This study was performed to assess effects of ES and FAH as new treatments for unresectable CHL in 22 patients with highly symptomatic disease. ES was performed in 12 patients with 25 tumors of 4 to 15 (mean 9) cm in diameter. Transcatheter selective arterial embolization was made with Ivalon/Gelfoam followed 2 weeks later by ultrasound-guided local sclerotherapy with 50% to 99% ethanol and/or thrombin. FAH was performed in 10 patients with 22 CHL. Magnetic particles 1 μm to 10 μm of $Ba_2Fe_2O_6$ (2 to 40 g, mean 12 g) were injected directly into the tumor under local external magnetic field. Local hyperthermia using ultra-high frequency machine was made 1 to 3 weeks later. Aseptic necrosis and vascular thrombosis was seen in 1 to 3 months after each treatment with following fibrosis 3 to 24 months later. Clinical improvement in all but 2 cases and 10% to 50% tumor decrease was seen during 5 to 40 month follow-up. It may be concluded that both treatments ES anf FAH are equally effective and seem to be useful in the management of selected patients with highly symptomatic inoperable CHL.

### P062

SURGICAL TREATMENT OF CAVERNOUS HEMANGIOMAS OF THE LIVER

Ö. Alabaz, H. Demiryürek, D. Ağdemir, A. Alpaslan, Department of Surgery, University of Çukurova, Adana, TÜRKİYE.

Cavernous hemangiomas are the most common benign tumors of the liver. Giant cavernous hemangiomas defined as these larger than 4cm in diameter, can reach enormous. Between 1979 and september 1994, cavernous hemangiomas of the liver were surgically treated in 18 women and 3 men over a 16-year period. Tumors were visualized by ultrasonography in all cases and by computed tomography in 9. The tumors were solitary in 18 cases and multiple in 3. Locations were the right lobe in 12 cases, the left one in 7 and both lobes in 2. The size of the tumors ranged from 4.0 to 25cm in diameter. Enucleation of tumors was carried out in 14 cases, anatomical lobectomy in 3 cases, an atypical liver resection in 3 cases and segmentectomy in 1 case.
Median operative blood loss was 700ml(range 300 to 4000ml). There were no surgical deaths. Three cases had postoperative complications. One patient had a pneumonia on the right side, one had subdiaphragmatic abscess, and the other had wound infection.
We reported the result of our experiences in removal of liver hemangiomas of various sizes.

### P063

VIRAL ETIOLOGY OF CIRRHOTIC PORTAL HYPERTENSION

Paula Szanto, Olimpia Chira, A.Neamţu

3rd Medical Clinic, Cluj, Romania

Viral hepatitis B,C and D markers were determined by ELISA method in 97 patients with liver cirrhosis(57 males and 4o females)dispensarised over a period of one year.The diagnosis was based on clinical,biochemical,histological and endoscopical criteria.Ninety-three of 97 patients with portal hypertension had viral hepatitis markers,two hepatic cirrhosis were alcoholic and two were primary biliary cirrhosis.Sixty-nine of the viral cirrhosis were with hepatitis B virus(HBV) and 17 of these were with D(HDV) virus(24.63%).Hepatitis C virus(HCV)was present in 59 patients(63.44%).In 29 patients,double HBV andHCV infection was present,and 8 patients had triple HBV,HCV and HDV infection.Among the 93 viral liver cirrhosis(VLC),81 were decompensated,61 having ascites.Alcohol consumption was present in 59 from 93 VLC(63.44%)and a significant association with the consequences of the portal hypertension(hepatic encephalopathy in 4o cases,variceal bleeding in 14 cases)was found in this group.
Viral etiology dominates the portal hypertension in liver cirrhosis and represents 95.97% in Transylvania,the nord-western area of Romania.The double or triple infection and especially the alcohol consumption represent a high risk of portal hypertension complications.

### P064

DUPLEX DOPPLER ULTRASOUND SIGNS OF PORTAL HYPERTENSION: RELATIVE DIAGNOSTIC VALUE OF EXAMINATION OF THE PARAUMBILICAL VEIN, PORTAL VEIN AND SPLEEN

C. Schulze
Department of Surgery, University of Brussels, Belgium

The sonographic parameters in portal hypertension (PHT) were examined in a consecutive population of 100 patients who had PHT, diagnosed using specific endoscopic, sonographic and Doppler signs. A patent or enlarged paraumbilical vein was found in 85% of the patients overall and 82% of the patients with varices, indicating a relatively high sensitivity. A portal vein of diameter ≥ 13 mm was found in only 42% and ≥ 15 mm in only 18% of the patients. A thrombosed portal vein and reversed portal vein flow were present in 4% and 6% of the patients, respectively. These signs have only been reported in the context of PHT and are felt to be specific for PHT, but both have very low sensitivity. Portal vein velocities were highly variable, suggesting that this is not a useful predictor of PHT. Splenomegaly was found in only 54% of the patients, demonstrating its poor sensitivity as a sign of PHT. Varices were found in 76% of the patients overall and in 100% of the patients with a patent or enlarged paraumbilical vein combined with ascites.
We conclude that the presence of a patent or enlarged paraumbilical vein is a practical, useful and sensitive ultrasound sign to look for in the diagnosis of PHT.

### P065

### Traumatic Lesion of Liver Parenchyma Following Blunt Abdominal Trauma, Complication of Primary Packing and Management of Haemorrharge With the Help of Interventional Radiology: A Case Report

K.T.E. Beckurts, H. Bartels, J. Adolf, K. Lehner, G. Oedekhoven and J.R. Siewert

We report on the case of a 16 year old male patient who experienced a winter sports accident (sledge collision) with blunt abdominal trauma. Primary treatment of the intraabdominal haemorrharge was by emergency laparotomy; control of bleeding was attempted by application of intraabdominal perihepatic swab packing. 16 hours after the trauma, the patient was refered to our institution for definitive treatment. He presented with severe hypovolaemic shock, hypothermia and coagulopathy. After adequate fluid replacement, correction of coagulopathy and stabilisation of vital parameters, further radiologic evaluation (CT-scan, angiography) revealed a large intrahepatic haematoma with ongoing intrahepatic arterial bleeding from a branch of the right hepatic artery; control of bleeding was achieved by repeated selective coiling of the ruptured vessel. After a stable interval of 12 hours, relaparotomy was performed with complete removal of the package, lavage and definite abdominal closure. The patient remained haemodynamically stable thereafter, but developed progressive swelling of both lower limbs; suspected compartment syndrome was confirmed by pressure measurements, and bilateral complete fasciotomy was immediately performed. The ethiology of the compartment syndrome was most propably a severely compromised venous outflow during the abdominal packing period, possibly intensified by prolonged shock and hypothermia. Repeated operative debridement with removal of avital muscle was necessary. The patient required 13 days of ICU treatment and treatment was continued in the unit of reconstructive traumatology. The further clinical course was largely uneventfull; a small bile fistula could be diagnosed by endoscopic retrograde cholangiography but resolved spontaneously. The patient recovered completely from his intraperitoneal injury but has residual handicaps in his lower limb function.
In conclusion, interventional angiography can contribute to the succesfull management of higher grade traumatic liver injury. The technique of intraperitoneal perihepatic packing bears definite risks, and the complications of this treatment can dominate the long term course after liver trauma. The demonstrated case emphasises the neccesity of proper packing technique with preservation of inferior caval flow.

### P066

### SELECTIVE HEPATIC ARTERY EMBOLIZATION IS THE TREATMENT OF CHOICE FOR HEPATIC HAEMOBILIA

JEJ Krige, SJ Beningfield*, PC Bornman J Terblanche.
Departments of Surgery, Radiology* and MRC Liver Research Centre, University of Cape Town and Groote Schuur Hospital, Cape Town, South Africa.

Fourteen patients (10 men, 4 women, mean age 36.6 years, range 18-36 years) with major haemobilia originating in either the liver (13) or gallbladder (1) were treated between 1977 and 1986. Bleeding was due to penetrating (4) or blunt (1) liver trauma, iatrogenic (3) [percutaneous transhepatic endoprosthetic stent placement (2) liver biopsy (1)], arteriovenous malformation (2) right hepatic artery aneurysm (2), pyogenic liver abscess (1) and chronic cholecystitis with gallstones (1). All patients had melaena, 5 had haematemesis and RUQ pain was present in 8 patients. Only 2 patients were jaundiced. Bleeding from the ampulla was identified in 2 patients during ERCP. Endoscopy identified fresh blood in the second part of the duodenum in 7 of 10 occasions. A liver lesion was identified in 6 of 10 patients who underwent either CT scanning or liver ultrasound. Selective hepatic angiography demonstrated an intrahepatic bleeding source in 13 patients. An arteriobiliary fistula in the gallbladder in 1 patient was not identified by angiography. Selective hepatic arterial embolization using either gelfoam pledgets or Gianturco coils controlled bleeding in 10 of 12 patients. Embolization failed in 2 patients (1 with segmental liver necrosis required a right hepatic lobectomy and a second patient underwent surgery and ligation of the left hepatic artery). Bleeding from the gallbladder in 1 patient was treated by cholecystectomy. Selective hepatic artery embolization was not attempted in 1 patient who underwent a left hepatic lobectomy. Selective hepatic artery embolization was successful in 10 of 12 patients (83%) of whom 1 patient developed subsequent complications. Selective hepatic artery embolization provides definitive control of liver bleeding with a low incidence of complications and should be considered the primary treatment of choice for intrahepatic haemobilia.

### P067

### A LARGE TRAUMATIC HEPATIC HAEMATOMA
### *POST-TRAUMATIC EVALUATION*

Stratis J.,Petridis A.,Kakoutis E., Chatzichristou A., Rafael S., Pilavaki M.,Makrantonakis N.

*1st SURGICAL CLINIC G.R. HOSPITAL "G. PAPANIKOLAOU" THESSALONIKI, GREECE.*

Despite it's protection by the lower rib cage, the liver is frequently injured by blunt mechanisms because of its large size and relative inelasticity.

We present the results of 6 months follow-up of a large central post-traumatic hepatic haematoma. This developed as a result of blunt abdominal trauma and was diagnosed with C.T scan. Monitoring was based on Ultrasonography and Computerized Tomography. The significant change in the echographic pattern and the change of size of the haematoma in the C.T scans indicated its organization.

The purpose of this study was : 1) To show the U.S and C.T findings in the hepatic haematoma in relation to the time after trauma. 2) To suggest that in haemodynamically stable patients with intrahepatic haematoma, where the diagnosis can radiologically ascertained, conservative treatment is a safe and reasonable option.

### P068

### THREE-HOUR OCCLUSION OF THE HEPATODUODENAL LIGAMENT FOR LIVER TRAUMA - CASE REPORT

S. Repše, E. Gadžijev, M. Omejc, R. Juvan
Department of Gastroenterologic Surgery
University Medical Centre Ljubljana, Slovenia

A case of liver trauma due to traffic accident with three-hour lasting vascular occlusion of the hepatoduodenal ligament and additional 35 minutes of normothermic liver ischaemia is presented.
At the first step management of the 23-year old patient in a regional hospital only inadequate tamponade of the liver injury and occlusion of the Rhumle's tourniquet had been performed. Transportation to our department had taken unexpectedly long time (2,5 hours) and additional vascular occlusion (35 minutes) was needed to perform liver resection (V.,VI.,VII. and VIII. segment).
Long duration of vascular occlusion was caused by exceptional circumstances at that time in our country. Data of managements, events and laboratory findings are presented. There were no major complications and the patient was discharged the day $20^{th}$ after the operation.
Two years after the accident and the right hepatectomy the patient is well and healthy, weighting 95 kilograms and working as a waiter.

### P069

NON-OPERATIVE TREATMENT OF HEPATIC INJURIES IN CHILDREN

M. Soutis, G. Christopoulos-Geroulanos, G. Kapouleas, P. Theodorou, D. Keramidas
2nd Dept of Pediatric Surgery, "Aghia Sophia" Children's Hospital, Athens, Greece

Hepatic injury occurs as frequently as does splenic injury in children and it is caused mainly by vehicle accidents and falls. Selective non-operative management is based on the experience that about one half of patients operated on for hepatic trauma require only drainage of a non-bleeding laceration. The aim of this study is to assess the efficiency and the reliability of the conservative treatment in patients with liver injury. Twelve patients with blunt liver trauma were treated in our Department during a nine year period. Ten of them were treated successfully without operation, but two children required laparotomy on admission because of hypovolemic shock due to intraabdominal hemorrhage. Ten patients were diagnosed on admission by ultrasonography or CT scanning and they were submitted to the concervative management with excellent results. Our protocol for this treatment is as follows: bed rest for about ten days, close monitoring of the vital signs, serial hematocrit and hemoglobin evaluations and blood transfusions up to 50% of blood volume as needed to maintain hemodynamic stability. At the 10th day the U/S or CT scanning was repeated in order to evaluate the course of the hepatic trauma. All patients who were treated non-operatively had an uneventful course, as well as one of the two operated patients who had additional renal damage. The other patient who was operated on for multiple deep hepatic lacerations needed reoperation for a hepatic abscess. Based on our experience we believe that non-operative management of hepatic injuries is the treatment of choice in precisely selected patients submitted to close monitoring; it is effective and safe method without complications following laparotomy.

### P070

RIGHT HEPATECTOMY (RH) IN EDERLY PATIENTS

D. Sommacale, V. Moutardier, L. Dugué, A. Sauvanet, R. Noun, J. Belghiti
Department of Digestive Surgery, Hôpital Beaujon, F - 92110 Clichy.

In order to evaluate the tolerance and postoperative course of major hepatectomy in ederly patients we retrospectively studied an homogenous subgoup of 16 patients aged > 70 years who underwent a RH (segment V, VI, VII and VIII).
Patients and Methods: From January 1990 to July 1994, 40 patients (26 men and 15 women) without cirrhosis and ASA I or ASA II underwent a RH and were divided in two groups according to age : age < 65 years, (13 men, 11 women) mean age : 46.1±12 years (range, 22-65) and age > 70 years, (12 men, 4 women) mean age : 72.7±3 years (range, 70-80).
The two groups age < 65 years (n=24) vs age > 70 years (n=16) were comparable to preoperative PT level (86±13 vs 89±12%), preoperative Factor V level (94±10 vs 102±13%), weight of resected specimen (1213±1348 vs 1080±1230 gms), type of vascular occlusion (2 hepatic vascular exclusion vs one) and duration of vascular occlusion (31±12 vs 36±8 min).
Results: Number of transfusions (1.5±2 vs 2.3±2.4 Units) and duration of the resection (318±110 vs 342±118 min) were comparable in the two groups. The ASAT test level at day 1 (357±233 vs 358±218IU/L) and at day 7 (44±38 vs 57±25) were not significantly different. PT level at day 1 (50±10 vs 43.3±14%) and at day 7 (71±17 vs 68±20%) were lower in the group age > 70 years but this difference was not statistically significant. Factor V level at day 1 (57±18 vs 43±20%) was significantly lower in the group age > 70 years (p<0.05) than Factor V level at day 7 (74±26 vs 85±45%) was comparable in the two groups. Comparison between bilirubin level at day 1 (43±22 vs 92±75µmol/L) and at day 7 (24±22 vs 47±50) was significantly higher in the group age > 70 years (p<0.01 and p<0.05 respectively).
On patient died in each group (4% vs 6%) (NS). Postoperative course was uneventful in 15/24 (62.5%) vs 11/16 (68.7%) (NS). The hospital stay was identical in the two groups (16±6 vs 16.5±2).
In conclusion, this work shows that in selected patients, the clinical tolerance of RH seems not to be according to age despite a more important impairing and a longer recovery of the liver function in the group age > 70 years.

### P071

SURGICAL THERAPY OF UNILOBAR CAROLI'S DISEASE

U.Jost, P.Lamesch, H.Witzigmann, K.Kohlhaw, F.Geißler, J.Hauss
Second department of Surgery, University Leipzig, Germany

Case report: Tumors in the liver are nowadays by means of radiology diagnostics and extensive lab-diagnostics early to record and thus one differential therapy accessible. We report on a 65 year-old woman who because of pains in the right upper abdomen and frequent vomiting the family doctor located. Clinically neither a weight loss nor a sudden drop in productivity was found. By ultrasound a tumor area in the right liver lobe could be localized. A computer tomography [CT] secured a multiple cystic 11x9 cm large, repeatedly subdivided area with sharp contour and a pseudo capsular, primarily compatible with a Echinococcosis. Since the echinococcus serology test remained however repeatedly negative, we supposed a Caroli-Syndrom. In diagnostic calculations an adenoma or haemangioma could be excluded by CT. No reference was found in laboratory tests for malignancy (CEA, AFP, CA19-9,CA72-4). Signs for a liver cirrhosis did not consist. The hepatitis test was completely negative. In situ then a large more roughly, cystic tumor was found in that right liver lobe, which could become through a resection by hemihepatectomia. Histology shows then a 10x8x4 cm large whitish, cystic tumor with granulo-nodular inflammation with central, purulent abscess and local cystic enlargement of the intrahepatic biliary tract with more chronically, recurrent cholangitis. No support for malignancy is seen. The woman was hospitalised for 19 days and is now outpatient for 2 month. Conclusions: The modern radiology diagnostic could be helpful in discussion of different liver diseases. Due to an incidence of cholangiocarcinoma by approx. 11% at unilobar Caroli's disease is to be striven for a curative resection always.

### P072

OUR EXPERIENCE WITH A FIBRIN SEALANT (TISSUCOL) AND FIBRIN-ADHESIVE COATED COLLAGEN FLEECE (TACHOCOMB) IN LIVER SURGERY

A.Severtsev, T.Tchilina, V.Bashilov
Dpt. of Surgery, Medical Centre for Russian Government (MCRG); Hosp.N51, Moscow, Russia

There are many pharmaceutical products, which can control the oozing from hepatic surface during liver resection. The aim of this study was to compare a fibrin sealant (Tissucol/TS/, Immuno AG, Vienna, Austria) and fibrin-adhesive coated collagen fleece (TachoComb/TC/, Hafslund Nycomed Pharma, Linz, Austria).
During a 1-year period, 14 hepatic resections were performed in 13 patients. TS or TC were applied after hepatic parenchymal division. Hemostasis of the blood vessels/bile ducts had been achieved using ligatures, plasma flowes, etc. TC was used for haemostasis in 8 patients and TS was used in 5 patients. During protocol we assigned primary haemostatic measures, laboratory examinations, hemostatic properties, etc.
Postoperative mortality (12%) and morbidity was low (for both groups). The assessment of the haemostatic properties was "very good/good" in 95% of the cases for TC and in 90% of the cases for TS. There were specific applications for TS, which couldn't be covered by TC and inversely.
A very careful surgical technique and the application of TS or TC to seal off the raw surface of the liver avoided in most cases the complications of oozing/biliary leakage. TC or TS are very effective in liver surgery and they have an own fields of application.

### P073

### BILIARY COMPLICATIONS AFTER EXTENDED LEFT HEPATECTOMY

O.Soubrane, F.Grozier, PP.Massault, B.Dousset, J.Pitre, D.Houssin, Y.Chapuis.

Clinique Chirurgicale, Hôpital Cochin, Paris, France.

Both safety and feasibility of major hepatic resection have been considerably improved by the use of hepatic vascular exclusion (HVE). However, extended left hepatectomy remains a challenging procedure with increased risks of biliary damage. We therefore retrospectively reviewed our experience of such hepatic resection with particular regard to biliary complications.

From Jan 1986 to Nov 1994, out of 320 hepatic resections, 13 patients (6 men, 7 women) of mean age 49.2 ± 12.3 yrs (range: 25-67) underwent a left extended hepatectomy, i.e. resection of segments II, III, and IV plus either segments V-VIII (n=6), or segments I-V-VIII (n=5) or segments I-V-VI (n=1) or segments V,VI,VIII (n=1). Indications were the following: intrahepatic biliary carcinoma (n=5), hepatocellular carcinoma (n=3), secondary malignant tumours (n=3) benign tumours (n=2). Portal fibrosis was present in 3 cases and cholestasis in 1. The procedure always began with section of the left portal pedicle. The parenchymal transection was done under HVE in 11/13 cases (median duration: 50 min, range: 5-70). The portal pedicle of the left paramedian sector (i.e. supplying segments V and VIII) to be ligated was localized using a probe introduced through the left bile duct. When resection was completed, methylene blue was injected in the bile duct to detect leakages from the transected surface. Two patients also had bile duct resection and hepaticojejunostomy on the bile ducts of segments VI and VII.

No postoperative death occurred. Six biliary complications occurred in 5 patients: bile leak (n=5) and stenosis of the right bile duct (n=1). Bile leakages resolved spontaneously in less than 3 months in 2 patients, required percutaneous drainage in 2 patients and reoperation in 1 patient. Right bile duct stenosis required hepaticojejunostomy 9 months after hepatectomy. Biliary stenosis recurred and was treated by transhepatic dilatation. In these patients, early CT-scan did not show any parenchymal ischemia of the remnant liver. The 2 patients who had intrahepatic hepaticojejunostomy did not experience any biliary complication.

In conclusion, this study shows that the incidence of biliary complications is especially high after left extended hepatectomy. The mechanism which accounts for bile leak is likely to be an ischemia of the right lateral bile duct due to intraoperative injury of its blood supply.

### P074

### THE SYSTEMIC CYTOKINE RESPONSE TO LIVER SURGERY UNDER TOTAL VASCULAR EXCLUSION

JM Badia, L Ayton*, T Evans*, A Carpenter*, G Nawfal, H Kinderman*, G Zografos, S Uemoto, J Cohen*, NA Habib.
Dep. of Surgery and Dep. of Infectious Diseases and Bacteriology*, Royal Postgraduate Medical School, Hammersmith Hospital, London.

The biological pattern of liver failure after liver surgery resembles that of sepsis. Several cytokines are involved in the acute phase response to sepsis and surgery.

*Aim.* To investigate the systemic cytokine response to major liver surgery as the basis for potential novel therapeutic strategies.

*Methods.* Thirteen patients undergoing elective liver surgery entered the study. All patients underwent operation using total vascular exclusion (TVE) of the liver. Samples of venous blood were taken from a central line preoperatively, intraoperatively six minutes after TVE, and during the first four postoperative days. Endotoxin, interferon gamma (IFN), Tumour Necrosis Factor alpha (TNF), Interleukin-1 (IL-1) and Interleukin-6 (IL-6) were measured. A clinical scoring system was used to evaluate the outcome of the patients during the postoperative periou.

*Results.* There were 6 right hepatectomies, 2 right extended hepatectomies, 4 segmental resections and 1 left hepatectomy. Time of total vascular exclusion of the liver was 32 ± 2 minutes. Endotoxin levels were raised in 3/13 patients before surgery and in 6 patients during the postoperative period. TNF concentrations were undetectable. IFN and IL-1 responses followed a low and inconclusive pattern. IL-6 showed a significant increase from 6 h after operation to the third postoperative day, peaking at 699 ± 277 pg/mL at 24 h after surgery. Two patients who died had the highest levels of postoperative IL-6. The intraoperative IL-6 level correlated with the change in the organ dysfunction score.

*Conclusion.* There is a marked systemic IL-6 response to liver surgery under TVE that correlates with the postoperative outcome and might be used as an indicator of the response to specific treatments in this type of surgery. Therapeutic interventions which minimise the IL-6 response to major liver surgery may be of value.

Dr.Badia had the grant FISS 94/5024 (Ministry of Health of Spain).

### P075

### STOMACH AND DUODENAL ULCERS IN PATIENTS WITH CHRONIC LIVER DISEASE

A.A.Sheptulin
Center "Gastroenterology (therapy)" Sechenov Moscow medical academy, Russia

In modern medical literature the problem of gastroduodenal ulcers and hepatic leisions combination is described insufficiently and contradictory. It seems, that different opinions are due to heterogenity of these patients Several groups of patients may be determined. The first includes patients with primary gastroduodenal ulcers and secondary (e.g. - alcoholic) hepatic leisions. The second group is patients with preexisting chronic liver disease and secondary ulcers, that appeared due impaired inactivation of histamine and gastrin, mucosa hypoxia and mucus generation disturbances. Clinical manifestations may be obliterated and atypical with sudden gastrointestinal bleedings. The next group is presented by patients with chronic hepatitis or hepatic cirrhosis and steroid ulcers. Often these ulcers are acute and tend to bleed. Gastroduodenal ulcers in patients with terminal st age of liver cirrhosis should be determinad as "stress ulcers". Such ulcers often lead to massive gastrointestinal bleeding.

Patients with combination of gastroduodenal ulcers and chronic liver disease should be divided to different groups to avoid contradictions in assesments.

### P076

### SURGERY OF HEPATIC HAEMANGIOMAS

V. Vishnevsky, R. Ikramov, A. Gavrilin.
A. Vishnevsky Institute of Surgery, Moscow, Russia

Of 155 evaluated patients with hepatic haemangiomas (HH), 105 were operated. Because of the danger of rupture and severe derrangement of hepatic haemodynamics, surgical treatment — hepatic resections (HR) were indicated mainly in patients with extensive HH, involving half or more of the organ (35). Operations were also perfomed for less extensive HH, involving 2 — 3 segments, measuring 10 — 15 cm. (46). All the patients grouped above complained of persistent hepatic pains or had various complications (haemobilia — 3,suppuration of the tumor 3). During doppler and radionuclide investigation, in 65 patients, signs of abnormalities of portal circulation of the excised specimens revealed parenchymatous changes resulting from the presence of the tumors. The main forms of resection for such HH were subtotal excision of the tumor or left cavanous lobectomy. Patients with HH <5 cm. were not operated, however they were followed up dynamically. Those with lesions 6 — 8 cm. were subjected to either operative treatment or to roentgeno-endovascular procedures, depending on age, risk factors, presence of other surgical diseases.The safely of HR for HH is significantly increased by the use of the technique developed in our institute, which involves endovascular occlusion of the arteries supplying the tumor preoperatively, and intraoperatively the use of modern technology (ultrasonic surgical dissector, apparatus for fast reinfusion of blood, intraoperative ultrasonography,flame and pneumothermo coagulators), which reduce operative trauma and the risk of injuring large vessels. Only one death was observed amongst patients who unserwent extensive HR.

### P077

### THE PREVALENCE OF H. PYLORI IN CIRRHOTIC PATIENTS

C. Agelopoulos[2], A. Constantinidis[1], A. Touliatou[2], P. Stampolos[1], C. Paraskeva[1]., B. Papanastasopoulos[2], N. Skandalis[1]
1. Gastrenterology Department
2. Second Pathological Department
General Hospital of Athens, Greece

It is well known the correlation of H. pylori (HP) and peptic ulcer disease (PUD) and the high prevalence of HP in healthy Greek population (70%) as well.
It is also well known the increased frequency of portal gastropathy (PG) and PUD in cirrhotic patients (CP). But it is not known the prevalence of HP in this subset of population.
For this purpose we studied 46 CP who didn't have any evidence of PVP. 39 were males and 7 females Mean age 58,3±10 years (37-78). 23 had alcoholic cirrhosis, 10 posthepatic HBV, 9 posthepatic HCV, 1 posthepatic HBV and HCV cirrhosis, 1 primary billiary cirrhosis, 1 cryptogenic and 1 autoimmune cirrhosis. 16 of pts were classified as having Child A cirrhosis, 20 child B, and 10 child C. All pts had an upper GI endoscopy and biopsies for CLO test were taken. The prevalence of HP and its correlation with cause and severity of cirrhosis (Child system), the patient's age, the sex and the coexistence of PUD and PG were studied. For statistical analysis chi-square test was used.
H.P. was detected in 6 pts (13,5%). All were males 5 were classified as child B and one as child A. In HP positive pts 4 had posthepatic B cirrhosis and one alcoholic cirrhosis. 5 pts had mild PG while in Hp-negative group of pts 4 had severe PG, 34 mild PG and one had duodenal ulcer.
In conclusion, the prevalence of HP in CP is low (13,5% versus 60% in Greek population) but it is mildly increased in pts with child B cirrhosis (p=0,005) and in pts with posthepatic B cirrhosis (p=0,002). Finally there is no correlation with age, sex and coexisting PG or PUD.

### P078

### ROLE OF PROSTANOIDS IN REGULATION OF HEPATIC BLOOD FLOW AND MICROCIRCULATION IN CHRONIC HEPATITIS AND CIRRHOSIS PATIENTS

A.Yagoda, I.Shestopalova, Y.Radtsev
Department of Internal Diseases No. 1, Medical Academy, Stavropol, Russia

Products of unsaturated fatty acids metabolism - prostaglandins (PG), prostacyclin, thromboxane - can contribute to regulation of the hepatic blood flow. The exact role which these vasoactive substances play in the impairment of hepatic circulation in chronic hepatitis (CH) and cirrhosis (C) patients remains unclear.
A total of 62 patients with chronic persistent hepatitis (CPH, 27), chronic active hepatitis (CAH, 13) and cirrhosis (C, 22) were studied. Hepatic blood flow (HBF) was tested using the dye (ueviridin) dilution technique, and the condition of the microcirculation was assessed by a biomicroscopy of the conjunctival blood vessels with subsequent calculation of the «conjunctival index» (CI). In liver specimens, the levels of prostaglandins (PG) - PGE, PGF2a, 6-keto-PGF1a, thromboxane B2 (TB2), along with their synthesis from 3H-arachidonic acid were assayed by the RIA. 17 healthy volunteers served as controls.
A significant decrease in HBF with boost in microcirculatory disorders level (CI), consistent with the severity of the disease, was observed in the patients with CH and C. The patients with CAH had decreased tissue contents and/or synthesis of PGE, 6-keto-PGF1a, and increased levels of PGF2a synthesis (compared to those with CPH). The relative increase in the levels and synthesis of TB2, PGF2a, along with decrease in PGF1a and PGE contents and/or synthesis took place in C patients. In all the groups tested (in CAH especially), the pattern of correlative interrelationship of TB2 and PGF2a with the CI parameters was straight, and with the HBF index - reverse. On the other hand, tissue levels and synthesis of PGE and 6-keto-PGF1a were directly related to the HBF index, and reversely - to the CI indicator.
The results of the study give ground to the possibility that the groups of prostanoids could alternatively affect the hepatic blood flow and the microcirculatory vessels. The impairments of microcirculation and HBF in CAH and C patients could be also related to the unbalanced hepatic synthesis of PGs and thromboxane.

### P079

### IMMUNOLOGICAL MONITORING IN THE SURGICAL TREATMENT OF LIVER CIRRHOSIS

S.Chooklin, A.Perejaslov
Department of Surgery, Lviv Medical Institute, Ukraine

The results of the surgical treatment of 45 patients with the liver cirrhosis were observed. The postoperative mortality was 17,8%, complications - 43,2%, in which purulent-septic complications were more often met in virus cirrhosis. In the complex investigation the immunological criteria prognosis of the outcome surgical intervention was recommended. Analysis of the death cases after operation gives the possibility to determine more significant decrease of T-lymphocytes level (0,420±0,039 · $10^9$/l) in the blood, on the given category of patients, with the hyperproduction of IgG (22,56 ± 3,91 g/l) and IgA (3,03 ± 0,63 g/l). At the same time, IgM - the marker of the primary immune answer - was decreased (1,00 ± 0,19 g/l). The deep depression of the neutrophile phagocytic function was observed (phagocyte index - 44,38 ± 2,47; phagocyte number - 2,21 ± 0,19). The disturbance in the immunoregulatory lymphocytes with hyperstimulation production immunoglobulins of the G (26,45 ± 4,57 g/l) and A (6,53 ± 0,91 g/l) classes, the complement decrease and deficit of the polymorphonuclear leukocytes phagocytic function progressed after operation. In these cases, immunoglobulins of the main classes intensified the spontaneous aggregation of thrombocytes and also aggregation, which had been evoked by ADP, adrenaline and collagen. Therefore, in obvious disturbances in immunoregulation of patients with liver cirrhosis, the danger of the acute thymic failure arised in the postoperative period, what should be taken into consideration in the preparation to the surgical intervention.

### P080

### DIAGNOSTIC IMAGING OF THE LIVER, BILIARY TRACT AND THE PANCREAS: CHANGES IN ROUTINES DURING THE LAST TWO DECADES

T.E. Gudmundsen, B. Vinje, H.K. Pedersen, H. Østensen, T. Pedersen
Dept. of Radiology, Central Hospital of Buskerud, Drammen, Norway

Purpose of the study:
The study was conducted to evaluate whether new imaging techniques had influenced or even substituted older routines for liver, biliary tract and pancreatic diagnostics.

Material and methods:
For the period between 1975 and 1993 files from five Norwegian hospitals were analysed retrospectively.

Results:
In all hospitals, X-.ray based examinations of the biliary tract, oral cholecystography and intravenous cholangiography, disappeared completely as ultrasonography (US) came into use. Likewise, an extensive use of scintigraphic examinations of the liver disappeared after introduction of both computed tomography (CT) and US, which in turn reduced the number of CT examinations of the liver substantially. With regard to pancreas, CT being the method of choice for some years also seems to have been replaced by US.

Conclusion:
The present study confirms that new imaging techniques rapidly are adopted into clinical routines not only as a supplement, but as a real substitute for older, more risky and more unreliable methods.

### P081

ROLE OF LYMPHSORBTION IN THE TREATMENT OF LIVER CIRRHOSIS WITH ASCITES

A.M.Granov, T.L.Pirtzchalava
Department of Surgery, St.Petersburg Research Institute of Roentgenology and Radiation Therapy, Russia

This study was performed to assess the feasibility and success of combination of lymphosorbtion and medical therapy vs medical therapy alone in liver cirrhosis complicated by ascites. Combined treatment was utilized in 28 patients with Child-Pugh class B (5 pts) and C (23 pts) liver cirrhosis (Group 1). Lymph purification and reinfusion (500 ml to 1500 ml dayly) was performed using chronic external surgical catheterization of the thoracic duct. The carbon adsorbent with fibers of $8x10^{-3}$ to $12x10^{-3}$ mm in diameter and $2m^2/g$ external geometric surface was used for lymph purification. Control Group 2, 7B and 29C class of cirrhosis patients, received only conventional medical therapy. In 2 weeks after beginning of the treatment, decrease of ascites was seen in all pts of Group 1 vs 70% of Group 2. Their diuresis was +1100 ml/day vs +200 ml/day and their weight loss was 7 kg vs 1.5 kg, respectively. Gastroesophgeal varices decreased in 50% vs 0%, and encephalopathy diminished in 90% vs 30% of pts, respectively. These data showed that lymphosorbtion combined with medical therapy is more effective than medical therapy alone in the management of patients with liver cirrhosis and ascites.

### P082

**MODULATION BY CYTOKINES OF HEPATOCYTE GROWTH FACTOR/SCATTER FACTOR PRODUCTION BY FIBROBLASTS**

**MCA Puntis, MB Hallett, and WG Jiang**
University Department of Surgery, University of Wales College of Medicine, Cardiff CF4 4XN, UK

Hepatocyte growth factor/Scatter factor (HGF/SF) is produced mainly by fibroblasts. This study was to determine the regulatory effect of cytokines on the production of HGF/SF by fibroblasts.

Human fibroblast cell-line, MRC5 was treated with cytokines for 24 hours and the HGF/SF level from the MRC5 conditioned medium measured by a MDCK bioassay. Interleukins(IL)-1,2,3,4,5,6,7,8,10,11,12, tumour necrosis factor (TNFα), transforming growth factor (TGFß), platelet derived growth factor (PDGF), insulin like growth factor (IGF-I), GM-CSF, Interferon-γ, and epidermal growth factor (EGF) were tested. A unit per ml (U/ml) was the minimum amount of human recombinant HGF/SF causing MDCK cell scattering (equivalent to 0.5ng/ml).

IL-1ß, IL-3, IL-6, IL-8, IL-12, EGF, and TNFα stimulated HGF/SF secretion, giving 353.0±9.0*, 529.6±6.3*, 176.0±8.5*, 353.1±4.0*, 194.0±1.9*, 529.7±8.6*, and 529.6±11.0* U/ml (mean±SEM) respectively. TGFß, IL-7, IL-10, and IL-11 inhibited the secretion, giving 33.1±4.0*, 64.0±7.9, 48.0±5.0*, and 64±5.0 U/ml respectively. * indicates p<0.05 (Student t test) compared with control level, 88±4.1U/ml. GM-CSF, PDGF, IFNγ, IL-2, IL-4, IL-5, and IGF-I had no effects.

The MRC5 produced HGF/SF was identified by neutralisation with anti human HGF/SF antibody and also confirmed by Western blotting.

We conclude that MRC5 human fibroblast production of HGF/SF can be regulated by cytokines, some are stimulatory while others inhibitory. This may be important in the regulation of generation of HGF/SF *in vivo* and therefore regulation of cancer cells in the metastasis in the liver.

### P083

HEPATIC ADENYLATE AND GUANYLATE CYCLASES SYSTEMS IN CHRONIC HEPATITIS PATIENTS AT DIFFERENT STAGES OF HBV-INFECTION

A.Yagoda, Y.Radtsev, N.Budagov
Department of Internal Diseases No. 1, Medical Academy, Stavropol, Russia

26 patients with HBsAg-positive chronic hepatitis (CH) were studied. Hepatic biopsies were taken and adenylyl and guanylyl cyclase (AC and GC) activities, along with cyclic nucleotides (CN) - cAMP and cGMP - levels were estimated. Contents of the primary products of lipid peroxidation (LP) - conjugated diens (CD) were also assayed. The stage of HBV development was determined using the serum replication markers (HBeAg or/and anti-HBc IgM), or virus integration indices (anti-HBe, anti-HBc IgG).

In 8 patients with signs of virus replication AC activity and cAMP contents in liver tissue were lower than in those who had the integrative stage of B-viral infection (p1<0.02; p2<0.001). GC activity in these groups did not differ, while tissue cGMP levels in patients with virus replication was higher than in those who had blood markers of the integrative stage of the disease (p1,2< 0.05). The AC/GC and cAMP/cGMP ratios in CH patients within the replicative stage of HBV were decreased, and the tissue levels of CD was enhanced in comparison with both groups of patients with the integrative stage of virus development.

Thus, the stage of virus replication in CH patients could be characterized as leading to the disproportions in intracellular messengers' systems. The exact mechanism, by which the replicating virus can alter the cyclases' activities, is probably related to either direct or indirect damage of the hepatocytes' membranes and excessive accumulation of lipid peroxides, which leads to inactivation of the membrane-bound enzymes (adenylyl-cyclase and, probably, cGMP phosphodiesterase).

### P084

URSODEOXYCHOLIC ACID VERSUS INTERFERON ALFA + URSODEOXYCHOLIC ACID IN CHRONIC C HEPATITIS

Leri O.*, Sinopoli M.T.*, Pugliese S.*, Paggi A.**, De Luca D.*, Paparo B.S.*
* Department of Infectious Diseases University " La Sapienza" Rome Italy
** II Internal Medicine University " La Sapienza" Rome Italy

The aim of this study is to compare therapeutical effects of natural interferon a (IFNa) versus IFN-a plus ursodeoxycholic(UDCA) in chronic C hepatitis. 19 patients (8 M/11F mean age 59,17 ) with chronic HCV infection were studied. All patiens were positive to HCV antibodies (ELISA 1st and 2nd generation Ortho-Sorin) and had HCV-RNA evaluated by Polymerase Chain Reaction (PCR). 5 F/5M (mean age 56,40) were treated wih placebo. The indexes of liver function were monitored before, during and 4 weeks after the treatment. In IFNa group, AST decreased from mean 196,30 u/l (SEM 39,24) to 102,88(10,96) (- 47,61% p<0,01), ALT from 199,13 u/l (41,81) to 90,38 (12,76)( - 54,61% p<0,05) and gGT from 77,25u/l (6,33) to 49,5 ( 3,59) (- 35,92% p<0,05). In UDCA+IFNa group, AST decreased from mean 101,71 u/l (15,29) to 47 (9,69) (- 53,79% p< 0,05), ALT from 140,29 u/l (35,92) to 59 (15,62) (-57,94% p< 0,05) and gGT from 47,71 u/l (8,15) to 23,89 (3,16) (-49,99% p<0,05). In placebo group serum transaminases and gGt values were only slightly reduced (ns). Transaminases and gGT were expressed as mean ± SEM. During the follow up the improve of those indexes was statistically significant in both groups ( Wilcoxon test); by contrast, performing the Kruskal-Wallis test no statistically signicant differences were observed among the two groups. So, we coudn't confirm previous observations showing IFNa +UDCA the most effective trial in controlling hepatitis C disease activity both improving the liver function tests and lowering relapse rate after stopping the therapy.

### P085

HEPATITIS FULMINANS AS A CONSEQUENCE OF ALCHOCOLIC LIVER CIRRHOSIS - case review

V.Kostic, Z.Rankovic,Lj.Konstantinovic,M.Krstic, N.Govedarevic,V.Zivkovic
Clinic for infectious Diseases Medical faculti Universiti of Nis, Yugoslavia

Patient D.M., 45 y.,old,alchocolic more than 20 years has been hospitalized because of the alchocolic liver demage.Last time,patient has been treated for 5 months (1994) with diagnosis:Cirrhosis hepatis decompensata,with high doses of blood preparates.These preparates were produced by an institution which routinelly performs analyses for HBV,and,in recent time for HCV infection.Such intensive treatment resulted in slight improvement,so patient was transferred to house care.Four months later,the patient has been hospitalized again because of worsening of liver disease (icterus,dyspepsia,pain below the right costal arc).Biochemical findings were charracteristic for the acute form of disease (abnormally high level of aminotranspheraze and bilirubine). The synthetic liver function was severelly demaged:haemostatic factors were 0-20% of the nor--mall value.

Marker screening pointed to acute B hepatitis infection.The patient has been submitted to an intensive treatment (plasmaferesis,vitamines, blood derivates) and there was no affect on the evolution of liver disease.After 7 days,the patient fell to comma,and on 9th dayhe died.Postmortem performed liver byopsy showed cirrhitic altered liver tissue with rare normal hepatocytes.

### P086

**PRIMARY INTRAHEPATIC LITHIASIS SURGICAL TREATMENT OF A RARE WESTERN'S WORLD DISEASE**

M.C.C. Machado, P. Herman, T. Bacchella, V. Pugliese, J.E. Monteiro da Cunha, H.W. Pinotti.
Department of Gastroenterology, Hospital das Clínicas-University of São Paulo Medical School; Brazil.

Thirty nine patients with intrahepatic lithiasis (IHL) were treated between 1974 and 1993. There were 21 women and 18 men with a mean age of 38 years (range 11 to 75). The clinical presentation was of cholangitis, and the most frequent symptoms were abdominal pain (100%), jaundice (97.4%) and fever (87.1%). More than half of the patients had been already submitted to a biliary surgery. Routine laboratory tests showed raise in serum gamaglutamyl-transferase (88%), alkaline phosphatase (78%), bilirrubins (48%), aminotransferases (47%) and leukocytosis (37%). Radiologic investigation with ultrasonography, CT scan and cholangiography were performed with 82.1%, 100% and 97% of sensitivity, respectively. In 64.1% of the cases stones were bilateral and in 23.1% were located only in the left lobe. We adopted a systematic approach in the treatment of these patients with a tailored surgery according to the presentation of the disease. Surgery was performed in 37 patients, including biliary drainage procedures and hepatic resections. Two patients with liver cirrhosis, were submitted to endoscopic papilotomy. Biliary infection was present in 86% of the cases. There was no operative mortality. Best late results occurred in patients with unilobar disease with 92.8% of good results, specially in cases where an hepatic resection was performed. In bilateral disease symptoms recurrence occurred in 47.5% of the cases. Overall good results were observed in 70.2% of the cases after a median follow up of 46 months.

### P087

**DIAGNOSIS OF NON-VASCULAR BENIGN LIVER NEOPLASMS.**

Moreno-Sanz C., Jiménez C., Rico P., De la Çalle A., Hidalgo M., Hernández D., Alvarado A., and Moreno-Glez E.
General and Digestive Surgery Department. Hospital "12 de Octubre". Madrid. Spain.

**INTRODUCTION:** Hepatic Adenoma (HA) and Focal Nodular Hyperplasia (FNH) are the most common non-vascular origin benign lesions of the liver and other neoplasias are extremely rare. Regression of HA after withdrawn of oral contraceptives has been documentedm but malignant transformation is possible. Moreover, it is not always possible to differentiate with certainly between HA, FNH and well differentiated hepatocarcinoma. The aim of this study is to report our experience with this type of tumors.
**PATIENTS AND METHODS:** Betwenn 1978 and 1994, 26 non-vascular origin benign liver tumors have been resected of 84 benign hepatic tumors. The neoplasms were: Hepatic adenoma (11), Focal Nodular Hyperplasia (12), Biliar Adenoma (1), Mielolipoma (1) and Angiomyolipoma (1).
We review the clinical features, laboratory tests, and imaging studies (US, CT, angiography and isotope scan). We obtained histopathologic studies prior to definitive surgical treatment in 14 patients. All patients underwent surgical treatment.
**RESULTS:** The mode of presentation and laboratory results were unspecifics. The differents imaging studies revealed "hepatic mass". The sensitivity for the imaging studies were: US: 0% for all the cases; CT: 11.1 % for HA, 0% for FNH and 7.6% for both lesions; angiography: 20% for HA; isotopic scan: 0% for HA and biopsy: 25% for HA, 50% for FNH and 37.5% in group. The resectability rate was 100%. Only two patients presented morbidity (pleural effusion) and there was no mortality. Only the postoperative anatomopathologic report gave an accurate diagnosis. No recurrence has been detected.
**CONCLUSIONS:** The low sensitivity for the different studies makes difficult the preoperative diagnosis. We recommend surgical treatment of these lesions

### P088

**DIAGNOSIS AND TREATMENT OF PYOGENIC HEPATIC ABSCESS: STUDY OF 51 CASES**

P. HERMAN, V. PUGLIESE M.Z. SALEM, M.A.C. MACHADO, T. BACCHELLA, J.E.M. CUNHA, M.C.C. MACHADO, H.W. PINOTTI.
Department of Surgery - University of São Paulo, Brazil.

Pyogenic hepatic abscesses are uncommon. We report our findings in 51 patients with pyogenic liver abscess treated from 1975 through 1992. Twenty-eight patients were men and twenty-three were women. The median age of patients was 46 years (range, 13 to 77 years). Fever was present in 100% of patients, abdominal pain in 58.8% and jaundice in 39.2%. Twenty eight patients (54.9%) had leukocytosis; 45% hiperbilirrubinemia and 35.3% high serum level of alkaline phosphatase. The most common cause of abscesses was biliary tract disease (66%), followed by portal origin (14%), idiopathic (10%), endocarditis (6%) and trauma (4%). The culture of abscesses was postive in 82.5% of patients with prevalence of gram negative bacteria. Thirty-seven (64.7%) were surgically treated and thirteen underwent percutaneous drainage with 90.4% and 69.2% of good results, respectively. Mortality was 9.6% in the surgical group and 0% in the percutaneously drained group.

A review of literature of this condition and a discussion about the diagnosis, treatment and etiopathogenesis are presented.

### P089

ACUTE ABDOMEN DUE TO TRAUMATIC RUPTURE OF AN AMEBIC LIVER ABSCESS

C.Vlahos, A. Mazis, K. Skoulas, C. Koutos, P. Myrillas, S. Rathosis

Department of Gen. Surgery, "Agios Andreas" Gen. Hospital, Patras, Greece.

Although the major manifestations of amebiasis are enteric in location the pathogenicity of the organism is not limited to the intestinal tract and other anatomic sites may be affected. We present a case of hepatic amebic abscess which ruptured in the abdominal cavity following a motorvehicle accident.

The victim was a 52 year old male driver, who was admitted in the emergency room an hour after the accident, in unstable condition. The initiative examination showed pain and tenderness in the upper abdominal wall, hematocrit 37%, BP: 110/80, RR: 25, P:120. The ultrasound examination showed the presence of fluid in the abdominal cavity while the peritoneal lavage resulted in the discovery of blood, bile and puss.

According to the medical history of this patient, he had fever (38 C) and pain in the right upper quandrant of the abdominal wall, for the last 3 weeks. He had being receiving antibiotics with no positive results.

The patient was admitted to the operating room for exploratory laparotomy.

We found a large ruptured central Liver abscess in segment 8, with a diameter of 12 cm. There were no other injuries to the other abdominal viscera. We performed a partial closure of the abscess cavity and inserted a drainage tube into it, followed by suction and irrigation of the abdominal cavity. The postoperative period was normal, without any complications and the patient discharged from the hospital on the 13th day.

The Liver is the most commonly involved extraintestinal organ in amebiasis, and hepatic abscess is a major complication, which if untreated, often proceeds to a fatal outcome. Amebic abscess of the Liver is a well-known entity that is frequently observed in many countries of the world; if amebic abscess of liver is diagnosed while confined, present modes of treatment are curative.

### P090

PROSTAGLANDIN E1 AND E2 EFFECTS ON CYCLIC NUCLEOTIDES LEVELS IN LYMPHOCYTES IN LIVER DISEASES

A.V.Yagoda, R.A.Rekvava, Y.A.Radtsev

Department of Internal Diseases No. 1, Medical Academy, Stavropol, Russia

Suppressive effects of the prostaglandins (PGs) upon lymphocyte functioning are believed to be mediated by fluctuations of the intracellular levels of the cyclic nucleotides (CN) - cAMP and cGMP. 78 patients with chronic persistent hepatitis (CPH), chronic active hepatitis (CAH), cirrhosis (C), along with 20 healthy subjects were studied. In vitro PGE1 and PGE2 (10-8M, 37oC, 30 min) effects on cAMP and cGMP contents in common pool and T- and B-cells populations, along with effects of intravenous PGE1 derivate, vasoprostane (0.5 mkg/ kg), and PGE2 analogue - prostenone (8 mkg/kg) were studied.

Cyclic AMP levels in lymphocytes of CPH patients were within the normal values, while in CAH and C patients they were found decreased ($p<0.05$). cGMP contents and cGMP/cAMP ratio did not significantly differ from that of the control group. T-lymphocytes of the patients with CAH and C had higher cAMP levels than B-cells, in comparison with those of healthy subjects and CPH patients. PGE1 and PGE2 in vitro caused significant increase in cAMP levels and cAMP/cGMP ratio in lymphocytes of the healthy people and CH patients. Adenylylcyclase (AC) system of the C patients showed no reaction to exogenous PGs at all. In vitro loading of the T-cells with PGE1 and PGE2 resulted in high cAMP levels only in healthy subjects and CHP patients, while all the groups tested showed marked B-cells response. cGMP levels in common pool and in lymphocyte subpopulations did not respond to PGs load.

Decreased cAMP levels in patients with CAH and C reflect high «immunological activity» of the lymphocytes, especially of the B-cells. The possibility of the PG-induced B-cell suppression in these patients combines with their limited impact on cellular immunity. «Inertial» pattern of the AC-cAMP system reaction to the PGs, refers to inability of the PGs to control the lymphocyte function in patients with cirrhosis as a possible explanation and could be regarded as one of the factors affecting the disease progrediency.

### P091

LIVER SURGERY AND FUNCTIONAL HEPATIC RESERVE REFLECTED BY ANTIPYRINE METABOLISM

A.Grieco, S. Marcoccia,S Alfieri*.A Giancaterini,G Addolorato, F Crucitti*. G Gasbarrini. Internal Medicine & *Clinical Surgery Depts. Catholic University ,ROME,ITALY

Surgery is the only approach that offers the possibility of a radical cure for primary and metastatic liver tumors, but patients with cirrhosis are considered poor candidates for hepatic surgery. We used the antipyrine metabolism test to investigate the prognostic role of cirrhosis in patients subjected to hepatic resection. Patients and methods: Twenty-three patients (11 females,12 males, range 18-64 yrs:) scheduled for hepatic resection were studied: 6 had liver metastases without cirrhosis (Group A); 8 bearing hepatic echinococcosis (Group B); 9 had hepatocellular carcinomas and cirrhosis (Group C). Antipyrine metabolism tests (18 mg/kg in water p.o., blood samples drawn 3. 24 hrs after administration, spectrophotometric measurement of serum levels) and routine liver-function tests were performed in all patients before surgery and on post-operative days 7 and 28 All patients were operated on by the same surgical team. Results: No significant differences were observed among the three groups as far as pre-and post-operative liver function indices were concerned. Mean pre-operative values for antipyrine clearance were not significantly different among the groups: Group A: 34.84 ± 5.8 ml / min; Group B: 30.34 ± 2.7 ;Group C: 22.3 ± 2.7 , but the mean half-time for Group C (27.9 ± 4.9 hrs) was significantly greater than those for Groups A and B (respectively 14.4 ± 1.8 hrs and 14 5 ± 1.5 hrs) (Scheffe F test: 4.3). On post-operative day 7, clearance was increased in GroupA (39.12 ± 4.6 and B ( 31.6 ± 5.5) and decreased in Group C (18.1 + 4 4 .Scheffe F 5.17). Three patients from the latter group died from liver failure during the post-operative period (1 post-segmentectomy. 2 post-bisegmentectomy).Conclusions: Cirrhosis represents a crucial pre-operative risk factor even for limited hepatic resection. The increase in hepatic microsomal oxidative activity that normally occurs during the early post-operative period is not observed in patients with cirrhosis.

### P092

PLASMA CHOLESTEROL (CHOL) IN CRITICALLY ILL SURGICAL PATIENS.

I. Giovannini, G. Nuzzo, G. Boldrini, C. Chiarla, F. Giuliante.

Dept of Surgery (Chirurgia Geriatrica), Catholic University School of Medicine, Rome, Italy

It has long been known that hypocholesterolemia characterizes critical illness and poor prognosis, however this phenomenon has never received adequate explanation. This study has been performed to asses the correlates of CHOL in 530 measurements (full SMA-12, hematocrit, coagulation profile) in 145 patients after major abdominal surgery or in concomitance with major complications. CHOL correlated well with prothrombin activity (PT, % of standard), cholinesterase, iron binding capacity ($r^2$= 0.41 to 0.20) percent hematocrit (HCT, $r^2$= 0.14) and alkaline phosphatase (ALP, nv 79-280 UI/L; $r^2$= 0.37) ($p \ll 0.01$ for all). The following "simplest best fit" was selected by regression analysis:

$$CHOL = -96 + 1.511(PT) + 2.701(HCT) + 0.071(ALP) \quad \text{total } r^2 = 0.57,\ p \ll 0.01$$

These data indicate that there is a cumulative effect of factors commonly associated with poor prognosis (inadequacy of hepatic protein synthesis, hemorrage) in lowering CHOL; this may contribute to explain the negative prognostic value of hypocholesterolemia and to improve use of CHOL for clinical purposes. Quantification of the relationship between CHOL and ALP, as provided in this study, may additionally help in the evaluation of the effect of cholestasis on CHOL.

### P093

SURGICAL TREATMENT OF LIVER ADENOMAE

Alperovich B.I.,Stupakov I.N.

Siberian Medical University,Tomsk,Russia

Adenomas rarely happen to he among tumours. 4o5 liver resections were made in the Siberian Hepatological Centre,1o2 - with different tumours,7o - with innocent tumours.28,6% patients **with liver tumours** and 27,1% patients with innocent tumours had adenoma.
19 patients with liver adenoma were observed. They were made resections.In 9 cases the tumours were identified by palpation,in 1o case the tumours were sound with the help of adjuvant methods of examination.
It was sound that liver adenoma is developing with small manifestation and can be discovered before operation by the ultrasound investigation,computer thomography and angiography. Liver adenoma is a rare illness it should be operated.The choiceof operation is the liver resection.Cryosurgical techniques improve the results of the operation,quarantees less recidivation of the illness.

### P094

INTERVENTIONAL RADIOLOGIC PROCEDURES FOR TREATMENT OF PRIMARY BUDD-CHIARI SYNDROME

Ü. Santaş, T. Cumhur, T. Ölçer, B. Şahin, U. Yılmaz, E. Özdemir, A. Tezel

Departments of Gastroenterology and Radiology, Yüksek İhtisas Hospital, TURKEY

Membranous or segmental obstruction of vena cava inferior (Primary Budd-Chiari Syndrome) is uncommon, but treatable form of Budd-Chiari Syndrome. Interventional radiologic methods are curable and encouraging. In this article 8 primary Budd-Chiari cases who were treated by interventional radiologic methods are presented.
One patient was applied Rotacs membranotomy and balloon angioplasty, three patients balloon membranotomy and four patients percutaneous transluminal angioplasty (PTA). PTA was insufficient in one patient who was inserted metallic stent in right hepatic vein later. All interventional methods were successfully completed. Restenosis occurred in two patients after one and fourteen months. These patients had undergone to balloon angioplasty. Stent occlusion had occurred in one patient in whom surgical interventions were also unsuccessful. A second metallic stent was replaced in this patient. All cases were followed-up 5-30 months (mean 17 months).
All patients showed clinical regression, and all symptoms regressed. Clinical regression was also confirmed with ultrasonography, duplex Doppler and angiography. These results show that the interventional radiological methods can be effectively and safely used to treat the Primary Budd-Chiari Syndrome.

### P095

POSTOPERATIVE NUTRITION OF PATIENTS WITH HEPATIC CIRRHOSIS

A.Kozłowski, M.Pardela, K.Witkowski, J.Piecuch, W.Orkisz, J.Kultys
Dept. of General and Vascular Surgery, Silesian Medical School, Zabrze, Poland

A group of 38 patients with portal hypertension caused by hepatic cirrhosis who underwent surgical treatment was the subject of this study. 17 patients after oesophageal transsectomy and 19 after venous spleno-renal shunts were treated with intravenous infusions of glucose and fatty emulsion (group I) and the next 10 patients with only glucose i.v. (group II). Several routine biochemical tests like GOT, GPT, alcaline phosphatase, serum bilirubine and glucose, total protein and fat acids level in serum were done. Intravenous fat tolerance test and the nitrogen ballance measurement were also performed. As a result of these teste we came to conclusion that the postoperative i.v. fatty emulsion infusions didn't worsen the analysed biochemical factors. The higher rate of clearance of fat emulsion after operation in comparison with preoperative period indicates that fat retention in these patients didn't exist during the postoperative period. Nitrogen balance was almost the same in both groups of patients. The aquired results suggested that i.v. administration of fat emulsion didn't worsen the liver function in cirrhotic patients.

### P096

DIFFUSE FATTY LIVER: CORRELATION BETWEEN HEPATIC ULTRASOUND AND HISTOLOGY

L.S.Gheorghe,G.Aposteanu,Al.Oproiu,C.Gheorghe
Center of Gastroenterology,Fundeni Hospital, Bucharest,Romania

Ultrasound(US) is claimed as a useful non-invasive method in the assessment of diffuse fatty infiltration of the liver(DFL).We developed a study comparing the accuracy of hepatic ultrasonography with hepatic histology in the detection of DFL. 122 subjects enroled in the study underwent an US-examination using 3.5 and 5 MHz transducers followed by liver biopsy using Menghini and Tru-Cut needles within 4 days of the US assessment. There were 83 male and 39 female patients,mean age 46.3 yr.,allocated as ethanol abusers(71), diabetes mellitus(17),obesity(23),corticotherapy (8),parenteral nutrition(3).We defined 3 US grades for DFL.Histology was assessed and classified in three grades of DFL.US had a good sensitivity and specificity in the detection of DFL when compared with hepatic histology: overall accuracy 85% with 91% sensitivity,and 58% specificity.The accuracy of the detection was correlated with the degree of DFL,increasing to 97% in severe cases (grade III),p 0.001,and did not correlated with the ethiology of DFL.We conclude that US is a valuable non-invasive method for the detection and grading of the DFL.

### P097

**TOTAL HEPATIC BLOOD FLOW IN HEPATOSPLENIC MANSONIC SCHISTOSOMIASIS**

V. Pugliese, P. Herman, A.M.M. Coelho, M.S. Kubruzly, S.N. Sampietre, N. Kisilevzky, M.C.C. Machado, H.W. Pinotti
Department of Gastroenterology, University of São Paulo, Brazil

Schistosomiasis is a leading cause of portal hypertension in Brazil. Hepatic hemodynamic in this disease is still controversial. The authors have measured the hepatic blood flow in 12 patients with hepatosplenic mansonic schistosomiasis by analysis of the disappearence curve and hepatic extraction of indocyanine green (ICG).

Seven patients were female and five were male, with mean age of 40 years (±12,6). All patients have portal hypertension and history of upper gastrointestinal bleeding and the diagnosis confirmed by liver biopsy. ICG was injected in a peripheral vein (0.15 mg/Kg body weight) after drawing a baseline plasma sample. Simultaneous blood samples were taken from indwelling catheters in the aorta and the right hepatic vein at 1,2,3,4,5,7,9,11,16 and 21 minutes after the ICG injection. ICG plasma concentration was measured by spectrophotometry. Total hepatic blood flow was calculated on basis of the ICG clearence and hepatic extraction and corrected for the haematocrit.

The mean hepatic blood flow was 1349.49 ± 422.57 ml/min (range: 791.43 - 2200.00 ml/min).

The mean hepatic blood flow was normal. The great individual variability could be explained by heterogeneous liver and splanchnic circulation derangement caused by the disease.

### P098

**HEPATIC PORTAL INDEX IN PORTAL VEIN THROMBOSIS, HEPATIC CIRRHOSIS AND MANSONIC SCHISTOSOMIASIS**

V. Pugliese, P. Herman, C. Buchpieguel, F. Hironaka, M.C.C. Machado, H.W. Pinotti
Department of Gastroenterology, University of São Paulo, Brazil

Measurement of portal flow is of great interest for portal hypertension pathophysiology. The authors have studied the portal venous fraction of total hepatic blood flow in 97 patients with portal hypertension and in 26 normal volunteers by radionuclide angiography.

The patients were divided in four groups:
Group I - Portal vein thrombosis (PVT):13 patients; Group II - Hepatic cirrhosis (HC):14 patients; Group III - Hepatosplenic mansonic schistosomiasis (HMS):70 patients; Group IV - normal volunteers (NLS): 26.

Hepatic portal index (HPI) was calculated from the arterial and portal slopes of hepatic radioactivity *vs.* time curves after injection of 25 mCi (925mBq) of 99mTc-pertechnetate.

Results:

| | |
|---|---|
| Group I - PVT | mean HPI: 11.17% ± 9.00% |
| Group II - HC | mean HPI: 23.29% ± 8.52% |
| Group III - HMS | mean HPI: 40.08% ± 12.71% |
| Group IV - NLS | mean HPI: 55.15% ± 6.48% |

(*t* test $p < 0.05$)

Conclusion: HPI by radionuclide angiography is a non-invasive method useful in the diagnosis of etiology and complications in portal hypertension.

### P099

THE EFFICACY OF URSODESOXYCHOLIC ACID IN TREATMENT OF PATIENTS WITH INTERAHEPATIC CHOLESTASIS

A.L.Grebenev, D.Speychene, L.S.Grebeneva
Center "Gastroenterology (therapy)", Sechenov Moscow medical academy Russia

The influence of ursodesoxycholic acid drugs on manifestations of intrahepatic cholestasis was assesed in 20 patients (I4 with cholelithiasis, 4 with chronic active hepatitis and 2 with hepatic cirrhosis). The presence and degree of interahepatic cholestasis were determined under $^{75}$Se-methionine test, serum alkaline phosphatase, gamma glutamyl transpeptidase and bilirubin levels. After treatment with ursodesoxycholic acid patients felt better, the $^{75}$Se-methionine test findings tend to become better, and alkaline phosphatase and gamma glutamyl transpeptidase - normal. Obtained results show that treatment of hepatobiliary diseases, a companied with intrahepatic cholestasis is highly promising.

### P100

SIMPLE HEPATIC CYSTS: STUDY OF 15 CASES

Z.Plaitakis, C.Lambidis, E.Sigellaki, G.Karavolias, G.Meimaris, P.Papapavlou
2nd Surgical Department, General State Hospital of Nikea's "St.Panteleimon", Greece

Nonparasitic cystic liver disease is a rare clinical entity which arises from developmental abnormalities in the liver parenchyma of the intrahepatic and extrahepatic biliary system. We herein report our experience from the surgical management of 15 patients with simple liver cysts. Four men and eleven women (mean age 53 years) with simple hepatic cysts have been operated in our department during the last eight years. Preoperative diagnosis was made in 12 of them who operated electively, whereas 3 patients underwent an emergent surgery (rupture of the cyst in the peritoneal cavity which resulted in acute abdomen in two and in intraperitoneal haemorrhage and hypovolemic shock in one patient). The location of the disease had a right lobe predominance (10 out of 15 patients. The diameter of the cysts was between 5 to 22 cm. Three patients were subjected to total excision of the cysts, 9 patients to partial excision with drainage and 3 patients to drainage with omentoplasty. Pathologic examination revealed that cysts originated from distended branches of the billiary tree with moderate to heavy inflammatory changes.One patient died during the immediate postoperative period whereas the morbidity was 20%. The mean hospitalization time was 14 days. In conclusion, simple hepatic cysts, represent developmental abnormalities that originate from the billiary tree. They may be quite large in diameter and may cause an acute abdomen because of their rupture. The diagnosis of the disease is rather easy and their surgical management without any particular difficulties.

### P101

THE VALUE OF INTRAOPERATIVE ULTRASONOGRAPHY

I. Kafetsis, H.Zisis, TH. Tsakiris, X. Karabinas, D. Kamilaris, I. Damtsios.
Surgical Unit of Athens Polyclinic. Greece.

Intraoperative ultrasonographic scanning is a reliable method of hepatic exploration. It locates all the important elements of the liver parenchyma more accurately than the hand palpation. The aim of this work is to present the potential value of peroperative ultrasonographic study in the liver surgery. Using this method in abdominal surgery and especially in hepatic and billary tract surgery with the classic technique(5,7.5,10 MHz probe), in many patients and in 45 pts selectively we had the following results: In 6 pts with hepatocellular carcinoma, 10 liver metastases, 10 echinococcus, 8 with cystic disease and 13 biliary tract lithiasis, we had additional informations of the origin , extension of the tumor and relation with hepatic and portal veins. We also examined if there existed other synchronous nodules and if after hepatectomy the margins were without disease. In the choledocholithiasis we had 10 positive answers and 3 false negative. CONCLUSION: The intraoperative ultrasound is a method of big value and gives answers to the following questions: 1. What is the origin of an hepatic tumor? 2. Where is it situated? 3.How can the surgeon reach this area safely?

### P102

LONGTERM FOLLOW-UP OF FOCAL NODULAR HYPERPLASIA IN THE LIVER. JHW de Wilt[1], RA de Man[2], JS Laméris[3], JNM IJzermans[1]. Departments of Surgery[1], Internal Medicine[2] and Radiology[3], University Hospital Dijkzigt, Rotterdam, The Netherlands.

Focal nodular hyperplasia (FNH) is a benign liver tumour. Treatment often is surgically, especially when the tumour is larger than 5-6 cm. Long-term follow-up studies after conservative treatment (stopping oral contraceptives) are rare. To analyse the diagnostic work-up and both treatment modalities, all consecutive patients (27 female/4 male) with a histological proven FNH lesion in the liver diagnosed between 1979 and 1993 were analysed. These patients were invited to visit the outpatient clinic where history, physical examination, ultrasonography (US) and serology (anti-HCV;anti-HBc) were performed. The mean age was 37 years (range 20-61), 45% (n=14) of the patients were asymptomatic on presentation. In these patients FNH was discovered during laparotomy for non-liver related causes. The mean follow-up in all patients was 57 months (range 1-178). Twenty two of the 27 (82%) women used oral contraceptives, all patients were advised to stop this medication. In all patients multiple imaging techniques were used, US and computer tomography could correctly diagnose 40% and 71% of the lesions respectively. Nineteen patients were treated conservatively, no malignant transformation or acute bleeding did occur. FNH lesions decreased or remained identical in size on follow-up US. Twelve patients underwent hepatic resection, one of these patients died post-operatively. No new lesions were identified at follow-up US in patients treated by surgery. No evidence of Hepatitis B or C was found in patients of both groups. In conclusion to diagnose focal nodular hyperplasia, the most efficient approach is ultrasonography followed by histological confirmation in a US guided needle biopsy specimen. Our follow-up study demonstrates that conservative management is the management of choice in FNH. Hepatic resection must only be performed in patients with tumour growth or uncertain histological diagnosis.

### P103

HEPATIC ADENOMA IN THE LIVER. J.H.W. de Wilt[1], R.A. de Man[2], J.S. Lameris[3], J.N.M.IJzermans[1]. Department of Surgery[1], Internal Medicine[2] and Radiology[3], University Hospital Dijkzigt, Rotterdam, The Netherlands.

Regression of hepatic adenoma (HA) after stopping oral contraceptives occurs, however malignant transformation and rupture has been documented. The choice between conservative (i.e. stopping oral contraceptives) and operative treatment is often debated. The aim of this study is to review literature and our personal experience with this presumed benign liver tumour. Between 1979 and 1993, fourteen patients (11 female/3 male) with HA in the liver were treated. Diagnosis was histologically proven with needle biopsy specimen or hepatic resection. Clinical features, imaging studies, laboratory results and treatment modalities were studied. All patients were invited to visit the outpatient clinic were history, physical examination, ultrasonography (US) and serology (anti-HCV/anti-HBc) were performed. On presentation, five (36%) patients were asymptomatic, one patients was in shock due to bleeding of the tumour. Imaging studies and laboratory results were not useful to predict the diagnosis. Ten women (91%) used oral contraceptives for a mean period of 13.2 (2-25) years. Seven patients were treated conservatively with frequent follow-up. In two patients transformation to a hepatocellular carcinoma occured, both men had hepatitis B. Other patients did not show tumor progression on follow-up US. Seven patients underwent hepatic resection without postoperative deaths. One patient died two years after an incomplete resection due to metastasis of a hepatocellular carcinoma. Other patients did not show new lesions at follow-up US. No evidence of hepatitis B or C was found in the patients at follow-up. In conclusion the diagnosis is hepatic adenoma is difficult with imaging techniques, laboratory results and histology, especially in patients with hepatitis B. A solitary hepatic adenoma lesion in the liver should be resected, because malignant transformation and rupture is not uncommon.

### P104

HEMODYNAMIC EFFECTS OF INTERMITTENT PNEUMATIC COMPRESSION OF THE LOWER LIMBS DURING LAPAROSCOPIC CHOLECYSTECTOMY

Y. Christen, M.A. Reymond, J.-J. Vogel, C.E. Klopfenstein, Ph. Morel, H. Bounameaux
University Hospital of Geneva, Switzerland

Background: The effects of pneumoperitoneum during surgical laparoscopy on lower limbs venous hemodynamics have been studied in animal models and in patients. However, the effects of intermittent compression boots are not known in such venous stasis conditions.
Methods: In 12 male volunteers and 12 patients the venous hemodynamic effects of intermittent pneumatic compression boots were studied under external abdominal pneumatic pressure or during laparoscopic cholecystectomy, respectively. Femoral venous diameter and peak femoral venous velocity were measured. Venous pressure was also monitored during the surgical procedure.
Results: External abdominal pressure of 50 mmHg and pneumoperitoneum increases the diameter (17% in volunteers and 14% in the patients) and decreases the blood flow velocity (49% and 32% respectively) in common femoral vein. Femoral pressure was also increased (by 106%) under pneumoperitoneum. In both venous stasis circumstances, intermittent compression of the lower extremities restores venous flow velocity but has no effect on vessel diameter and venous pressure.
Conclusions: The lower limb venous hemodynamic changes were similar during external abdominal pressure or pneumoperitoneum and the flow velocity decrease was intermittently reversed by pneumatic compression boots.

### P105

FREE RADICALS AND ANTIOXIDANTS IN OBSTRUCTIVE JAUNDICE.

A.V.Shaposhnikov,G.A.Vilkov,A.V.Carpov,V.G.R.Nayanar

Rostov Medical University,Rostov-on-Don,Russia.

The 34 patients with benign (24) AND malignant (10) obstructive jaundice were investigated to find out concentrations of free radicals (FR),diene conjugations (DC), antioxidants (A),ammonia (Am) in blood serum and hepatic bile before and at 1,3,5,7,10th days after operations with provisional external drainage of bile ducts.Patients were divided for 3 groups:1 - plasma bilirubin (PB) up to 100mmol/l;2 - 101-180mmol/l;3 - 181-250mmol/l.FR,DC,A WERE measured by chemoluminescence method,Am - by Zelingson-Broun.Levels all of these values in blood plasma were elevated over 2-3 times before and at 3-7th days after operations in comparison with control.Results of these measurement are given below in hepatic bile:

| GROUP | DAYS | FR | A | DC | Am |
|---|---|---|---|---|---|
| Normal Values | | 1015 imp/sec | 0.4* tg $\alpha$ | 1.6 U | 0.3 mg% |
| 1 | 1 | 2217 | 0.8 | 3.5 | o.5 |
| | 3 | 2463 | 0.9 | 4.0 | 0.6 |
| | 10 | 1468 | o.4 | 1.7 | 0.3 |
| 2 | 1 | 2683 | 1.0 | 4.8 | 0.7 |
| | 3 | 2912 | 1.1 | 5.1 | 0.7 |
| | 10 | 2366 | 0.7 | 2.0 | 0.4 |
| 3 | 1 | 2813 | 1.2 | 5.0 | 0.8 |
| | 3 | 3011 | 1.3 | 5.3 | 1.0 |
| | 10 | 2480 | 0.8 | 2.6 | 0.6 |

*Increasing tg $\alpha$ means decreasing A;$P<0.05$ (all cases).

Conclusions: Obstructive jaundice significantly increased the levels of free radicals,diene conjugations,ammonia in blood serum and hepatic bile.Antioxidant activity decreased.These changes strongly depend on the level of plasma bilirubin.In postoperative period the decrease of these toxic products was more intensive in blood serum than in hepatic bile.

### P106

***EXPERIMENTAL ASPECTS IN CREATION OF BILIODIGESTIVE ANASTOMOSIS***

*A. Tomanović, B. Daković, S. Sekulić, P. Bojović, B. Lazić, S. Bacić, N. Mitić.*

*Clinic of Surgery, Faculty of Medicine, University of Priština, 38000 Priština, Yugoslavia*

*The main purpose in a creation of biliodigestive anastomoses (BDA) is, that the anastomosis has to be permanent and with the lumen which is enough wide for a biliodigestive derivation of the gall. To performed this is needful good indication and good surgical procedure. In a prevention of strictures in BDA it is important to have right selection of suture material used in a creation of BDA.*

*In a exact experimental procedure which is performed on 16 dogs we analyst the toleration of tissues to used sutures of different origin in contact with the tissues.*

*In a experiments were used the anastomoses holeciste with a jejunum or duodenum. In a study was investigated the behaviors of suture materials: a) Prolen 000-monofilament, b) Dexon 000, c) Vicryl 000, d) Flax fibre No. 90 and d) Hrom-Catgut 00. The relaparatomia was made 15 and 30 days after beginning of experiments. On the base of results obtaining in our study we could conclude that: 1) the reaction of the tissue around the suture implanted in the duodenum is more reactive than the reaction in tissue of jejunum, 2) the reaction was largely appear in digestive organ while the reactions on holeciste were less or without reaction, 3) the reactions to Monofilament Prolen were always less in BDA, 4) Dexon 000 gave moderate reactions followed by the gigantic cells, 5) Vicryl 000 gave the similar reaction as reaction produced by Dexon 000, 6) Flax fiber No 90 produced large reaction followed by mononuclear and neutrophil cells, and 7) Hromic catgut in its decomposition gave much more moderate infiltrations of mononuclear and gigantic cells in all spaces of BDA.*

### P107

THE EFFECTS OF THE H2-RECEPTOR BLOCKERS ON BILE COMPOSITION IN DOGS

Emin YEĞİNBOY, Atilla ÇÖKMEZ, Rasih YILMAZ

First Department of Surgery,Atatürk State Hosp

Surgery Department,Aegean University Hosp

İZMİR TURKEY

An experimental study was made to investigate the effects of the H2-Receptor Blockers in dogs.

It was performed by cholecystostomy,common bile duct ligation and jejunostomy in seven dogs.All animals received intramuscular 3 mg/kg injections of Ranitidin twice per day from postoperative first day for 10 consecutive days.Daily bile secretion was collected, measured and given back through jejunostomy. In the 5th and 10th postoperative days,total bile acid, cholesterol,phospholipid were measured in 10 ml of bile.The results were compared with control values obtained peroperatively.

From the measurements of bile analysis either in the study group or in the control group bile acid was not significantly different.The cholesterol concentration was significantly increased and phospholipid concentration was significantly decreased.The lithogenic index was not significantly different.

We conclude that ranitidin may alter in gallbladder the bile composition.And also can make bile lithogenic and facilitates the formation of bile stones.

### P108

CHANGES IN SERUM INTERLEUKIN-6 AND C-REACTIVE PROTEIN FOLLOWING LASER OR DIATHERMY LAPAROSCOPIC CHOLECYSTECTOMY

A.C. de Beaux, S.J. Wigmore, K.C.H. Fearon, O.J. Garden
University Department of Surgery, Royal Infirmary, Edinburgh, EH3 9YW

The metabolic response to trauma following laparoscopic cholecystectomy is diminished compared with the open approach. This study was undertaken to examine whether surgical dissection with laser, in comparison with diathermy, influenced the size of the metabolic response to trauma as measured by changes in serum interleukin-6 (IL-6) and C-reactive protein (CRP) concentration. Twelve consecutive patients, with symptomatic cholelithiasis, underwent elective cholecystectomy with pre-operative randomisation to the use of laser (SLT-CL MD, Fuji Electric Co. Ltd. Japan) or monopolar diathermy (Force 4, Valleylab, UK.) as the method of gall bladder dissection. Venous blood samples were collected from each patient pre-operatively and at timed intervals during the 24 hours after the first incision. Serum IL-6 and CRP were measured by enzyme-linked immunosorbent assay. There was no significant difference in the age, sex distribution, mean length of operation or intravenous fluid administration between the two groups of patients. Recovery was uncomplicated with no episodes of pyrexia or obvious sepsis.

Pre-operative serum IL-6 and CRP concentrations were undetectable in all patients. There was no significant difference in the mean post-operative serum IL-6 reponse between the diathermy and laser groups (163.5±18.4 v 168.3±21.5; mean area under the curve±SEM, p = 0.81; Mann-Whitney U test respectively) while the CRP concentration at 24 hours was significantly higher in the diathermy group compared with the laser cholecystectomy group (20.3±1.6 v 11.8±0.7 mg/dl, p = 0.004 respectively). These observations suggest that patients undergoing laparoscopic cholecystectomy with diathermy dissection have a greater activation of the acute phase response as measured by serum CRP, despite having a similar serum IL-6 response, compared with the laser dissection group.

### P109

### HEPATIC MITOCHONDRIAL RESPIRATORY FUNCTION FOLLOWING OBSTRUCTIVE JAUNDICE.

R.N. Younes, M.M. Itinoshe, V.M. Yoshida, M.E. Carvalho, D Birolini. Department of Surgery (LIM-62), and Heart Institute, University of Sao Paulo School of Medicine, Sao Paulo, Brazil

Obstructive Jaundice affects hepatic cell function, with significant effects on organ metabolism. Duration of jaundice has direct implication with detrioration of metablic function. The present study evaluates the effects of bile duct ligation (BDL) of different durations (1 or 7 days) on hepatic mitochondrial respiratory function. Adult Wistar rats (n=32) were randomized into 3 groups: **CTL**(n=12-sham operation), **Jaun1** (n=10-BDL for 1 day), **Jaun7** (n=10, BDL for 7 days). BDL was performed under anesthesia. Livers were excised and mitochondria extracted and studied *in vitro* at S3 and S4 phases of mitochondrial respiration. We also determined interphase rates S3/S4 (ACR), as well as ADP consumption rate per oxygen consumed (ADP/O).

**RESULTS**: (± SD)

| | CTL | Jaun1 | Jaun7 | ANOVA |
|---|---|---|---|---|
| S3 | 179±9.4 | 190±9.2 | 161±10.4 | p=0.126 |
| S4 | 23±1.2 | 28±0.9 | 27±1.4 | p=0.009 |
| ACR | 7.7±0.1 | 6.6±0.1 | 5.8±0.2 | p=0.001 |
| ADP/O | 2.8±0.1 | 3.1±0.2 | 2.2±0.1 | p=0.014 |

Obstructive jaundice affected mitochondrial respiration early in the course of the disease (within 24 hs), with significant decrease of the production of high energy bonds. The early hepatic mitochondrial dysfunction could explain the high incidence of severe metabolic disturbances observed in the jaundiced patients. Revrsal of bile duct obstruction might affect positively the outcome of these patients.

### P110

### EPIDERMAL GROWTH FACTOR (EGF) ENHANCES ENDOCRINE PANCREATIC SECRETION?

F. Marotta, *H. Tajiri, +J. Wu, +D.H. Chui, O. Bellini, G. Barbi.
GI Service, S. Anna Hosp., Como, Italy; *2nd Intern. Med. Dept., Natl. Def. Med. College, Tokorozawa, Japan; Intern. Med. Dept., N. Bethune Univ., Changchun, China;

The aim of the present research was to study the effect of exogenous EGF on endocrine pancreatic secretion. Wistar rats were given a 0.1ml i.p. injection of 10μg/kg EGF or saline t.i.d./week. Insulin, glucagon and somatostatin was determined in the blood and in the pancreatic tissue isolated soon afterwards. Pancreas was homogenized, diluted and centrifuged at 3000 rpm for 20min at 4°C. The supernatant was processed by RIA for insulin, glucagon and somatostatin. Part of the tissue was fixed and cell immunostaining for endocrine component was performed. No difference in body and pancreas weight was observed. As compared to controls, only blood level of somatostatin was significantly higher in rats treated with EGF ($p<0.05$). Glucagon and somatostatin tissue level showed a significant increase ($p<0.05$). Cell counting/$mm^2$ of both A-cell and D-cell was 2- to 5-fold higher in treated rats ($p<0.01$). The present data show that EGF, employed at near to physiological level, enhances pancreatic population of either A- and D-cell thus promoting either tissue and blood levels of these hormones. It could be suggested that EGF indeed exerts a physiological DNA and RNA synthesis effect on endocrine pancreas whose specific receptors have to be identified yet.

### P111

### ERCP-RELATED PANCREATITIS: THE DANGER OF LEAVING AN OBSTRUCTED BILIARY SYSTEM

A.C. de Beaux, K.R. Palmer, D.C. Carter
University Department of Surgery, Royal Infirmary, Edinburgh EH3 9YW

Pancreatits following ERCP is a recognised complication, more common after therapeutic ERCP or pancreatic duct injection. We report 14 cases of ERCP-related pancreatitis observed over a five year period with 4 deaths (29 % mortality). The diagnosis of pancreatitis was based on an elevated serum amylase in the presence of clinical or radiographic evidence. The ERCP was performed under elective conditions for suspected choledocholithiasis, with antibiotic prophylaxis, by experienced endoscopists. Pre-ERCP serum bilirubin was above the upper limit of normal (17 μmol/l) in all cases with a mean of 62 (range 22 - 97 μmol/l). Gallstones were confirmed in 12 patients, with choledocholithiasis at the time of ERCP in 10 patients. These 10 patients underwent endoscopic sphincterotomy and bile duct clearance by basket or ballon catheter methods, with successful duct clearance in 7 patients. In the remaining 3 patients, the stones were considered 'small' by the operator and deemed able to pass through the sphincterotomy. Pancreatic duct injection was inadvertently performed in 6 patients.

Comparing the 4 patients who died with the 10 survivors, there was no significant difference in age (65±7.5 years v 55±3.0, p=0.20 Mann-Whitney U test), sex (p=0.15), pre-ERCP bilirubin (62±13 μmol/l v 61±7, p=0.89), the presence of choledocholithiasis (p=0.15), successful duct clearance (p=0.76) or pancreatogram (p=0.74). However, in the 4 patients who died, the serum bilirubin continued to rise on the first and second day post-ERCP (suggesting an obstructed biliry tree from stone impaction or oedema of the ampulla of Vater) whereas in the 10 patients who survived, the serum bilirubin level fell post-ERCP (p=0.0003). The concomitant presence of ERCP-related pancreatitis with an obstructed biliary tree post-ERCP in this small series was a lethal combination. Monitoring of serum bilirubin in ERCP-related pancreatitis may allow early surgical duct decompression in this high risk group.

### P112

### THE EFFECT OF TRIMETAZIDINE ON THE CERULEIN-INDUCED ACUTE PANCREATITIS IN RATS

Mehmet İŞLER, Ömer ÖZÜTEMIZ, Galip ERSÖZ, Gül YÜCE, Yücel BATUR

*Ege University, Depts.of Gastroenterology and Pathology, İzmir, Turkey*

We aimed to evaluate the effect of trimetazidine (TMZ), which has scavenger activity on free oxygen radicals, on histologic improvement and decline in hyperamylasemia in cerulein-induced pancreatitis in rats. METHODS: Male Wistar rats weighing 240-255 g were used. Group I (n=11): Saline + Placebo, Group II (n=10): TMZ (2.5 mg/kg body weight/day, ip, for 1 week) + Placebo, Group III (n=10): Saline + Cerulein (20 μg/kg body weight, sc hourly, 4 times), Group IV (n=11): TMZ + Cerulein. Twelve hour later of the first cerulein injection blood was drawn via an intracardiac punction, and the animals were sacrificed by cervical dislocation, and pancreas was taken out. RESULTS: Pancreas weight and serum amylase activity in Group III (Saline + Cerulein) were significahtly higher than those in Group I ($p<0.001$), II ($p<0.001$), and IV ($p<0.05$). These parameters were also higher in Group IV than those in Group I ($p<0.05$) and II ($p<0.05$). Oedema and neutrophilic inflammatory response in pancreas were more pronounced in the animals in Group III (Saline + Cerulein) than those in Group IV (TMZ + Cerulein) ($p<0.01$). Malondialdehyde concentration in pancreas was highest in Group III, lowest in Group I and II, and medium in Group IV. CONCLUSION: TMZ pretreatment protects the evolution of cerulein - induced pancreatitis in rats. It decreases pancreas malondialdehyde concentration, suggesting that this preventive effect may result from the elimination of free oxygen radicals.

### P113

### HEPATIC LESION IN ACUTE PANCREATITIS: EXPERIMENTAL STUDY IN RATS

A.M. COELHO, S.N. SAMPIETRE, E.E. ABDO, M. MACHADO.
Department of Surgery - University of São Paulo, Brazil.

The aim of this study is to analyze hepatic lesion following acute pancreatitis (AP) with hepatic mitochondrial function study. We studied 54 Wistar rats divided in six different groups. Acute pancreatitis was produced with injection of 0.5 ml of 5% sodium taurocholate in the bile-pancreatic duct. The hepatic mitochondrial function evaluation was polarographically determined using the Clark's electrode with determination of $O_2$ consumption with ADP (state 3-activated) and in the absence of ADP (state 4), using potassium succinate as substract. Respiratory control ratio (RCR) and ADP/$O_2$ ratio (ADPR) were calculated.

There were significant alterations in RCR, state 3 and 4 of mitochondrial respiration and alterations in the ADPR 2 and 4 hours after the induction of acute pancreatitis.

This data show that in the early phase of AP (2 and 4 h), where the hepatic lesions seems to be dependent on depressive action of toxic substances released during AP, there is mitochondrial uncoupling manifested by increasing of S4 and decreasing of RCR and ADPR. Twelve and 24 hours after AP, RCR is the same as of the control group. 48 hours after AP, we observed decrease of RCR, S3 and ADPR, suggesting degenerating and necrotic process, characteristic of cellular ischemia.

We conclude that the mitochondrial alterations are bifasic: early alterations characterized by uncoupling of oxydative phosphorilation, may be the result of distant action of enzymatic products while late alterations seems to be the result of tissue ischemia.

### P114

### CYTOKINE GENE EXPRESSION IN FOETAL PANCREATIC ISLET ISOGRAFTS IN NON-OBESE DIABETIC (NOD/Lt) MICE

J. Kovarik, BE Faulkner-Jones, M. Koulmanda, TE Mandel
*The Walter and Eliza Hall Institute of Medical Research, 3050 Victoria, Australia*

The aim of this study was to analyse cytokine gene expression in islet isografts undergoing autoimmune β-cell destruction accelerated by cyclophosphamide (CP) injection. Foetal pancreatic islet tissue after 1 week of culture was transplanted under the left kidney capsule of prediabetic, 67-69 days-old NOD/Lt female mice, one week later mice were injected with CP 300mg/kg or saline (controls). Blood glucose was monitored starting at the day of CP injection (day 0), at day 7 and day 17. Seven and 13 days post CP injection left kidneys were removed by nephrectomy and the grafts processed for RNA isolation, reverse transcription and PCR amplification of cytokines. At day 17 all animals were sacrificed and their pancreas examined histologically for infiltration.

Non of the 4 control mice became diabetic whereas 3/12 CP injected mice had blood glucose levels >17mmol/L at day 17. Pancreas sections of CP injected mice showed a severe lymphocytic infiltrate leading to complete (diabetic mice) or partial (non diabetic mice) islet destruction whereas control mice had mostly either intact islets or only mild insulitis. Three grafts from each group (controls, CP injected non diabetic and CP injected diabetic mice) were selected to analyse cytokine gene expression at day 7 and day 13 post CP injection. IL-12p40 and INF-γ were both expressed generally in all tested isografts as well as in ungrafted kidney tissue, in contrast TNF-α and β were expressed in grafted tissue only at both time points. IL-2 and IL-12p35 were expressed in 3/3 day 7 isografts but only in 1/6 day 13 isograft. In this same graft (CP injected animal) IL-6 expression was detectable whereas all other grafts were negative for IL-6 message. IL-10 expression was completely absent in all tested grafts and control tissue.

The pattern of cytokine gene expression of immune cells infiltrating the foetal pancreatic NOD/Lt isografts after CP injection does not predict the risk of progression to diabetes for each individual mouse, at least with the tested cytokines.

### P115

### DOES INTERCELLULAR ADHESION MOLECULE-1 EXPRESSION IN STORED HUMAN LIVER ALLOGRAFTS AND FOLLOWING REPERFUSION CORRELATE WITH EARLY POST-OPERATIVE OUTCOME ?

**A. Bhargava[1], N. J. Bradley[1], A. K. Burroughs[2], A. P. Dhillon[3] K. Rolles[1], B. R. Davidson[1].**

**[1]University Department of Surgery, [2]LiverTransplantation Unit and [3]Department of Histopathology**

**Royal Free Hospital School of Medicine, Pond Street, London NW3 2QG UK.**

Intercellular Adhesion Molecule-1 (ICAM-1) is a cytokine inducible endothelial antigen. Graft preservation induced injury is associated with higher rates of acute cellular rejection (ACR).

The aim of this study was to elucidate the distribution of ICAM-1 on liver allografts after overnight cold storage and reperfusion: correlating expression with post-operative outcome.

Following cold storage (723 ± 31 mins) and reperfusion (at 90 mins), liver biopsies from 30 grafts were snap-frozen. 5μm frozen sections were stained immunohistochemically for ICAM-1. Expression of ICAM-1 was analysed by light microscopy. Liver from resection margins of benign tumours were used as controls: demonstrating weak sinusoidal staining.

Twenty-one of the 30 grafts, biopsied after storage, had induction of ICAM-1 on sinusoidal endothelium and hepatocytes. Of these, 14(66.6%), recipients had 3 or more rejection episodes (no non-rejecters). In 9/30 recipients with no ICAM-1 induction, 6 had one episode of ACR (3 non-rejecters).The difference between these two groups was statistically significant ($p<0.001$, Fisher's Exact test). The expression of ICAM-1 on reperfusion biopsies showed further increase in staining intensity on hepatocytes and sinusoidal endothelium. Further material is being collected currently, to evaluate larger numbers of biopsies.

Cytokine activation of ICAM-1 occurs during graft storage and is further increased after reperfusion. Induction of ICAM-1 on sinusoidal endothelium is likely to contribute to increased adhesiveness of circulating leukocytes. ICAM-1 induction may well enhance the immunogenicity of the graft. Our results suggest that induction of ICAM-1 following graft storage, contributes to increasing risk of acute cellular rejection post-transplantation.

### P116

### BACTERIAL TRANSLOCATION IN ACUTE PANCREATITIS: EXPERIMENTAL STUDY IN RATS

L.J. SOUZA, S.N. SAMPIETRE, M. MACHADO, H.W. PINOTTI.
Department of Surgery - University of São Paulo, Brazil.

Infection of necrotic tissue and abscess formation are the most serious complications in acute pancreatitis, responsible for 80% of the mortality associated to acute pancreatitis. The frequent finding of negative gram bacteria in this tissue is suggestive that intestinal tract is involved as a source of this infection.

We studied 90 Wistar rats divided in eight different groups. Acute pancreatitis was produced with injection of 2.5% taurocholic acid in the bile-pancreatic duct (0.1 ml/100g rat weight). Bacterial culture of blood, pancreas, mesenteric lymphonodes, peritoneal cavity and cecum were performed within 6h, 24h, 48h and 96 hours after induction of acute pancreatitis.

Bacterial growing was present in mesenteric lymphonodes in 40% (6h), 90% (24h), 70% (48h) and 40% (96h). In the control groups these results were 10% (6h), 0% (24h), 10% (48h) and 10% (96h). The main bacterial types isolated in the culture were: *E. coli, Streptococcus, E. aerogenes, P. aeruginosa, S. faecalis, S. epidermidis.*

We conclude that bacterial translocation is an early phenomena, already present six hours after acute pancreatitis with maximum at 24 hours, decreasing after that time.

### P117

SEVERE ACUTE PANCREATITIS IS ASSOCIATED WITH ELEVATED SERUM SOLUBLE TUMOUR NECROSIS FACTOR RECEPTOR CONCENTRATIONS

A.C. de Beaux, A.C. Goldie, J.A. Ross, K.C.H. Fearon, D.C. Carter
University Department of Surgery, Royal Infirmary, Edinburgh, Scotland. EH3 9YW

Excessive production of tumour necrosis factor (TNF) is believed to be responsible for many of the features of septic shock, but its role in the pathophysiology of the systemic sequelae in acute pancreatitis remains controversial as TNF is often undetectable in the serum of patients, even with severe disease. Cells expressing surface TNF receptors down-regulate their responsiveness following exposure to TNF by receptor shedding. These soluble receptors ($sTNFR_{55}$ and $sTNFR_{75}$) bind TNF in the circulation thereby reducing its bioavailability. This study assessed the serum concentration of TNF and sTNFR in 58 patients with acute pancreatitis on the first day of admission. Thirty patients had mild disease, 28 had severe disease of whom 18 patients developed local pancreatic complications alone (Atlanta classification) and 10 patients developed organ failure (Goris score). TNF was only detected in 18 patients, 1 with mild disease, 10 with local complications only and 7 with organ failure (minimum detection level; 15 pg/ml). sTNFR was detectable in all patients. The results, given in the table are expressed as the median (interquartile range) in pg/ml.

| Severity | TNF | $sTNFR_{55}$ | $sTNFR_{75}$ |
|---|---|---|---|
| mild | 15<br>(15-15) | 1058<br>(652-1388) | 1312<br>(963-1927) |
| local complication | 17.8<br>(15-23.8) | 2125<br>(1751-2717) | 2687<br>(1760-3239) |
| organ failure | 22.6<br>(15-75.1) | 4625<br>(3615-5307) | 4916<br>(2704-11363) |
| p value Kruskal-Wallis | 0.34 | 0.001 | 0.001 |

Organ failure in patients with acute pancreatitis is associated with significantly increased levels of both $sTNFR_{55}$ and $sTNFR_{75}$ but not TNF. These observations would support the central role of TNF in mediating inflammatory events early in the course of the disease.

### P118

EXOGENOUS PUTRESCINE ADMINISTRATION AMELIORATES THE SUPPRESSED REGENERATIVE CAPACITY OF CADMIUM-PRETREATED RAT HEPATOCYTES

A.Margeli, S.Theocharis, S.Skaltsas, C.Spiliopoulou, An.Koutselinis
Department of Forensic Medicine and Toxicology, School of Medicine, University of Athens, Athens, GR 11527, Greece

Cadmium (Cd) is a rare element that is nevertheless widely distributed throughout the biosphere and its toxic effects are becoming potentially more serious, due to industrialization. Liver regeneration can be considered as a spectacular example of controlled tissue increase. The purpose of this study was to document liver regeneration after partial hepatectomy (PH) in a model of acute liver injury due to Cd treatment and to determine whether the administration of exogenous putrescine affects the regenerative capacity of hepatocytes. Putrescine is a polyamine that has been reported to stimulate liver regeneration in animal models of acute liver failure.Cd pretreatment, 24 hours prior to PH, resulted in decreased regenerative capacity of hepatocytes compared to that observed in simply partially hepatectomized rats ($p<0.001$). Tritium thymidine incorporation into liver DNA, thymidine kinase activity into the hepatic tissue and mitotic index were used as indices of liver regeneration. The intraperitoneal administration of putrescine, at doses of 1 and 10 mg/Kg body weight, at the time of surgery and at 4 and 8 hours after PH in Cd-pretreated rats, partly restored the liver regenerative capacity ($p<0.001$). The results of this study indicate that hepatic DNA synthesis is impaired in Cd-pretreated rats after PH and that exogenous putrescine administration enhanced liver regeneration in this model of acute liver disease.

### P119

FUNCTIONAL PARAMETERS OF ISOLATED LIVER IN AN EXTRACORPOREAL LIVER ASSIST CIRCUIT

G. Kostopanagiotou, N. Arkadopoulos, V. Kontopoulos, H. Manolis, C. Hondros, M. Demonakos, J. Papadimitriou
Second Dept. of Surgery, Dept. of Anesthesiology and Lab. of Biochemistry, University of Athens, Areteion Hospital, Athens, Greece

The aim of this experimental protocol was to evaluate the morphological and functional characteristics of isolated pig livers, perfused in an extracorporeal liver assist circuit. The circuit has been developed in our department and consisted of the graft liver,a membrane oxygenator, a heater, a centrifuge pump and a fluid reservoir. Twelve pig livers, weighing 780 (610-870) gms, were perfused for a mean of 5.2 (4.5-9) hours. Perfusion was terminated when morphological and functional signs of decreased viability were present. Inflow to the graft liver was performed at a pressure of 16 (12-20) mmHg at 38°C through the portal vein and outflow was secured through the suprahepatic inferior vena cava. Perfusion solution consisted of R/L and 2% bovine albumin. During perfusion the following parameters were evaluated through pre- and post-hepatic sampling: oxygen tension, pH, $HCO_3^-$, BE, $[Na^+]$, $[K^+]$, $[Ca^{++}]$, osmolarity, glucose, lactates, AST, ALT, ALP, γGT, bilirubin and coagulation factors I, V and VII. All samples were collected at time 0 (end of priming and connection to the graft liver) and +3, +15 and +30 minutes after starting of perfusion and hourly afterwards. Biopsies were obtained periodically. Mean values were as follows:

| Time | 0 | +1 h | +2 h | +4 h | +6 h | +8 h |
|---|---|---|---|---|---|---|
| $[K^+]/[Na^+]$ (mmol/l) | 3.93/125 | 4.31/129 | 4.66/130 | 6.09/130 | >8/133 | >8/144 |
| $[Ca^{++}]$/[lactate](mmol/l) | 2.13/14.4 | 1.87/55.6 | 1.79/45.15 | 1.44/28.6 | <1/14.06 | <1/0.27 |
| AST/ALT (IU/l) | 0/0 | 795.5/33.7 | 1349/42.1 | 1685/54.3 | >2000/207 | >2000/1058 |
| pH/OSM (mOsm) | 7.45/245 | 7.34/263 | 7.50/267 | 7.39/270 | 7.31/276 | 7168/290 |
| $PO_2$ input/output (mmHg) | 527 | 643/320.7 | 617.5/2092 | 578/311.7 | 581/218.1 | 372.2/10 |

Results show that the viability of the liver graft decreased after a five-hour perfusion course in an extracorporeal assist circuit. Correction of pH contributed to an increase in bile flow. Technical problems immediately aggravated liver graft morphology and function. While its oxygen consumption remained unaffected until the late stages.

### P120

SUPPRESSION OF TUMOR METABOLISM BY NORCANTHARIDIN IN MORRIS HEPATOMA BEARING RATS.

P.Mack,* X.F.Ha,* L.Y.Cheng.#
*Department of Surgery, Singapore General Hospital & #Department of Biochemistry, National University of Singapore, Republic of Singapore.

Norcantharidin is the demethylated form of Cantharidin, which is the active ingredient of the blister beetle, Mylabris, a long used traditional medicine in China. It has anti-cancer effects especially towards hepatoma, and is relatively free from side effects. This study was conducted on a rat Morris Hepatoma model to compare the efficacy of Norcantharidin treatment against that of Adriamycin treatment and hepatic artery ligation. The degrees of tumor metabolism suppression achievable within 1 hour with the various types of treatment were quantified using $^{14}C$-glucose oxidation measurements on liver and tumor tissues harvested from the treated animals. Results of the tissue glucose oxidation were expressed as a tumor/liver ratio. The tumor/liver ratio was $4.2\pm2.2$ in untreated tumor-bearing controls (n=9), but this ratio dropped to $2.3\pm0.5$ ($p<0.05$) with intra-arterial Norcantharidin at 0.5 mg/kg (n=9), to $2.3\pm0.7$ ($p<0.05$) with intra-arterial Adriamycin at 2.4mg/kg (n=8), and to $2.2\pm0.7$ ($p<0.05$) with hepatic artery ligation (n=8). With intravenous Adriamycin at 2.4 mg/kg (n=7), the ratio became only $3.5\pm2.0$ and was not significantly lower. The Analysis of variance and Duncan's multiple range tests were used for statistical analysis. It is concluded that intra-arterial Norcantharidin is as effective as intra-arterial Adriamycin and hepatic artery ligation in suppressing tumor oxidative glucose metabolism in this animal hepatoma model.

### P121

### EFFECT OF ENDOTOXIN (LPS) AND LACTOBACILLUS R2LC (LB) ON MACROPHAGE BEHAVIOR IN ACUTE LIVER INJURY

F.B. Kasravi, D. Gebreselassie, D. Adawi, B. Jeppsson, S. Bengmark
Dept. of Surgery, Lund Univ., Lund, Sweden

Phagocytic index, $O^2$ free radical (OFR) production, and metabolic response of the rat's peritoneal macrophage were evaluated in D-galactosamine liver injury after LPS and LB pretreatment. D-galactosamine increased the OFR response in the luminometer, which was unaffected by LPS, but highly potentiated by LB pretreatment. Metabolic response in the calorimeter was also increased after D-galactosamine administration and was unaffected by LB, but absent in the LPS pretreatment. Phagocytic response was lower than normal in all experimental groups and was unaffected by any pretreatment.

### P122

### IN VITRO COLON CARCINOMA CELL GROWTH IS STIMULATED BOTH BY PORTAL SERUM AFTER PARTIAL HEPATECTOMY AND HEPATOCYTES

K.P. de Jong[1], M.A.M Brouwers[1], M.L. van Veen[2], T. Daemen[2], M.J.H. Slooff[1].
Department of Surgery (Division HB Surgery and liver transplantation[1]) and Department of Physiological Chemistry[2]. University Hospital and State University Groningen, The Netherlands.

In rats increased tumor growth in the remnant liver after partial hepatectomy (PH) was found. Growth factors responsible for liver regeneration could also influence tumor growth in the remnant liver. In liver regeneration both endocrine and paracrine mechanisms play a role. In this study we analyzed the effect of portal and systemic serum obtained from PH or sham operated rats on proliferation of colon carcinoma cells (CC 531) in vitro. The effect of adding hepatocytes to these cultures was also studied. Cell proliferation was measured by $^3$H-thymidine ($^3$H-thy) incorporation. Sera were withdrawn at intervals of 1,3 and 14 days after 70% PH or sham operation. Cultures of CC 531 cells in the presence of 5, 10, 20 or 50 % serum were harvested after 48 hours and incorporation of $^3$H-thy was measured using liquid scintillation counting. Cultures with portal serum obtained at days 1 and 14 after PH or sham operation did not show a difference between PH and sham serum. Portal serum obtained 3 days after PH resulted in a 25 to 40% increase of $^3$H-thy incorporation in CC 531 cells as compared to sham operated portal serum ($p<0.01$ ANOVA). Cocultures of hepatocytes and CC 531 cells in the presence of portal serum (either PH or sham) showed an increased $^3$H-thy incorporation in CC 531 cells compared to CC 531 cells cultured separately. This synergistic effect was more pronounced if the cells were plated at a low cell density. Ratios of (CC 531:hepatocytes) of 1:10 and 1:1 showed a more pronounced effect than at a ratio of 10:1. Using the same cocultures, incorporation of BrdU was observed in CC 531 cells in varying amounts but very rarely in hepatocytes.

Conclusion: Changes in PH portal serum are responsible for a direct stimulating effect on proliferation of CC 531 cells in vitro. These changes were found only in serum obtained at day 3 after PH and not at days 1 and 14. Apart from these endocrine effects on cultured colon carcinoma cells a direct paracrine effect of hepatocytes on CC 531 cells was found. This effect was found irrespective of the presence of portal serum obtained from PH or sham operated rats.

### P123

### EFFECTS OF LIVER RESECTION AND TRANSPLANTATION ON LIPID PARAMETERS - A LONGITUDINAL STUDY

K Hardy, *A Akdeniz and *ME Cooper Departments of Surgery and *Medicine, Austin/Heidelberg Repatriation Hospitals, Melbourne, Victoria, Australia

The liver plays a vital role in the production and clearance of a large number of lipoproteins and is an important determinant of the plasma levels of various lipids including cholesterol as well as apoproteins such as apoprotein (a). To explore the role of the liver in the regulation of lipid and apoprotein levels, a serial prospective study over 6 months was performed measuring fasting total cholesterol [TC], triglycerides, HDL-cholesterol [HDL-C] and apoprotein (a) in individuals undergoing hepatic resection for isolated hepatic metastases secondary to colo-rectal malignancy [n=8, aged 52±4yrs] or transplantation for end stage liver disease [n=11]. Controls, who were individuals undergoing colorectal surgery for malignancy, were studied in an identical manner [n=9, aged 57±5yrs]. In addition, a blood sample was taken from the donor for liver transplantation immediately before removal of the liver.

In the group with hepatic resection, baseline total and HDL-cholesterol were normal (5.0±0.5 and 1.2±0.1mM respectively). Over the next few days there was a rapid decrease in total (day 3, 2.9±0.3) and HDL-cholesterol (day 3, 0.79±0.12mM). However, these changes could be explained by fasting and surgical intervention since a similar phenomenon was observed in the control subjects (TC, pre-op, 5.3±0.3 vs day 3, 3.2±0.3mM; HDL-C, pre-op, 1.22±0.12 vs day 3, 0.81±0.08mM). In patients undergoing liver transplantation, TC decreased over the next few days but had fully recovered by day 40 (pre-op, 3.8±0.5, day 1, 2.2±0.2, day 40, 5.2±0.3mM). Triglycerides were low pre-operatively and rose over weeks to months (data not shown). HDL-C was very low pre-operatively (0.47±0.12mM), dropped further in the early post-operative period (nadir, day 3, 0.16±0.05mM) and had returned towards but had not reached the normal range by day 40 (0.9±0.1mM). Apoprotein (a) was low pre-operatively (30x/÷1.4IU/l, geometric mean x/÷ tolerance factor), remained low over the first week (day 3, 24x/÷1.8IU/l) but had risen by day 10 (day 40, 64x/+1.5IU/l). Importantly, apoprotein (a) at day 40 correlated with the apo(a) level of the donor ($r=0.80, p<0.01$) but not of the recipient's pre-operative level ($r=0.19, p=0.57$).

In conclusion, the liver has a large reserve and is able to maintain lipoprotein production and removal despite greater than 50% removal. The major cause of reduced lipid levels in the post-operative period relates to other factors such as fasting and handling of the gut during surgery. In liver transplantation, apoprotein (a) levels resemble those of the donor within 2 weeks of organ donation, consistent with the liver being the major site of production of this apoprotein.

### P124

### VASCULAR RESPONSES OF THE ISOLATED DUAL-PERFUSED RAT LIVER TO HEPATIC ARTERIAL AND PORTAL VENOUS INJECTIONS OF NORADRENALINE

W Yang, IS Benjamin, B Alexander
Department of Surgery, The Rayne Institute, King's College School of Medicine & Dentistry, London, United Kingdom.

A reliable and reproducible model for the study of hepatic arterial vascular changes *in vitro* during pathological conditions of the liver has yet to be described. This study was conducted to determine whether it was possible to develop an *in vitro* isolated rat liver preparation perfused through the portal vein (PV) and the common hepatic artery (HA) initially under control conditions.

6 male Sprague Dawley rats (258.3 ± 8.35g) were anaesthetised with sodium pentobarbitone (3mg $100g^{-1}$) and a midline incision made to expose the viscera. The bile duct, the common hepatic artery and the portal vein were cannulated under an operating microscope. The livers (11.4 ± 0.4g) were then connected to our perfusion circuit (Alexander et al 1993) and perfused at 0.32 ± 0.01 and 0.98 ± 0.03 ml $min^{-1}$ g $liver^{-1}$ through the HA and PV respectively. Dose-related responses to HA and PV injections of noradrenaline (NA $10^{-5}$ - $5x10^{-3}$M) were measured as transient increases in perfusion pressure. Maximal increases in perfusion pressure of 57.5 ± 7.7 and 4.3 ± 0.5 mmHg were measured in the HA and PV respectively to HA injections of up to $10^{-3}$M NA. Maximal changes in HA and PV perfusion pressure of 29.3 ± 5.1 and 5.32 ± 0.7 were measured to PV injections of up to $5x10^{-3}$M NA. It is concluded that this is a stable and responsive model suitable for investigation of changes in vascular pharmacology in the normal and diseased liver.

Alexander B., Aslam M., Benjamin IS. J Physiol 467: 231P.

### P125

MORTALITY AND CYTOKINE CONCENTRATIONS FOLLOWING INTERMITTENT AND CONTINUOUS HEPATIC ISCHAEMIA

GR Hewitt MI Halliday MD McCaigue BJ Rowlands T Diamond
Department of Surgery,The Queen's University, Belfast, N. Ireland

INTRODUCTION: Hepatic pedicle occlusion reduces blood loss during liver surgery. Little is known about the possible benefits of intermittent occlusion. This study compared systemic tumour necrosis factor (TNF) and interleukin 6 (IL6) concentrations and mortality following continuous and intermittent hepatic ischaemia.
METHODS: Two groups of male Sprague-Dawley rats (300-400g) were subjected to left hemi-hepatic ischaemia for a total ischaemic period of 120 min. Group 1 underwent continuous ischaemia (n=20). Group 2 underwent intermittent ischaemia (clamp released for 5 min every 30 min) (n=20). Mortality was assessed at Day 7. Further groups of rats underwent continuous or intermittent ischaemia (120 min) following which systemic blood was sampled at 0 min, 1 hr, 3 hr and 5 hr for measurement of IL6 (bioassay) and TNF (ELISA). Sham animals underwent laparotomy and mobilisation of left hepatic vessels (n=5 all groups).
RESULTS:

| Reperf(hr) | | Sham | 0 | 1 | 3 | 5 |
|---|---|---|---|---|---|---|
| IL6 | C | 0 (0) | 566 (160)* | 1880 (711) | 7980 (5795) | 9055 (7873) |
| (pg/ml) | I | 0 (0) | 1284 (1073) | 1779 (1320) | 2514 (640)* | 2912 (716)* |
| TNF | C | 0 (0) | 0 (0)* | 232 (198) | 324 (111) | 383 (212) |
| (pg/ml) | I | 0 (0) | 120 (82) | 149 (27) | 144 (64)** | 171 (73)** |

Results expressed as median (inter-quartile range). C = continuous, I = intermittent ischaemia.
* = $p<0.01$ and ** = $p<0.05$ at same reperfusion interval (Mann-Whitney, U test).
Mortality following continuous occlusion was 15/20 (75%) and following intermittent was 4/20 (20%) (p = 0.0015, Chi squared test).
CONCLUSIONS: Intermittent hepatic ischaemia was associated with significantly lower systemic IL6 and TNF concentrations and significantly reduced mortality compared to continuous ischaemia.

### P126

**PPRODUCTION OF HEPATOCYTE GROWTH FACTOR/SCATTER FACTOR FROM FIBROBLASTS IS INHIBITED BY GAMMA LINOLENIC ACID**

WG Jiang, MB Hallett, C Scott, DF Horrobin, MCA Puntis
University Department of Surgery, University of Wales College of Medicine, Cardiff, and Scotia Pharmaceuticals Ltd, Guildford, England, UK

Hepatocyte growth factor/Scatter factor (HGF/SF) is a tumour cell motility and invasion promoter, which is mainly produced by fibroblasts,. This study was to determine the effects of gamma linolenic acid, an agent used in anti-cancer treatment, on the production of HGF/SF by fibroblasts.
Human fibroblast cell line, MRC5 was used. The cells were cultured in the presence or absence of fatty acid (FA) at a range of concentrations (1-100μM) for 24 hours and HGF/SF production was quantified by the MDCK bioassay. In this study, gamma linolenic acid (GLA), its water soluble lithium salt (LiGLA), linoleic acid (LA), arachidonic acid(AA), and eicosapentaenoic acid (EPA) were used. HGF quantity is shown in the following table as units per mililiter conditioned medium and comparison made against control level of 88.0±2.1U/ml (significant level taken at $p<0.05$).

| | FA(6.2μM) | FA(25μM) | FA(100μM) |
|---|---|---|---|
| GLA | 64±3.2 | 32±2.3* | 16±4.0* |
| LiGLA | 64±1.6 | 48±6.0 | 16±3.2* |
| LA | 96±6.3 | 64±5.1 | 64±2.5 |
| AA | 80±5.6 | 64±4 | 48±5.3 |
| EPA | 64±2.3 | 48±6 | 16±2.5* |

GLA, LiGLA, and EPA showed a concentration dependent inhibition of HGF/SF production without causing cytoxicity (determined by MTT assay). Linoleic acid and arachidonic acid had no effects.

We conclude that the parent form of n-6 EFAs gamma linolenic acid can inhibit the production of HGF/SF from human fibroblasts and this may have important implication in the mechanism controlling the initiation and growth of liver metastasis.

### P127

**IMPAIRMENT OF EPIDERMAL GROWTH FACTOR (EGF) RECEPTORS IN EXPERIMENTAL LIVER CIRRHOSIS.**

F. Marotta, * J. Wu, * D.H. Chui, + P. Safran, L. Boncinelli, A. Colombo, G. Barbi. Intern. GI Service, S. Anna Hosp., Como, Italy; * Med. Dept., N. Bethune Univ., Changchun, China; + Econum Lab., Villeneuve d'Ascq, France.

The aim of this study was to provide a time-course mapping of EGF receptor features in experimental liver cirrhosis (LC). Wistar rats underwent LC by inhaling twice a week $CCL_4$-saturated air (5 l/min flow rate) while drinking water being added with 0.5 g/l phenobarbital. Controls were given only phenobarbital-enriched water. Rats were sacrificed at weekly intervals and the liver microsome fraction was obtained. EGF binding assay was performed by using iodinated recombinant human EGF (0.1ml:$5x10^{-11}$-$1.6x10^{-9}$ M) on 0.1ml of 200μg liver membrane. Western blot analysis of EGF receptors was done as well. Scatchard plot of $^{125}I$ EGF showed a linear relationship. EGF maximum binding capacity showed a significant time-course decrease. However, total concentration of immunoreactive EGF receptors did not change and no difference was observed in the dissociation constant. These data suggest the presence of a single class of liver EGF receptors with similar affinity. During the development of LC a significant drop of EGF receptors binding capacity occurs without any relevant change in binding affinity. Thus, such impairment it is likely to be accounted for by an intrinsic receptor abnormality or endogenous substrate occupancy.

### P128

LIMITS OF VASCULAR OCCLUSION IN HEPATIC RESECTIONS IN CIRRHOSIS. EXPERIMENTAL STUDY.

J.Figueras, L.Farran, F.G.Borobia, R.Fradera, J.Castellvi, C. Lama, Y.Ribas, H. Aranda, E.Jaurrieta.
Department Surgery. Hospital Bellvitge. University Barcelona. Spain.

Hepatic resections with vascular occlusion are used with increased frequency in the treatment of hepatocarcinoma. The aim of this study is to evaluate the limits of normothermic liver ischaemia in different degrees of liver function in the rat. Hepatic cirrhosis was induced in male Wistar rats, weighing 120-140 g, using Carbon tetraclorure in water. Hepatic function was graded determining ATIII, albumin, bilirubin in plasma and the presence of ascites. Rats were divide in four different groups, using the modified Child-Pough score: Group Control (non cirrhotic), Group A well compensated cirrhosis, Group B decompensated cirrhosis, Group C decompensated cirrhosis with ascites. All groups were different between them $p<0.05$. Liver ischaemia was performed using the model of ASAKAWA for periods of 0, 30, 45, 60 and 75 minutes. At the end of procedure the non ischaemic lobes were resected. Survival for the different times of ischaemia is shown in the table

| | 0 min | 30 min | 45 min | 60 min | 75 min |
|---|---|---|---|---|---|
| Ctrl n=23 | | | 7/7 100 | 7/7 100 | 4/9 44 |
| A n=14 | | | 7/7 100 | 1/7 14 | |
| B n=21 | 7/7 100 | 6/7 86 | 1/7 14 | | |
| C n=12 | 0/5 0 | 1/7 14 | | | |

Conclusions: The ischaemia time tolerated for cirrhotic livers is shorter than in normal rats. The limits of hilar vascular occlusion depends on the degree of hepatic failure. Decompensated cirrhosis with ascites cannot tolerate any surgical procedure in the liver.

### P129

THE RAT'S LIVER MICROSOMAL ENZYME OXIDATION SYSTEM UNDER THE INFLUENCE OF OMEPRASOLE

Gligorijević,J.,Katić,V.,Tasić,D.and Ilić,R.

Institute for Pathology,Medical Faculty on Niš, Yugoslavia

Several agents known as gastric acid inhibitors have been introduced in clinical use,more or less recently.Among them,the omeprasole has a very strong acid suppresing effect, acting directly on the proton pump located in the secretory membrane of the parietal cells.The investigations of their reactions with the process of drug elimination by interfering with hepatic microsomal oxidation system, gave us a lot of statements about $H_2$-receptor blockers, but a few about the omeprasole. The purpouse of this study was to establishe the effects of the antisecretory dose of omeprasole on the activity of the enzyms in the microsomal oxidasing system 40 male Wistar rats (200±20G) were treated by 40μMol/kg/tt by intragastric instilation up to 56th day from the begining of the experiment.There was control group of animals (5) and three experimental groups (12 rats in each) formed acording to the duration of the treatment. The sacrificing of the animals was done on 10th,30 and 56th day of the experiment.The semples of the liver tissue were immediately prepared for enzymehistochemical detection of the activity of NADPH cytochrome P-450 reductase and cytochrome P-450. Our results show decreased activity of the enzymes tested, which was time dependent and was most expressed in the third group.Significant differences in the enzyme activities among the animals of the same experimental group was atributed to the existence of multiple izoensimes of citochrome P-450, that may be differently affected by omeprasole.

### P130

ORAL ARGININE SUPPLEMENTATION IN ACUTE LIVER INJURY

D. Adawi, F.B. Kasrawi, G. Molin, B. Jeppsson
Department of Surgery and Department of Food Technology, Lund University, Sweden

Nitric oxide has many biological roles. It will be formed from the amino acid L-arginine. Studies suggest that nitric oxide has a protective effect on the liver during endotoxemia and chronic inflammation. The mechanism of this action is not clear. We therefore studied the effect of oral arginine supplementation on the extent of liver injury and the associated bacterial translocation in an acute liver injury model.

Sprague-Dawley rats were used. 2 % arginine has been supplemented daily by a nasogastric tube for 8 days in the experimental group. Acute liver injury was induced on the 8th day by intraperitoneal injection of D-galactosamine (1.1 gm/kg body wt.) In the control group of acute liver injury, saline was given by the nasogastric tube during the same period. Blood samples were collected 24 h after induction of the liver injury. Levels of Alkaline Phosphatase (ALP), bilirubin (bil) and Aspartate Aminotransferase (ASAT) was significantly reduced by arginine supplementation compared to the acute liver injury control group. (ALP 11.58+1.15 vs 16.12+1.82 $p<0.05$; bil 7.31+0.64 vs 14.66+2.48 $p<0.01$; ASAT 20.96+4.48 vs 33.66+5.0 $p<0.05$). Arginine supplementation also reduced bacterial translocation to arterial blood, liver and mesenteric lymph nodes with a significant differance in the liver (447.5+226.2 CFU/gm vs 5112.9+1766 CFU/gm $p<0.05$) On histological examination the liver in the arginine supplemented group exhibited scattered areas of hepatocellular necrosis and inflammatory cell infiltration compared to the control acute liver injury group which showed more and widespread hepatocellular necrosis and more inflammatory cell infiltration.

The results of this experimental study show that oral arginine supplementation significantly improves the level of liver injury and bacterial translocation after galactosamine induced liver injury.

### P131

HLA COMPATIBILITY, VIRAL INFECTION AND ACUTE REJECTION IN LIVER TRANSPLANT.

J. Ontañón, M. Muro, A. Minguela, P. Ramírez, J. A. Pons*, P. Parrilla** A. M. García-Alonso and M. R. Álvarez.
(Sección de Inmunología, *Servicio de Medicina Digestiva, **Servicio de Cirugía General, Hospital Universitario Virgen de la Arrixaca de Murcia, Spain)

The HLA and liver transpalnt (OLT) relationship is still unclear in contrast to other solid organs such as kidney and heart allograft where the role of HLA complex has been widely studied. It is possible that the low antigenity of the liver and its inability to activate naive allogenic T-cells could explain the lack of a clear association between HLA compatibility and rejection.

In this work, we review a total of 118 OLT performed from October 1989 to December 1993 in the Hospital *Virgen de la Arrixaca* in Murcia, (Spain) and we selected a series of 81 OLT, in which the HLA A, B and DR match between receptor-donor pairs and the cause of transplant were known in all cases. On the other hand, the incidence of viral infections such as CMV, HCV and HBV (including fulminant hepatitis) was also studied in each patients. The graft in which the HLA was unknown (n=20) or when the transplant was performed in the AB0 incompatibility (n=1), dead (n=9) or retransplanted (n=3) in the first 3 days postransplant, were excluded.

We observed that the acute rejection rate was not influenced by the differences in Class I or Class II compatibility (0 vs. 1 or more matches). However, the presence of viral infection correlated with acute rejection ($p<0.05$) and this relation was dependent of Class I compatibility: Concurrence of viral infection and a partial Class I match (1 to 3 matches in A+B loci) was associated with acute rejection ($p<0.01$) but, none of these circumstances without the other carries a significant risk of acute rejection.

In conclusion, these results suggest that in liver transplant the simultaneous presence of partial class I match and viral infections might lead to an increased allograft antigenity and trigger the allogenic response involved in the acute rejection.

### P132

EVALUATION OF BACTERIAL AND FUNGAL SURVEILLANCE CULTURES IN LIVER TRANSPLANTATION.

Nuño J, Cuervas V, Vicente E, Turrión V, Pereira F, Barrios C, Mora NP, Ardaiz J.
Liver Transplant Unit. Puerta de Hierro & Ramón y Cajal Hospitals. Madrid. Spain.

**INTRODUCTION**: Surveillance cultures *(SC)* are a common practice in the follow-up of patients undergoing liver transplantation (LT), though, their utility has not been clearly clarified. The aim of our study is to analyze the diagnostic yield of these post-LT *SC*, assess their microbiological aspects and their financial cost.

**PATIENTS AND METHODS:** The clinical records of the first 139 consecutive LT performed in 121 patients in our hospital were analyzed. Standard immunosuppression included cyclosporine A and steroids. Selective bowel decontamination was performed during the first 21 post-LT days with oral quinolones, associated with nystatin (n=95), or fluconazole (n=44). *SC* for bacteria (anaerobes included) and fungi were routinely performed on a daily basis during the first post-LT week, and 3 times a week thereafter until hospital departure and also when clinically indicated.

| **RESULTS** | **S/T** | **ORL** | **Urine** | **BC** | **Bile** | **AE** | **AD** | **VC** | **Stools** |
|---|---|---|---|---|---|---|---|---|---|
| Patients with *SC* | 100 | 134 | 137 | 41 | 119 | 138 | 121 | 104 | 98 |
| Patients with (+) *SC* | 54 | 94 | 61 | 32 | 79 | 95 | 55 | 47 | 83 |
| Total number of *SC* | 397 | 1662 | 1949 | 52 | 1592 | 2.996 | 325 | 241 | 479 |
| Total positive *SC* | 162 | 408 | 180 | 41 | 321 | 393 | 99 | 66 | 258 |
| (+) *SC* / patient | 1.6 | 3.0 | 1.3 | 1.0 | 2.6 | 2.8 | 0.8 | 0.6 | 2.6 |
| (+) *SC* / total *SC* | 0.4 | 0.2 | 0.09 | 0.8 | 0.2 | 0.1 | 0.3 | 0.2 | 0.5 |
| Infectious episodes | 61 | 61 | 12 | 12 | 7 | 56 | 56 | 72 | - |
| *SC* cost / patient ($) | 105 | 296 | 262 | 37 | 738 | 970 | 152 | 133 | 125 |

**S/T**: Sputum / trachea. **BC**: bladder catheter (*ct*). **AE**: abdominal exudates.**AD**: abdominal drainage *ct*. **VC**: vascular *ct*.

Gram-positive cocci (GPC) were isolated in 90.5% of the LT (mainly coagulase-negative Saphylococcus, n=619); fungi in 61% (mainly Candida sp., n=453); and gram-negative bacilli (GNB) in 49% (mainly Pseudomonas sp., n=180). Overall, the most common pathogens were GPC (63% of those isolated), followed by fungi (21%), GNB (15%) and others (1%). The choice of antiinfectious therapy was based exclusively on the *SC* and was determined prior to clinical onset in only 7 of the 197 infectious episodes recorded. Despite the *SC*, 13 of the 21 patients with invasive mycosis died; in 5 cases (24% of the mycoses), diagnosis was not obtained until autopsy. **CONCLUSIONS**: The low diagnostic yield of the bacterial and fungal *SC* and their high financial cost, make their utility in LT questionable.

### P133

BACTEREMIA AND FUNGEMIA IN LIVER TRASPLANT PATIENTS UNDER SELECTIVE BOWEL DECONTAMINATION (*SBD*) REGIMEN.

Nuño J, Cuervas V, Vicente E, Turrión V, Pereira F, Barrios C, Mora NP, Ardaiz J.
Liver Trasplant Unit. Puerta de Hierro & Ramón y Cajal Hospitals. Madrid. Spain

**INTRODUCTION**: By 1976 the reported incidence of bacteremia or fungemia in liver transplantation (LT) exceeded 70%. Nowadays, it concerns between 26% and 39% of patients. The aim of our study is to analyze the incidence, etiology, source, and related mortality of bacteremia and fungemia during the first 3 post-LT months in a series of 139 LT.

**PATIENTS AND METHODS**: The clinical records of the first 139 consecutive LT performed in 121 patients in our hospital, (March 1, 1986 to January 1, 1992), were analyzed in order to identify those with blood cultures presenting bacterial or fungal positivity. Immunosuppression therapy consisted of cyclosporine A and steroids. Rejection episodes were treated with a 3-day steroid pulse and recycling. The monoclonal antibody OKT3 was employed for treatment of steroid-resistant rejection. *SBD* was performed during the first 21 days post-LT with oral norfloxacin (400 mg/d, n=108) or ciprofloxacin (250 mg/d, n=31), associated with nystatin ($2x10^6$ U, n=95), or fluconazole (100 mg/d, n=44). Blood cultures were obtained only when clinically indicated.

**RESULTS**: 39 patients (32%) (43 LT, 31%) had at least one blood culture presenting bacterial or fungal positivity, wiht a total of 69 episodes (.1.6 episodes/patient): 60 bacteremia episodes, 7 of fungemia and 2 involving both. Sixty-three were monomicrobial and 6 polymicrobial. Fifty-seven (82%) took place in the first post-LT month, 9 in the $2^{nd}$ and 3 later on. No differences were apreciated according to the antibiotic prophylaxis employed. The origin of the causal infection could be identified in 38 (55%) episodes as follows: abdominal (18), pneumonía (16), catheter (4). Four episodes were considered as sample contamination owing to the low growth index (1/6 positive blood cultures) and the kind of microorganism (staphylococcus epidermidis). There were 27 episodes (39%) with an unknown source. The pathogens identified were: gram-positive cocci (GPC) (54), gram-negative bacilli (12), Candida sp. (8), anaerobes (2) and Aspergillus sp. (1). Sixteen of the 39 patients died (41%). Mortality rate was 29.5% (n=5) *vs* 50% (n=11) (N.S.) among those with infection of unidentified source and those with a known origin, respectively.

**CONCLUSIONS**: Under a *SBD* regimen with quinolones the incidence of post-LT bacteremia is high (32%), without a significant difference between norfloxacin or ciprofloxacin. Fungemia episodes are much lower (4%). The predominant pathogens are GPC. In 39% of cases, the origin of infection remains unidentified.

### P134

LONG-TERM OUTCOME OF BONE DISEASE AFTER LIVER TRANSPLANTATION

M.A. Valero [1], C. Loinaz [2], A. Alvarado[2], M. Leon[1], A. Lopez[2], R. Gomez[2], C. Jimenez[2], E. Moreno[2], F. Hawkins[1]

Service of Endocrinology1 and Depertament of Surgery2, University Hospital 12 de Octubre, Madrid, Spain.

Background: Osteopenia is a major complication of orthotopic liver transplantation (OLT). However, few data have been published about long-term bone disease after transplantation. We have evaluated vertebral bone mineral density (VBMD) and biochemical markers of bone metabolism in 120 patients after OLT.

Material and methods: VBMD ($L_2$-$L_4$) was measured after OLT by Dual energy X-ray absorptiometry (Hologic QDR 1000) in two occasions, separated by 12 months. Fasting serumintactPTH(iPTH), 25(OH) vitamin D, 1,25$(OH)_2$ vitamin D, osteocalcin (BGP) and carboxyterminal propeptide procollagen type I (PICP) were measured by RIAs and serum calcium (Ca) and phophorus (P) by autoanalyzer. Our goal was to estimate annual bone loss in no osteoporotic patients without bone treatment (77 patients).

Results: Prevalence of Osteoporosis (Z below - 2SD) was 35,8%. In no osteoporotic group VBMD was 0,906 ± 0,100 g/cm$^2$ (Z = -0,93 ± 0,82). After 12 months, VBMD was 0,880 ± 0,111 g/cm$^2$ (Z = -1,11 ± 0,89). Annual bone loss was 2,9%. In the first study, serum BGP (7,8 ± 4,4 ng/ml) and PICP (205,0 ± 80,5 ng/dl) levels exceeded our normal range. However, Ca, P, iPTH, 25(OH) and 1,25$(OH)_2$ vitamin D were within normal range. After 12 months, Ca (8,9 ± 0,7 mg/dl), P (3,6 ± 0,4 mg/dl) and iPTH (38,3 ± 17,9 pg/ml) were in normal range, but BGP (9,6 ± 6,0 ng/ml) remained higher than normal. No differences in serum biochemical markers were found between the two measurements.

Conclusion: Our results suggest that in no osteoporotic patients bone density decreases during the follow-up period after OLT, in spite of the fact that bone synthesis is increased.

### P135

INTRAOPERATIVE BLOOD TRANSFUSION AND REJECTION FOLLOWING ORTOTOPIC LIVER TRANSPLANTATION (OLT)

J.C. Palomo, C. Jimenez, E. Moreno, I. Garcia, C. Loinaz, I. Gonzalez, A. Alvarado, F. Palma
Gral. Dig. Surgery Dept. "12 de Octubre" Hos.Madrid. Spain.

INTRODUCTION.- Blood transfusion can alter the immune status of the recipient. OLT is associated with the use of large volumes of blood, considerably more than with most other surgical procedures. This communication reviews the influence of intraoperative blood transfusion on rejection in patients undergoing OLT.

PATIENTS AND METHODS.- Ninety-seven OLTs is adult patients (age range: 16-67 year) were reviewed. Patients were grouped into three categories according to intraoperative blood volumes transfused: group A (n=30), 1.5 or less blood volumes transfused (mean volume: 1 ± 0.3); group B (n=34), more than 1.5 but less than 3 volumes used (mean volume: 2.1 ± 0.3), and group C (n=33), when 3 or more volumes were given (mean volume: 5 ± 1.7). Preoperative clinical parameters analyzed in recipients showed significant difference in previous abdominal surgeries between group C and other groups. Also albumin serum level was significantly lesser in group B than A. Donor-recipient ABO compatibility, total ischemia time, steatosis and preservation injury in liver grafts showed no difference between groups. Warm ischemia time was significantly longer in group C than in B. Postoperatively we analyzed acute rejection (AR) and chronic rejection (CR) in each group.

RESULTS.- 1.- AR incidence was lower in group C (57.6%) without significant difference between groups (group A, 76.6%) and group B, 70.6%). 2.- Mean number of AR episodes per patient was lower in group C (0.8 ± 0.8 AR episodes) and higher in group A (1.5 ± 1.2 AR episodes). 3.- Group A patients also experienced a significantly higher incidence of steriod-resistant AR episodes/patient than group C patients (0.66 ± 0.8 vs 0.24 ± 0.5). 4.- There was no significant difference in the grading of AR episodes between groups. 5.- CR incidence was lower in group C (7.7%) than in groups A and B (23.3% and 31.2% respectively). The difference was only significant between B and C.

CONCLUSIONS.- Intraoperative blood transfusion has an

### P136

POST-TRANSPLANT HEPATITIS DUE TO HCV INFECTION IN LIVER TRANSPLANT RECIPIENTS: A PROSPECTIVE CLINICAL AND PATHOLOGICAL STUDY.

MªJ.Domingo; C.Loinaz; A.Alvarado; C.Lumbreras; F.Colina; R.Gómez; C. Jiménez; A.Fuertes; I.González-Pinto; E.Moreno. Hospital "12 de Octubre". Madrid (SPAIN).

Hepatitis C virus (HCV) infection is the main cause of post-transplant hepatitis (PTH) in liver transplant (LT) recipients. In order to evaluate the long-term clinical and histological consequences of PTH due to HCV infection, we have performed a prospective study in 44 consecutive LT recipients infected by this virus HCV infection was defined by the presence of VHC-RNA in a post-transplant serum sample by means of PCR using primers of the 5´untraslated region. Liver biopsies were obtained when liver tests became abnormal and routinely once a year. Mean follow-up was 29 months.

PTH was diagnosed in 29/44 (66%) infected recipients. The mean time elapsed from transplantation to the diagnosis of PTH was 316+242 days (57-814). PTH was a largely asymptomatic complication, and only 5/29 developed mild to moderate hepatitis related-symptoms. Twenty-one of the 29 HCV infected patients who developed PTH showed chronic active hepatitis (19) or cirrhosis (2) in the last available biopsy. Histological activity measured by the Knodell index increased during the follow-up (mean of 7.5+3,3 at PTH diagnosis vs. 9.8+3.6 in the last available biopsy; p 0.05). Eleven of the 44 recipients died during the follow-up, but only in one case the death was related to liver failure due to HCV-PTH, and retransplantation was not performed in any patient who developed PTH .

In conclusion PTH was very frequent among HCV infected LT recipients. From a histological point of view graft lesion seemed to be progressive and severe. However, at this length of follow-up PTH induced by HCV was not directly associated with graft failure or mortality.

### P137

MORBI-MORTALITY AND SURVIVAL IN LIVER TRANSPLANTATION RELATED WITH THE AGE OF THE DONOR

C. Jimenez, C. Esquivel*, E. Moreno, I. G-Pinto, C. Moreno, C. Loinaz, M.A. G. Ureña, A. Alvarado, J. Bercedo.
Hospital "12 de Octubre". Serv. Cirugía Ap. Digestivo "C" y Trasplante. *Pacific Medical Center. San Francisco (USA).

INTRODUCTION.- The aim of this study is to investigate the possible influence of the donor liver age over the results in the liver transplantation (OLTx), measured by the morbi-mortality and survival rates in two different age groups (pediatric and adult).
PATIENTS AND METHODS.- We considered a group A of 43 pediatric donors (<18 years old and mean age of 6.9 ± 6.1 years, whose livers were transplanted to recipients of different ages (mean: 17.8 ± 21.4 years) and another one group B of 48 donors (>of 18 years old and mean age of 28.6 ± 10 years) whose livers were transplanted to a group of recipients with a mean age of 46.3 ± 13.3 years. After transplantation, we comparatively analyzed the postoperative mortality, patient and graft survival, acute and chronic rejection, infections, surgical complications and retransplant rate.
RESULTS.- The postoperative mortality was 7% (3 patients) in group A and 8.3% (4 patients) in group B. The patient actuarial survival was: 93% in group A and 91.6% in B at one month, 93% in A and 86.7% in B at six months, 88.6% in A and 86.7% in B at one year, without significant differences (N.S.) between the groups. The same happened (N.S.) with the graft actuarial survival: 90.7% in group A and 81.2% in B at one month, 87.9% in A and 76.1% in B at six months and 83.5% in A and 76.1% in B at one year. There was N.S. differences in the rates of acute rejection (82.5% in A and 73.8% in B) and chronic rejection (2.4% in A and 2.3% in B). There was N.S. differences in the infection rates (65.9% in A and 63.6% in B). The overall rate of surgical complications was the same in both groups (37.2% in A and 37.5% in B). The retransplant rate was double in group B (14.6%) than in A (N.S.).
CONCLUSIONS.- In spite of the significant difference of age between the donor groups, we did not find significant differences in the rates of morbi-mortality and survival between the groups.

### P138

RECIPIENTS OF LIVER TRANSPLANT WITH ACUTE REJECTION DISPLAY DIFFERENT DQB1*03 ALLELES PROFILES THAN THOSE WITHOUT ACUTE REJECTION.

M. MURO, J. ONTAÑÓN, A. MINGUELA, R. ROBLES**, J. M. RODRÍGUEZ**, A. TORÍO, L. MARÍN, M. MIRAS*, F. SÁNCHEZ BUENO**, A. M. GARCÍA-ALONSO AND M. R. ÁLVAREZ.
(Sección de Inmunología, *Servicio de Medicina Digestiva, Servicio de Cirugía General, Hospital Universitario Virgen de la Arrixaca, Murcia, Spain)

Different species have consistently shown that the liver behaves as an immunologically favoured organ. However, the influence of HLA in liver transplant remains uncertain in contrast to other solid organ allografts. In addition, it is reported that certain DQ alleles could be implicated in the induction of immune responses in several autoimmune diseases. The aim of project was to study the DQ phenotypes expressed by liver transplant recipients and establish their relationship with acute rejection episodes.

A non radioactive SSOPs to screen PCR-amplified DNA from peripheral blood lymphocytes to analyse the polimorfism of HLA DQB1 was used. Forty-five liver recipients were studied, whose diagnosis of acute rejection was based on the conventional clinical and anatomopathological criteria. The exact Fisher test was used to contrast HLA DQB1 frequencies in patients with acute rejection (AR) and those without acute rejection episodes (NAR). The significance level was set to 0.05.

Significant augments were observed for DQB1*0302, in the AR group compared to controls ($p<0.01$) and NAR group ($p<0.01$). On the other hand, DQB1*0301 allele appeared significantly decreased in the AR groups when compared to the NAR group ($p<0.05$). However, although DQB1*0301 seemed decreased in the AR group when we compared it to controls, no significant differences were observed in this case. In contrast, the NAR group showed a similar distribution to the control group.

The observed results, suggest that the DQB, specially DQB1*03 alleles, locus could be implicated in the regulation of the allogenic immune response in liver transplant recipients.

### P139

LIVER DEVASCULARISATION IMPROVES THE HYPERKINETIC SYNDROME OF PATIENTS WITH FULMINANT HEPATIC FAILURE.

R. Noun, E. Zante, A. Sauvanet, F. Durand *, J. Bernuau *, J. Belghiti
Department of Digestive Surgery and *Hepatology, Hôpital Beaujon, F-92110 CLICHY.

High cardiac output and low systemic vascular resistance often occur in the course of fulminant hepatic failure (FHF). Recent reports suggested that vasoactive metabolites derived from the failing liver are involved in this hyperdynamic state and leaded to the concept of salvage hepatectomy for rapidly deteriorating patients.
When starting total hepatectomy into patients with FHF, by interrupting the blood supply to the failing liver, we have observed an improvement in the hyperdynamic state. We report herein the hemodynamic modifications relative to liver devascularisation of 24 consecutive patients undergoing liver transplantation (LT) for FHF.
Patients and methods: From July 1991 to March 1994, 24 patients with a mean age of 38 ± 12 years (range, 16-62) underwent LT for FHF. The cause of hepatic failure was drug induced hepatitis in eight, paracetamol poisoning in two, acute hepatitis B infection in five, and of unknown origin in nine. In all the patients, severe confusion (n=9) or coma (n=15) were associated to a mean factor V of 13± 5% (range, 4 - 23). The procedure started in all, by hepatic artery transection and by an end-to-side portacaval anastomosis. Baseline (T0) and 5 min. after devascularisation (T1) hemodynamic data were recorded.
Results: Hemodynamic results are reported in the following table.

| | T0 | T1 |
|---|---|---|
| Mean arterial pressure(mmHg) | 79±14[a] | 87±11[a] |
| Cardiac index($L.min^{-1}m^{-2}$) | 6.8±1.6 | 6±1.3 |
| Pulmonary capillary wedge pressure(mmHg) | 9±4.9 | 9.5±4.9 |
| Systemic vascular resistances($dyn.sec.cm^{-5}$) | 541±179[b] | 698±130[b] |

[a] $p<0.05$. [b] $p<0.02$

After liver devascularisation elevated baseline CI decreased whereas low initial SVR and MAP significantly increased.These modifications occurring without variations in cardiac filling pressure and in the absence of vasopressive agents.
Conclusion: The hemodynamic benefit after devascularisation of a failing liver suggests that total hepatectomy with a temporary portacaval shunt may be indicated to stabilise some patients with a threatening hemodynamic condition.

### P140

RECIPIENT HEPATECTOMY WITH PRESERVATION OF INFERIOR VENA CAVA: ROUTINE TECHNIQUE IN ORTHOTOPIC LIVER TRANSPLANTATION.

C.Margarit, J.L.Lázaro, J.Balsells, R.Charco, E.Murio, I.Bilbao, E.Gifre, C.Ruiz.
Liver Transplant Unit. Hospital "Vall d'Hebrón". Barcelona. Spain.

Some patients do not tolerate the inferior vena cava (IVC) and portal clamping during the anhepatic phase of liver Tx and an a veno-venous bypass is needed. Recipient hepatectomy with IVC preservation (piggy-back technique) was introduced in our program in cases of segmental liver Tx in children and in adult patients with portocaval shunt. Later on, it has been the routine technique in all cases. Graft implantation is performed by anastomosing the donor suprahepatic IVC to the stump of the recipient hepatic veins whereas the donor infrahepatic IVC is closed. We present our experience with this technique.
Between october 1988 and november 1994, 168 liver tx in adult patients have been performed in our Unit. In the first period (47 LTX), veno-venous bypass was used in 28 cases (59%), and crossclamping in the rest. In the second period, since the introduction of the piggy-back technique, 121 LTX were performed. Venovenous bypass was used in only 4 cases (3.3%), piggy-back technique in 112 (92.6%) and crossclamping in 5 (4.1%).
There has been a significant reduction of the need of venovenous bypass in the second period. Operating time, PRC, plasma and platelet transfusion were significantly higher in venovenous bypass group. No complications related to the piggy-back technique were found.
Conclusion: Piggy-back technique reduces the need of venovenous bypass with the consequences of saving time, blood transfusion and reducing the cost of liver transplant.

### P141

### INFERIOR MESENTERIC VEIN CANNULATION FOR VENO-VENOUS BYPASS IN SELECTED LIVER TRANSPLANT RECIPIENTS

B Nardo, S Todo, H Furukawa, A Steiber, TE Starzl, JJ Fung.
Department of Transplantation Surgery, University of Pittsburgh Medical Center, Pittsburgh, PA 15213, USA.

The inferior mesenteric vein (IMV) cannulation for veno-venous bypass during orthotopic liver transplantation (OLT), for decompressing the portal system, has been used in 6 selected liver transplant recipients. From May 1, 1985 to January 31, 1993 a total of 2667 liver transplants on adult recipients were performed. IMV cannulation has been used in 6 patients (5 M, 1 F) with mean age of 39.3 years (range 21-60). Four patients underwent primary OLT for end-stage liver diseases. The reason to use IMV cannulation for veno-venous by-pass was because of difficult hilar dissection in 5 cases and because of portal vein thrombosis after H-graft portocaval shunt in one case. Main hemodinamic parameters like heart-rate, cardiac output, mean arterial pressure, central venous pressure, pulmonary artery pressure, were monitored before during and after the IMV bypass in this group of patients. Arterial blood gas data, $Na^+$, $K^+$, $Ca^{++}$, glucose, osmolarity and lactate were also monitored. Similarly, these parameters were monitored in a group of 6 liver transplantations during which was performed the portal vein cannulation technique for the veno-venous bypass. Total bypass time, temperature change, bypass flow, total intraoperative transfusion of PBRC (units) and urine output were recorded in both groups. Statistical analysis was performed using ANOVA test. The statistical analysis of all the parameter values showed no significant variation before during and after the veno-venous bypass in the IMV cannulation group as well as in the portal vein group. Furtherly, no significant difference was found between the two study groups for those parameters. Four patients are alive and well respectively with 8.5 years, 2.3 years, 14 months and 9 months. Two patients died; one 3 weeks after the operation because of multiorgan failure and sepsis, the other one, 1 year later because of multiorgan failure. Difficult hilar dissection or portosystemic shunt with portal vein thrombosis are the main indications for the IMV cannulation for bypass system. Our intraoperative results confirm that good hemodinamic stability is obtained using this modified technique. In conclusion, IMV cannulation for veno-venous bypass is an effective procedure for early decompression of the portal system in case of an impossible portal vein cannulation.

### P142

### IN-SITU SPLITTING OF THE LIVER IN THE HEART BEATING CADAVERIC ORGAN DONOR FOR TRANSPLANTATION IN TWO RECIPIENTS

K.A. Gawad, X. Rogiers, M. Malago, W.T. Knoefel, A. Frilling, T.E. Langwieler, U. Dahmen, B. Nachtwey, W. Pothmann* and C.E. Broelsch
Departments of Surgery and Anesthesia and Intensive Care*, University Hospital Eppendorf, Hamburg, Germany

Split-liver transplantation presents an interesting concept to alleviate the organ shortage for children with end-stage liver disease. The procedure has, however, not gained wide acceptance yet. This is not only related to the complexity of the procedure, but also to the less good results and the complications reported on the right side graft.

We report on a first case in which we applied a new concept for splitting. The liver was splitted in-situ in the heart beating cadaveric donor using the technique of living related liver procurement, with the aim of reducing the problems with the right side graft. The two recipients (one adult with alcoholic cirrhosis and one child with Crigler-Najjar-Syndrome II) are alive and at home six months postoperatively. The child has a progressive worsening graft function following portal steal by native liver with ischemic damage to the graft.

This procedure makes splitting of the liver possible without compromising the hilar structures, with the possibility to judge perfusion and to achieve optimal hemostasis. Therefore, in-situ splitting of the liver has the potential of making splitting of liver grafts the rule rather than the exception, thus increasing the organ pool significantly.

### P143

### Liver transplantation in patients with diffuse nodular hyperplasia

LOINAZ C, *MUSELLA M, COLINA F, ALVARADO A, GOMEZ R, GARCIA I, MORENO GE.
Department of General and Digestive Surgery and Abdominal Organs Tranplantation-University Hospital "12 de Octubre" Madrid-Spain. *CNR-ESTERO. Italy.

Problems associated with portal hypertension, such as ascites, bleeding esophageal varices and sometimes hepatic encephalopathy, frequently complicate an unusual form of a noncirrhotic liver disease, the diffuse nodular hyperplasia (**DNH**). Since June 1986 until December 1994, 402 liver transplantations (**OLT**) have been performed in our center. Three cases of liver **DNH** treated with **OLT** are here summaryzed. **Patient 1)** A 36-year-old man presented in December 1987. The preoperative clinical and instrumental diagnosis was: patient with cronic hepatopaty probably due to ethilism, B/C Child grade, esophageal varices III/IV grade, splenomegaly. The pt. was transplanted on February 18, 1988 and he feels currently good. The histopathologic response was: **diffuse nodular hyperplasia. Patient 2)** A 40-years-old man presented in June 1988. He underwent kidney transplantation due to Alport syndrome in 1976. The tranplantation provoked a chronic rejection with progressive kidney insufficiency. In this period the pt. developed a hepatic **DNH** with severe portal hypertension, esophageal varices IV/IV grade and hypersplenism with splenomegaly. He received a double hepatorenal transplantation on October 21, 1989 with histologic confirmation of hepatic **DNH**. Since then he has presented a valid renal function, complaining one episode of obstructive post-transplant uropathy. The hepatic histology has been relatively compromised by an acute B hepatitis arose on chronic C hepatitis. **Patient 3)** A 37-years-old man presented in January 1994. The physical and instrumental examinations revealed: chronic diffuse hepatopaty, with a previous C hepatitis virus infection, portal hypertension with several episodes of esophageal varices bleeding, portal vein thrombosis. The pt. was transplanted on April 3, 1994 and the histopathologic finding was **DNH**. He suicided three months after receiving **OLT** despite of his satisfactory conditions. **OLT** may be considered an effective therapy for those patients bearing life-threatening or disabling complications of advanced portal hypertension due to diffuse parenchymal non-cirrothic chronic disease.

### P144

### VASCULAR PROBLEMS IN LIVER TRANSPLANTATION

J. Carles, V. Dubuisson, E. Rullier, J. Saric

Unité de Transplantation Hépatique, Hopital Pellegrin, Bordeaux, France

Standardization of reconstruction procedures in liver transplantation led to an improvement of graft and patient survival. Arterial reconstruction remains the "Achille's heel" of the procedure ; inferior vena cava (IVC) reconstruction has been modified recently by introduction of the "Piggy-back" procedure in which the recipient's IVC is conserved. We focalized our study on those tow aspects. We review a series of 165 consecutive orthotopic liver transplantations performed in 146 patients including 3 children. Recipient's IVC was conserved in 32 transplantations. IVC complications occured in 5 cases (3 thrombosis, 1 Budd-Chiari syndrom and 1 stenosis), always when the recipient's IVC was not conserved. The rate of arterial complications was 11 % with 8 stenoses, 5 thrombosis and 5 pseudoaneurysms ; 4 patients underwent a new transplantation and 7 died. Biliary complications were more frequent in case of arterial complication, specially when the arterial blood flow was interrupted (thrombosis or surgical ligature of a ruptured pseudoaneurysm). In conclusion, the Piggy-back procedure is now routinely used for IVC reconstruction. Arterial complications have severe consequences, specially on the biliary tract. Early recognition of such complications is essential.

### P145

**K+- and pH Activity on the Liver Surface: Determination of Graft Viability and Preservation Quality during Liver Transplantation**

J. Kurzbach, A.Visser, M. Gundlach, R. Kuhlenkordt, X. Rogiers, C.E. Broelsch
Dept. of Surgery, Univ. Eppendorf, Martinistr. 52, 20246 Hamburg, Germany

**Introduction**: It is known from experimental and clinical studies, that $K^+$- and pH are indicators for graft viability and preservation quality. In a first preliminary study we tested a system based on ionselective electrodes for $K^+$ and pH during liver transplantation. **Patients and Methods:** Measurements were taken on the liver surface of segment 3, 4 and 5 in (n=27) donors and (n=19) liver recipients: before explantation; at the end of cold ischemia; 45 min. after reperfusion of the liver graft. For preservation UW solution was used. Postoperative follow up included SGOT-Scoring and transplant biopsies. **Results:** The measurements showed no significant difference between accepted (n=19) and refused (n=8) grafts, of whom 7 were discarded because of clinical parameters, fatty liver or fibrosis. There was no correlation between $K^+$- and pH levels and the fat content of the liver. Measurements before perfusion did not correlate with donor complications (e.g. Hypotonia, Diab. insipid). As well no correlation with reperfusion injury was found. $K^+$ increased significantly ($p<0.05$) with the duration of cold ischemia. Most transplants showed normal levels of $K^+$- and pH levels after reperfusion. In two grafts poor distribution patterns of $K^+$ indicated a reduced postoperative liver function. **Conclusion**: Measurements of $K^+$- and pH in donor livers provide no additional objective parameters for the selection of liver grafts. In individual cases there is a correlation of the measurements with perfusion and reperfusion injury. More cases have to be studied to show whether $K^+$- and pH changes correlate with perfusion and reperfusion injury.

### P146

POSTOPERATIVE CLINICAL IMPROVEMENT AFTER LIVER TRANSPLANTATION IN FAMILIAL AMYLOID POLYNEUROPATHY (1st CASE IN GREECE)

G. Kostopanagiotou, N. Arkadopoulos, M. Tzagournisakis, J. Kapiris, I. Phocas, J. Papadimitriou
Second Dept. of Surgery, Dept. of Anesthesiology, Areteion Hospital, University of Athens and Dept. of Neurology, University of Crete, Heraklion, Greece

Familial amyloid polyneuropathy (FAP) transthyretin (TTR) related disease is a disorder of protein metabolism with a fatal course. FAP is a autosomal dominant disease, associated with progressive autonomic and peripheral neuropathy because of amyloid's deposition. Replacement of the liver to correct this metabolic deficit, that causes damage to other organs, has been reported previously. A 33 years old female Greek patient had developed sensorimotor neuropathy and autonomic symptoms of diarrhea, difficulty in swallowing and mild urinary retention as well as orthostatic hypotension and weight loss. Amyloid affected the kidney, liver, small bowel and heart as confirmed by biopsies. Molecular analysis by PCR amplification of genomic DNA followed by Nsil digestion was performed. The data documented the presence of the TTR Met-30 variant. Our patient underwent orthotopic liver transplantation (OLTx) in September 1993 being the first case of liver replacement for metabolic disease in Greece. The patient had an uneventful recovery. During the follow-up period she manifested two episodes of mild rejection (15th postop. day and 9th postop. month). The patient 15 months after OLTx presents with improvement of shooting and throbbing pain in the lower limbs and disappearance of nausea, vomiting, urinary retention, inappetence and orthostatic hypotension. Sensory deficits in arms and legs, distal motor weakness in the legs has slightly improved during the course of the last few months. Body weight has increased (+6 Kg). OLTx seems to have benefits in FAP, but multidisciplinary clinical studies are required to determine the role of liver replacement.

### P147

INTEREST OF TRANSJUGULAR INTRAHEPATIC PORTO-SYSTEMIC SHUNT BEFORE ORTHOTOPIC LIVER TRANSPLANTATION.

J. BAULIEUX, E. DE LA ROCHE, G. GENIN, C. DUCERF, C. PIGNAL, E. PETIPAS, M. ADHAM, Ph RENY, A. RODE, O. BORSON, M. POUYET.
Service de Chirurgie Digestive et de la Transplantation Hépatique - Hôpital de la Croix Rousse Lyon 69004.

Evolution of liver cirrhosis during the period preceeding orthotopic liver transplantation (OLT) was frequently marqued by severe complications related to portal hypertension (PHT). Between November 1991 and July 1994, 12 patients (10 men & 2 women) mean age 47,4 years received transjugular intrahepatic porto-systemic shunt (TIPSS) before OLT. Indications for OLT were : 5 post-hepatitis cirrhosis (hepatitis C n=3; hepatitis B n=2), 1 primary biliary cirrhosis, & 6 alcoholic cirrhosis. Indication for "TIPSS" were upper gastrointestinal tract hemorrage (UGITH) for 8 cases and refractory ascitis in 4 cases. Some complications were recorded during these setting : 1 migration of the "TIPSS" into the pulmonary artery, 3 thrombosis (2 early & 1 late) and 2 stenosis were treated by baloon dilatation, 2 cases of transient encephalopathy. Evolution of Child classification was : regression of the ascitis n=2, stabilisation n=1, & aggravation n=1. UGITH were stopped in 5 cases, 3 patients had recurrent UGITH, 2 of them received baloon dilatation and new successful attempt of "TIPSS". During surgery difficulty related to the "TIPSS" were recorded in 3 patients. PHT regressed in 8 cases & persisted in 4. Mean packed RBCs transfused during OLT were 6,4 (0-26) and mean Fresh Frozen Plasma were 16,7 (0-47). Post operative mortality was 8,3% (1 case) secondary to air embolism. "TIPSS" constitute an interesting helpfull mean before OLT : - It can prevent & treat complications related to PHT. - Baloon dilatation can be done in unsuccessful "TIPSS". - It avoids abdominal aproach for treatment of PTH, source of surgical difficulty during OLT. - It facilitates dissection during OLT & minimize peroperative hemorrage. But its proper morbidity seems to decrease by experience.

### P148

**ADHESION MOLECULES AS A USEFUL MARKER OF PRESERVATION INDUCED INJURY IN ORTHOTOPIC LIVER TRANSPLANTATION**

**A. Bhargava[1], N. J. Bradley[1], A. K. Burroughs[2], A. P. Dhillon[3] K. Rolles[1], B. R. Davidson[1].**

**[1]University Department of Surgery, [2]Liver Transplantation Unit and [3]Department of Histopathology**

**Royal Free Hospital School of Medicine, Pond Street, London NW3 2QG UK.**

Preservation induced injury, a combination of ischemia and reperfusion, results in endothelial and hepatocellular damage. Adhesion molecules (A.M.) are cytokine inducible polypeptides involved in leukocyte/endothelial cell interaction.

The aim of this study was to compare liver allograft A.M. expression following reperfusion with degree of preservation injury classified by serum levels of aspartate transaminase (AST).

Following reperfusion (at 90 mins), liver biopsies from 28 grafts were snap-frozen. 5μm frozen sections were stained immunohistochemically for Intercellular adhesion molecule-1 (ICAM-1), E-Selectin, Platelet and endothelial cell adhesion molecule (PECAM) and Vascular cell adhesion molecule (VCAM). Intensity of stain and distribution was analysed by light microscopy and compared to normal liver.

Levels of AST were measured upon arrival in the ITU. Patients were placed into 2 groups: those with AST > 1000 i.u. classified as moderate/severe preservation injury. AST < 1000 i.u. were classified as minimal preservation injury.

12 patients had AST > 1000 i.u., of these, 8(75%) had all four A.M.s expressed at a greater intensity than normal controls with *de novo* appearance of E-selectin on endothelial cells, ICAM-1,PECAM and VCAM on sinusoids and ICAM-1 on hepatocytes. In the group with AST < 1000 only 2/16 had all four A.M.s increase in intensity.

Moderate/severe preservation injury is associated with a higher intensity of expression of cytokine inducible cell A.M.s. providing evidence of endothelial cell damage. This is probably related to Kupffer cell activation. Attraction of inflammatory cells into the graft together with cellular damage result in poor early graft function.

### P149

### A NEW NON-INVASIVE OXYGEN SURFACE ELECTRODE FOR THE CONTINUOUS MEASUREMENT OF LIVER BLOOD FLOW DURING ORTHOTOPIC LIVER TRANSPLANTATION (OLT)

A. M. Seifalian, B. R. Davidson, and K. Rolles
University Department of Surgery and Liver Transplantation, Royal Free Hospital and School of Medicine, London, U.K.

**Aim:** The purpose of this study is the first to our knowledge to investigate liver blood flow perfusion intraoperatively in a population of patients with end-stage liver disease during OLT. To allow a more accurate assessment of liver perfusion, we have modified a Clark-type oxygen electrode. We used an electrode with high oxygen consumption thus rendering it more sensitive to blood flow rather than to oxygen partial pressure (Po2), and this was achieved by having a large cathode (silver, 3 mm in diameter) and a membrane with high permeability to oxygen (12.5 μm Teflon).
**Method:** In 16 patients, with mean age = 51 ± 11 years the electrode was applied to the liver surface and in one patient an electromagnetic flowmeter (EMF) was applied to the portal vein (PV). Continuous readings of perfusion from the surface of the liver were examined with respect to (a) effects of PV perfusion up to 30 min. after revascularisation of the PV blood flow, (b) effects of hepatic artery (HA) perfusion up to 30 minutes after revascularisation of the HA blood flow.
**Results:** There was a good correlation between liver tissue perfusion using oxygen electrode against EMF in stepwise clamping of PV ($r = 0.953$, $p < 0.001$). Re-perfusion of the transplanted liver with venous blood was accompanied by an immediate increase in liver blood flow perfusion. Over the subsequent 10-30 minutes there was no significant increase in flow and re-perfusion of the graft with arterial blood did not increase liver blood flow perfusion.
**Conclusion:** This surface electrode allows continuous monitoring of liver blood flow in chosen sites and it is cheap and simple to use.

### P150

### ACUTE GALLSTONE CHOLECYSTITIS: PRELIMINARY RESULTS AFTER A TWO-PHASE MANAGEMENT.(A prospective clinical study).

K.J.Manolas, N.Lyratzopoulos, G.Minopoulos, P.Kalofolias and K.Romanidis.
Dept of Surgery, University of Thrace School of Medicine, University Reg. Gen. Hospital, Alexandroupolis HELLAS.

The optimum timing for surgical intervention in acute gallstone cholecystitis (AGC) still remains a matter of debate. Early surgery during the acute phase is followed by higher morbidity and mortality rates than conservative management which, in its turn, seems to be time and money consuming.

The aim of this prospective clinical study was to investigate the effect of a combined therapeutic protocol (conservative originally followed by surgery) upon the overall morbidity and mortality rates, in cases of AGC, and its cost effective impact.

Between 1990 and 1994 in 37 consecutive patients with AGC (13 males-24 females, m.a.62,5yrs) the conservative management resulted in full clinical and laboratory recovery within 7 on average days (range 6-12 days). All these patients were discharged and were readmitted for appropriate surgery after 4-6 weeks. The mean perioperative hospitalization lasted 10 days on average. The overall morbidity rate was 14,5% and was exclusively due to superficial thrombophlebitis which was developed during the acute phase from TPN intravenous catheters in 6 out of 37 patients. There were no deaths throughout this serries.

It is assumed that the beneficial results obtained with this therapeutic regimen, especially by being applied on a relatively high-risk group of patients, may form a basis for comparison with other therapeutic modalities and for future reference.

### P151

### ACUTE CHOLECYSTITIS: URGENT OR ELECTIVE SURGERY ?

A. López Labrador, M. Abradelo, C. Loinaz, A. Alvarado, P. Rico, M.A. Corral, M. Manzanera, E. Moreno González.
General and Digestive Surgery Dept. "12 de Octubre" Hospital. Ctra. de Andalucia Km. 5,5. Madrid. Spain.

INTRODUCTION.- Acute cholecystitis is a common pathology. However timing of the surgical indication is still controversial.
MATERIALS AND METHODS.- From January 1986 to December 1993, 121 patients (75 females and 46 males) were operated on with diagnosis of acute cholecystitis. Mean age: 67.6 years (females: 70,15 ; males: 65 years). Age range was 26-91 years. Forty six of these patients (group I) underwent urgent surgery (less than 72 hours after diagnosis). The remaining 75 (group II) were operated on after three days. Mean age of the first group of patients was 72 years and 65 for the second group.
RESULTS.- Morbidity in group I was 15%. Three patients died due to to respiratory insufficiency and progressive deterioration (2 neurologic patients). 25/46 (54.3%) had perforation of the gallbladder, gangrenous or emphysematous cholecystitis or empyema. In group II 23% of morbidity and 4% of mortality (1 respiratory failure, 2 sepsis). 21/75 (28%) had perforation, gangrenous cholecystitis empyema. No emphysematous cholecystitis. Hospital stay was 15.05 days in group I, and 28,1 days in group II.
CONCLUSIONS.- Acute cholecystitis has a high morbidity and mortality rate. In order to reduce hospital stay and morbidity we recommend prompt surgical treatment with close monitoring of seriously ill patients.

### P152

### MISDIAGNOSIS OF BILE PERITONITIS (BP)

Yang Sen Hua, Yang Yi cun

Department of Surgery, Chengdy Third People's Hospital, Chengdu, Sichuan,P R china

BP is a severe disease. Sometimes its diagnosis might be difficult, making the treatment delayed. In this hospital in recent years 24/57 cases (42.1%) with BP were misdiagnosed, aged 32-76 (≥60 in 12 cases), with previous history of biliary diseases (BD) in 10. Pain shifting into the right lower qudrant presented in 8 cases, jaundice in 8,epigastric or right upper abdominal tenderness in 24, diffuse tenderness and rigidity in 13 and abdominal distension in 13. BP complicating severe acute cholecystitis or/and cholangitis were operatively demonstrated in these 24 cases, being preoperatively misdiagnosed as acute appendicitis (AA), perforated peptic ulcer, acute pancreatitis, upper gastrointestinal bleeding or acute BD. There are various causes of BP in which acute BD is mostly reponsible. To diagnose BP is generally not difficult, but not easy in some cases belonging to the subacute and chronic groups and also in the elderly. The misdiagnosis in our patients included miss of diagnosis of BP (11 cases) and causes of peritonitis misinterpreted(13). The chief causes of errors and ommissions in diagnosis were : (1) Severe inflammation of biliary tract (BT) with no naked perforation, might have increased permeability of the tract from impaired mucosal barrier, permitting the bile to exude into the abdominal cavity only presenting distension in chemical stimulation . (2) The exuded or leaked bile would drain to the parocolic gutter, mimicing shifting pain of AA, the more prominent signs of the primary focus in the upper abdomen omitted. (3) Abdominal pain was one-sidedly supposed to be the syptome of peptic ucler. (4) Abdominal Paracentesis (AP) is diagnostic for BP, but only adopted in 2 cases (1 positive, 1 negative), contrasting with 16/16 cases confirmed as with BP by this procedure in the accurate diagnosis group (33). (5) The elderly ( ≥60 ) consisted of a half of the misdiagnosed cases. They made more difficulties in diagnosis. Besides, the following are noteworthy in diagnosing: (1) A high index of suspicion of BP should be maintained when peritonitis occurred in potential cases such as those with previous history or operation of BD. (2) BP could not be casually excluded in cholecystitis without the presentation of jaundiuce and chill. (3) Early signs in some cases might only be tachycardia (110-140 in 19 cases) and abdominal distension. (4) AP should be more actively chosen in doubtful cases. The mortality of BP is high---37% (21/57 deaths) and delayed diagnosis is one of the main lethal causes.

### P153

### PREVENTION AND TREATMENT OF BILIARY INFECTION (BI) AFTER CHOLEDOCHODUODENOSTOMY (CD)

Yang Sen Hua, Yang Yi Cun

Department of surgery, Chendu Third People's Hospital, Chengdu, Sichuan, P R China

Finsterer CD is a simpler operation. sometimes with BI as a serious postoperation, possibly inducing a fatal outcome. How to prevent and treat this complication is worthy of studying. In this hospital since 1973 there were 21 cases perfomred on with CD, chiefly for common bile duct (CBD) disease and chronic pancreatitis and the procedures as follows.

| Procedure | N of cases | Postoperative follow-up | | |
|---|---|---|---|---|
| | | Results(N) | Period (Yr) | Cause |
| CD+ Billroth | 10 | Fair (4) | 11-13 | |
| II gastrectomu (BII)* | | Death (1) | | Cholangitis |
| Finsterer CD | 8 | Fair (3) | 5-13 | Changing CD into Choledochojeunostomy(1) |
| | | good (1) | 2 | As above |
| | | Death (4) | | Cholangitis |
| | | Fiar (1) | 1 | |
| Posterior CD(PCD)** | 1 | | | |
| CD+Duodenojejunostomy (DJ) | 2 | Fair (2) | 18/12-2 | |

*BII preformed in one(7) and two stages( 3); end-to-side CD ( 5); additional PCD (1) in the third stage, finding turmide bile deposited in the lower CBD.

**Anastomosis of about 4 cm between the lower CBD and desending duodenum.

Chief factors contributing to postoperative BI included: (1) Indication of CD wrongly chosen(3) with intrahepatic stone or stricture, repeated cholangitis resulted. (2) Retrograde biliary reflux (RBR). Repeated chemical stimulation to BT is harmful and BI unavoidable, favorable to the formation of gallstones. BII and DJ could effectively prevent and treat the RBR. (3) "Blind lump." End-to-side CD simply eliminates this phenomenon. PCD diminishes the lump to a high degree with satisfectory result (2 ). (4) Stenotic CD (3). CD needs a wide stoma (1.5-3.5 cm in our practice). These 3 cases complicated with AOSC had been emergently reoperated on(2 recoveries, 1 death). Post-CD cholangitis is often very severe, requiring timely surgery. For achieving fair result, the individual indications should be strictly followed, and the potential complications of CD effectively prevented.

### P154

### ELECTIVE CHOLECYSTECTOMY FOR LITHIASIS. OPERATIVE FINDINGS VS PREOPERATIVE CONSIDERATION

G. Kyratzis, I. Virlos, L. Lapidakis, L. Papastamatiou
2ND Dpt of Surgery "Apostle Paul" Hosp.-KAT, ATHENS-HELLAS

Simple cholecystectomies are well known as easy operative procedures, without mortality (0.018%). In some cases neither history and physical examination nor laboratory results are indicative of pre-existing complications of the underlying lithiasis and operative problems are suddenly faced with.

This study deals with 132 such cases, out of a total of 1098 simple cholecystectomies (12.09%) performed over the last 10-year period. Sex ratio was female 2.3:1 male and the mean age 61.2 years (43 - 97 y). Wrinkled intrahepatic gallbladder 54c. Hydrops 14c. Acute cholecystitis 22c. Gangrene 11 c. Empyema 13 c. and Choledocholethiasis 21 c. The above operative findings necessitated modifications of the operation and postoperative treatment.

This controversy between preoperative estimation and operative findings is mostly due to long standing lithiasis without acute symptoms in correlation with advanced age of the patients. Another 296 cases were promptly estimated as complicated preoperatively.

It is concluded that in a respectable percentage of "simple" cholecystectomies arise major operative problems, demanding proper and immediate management regardless of operative time or general condition of the patient.

### P155

### THE ROLE OF $PGI_2$, $TXA_2$ AND BILE PROTEIN IN THE PATHOGENESIS OF CHOLESTEROL GALLSTONE FORMATION

Wenxuan Yang*, Dezheng Xu
Department of Surgery, First Affiliated Hospital,
Xinjiang Medical College, Urumqi, China.
*Present address: Department of Surgery, King's College School of Medicine & Dentistry, The Rayne Institute, London, U.K.

This study was performed to investigate the role of prostaglandins (PG) and bile protein in pathogenesis of gallstone formation. $PGI_2$ and $TXA_2$ in gallbladder mucosa in patients with chronic cholecystitis and gallstones were determined by RIA of 6-keto-$PGF_{1\alpha}$ and $TXB_2$. Bile protein was measured with fluorometric and amino acid analysis. Pathological and histochemical changes in gallbladder mucosa were observed to estimate the degree of inflammation and glycoprotein synthesis. The results show that increased $PGI_2$, $PGI_2/TXA_2$ values and bile protein were consistent with the degree of gallbladder inflammation and staining grade (PAS,AB) for glycoprotein. The values of $PGI_2$, $PGI_2/TXA_2$ in the cholesterol gallstone group (30.07±5.36, mean±se) were significantly higher compared to the groups with pigmented stones (11.53±1.76) and acalculous cholecystitis (15.35±4.41), ($P<0.05$, Student's unpaired t-test). Bile protein in the group of cholesterol gallstone (1.64±0.14) was much higher than the pigmented stone group (0.77±0.14) and control (0.95±0.11), ($P<0.01$). The conclusion is that $PGI_2$, $PGI_2/TXA_2$ and bile protein were related to the progress of gallbladder inflammation and glycoprotein synthesis and probably plays a significant role in cholesterol gallstone pathogenesis.

Key words: $PGI_2$, $TXA_2$, bile protein, cholecystitis, gallstone formation

### P156

### INTRAHEPATIC LITHIASIS: VALIDITY OF THE SURGICAL TREATMENT

A. Principe, M. Lugaresi, A. Mazziotti, F. D'Ovidio and G.Gozzetti.
2° Department of Surgery - Policlinico S.Orsola - University of Bologna - ITALY

The primary goals in the management of intrahepatic lithiasis (IL) are: 1) remove the stones from the intrahepatic bile ducts; 2) reestablish a good biliary flow; 3) prevent recurrences. Aim of this study is to demonstrate that the surgical treatment is the best solution of the problem.

Material and Methods: Since 1982 37 pts (22m, 15F, mean age 53 years, range 24-78) have been observed.

In one case the etiology was primitive while in the rest it was secondary to anatomical disorders or to stone migration from the extrahepatic ducts. The intraoperative workup with cholangiography and ultrasounds revealed the exact location of the stones in 100% of the cases while the preoperative diagnostic assessment was exact only in the 62.8% of the cases. The hepatic diffusion was multiple (MD) in 8 cases and segmental in 29 (SD). Patients wityh MD were treated with lithectomy, associated to a wide biliary digestive anastomosis (BDA) at the hilum in 6 cases, in one the BDA was completed with a cutaneous stoma, while in the remaining 2 cases the BDA was intrahepatic. Of the 29 pts with SD 5 were treated with lithectomy, associated to papillosphincterotomy and to BDA in 16 of which one was intrahepatic. In the remaining 8 pts was performed an hepatic resection.

Results: One patients died in the post operative period for hepatic failure. Four had specific complications for which 2 cases needed reintervention. Lithectomy was not complete at surgical intervention in 3 cases (8%) one of them was successfully treated with ERCP. The rest of the pts are to date in good clinical conditions without recurrence.

Conclusions: To date surgical management is the gold standard for intrahepatic lithiasis: it permits a precise localization of the stones; a definitive solution of the problem; low recurrence rate and small percentage of incomplete lithectomy although always curable with a non surgical approach.

### P157

**ABO BLOOD GROUP AND GALL STONES IN THE GREEK POPULATION**

S.N.Pagoni*, S.A.Paximadas*, A.G.Vlachos**, M.Agiannitou*, V.Carathanasis**, E.Niotis**
First Dept. of Internal Medicin, General Hospital of Athens "ELPIS"* and First Dept. of Surgery, 417 N.I.M.T.S. of Athens**, Greece.

Purpose: The possible correlation between ABO blood group and gall stones.
Patients: 169 inpatients with symptomatic or asymptomatic gall stone disease (56 males and 113 females) aged 59±14 (range 16-94 years) were studied. Diagnosis was confirmed with ultrasound and/or cholocystography and in some patients surgically.
Methods: ABO blood group of 169 patients (Group A) compared with 1.283 random volunteer blood-donors at our hospital (Group B).
Results:

| | AB | A2B | A | A2 | B | O | Rh+ | Rh- |
|---|---|---|---|---|---|---|---|---|
| Group A | 8* | 2* | 55* | 8* | 21* | 75* | 148* | 21* |
| Group B | 3 | 23 | 422 | 55 | 131 | 514 | 1059 | 124 |

* P=Not Significant
Statistical analysis using t-test and $x^2$.
Similar results emerged in patients aged <60, <50, <40 and <30 years.
Conclusion: These data give some support to the hypothesis that the ABO blood group isn't a risk factor for developing gall stones in the Greek population. It was also observed that the frequency of lithogenesis and cholelithiasis depends on age, gender, diet, obesity, drug regimens, liver function, abnormalities in lipid metabolism, haemolytic anaemias e.t.c.

### P158

**INFLUENCE OF DIET, PHYSICAL ACTIVITY AND HEREDITY IN THE CREATION OF CHOLELITHIASIS IN THE GREEK POPULATION**

A.G.Vlachos**, S.A.Paximadas*, S.N.Pagoni*, C.Costaglanis**, E.Vervesou*, A.A.Armenaka*
First Dept. of Internal Medicin, General Hospital of Athens "ELPIS"* and First Dept. of Surgery, 417 N.I.M.T.S. of Athens**, Greece.

Purpose: Possible causes of cholelithiasis in the Greek population.
Patients: 172 individuals (54 males and 128 females) aged 60±17 (range 16-88 years) with asymptomatic cholelithiasis were studied. Diagnosis was confirmed with ultrasound and/ or cholocystography.
Methods: Investigation of the role of dietary and environmental factors in patients with cholelithiasis [overall diet, olive oil consumption, coffee drinking, smoking, work environment, living conditions, heredity factors (stomach and duodenal ulcers, diabetes mellitus, cholelithiasis inparents)].
Results: Dietary habits-Environmental factors-Habitation: Predominately meat-eaters 31%, fish-eaters 26%, vegetarians 24%, and mixed diet 20%, olive oil consumption 86%, coffee drinking 52%, smoking 25%, easy working conditions 34%, average conditions 56%, and hard labor 10%, urban 55%, rural 26% and both 19%, heredity factors (stomach and duodenal ulcers 17%, diabetes mellitus 20%, cholelithiasic parents 23%).
Conclusion: Diet, lifestyle and heredity do not seem to play a significant role in the creation of cholelithiasis in the Greek population.

### P159

**BILIARY LITHIASIS IN THE ELDERLY PATIENT. MORBIDITY AND MORTALITY OF THE SURGERY**

J.J.González, J.L.Graña, G.Bermejo, L.Sanz, F.Navarrete, E.Martínez

Department of Surgery B. Hospital "Covadonga". Universidad de Oviedo. SPAIN

Surgical operations for gallstones are associated with an increase in perioperative mortality in the elderly. The operative risk factors were assessed in patients older than 80 years to develop methods to improve patient management.
In this study, there were 76 patients, mean age of 83.1 ± 2.96 (80-93) years. They all underwent operations between January 1989 and December, 7(9.2%) on elective basis. 58 (76.3%) patients were seen with associated preoperative diseases. From a clinical point of view, it is noteworthy taht, upon admission , 33 patients (43.4%) had jaundice and 21(27.6%) fever. The operative findings included gallbladder wall infection in 46 patients (60.5%) and common bile duct stones in 25 patient. Uni and multivariate analysis were performed to discriminate variables in relation to mortality and morbidity.
Nine patients (11.8%) died, and 38 had complications which developed in the postoperative period (50%). The main causes of death were pulmonary complications (4) and multisystem failure (3). The morbidity was associated mainly with wound infection (14), urinary infection (13) and respiratory disease (10). Three variables showed influence on morbidity: sex (men), cardiovascular disease and jaundice upon admission. When they were introduced in the regression model only cardiovascular disease (p=0.01) and jaundice (p=0.008) revealed independent influence. The mortality rate was associated with preoperative jaundice (p=0.01).
Mortality and morbidity are related mainly to preoperative presentation, irrespective of surgical findings and supplementary procedures to cholecystectomy. Jaundice is the main determinant of the future.

### P160

THE LONG-TERM RESULTS OF CHOLELITHOLYSIS IN PATIENTS WITH GALLSTONE DISEASE

A.L.Grebenev, V.S.Golotchevskaya, L.P.Genya
Center "Gastroenterology (therapy)", Sechenov Moscow medical academy Russia

We studied the long-term results of the use of chenodesoxycholic and ursodesoxycholic acids drugs (Chenofalk, Ursofalk) in I04 patients with gallstone disease, specially selected, according to indications and contraindications. Patients received ordinary doses of medicines. Mean duration of treatment was I2 months. Stones dissolved completely in 22 patients, mostly in those, who had stones below I0 mm in diameter.
We followed-up I8 patients during 4,5 - 5 years In 9 of them formation of stones took place relatevely early - in I3 to I4 months (mean - II months). In the other 9 patients, who did not manifested formation of stones in these terms, gallstones were not found during following examination up to 4,5 years (mean - 36 months). None of different dietic and pharmacological methods of cholelithiasis prophylaxis demonstrated advantages for the others.In 9 patients with gallstone relapses the clinical flow of disaese became much better, with no episodes of gallstone colic.
Our results demonstrate high efficacy of the chenodesoxycholic and ursodesoxycholic acids drugs, such as Chenofalk and Ursofalk in treatment of the gallstone disease.

### P161

**THE SOMATOMETRY INDEX IN ASYMPTOMATIC CHOLELITHIASIS IN THE GREEK POPULATION**

**S.A.Paximadas*, A.G.Vlachos**, S.N.Pagoni**, L.Mantzaris*, S.Stavrianidis**, E.Vervesou***
**First Dept. of Internal Medicin, General Hospital of Athens "ELPIS"* and First Dept. of Surgery, 417 N.I.M.T.S. of Athens**, Greece.**

**Purpose: Somatometry profiles in individuals with asymptomatic cholelithiasis in the Greek population.**
**Patients: 172 individuals (54 males and 128 females) aged 60±17 (range 16-88 years) with asymptomatic cholelithiasis were studied. The diagnosis was confirmed with ultrasound and/ or cholocystography.**
**Methods: The somatometry index was investigated. Height, Weight, Body Mass Index (BMI = Weight in kg/(Height in m)²), Waist (W), Hips (H) and the W/H ratio of 172 individuals with asymptomatic cholelithiasis (group A) compared to males in group A (group B) and females in group A(group C).**

| Results: | Group A | Group B | Group C | |
|---|---|---|---|---|
| Weight (kg) | 73±12 | 77±11 | 72±12 | # |
| Height (cm) | 164± 9 | 174± 7 | 160± 7 | ## |
| BMI ($kg/m^2$) | 27.3± 4 | 25.3± 3 | 28± 5 | * |
| Waist (cm) | 95±12 | 95±11 | 95±12 | NS |
| Hips (cm) | 110±12 | 105± 9 | 111±13 | ** |
| W/H | 0.87±0.1 | 0.91±0.1 | 0.86±0.1 | *** |

*** p=0.01, ** p=0.02, *** p=0.003, # p=0.05, ## p<0.0001, NS=Not Significant**
**Conclusion: The somatometry profile which emerged in patients with asymptomatic cholelithiasis is that the BMI is approximately in the normal range (≤27 kg/m²), while there was a statistically significant difference in both the BMI and the W/H ratio in male versus females patients.**

### P162

**CHOLELITHIASIS IN PATIENTS OVER 80 YEARS OF AGE**

J.L. Graña, J.J. González, L. Sans. G. Bermejo. F. Navarrete, E. Martínez.

Department of Surgery B. Hospital "Covadonga". Universidad de Oviedo. SPAIN.

Recent advances in the medical care of elderly patients has resulted in an increased life span, thus increasing the number of people alive who are older than 80 years of age. The purpose of this paper is to review our experience in the treatment of biliary lithiasis in this group of patients.
From January 1989 to December 1993, 209 patients older than 80 years of age, with symtomatic gallstones, were admitted to our unit. The mean age was 84.6 +/- 3.66 (80-96) years. We studied the role of associated disease, treatment changes and prognosis.
201 (96.2%) were admitted as emergency cases. 108 (51.7%) were without past biliary disease. 159 (76.1%) had evidence of associated diseases. Upon admission 84 (40.2%) had jaundice, 73 (34.9%) right upper quadrant peritonitis, and 49 (23.4%) pancreatitis. Around 50% had increased levels of leukocytes, alkaline phosphatase, direct bilirrubin and/or serum creatinine. 76 (36.4%) patients underwent surgery, mainly in the same admission (median 13 days). The operative findings included gallbladder wall infection in 46 (60.5%) cases and common bile duct stones in 25. ERCP was perfomed 23 (11%) times. 61 (29.2%) patients had complications, and 28 (13.4%) died, mainly from respiratory diseases and multisystem failure. The operative mortality was 4.3%.
The majority of elderly patients with gallstones are "high risk" patients (preoperative associated diseases, jaundice, leukocytosis, elevated serum creatinine level), which means that your treatment still has high morbidity and mortality.

### P163

THE COMPLICATIONS OF ESWL TREATMENT (FIVE YEAR'S EXPERCINCE)

İ. Özman, G. Avcı, S. Bora, H. Bakır, M. Füzün, N. Erkan

Dokuz Eylül University Medical Faculty General Surgery Department, İzmir -TURKEY

Between July 1989 and July 1994 167 patients were treated with ESWL plus litholytic agent for symptomatic gallbladder disease. Major and minor complications were observed in 55 patients (32%). As a major complication acute pancreatitis was observed in 7 patients (4%) and 4 of them were treated at first by medical means and later surgically while 3 patients were operated urgently and one of them had died. Two patients with acute cholecystitis were operated (0,01%). As minor complications, cutaneous pathesia was observed in 33 patients (19%), transient obstructive jaundice in 2 (0,01%), microscopic haematuri in 4 (0,2%) and cardiac aritmia in one patient (0,05%). In conclusion, ESWL has serious complications and must be used in selected patients with symptomatic

### P164

LITHOTRIPSY FOR RETAINED BILE DUCT STONES

S. Bora, G. Avcı, İ. Erdamar, H. Gülay, İ. Özman, E. Tankurt

Dokuz Eylül University Medical Faculty General Surgery Department, Inciraltı, İzmir - TURKEY

Over 3 years, 17 patients with retained bile duct stones underwent extracorporeal shock wave lithotripsy (ESWL) by using a second generator lithotriptor. Stone visualization in the common bile duct was achieved by T tube cholangiograpy in 10 and via nasobiliary drainage tube in 7 patients. Sphincterotomy was performed in only 7 patients. Stone disintegration and complete clearance was achieved in 14 patients (83.2%) after a mean of 1.2 ESWL sessions. Three patients were operated because of failed ESWL treatment. As a serious complication hemobilia was seen in one patient (5.9%). ESWL seems to be an alternative therapy for retained bile duct stones with or without other non-surgical procedures.

### P165

**ESWL FOR TREATMENT OF DIFFICULT BILE DUCT STONES.**

D. Lomanto, F. Fiocca, M. Nardovino*, E. Grasso, E. Lezoche**, E. Zarba Meli, F. Giacovazzo, P. Lepiane, F. Carlei, V. Speranza.
II Clinica Chirurgica, University of Rome "La Sapienza";* Hepato-Biliary Divison, INI Canistro (AQ); ** Patologia Chirurgica, University of Ancona.

In recent years, alternatives to surgery for difficult CBD stones have been developed. Routine endoscopic measures fail in about 10% of patients, while advanced endoscopic procedures such as laser, electrohydraulic lithotripsy or dissolution by solvents, require a skilled endoscopist or a close and effective physical contact with the stone, therefore these procedures are technically difficult and sometime uneffective. ESWL can be used to disintegrate stones and since1989 has been applied in CBD stones. To verify the usefulness of ESWL in biliary tract stones, we treated, from 1990 to 1994, 26 patients (16 F-10 M), mean age 67±20 yrs (range: 34-89). 16 (62%) had multiple stone and size range:10-25 (mean: 18 mm). We utilized the Dornier lithotripters HM4 (X-ray guide, n=16) or MPL 9000 (US guide, n=10). 1513± 521 shock waves (range:260-2226) was delivered in 68.8±25.4 min (range: 28-104) at 22±2.5 Kv (range:18-25), triggered by an ECG. All patients have had an endoscopic (n=22) or surgical sphincterotomy (n=4). In pts treated by HM4 the stones were visualized by contrast medium injected through a nasobiliary tube (n= 8), a postsurgical drain (n= 4; 2:T-tube, 1:transcystic and 1:cholecystostomy) or a PT catheter (n=4). In 4 pts i.v. opiate analgesia has been necessary. In all pts endoscopic or radiologic routine or advanced measures had previously failed. 20 pts had CBD stones, in 2 pts (7.6%) the stones were localized in the RHD, in 2 pts in the LHD, in 1 pt at the carrefour and in one at the CHD. 2 pts had a massive lithiasis and in one pt GB was in-situ. 31% of pts needed two ESWL sessions and 2 pts three.Our results showed a mean stone size of 5 mm in 12 pts, 7 mm in 10 pts and no fragmentation in 4 pts. Complete clearance was obtained in 23 pts (88%) after one or more sessions either by endoscopic (n=17) or percutaneous extraction (n=6) of the debris; in the remaining 3 pts, in 2 a bilio-duodenal stents was placed and in one EHL was performed. Moreover, we report a 23% (6 pts) of transient mild hemobilia, microhematuria in 15%. No mortality was reported. In conclusion, in anatomic or size-related difficult biliary stones, ESWL, is an additional nonoperative option to resolve the failure of routine endoscopic measures. Moreover ESWL in contrast of the advanced procedures presents certain advantages: direct contact with the stone is not necessary, treatment is rapid, safe and highly effective.

### P166

FIVE YEAR'S EXPERIENCE WITH ESWL FROM A SINGLE CENTER

İ. Özman, G. Avcı, S. Bora, İ. Erdamar, H. Gülay

Dokuz Eylül University Medical Faculty General Surgery Department, 35340, İnciraltı, İzmir -TURKEY

Between July 1989 and April 1993, ESWL + litholytic therapy was performed in 167 patients with symptomatic gallbladder stones. One hundred and seventeen patients were female and 50 were male with ages ranged from 23 to 78 years (mean of 56.8 years). The number of gallbladder stones were 1,2,3 and multiple in 137, 13,11 and 6 patients respectively. The number of ESWL sessions were one in 61 patients, two in 59, three in 38, four in 7 and five in 2 patients. ESWL therapy was discontinued when the fragments of stones were lesser than 4mm. in diameter. In 114 patients (68%) the fragmentation was achieved while in the rest ESWL had failed. After successful ESWL treatment, chenodeoxycholic acid (CDA) was used as the chemolytic agent for 6 to 14 months (mean of 11 months). During the follow-up, 53 patients became stone-free but in 16 of them (30%) stone recurrence was detected between 3 months to 5 years after and treated with cholecystectomy. Major and minor complications were observed in 55 (32%) patients. In conclusion, ESWL can be used in selected patients with cholelithiasis having high risks for operative treatment.

### P167

CHOLECYSTECTOMY BY MINI-LAPAROTOMY WITH THE USE OF HAEMOSTATIC CLIPS.

M. Gyras, K. Tsolias, A. Papathanasiou, N. Sias.

1st Surgical Dep. Asklepeion Hospital
Therapeutic Hospital of Athens

**Our purpose is to describe the technique of cholecystectomy, through a small transverse incision with the use of haemostatic clips.**
**Our study includes 81 selected cases (67 women and 14 men) aged 22-88 years, between August 1, 1989 and December 1994. The method is based upon the principles of laparoscopic cholecystectomy. 1.- The surgeon's hands do not enter the peritoneal cavity. 2. The cholecystectomy is performed with haemostatic clips. 3. The surgical wound, as well as the postoperative incision, is greatly limited. (A small transverse incision of up to 5 cm, based on the preoperative clinical and ultra-sonic studies of the position of the gall bladder.) 4. The limitation of cost and hospital stay (the patient is fully motile by evening and is released the following day). 5. It has the added advantage that it can be done with spinal anaesthesia or even local anaesthesia.**
**In conclusion, we report that in our 67 selected cases, we had only two cases with post-operative complications.**
**In one case, due to bleeding, the mini laparotomy was extended to laparotomy.**
**In another case, we re-admitted the patient, because of a small retained gallstone, which passed using conservative treatment.**

### P168

**FIVE YEARS EXPERIENCE ON GALLBLADDER AND BILIARY TRACT SURGERY USING CONVENTIONAL METHODS**

**NESET KÖKSAL**
*1. DEPT OF GENERAL SURGERY, HAYDARPAŞA TEACHİNG HOSPITAL, ISTANBUL, TURKİYE*

The widespread use of laparoscopic surgery has resulted in decreased use of conventional methods in hepatobiliary tract diseases. This retrospective study is a report on 112 patients with hepatobiliary tract disease, who underwent open surgery during January 1988 and June 1992.

Of all the patients, 86 (77%) were cholecystectomized and 26 (23%) had common bile duct exploration in addition to cholecystectomy. All of the operations were performed from the left side of the patient, in 95 cases we preferred a midline superior incision. There were 96 females and 16 males, with a 1/6 male to female ratio. The mean age was 46.8 years. In 96.4% of the cases the underlying cause of the disease was cholelithiasis, where in one case it was neoplasia of the gall bladder, a polypoid lesion in one and acalculous cholecystitis in another.

The reason for common bile duct exploration was gallstones in 24 cases, choledochal cyst in one and hydatid disease in another. 29 cases underwent surgery in more than one organ system during cholecystectomy. We have observed anatomic variations of the biliary tract in 23 cases. 83 patients were operated under elective circumstances where as 29 underwent surgery in the emergency unit. We have observed two complications.

### P169

### ARE IATROGENIC LESIONS OF EXTRAHEPATIC BILE DUCTS DECREASING WITH INITIATION OF PROSPECTIVE STUDIES ?

Flis V, Koželj M.
Dept. of Vascular and Abdominal Surgery, Teaching Hospital Maribor, SLOVENIA

***Background***: Although complications in cholecystectomy are infrequent, bile duct injury following cholecystectomy is one of the most serious complications in surgery. Numbers are increasing with laparoscopic procedures.
***Methods***: The auhtors report on iatrogenic lesions of bile ducts injuries occured in Teaching Hospital Maribor, which were followed up in a prospective study between 1980-1989 and between 1990-1994. In the first period (1980-1989) 6646 open cholecystectomies were done (1927 men, 4179 women, average 52.6 years, range 14-91). In the second period 2646 open cholecystectomies and 235 laparoscopic (starting year 1992) procedures were performed.
***Results***: In the first period 8 cases (six women, two men, average 62 years, range 28-75) of iatrogenic bile duct injury occured. There were no iatrogenic injuries in the second period. Analysing the first period, seven lesions were detected and treated in the case of surgery, once lesion was overlooked. All lesions occured before intraoperative cholangiography was carried out. In partial lesion direct suture was applied once and three times suture over the T tube. In complete transsection termino-terminal reconstruction over the T tube was performed. In patient with overlooked lesion after six months stricture developed. She was reoperated and biliodigestive anastomosis by Roux was performed. In two female patients reoperations were required after primary reconstruction over T tube. In both cases finaly biliodigestive anastomosis after Roux was done. All patients are now without complaints.
***Conclusions***: In the first period there was 1 injury in 830 operations (0,12%). No iatrogenic injuries occured in the second period. It is stated that prospective follow up of iatrogenic bile duct injuries may decrease the morbidity after reconstruction. Even more, it may lead to disappearance of iatrogenic bile duct injuries.

### P170

### THE EFFICACY OF PREOPERATİVE AND INTRAOPERATIVE CRITERIA IN DETECTING COMMON BILE DUCT STONES

A. GÜRKAN, A. USLU, M. TİRELİ
SSK Tepecik Hospital, Third Department of Surgery, Izmir, Turkey

During the past 3.5 years, 657 patients were operated for bile stones in our clinic. In 83 of these patiens the common bile duct was explored as they had the suspicion for carrieing a stone. Patients who had had choledochotomy were assessed using 13 parameters, and their efficacy for the diagnosis was evaluated. The criteria were: age, sex, jaundice, pancreatitis, acute cholangitis, cholecystitis, palpation of stone, the diameter of common bile duct, small stones in the gall-bladder, patent cystic duct, hyperbiluribinemia, alkalen phosphatase,and ultrasound findings.
In 24 of the eigthythree patients one or more of the criteria were found to be positive, but in their exploration no stones were detected. Three of the parameters (palpation of the stone, jaundice, and alkalen phosphatase) were found to be efficant for detecting the common bile stones (p 0.01). When the diameter of the common bile duct is accepted as 12 mms., the width of the common bile duct has no value for dianosis; but when the cut-off point is taken as 15 mms. this parameter is effective in detecting the common bile duct stones.

### P171

### P172

### EXTRA-HEPATIC BILIARY SYSTEM TRAUMA: STUDY OF 45 CASES

M.A.C. MACHADO, P. VOLPE, L.F.C. ZANTUT, R.S. POGGETTI, D. BIROLINI.
Department of Surgery - University of São Paulo, Brazil.

Injury of the extra-hepatic biliary system lesion is infrequent, occurring in approximately 3.5 % of all patients with blunt and penetrating abdominal trauma. The incidence of this injury due to blunt abdominal trauma is rare.

The aim of this study is the analisys of 5069 patients with abdominal trauma treated at the Department of Surgery University of São Paulo (Brazil) over a six-year period to identify those with injury of the extra-hepatic biliary system. Forty five patients with gallbladder and extra-hepatic ducts injury were identified (0.89%) and divided in two groups according the nature of trauma: 12 due to non-penetrating injuries and 33 due to penetrating injuries. Records, including operative and pathology reports, were reviewed to study the site of injury, associated intra-abdominal injuries, incidence, trauma scores, treatment, morbidity, mortality rates and correlated with the nature of the trauma.

Overall mortality was 24.4%. The incidence was greater in the patients sustaining penetrating abdominal trauma ($p<0.05$). Forty of the 45 patients (88.9%) had liver lacerations, the most commonly seen injuries. The patients with blunt abdominal trauma had significant different trauma scores ($p<0.05$) than those with penetrating trauma, indicating greater severity in this group of patients.

We conclude that there is relation between severity of trauma and incidence of extra-hepatic biliary system injury. However in the penetrating trauma, the incidence of trauma is correlated with the direction of the wound and there is no relation with the severity of trauma. The greater mortality seen in the patients sustaining non-penetrating injury ($p<0.05$) supports this idea.

### P173

**THE SPILLED STONE: A POTENTIAL DANGER AFTER LAPAROSCOPIC CHOLECYSTECTOMY.**

E Mª Targarona, A Cifuentes, C Balagué, , J Martínez, J Tabet, M Trías.
Service of Surgery. Hospital Clinic. University of Barcelona. Barcelona. SPAIN

The spillage of stones is a frequent event during laparoscopic cholecystectomy, and initially, it was considered as a harmless operative incident. Some experimental studies have shown that stones leaved in the abdominal cavity induce immflamatory changes with a low incidence of intrabdominal abscess. Since 1991 to date, 50 cases of intrabdominal complications secondary to retained stones has been published and most of the required a reintervention to treat this complication. **Case report:** A 45-y-old-male diagnosed of symptomatic cholelithiasis, in which an ultrasonography revealed a gallbladder with a 4 mm wall and stones larger than 30 mm . Laparoscopic cholecystectomy was performed uneventfully but the gallbladder ruptured and several stones fell under the liver and it was not possible to retrieve one stone. The patient evolved satisfactorily. Two and a half y. later, the patient presented swelling in the right flank. X-ray examination showed a calcified image in the right fossaand a CT scan showed a round, well defined bilobular collection with an image compatible with a stone. The patient was operated and a well defined cavity containing sterile serohematic fluid was opened and a 30x15 mm stone were recovered. The patient evolved satisfactorily and is free of symptoms. **Comment.** Stone spillage has been not considered an indication of conversion of laparoscopic cholecystectomy, but it is now accepted that it is a source of infrequent but severe complications that may require a reintervention for treatment. Thus, it is recommended that any effort should be made to retrieve all the spilled stones and prolong the surgical procedure until this is achieved, in order to reduce one source of unpredictable morbidity. In selected cases, if a large number or big stones are lost, open retrieval should be considered.

### P174

INJURIES ON EXTRAHEPATIC BILIARY TREE

L. Lapidakis, L. Papastamatiou, D. Kalokerinos, A. Zarzali
2nd Dept. of Surgery "Apostle Paul" Hosp.-KAT. ATHENS -HELLAS

Injury on the extrahepatic biliary tree (EBT) associated to complex liver trauma is not often and as isolated injury is extremely rare. Biodynamic mechanisms and intraperitoneal conditions are responsible for EBT trauma.

Exact preoperative diagnosis especially in multiinjured patients is practically impossible. Moreover intraoperative recognition of EBT trauma is sometimes difficult, due to associated major visceral or vascular injuries. Therefore, early relaparatomy is "acceptable" for overlooked EBT trauma.

This report deals with 24 cases of EBT trauma detected in 160 cases of liver injury (15%), after blunt (22 cases) and penetrating (2 cases) abdominal injury, in the last 10-year period. In 18 cases injury was to the gallbladder, in 2 cases to the bile ducts and in 4 cases in both structures. All injuries were recognised during the emergency intervention, but chance was of great help in 2 cases of ductal trauma. All patients underwent cholecystectomy and either bilioenteric anastomosis or primary repair with T-tube for ductal injury. Mortality rate was high (54,2%) associated to coexistent injuries. One patient with penetrating isolated ductal injury died after sepsis and multiple organ failure. Three of the survivors developed early (bile leakage) and 2 of them late (stricture) complications, treated conservatively.

It is concluded that the detection of bile duct injury in multi-trauma patient is not easy. The type of the operative repair is dictated by the location and the extent of the ductal injury, but the operative technique applied is personalised by the choice of the surgeon.

### P175

MANAGEMENT OF IATROGENIC BILIARY TRACT INJURIES

RW Parks, EFA Spencer, EM McIlrath, GW Johnston
Professorial Surgical Unit, Royal Victoria Hospital, Belfast, Northern Ireland

Iatrogenic biliary tract injuries are not uncommon, and their management remains a significant challenge. The management and outcome of 33 consecutive patients with iatrogenic biliary tract injuries in a tertiary referral centre over a 21 year period are analyzed. The mean age was 43.5 years. The median time to diagnosis of the injury was 2 weeks (range = 0 - 11 years). The median time from original operation to referral was 3 months (range = 0 - 17 years). Thirty patients (90.9%) had undergone an open cholecystectomy, 7 of whom had exploration of the common bile duct; 2 patients had a laparoscopic cholecystectomy, and 1 patient had undergone revisional gastric surgery. Fifteen patients (45%) had undergone one or more subsequent operations prior to referral. Five patients (15%) had established secondary biliary cirrhosis, portal hypertension and variceal bleeding when referred. Percutaneous transhepatic cholangiography was the radiological investigation of choice. Six patients had percutaneous dilation, and 23 had surgical procedures in this unit, some patients requiring both radiological and surgical intervention. One patient is awaiting surgery and 1 patient died prior to intervention whilst undergoing investigation. Five patients in this series have been re-referred to other specialist hepatobiliry centres for further advice on management. Fifty per cent of those treated by balloon dilatation have subsequently required surgery. Of 23 patients who have had surgical reconstruction in this unit, only 2 have required revision surgery. Mean follow-up has been 5.7 years. We recommend early referral of patients with iatrogenic injuries to units experienced in dealing with such injuries, with no attempt at repair prior to referral.

### P176

IATROGENIC INJURIES TO THE BILE DUCT - SURGICAL MANAGEMENT

Z.Wajda, Z.Gruca, R.Marczewski
II Department of Surgery, Medical University of Gdańsk Poland

Fifty-four patients operated in between 1950 and 1990 were divided into three groups according to the injury, time of diagnosis and time of corrective procedure.
The first group consisted of 18 patients in whom reconstruction of the bile ducts was performed during the primary operation. The second group comprised nine patients in whom injury was diagnosed in the early postoperative period. The third group of 27 patients had restenoses after reconstruction performed in other hospitals. The primary operation, localization of the injury, diagnostic procedures and operative treatment of the bile duct injures were analysed in each group. Thirty-two patients reviewed from 3 to 20 years after corrective surgery were studied as long-term follow-up group. According to clinical examination, laboratory tests, radiography and biliary scintigraphy, long-term results were satisfactory in 24 patients.
The authors consider hepaticojejunostomy Roux-en-y to be the procedure of choice in the majority of patients with iatrogenic bile duct injuries.

### P177

### THE EFFECT OF NUTRITIONAL THERAPY IN THE MANAGEMENT OF EXTERNAL BILIARY FISTULAS

V.Alivizatos, Ch.Kyrkos, F.Karvelas, V.Athanasopoulos.
Department of Surgery and Nutrition Unit, "St.Andrews" General Hospital of Patras, Greece

This study was undertaken to assess the effect of Nutritional Therapy in the management of external biliary fistulas. During a seven year period (1987-1993) 7 patients, average age 66,8 years,were treated in our Department suffering from external biliary fistulas developed as s result of surgery in the hepato-biliary system. There were two high-output and 5 low-output fistulas which appeared 1 to 15 days after surgery. Four patients had moderate to severe malnutrition at the time of presenting of the fistulas. Five patients were treated with Total Parenteral Nutrition via a central venous catheter, and 2 patients with low-output fistulas have taken Total Enteral Nutrition via a fine naso-duodenal tube. All the fistulas closed after 7 to 17 days of treatment, except for one patient with high-output fistula who died from uncontrolled sepsis. The nutritional status remained unchanged at the end of the treatment in one patient, whereas it improved in all the others. No serious side effects were noted during the nutritional therapy. It is concluded that artificial Nutrition plays a significant role in the conservative treatment of the external biliary fistulas because it improves the nutritional status of these patients and maybe shortens the spontaneus closure time.

### P178

### IATROGENIC BILE DUCT INJURIES

M.Jekić,P.Aleksić,C.Hajduković,I.Jekić

Surgical Service,Clinical Hospital Centre and Institut for Digestive Surgery,Medical Faculty,Zemun-Belgrade,Yugoslavia

42 patients with iatrogenic bile duct damage were referred.Before referral,11 patients had no attempt at reconstruction,while 31 had undergone 41 operations to repair the damage. At admission,4 patients had secondary biliary cirrhosis,1 had portal vein thrombosis,and 1 had sepsis.Operative treatment includes 41 patients.Fifty-two operations have been performed,and 34 patients(83%)have had an exeellent long-term result,median 13 years.Five patients had 4 operations or more(before and after referral)and 3 are alive in good conditions. Various methods od repair were employed,and 8 patients(20%)had recurrence of stricture.Restricture was lowest for hepaticojejunostomy Roux-en-Y(15%),in particular when no stent was used across the anastomosis(8%).The hospital mortality rate was 2(5%)of 41 and overall mortality,7(17%)of 41.The lowest mortality rate(9%) was associated with hepaticojejunostomy Roux-en-Y.Low rate of recurrence and mortality are correlated to early referral.Patients with iatrogenic bile duct injury should be referred early to a competent center,where adequate treatment of infectioh,reconstruction with a hepaticojejunostomy Roux-en-Y without stenting,and lifelong follow-up can be performed.

### P179

### INTRAHEPATIC BILIARY STRICTURES AFTER RIGHT HEPATIC ARTERY OCCLUSION IN TRANSPLANT RECIPIENTS.

**B Nardo*, J Madariaga*, R Sheng°, AB Zajko°, S Iwatsuki*, TE Starzl***
**Department of Transplantation Surgery*, Department of Radiology°, University of Pittsburgh Medical Center, Pittsburgh, PA 15213, USA.**

Among the risk factors for the development of nonanastomotic biliary strictures after liver transplantation an important role has the hepatic artery occlusion. Four patients who developed intrahepatic strictures at the biliary duct confluence after right hepatic artery occlusion are presented. During a 10-year period, 643 liver transplantation patients (687 allografts) with choledochojejunostomy biliary anastomosis underwent 1728 cholangiographic studies. The presence, number, and locations of biliary strictures were recorded. Cholangiograms showed intrahepatic biliary strictures in 105 allografts (15.2%), anastomotic strictures in 105 allografts (15.2%), and nonanastomotic extrahepatic biliary strictures in 17 allografts (2.5%). Hepatic artery occlusion was detected in 28.8% (32/111) of the allografts with nonanastomotic strictures and in 6.6% (7/105) of the allografts with anastomotic strictures. Right hepatic artery occlusion was seen in 4 of the 105 allografts with intrahepatic biliary strictures (3.8%). Clinical presentation included fever and cholangitis in all cases. The diagnosis of intrahepatic biliary strictures was established in all cases by cholangiography. Cholangiographic findings included multiple strictures at the biliary duct confluence. Two patients had peripheral reconstitution of the occluded artery via intrahepatic collaterals. Percutaneous balloon dilatations combined or not with stent placement has been the treatment of choice in all patients. Hepaticojejunostomy was performed in one patient because of recurrent biliary sepsis. There was 1 death directely related to intrahepatic biliary strictures. The patient developed multiple intrahepatic strictures requiring retransplant 9 months later; a third graft had to be placed to treat severe rejection and he died from sepsis and multiple organ failure 20 days later. Three patients are alive and well at 3.5 years, 2.8 years and 2.7 years after diagnosis. Cholangiographic findings of intrahepatic biliary strictures should be evaluated for occlusion of the hepatic artery or its branches as probable cause.

### P180

### ADMINISTRATION OF SOM ATOSTATIN IN BILIARY FISTULAS COMPLICATING LIVER HYDATID DISEASE SURGERY.

M.Velegrakis, Th. Kokkinakis, M. Savvidou, A.Saroukos, E.Gaganis, S.Kandylakis.
First Department of Surgery, Venizelion General Hospital, Iraklion Crete Greece.

The occurence of external biliary fistulas after liver surgery for hydatid cysts is not rare. On several occasions their frequency ranges from 3.8-5.7%.

Therapy is often troublesome especially in Hospitals where there is no possibility of endoscopic treatment (nasobiliary tube, sphincterotomy). There are studies in which it is claimed that the administration of synthetic somatostatin decreases the production of bile by approximately 30% due to its inhibitory action on the composition of biliary acids, cholesterol and phospholipid.

Considering the above, synthetic somatostatin was used to treat the external biliary fistulas complicating liver hydatid disease surgery.

Over the past five years, ten cases of large hydatid cysts of the right hepatic lobe of have been operated on our department and partial capsectomy with drainage of the remaining cavity was carried out. External biliary fistulas developed in four cases, three of which had an output of 400-600 ml per day. Treatment was conservative and synthetic somatostatin was administered. There was positive response to the conservative therapy with gradual decrease of output to zero in 22, 17 and 28 days respectively.

It appears that synthetic somatostatin has a major role in the theatment of external biliary fistulas. However, further studies are required to confirm its effectiveness.

### P181

SURGICAL TREATMENT OF SPONTANEOUS INTERNAL BILIARY FISTULAS.

C. Jiménez, D. Rodríguez, C. Moreno, P. Rico, D. Hernández, J.C. Palomo, A. López, M. Manzanera, A.Alvarado, E. Moreno.

General and Digestive Department. "12 de Octubre" Hospital. Crtra. Andelucía 5,400. Madrid. Sapin.

INTRODUCTION: Internal biliary fistulas are usually late complications during the course of cholelithiasis, most frequent in the sixth decade of life and are associated with high morbidity-mortality because of age and concomitant diseases. The preoperative diagnosis is often difficult.

PATIENTS AND METHODS: From November 1973 to May 1992 we operated upon 2700 patients with biliary pathology, 47 of them with internal biliary fistula (incidence: 1.74%). The clinical presentation was as gallstone ileus in 11 patients (23.4%) and as a biliary fistula in another 36 patients confirmed at operation. The mean age of this series was 65.9 years (range: 39-83 years), 27 were women and 10 were men.

RESULTS: The clinical findings were: abdominal pain in30 patients (64%), jaundice in 19 (40%), fever in 8 (17%), guarding in 6 (13%) and dehydration in 5 (11%). By means of radiological studies (plain films, oral cholecystography, i.v. cholangiography, gastroduodenal barium stydi, ultrasonography anal CT) we suspected (pneumobilia, stone) or preoperatively diagnosed a biliary fistula in 10 cases (21%). The confirmation of the fistulas was at operation, and the location was: cholecystoduodenal in 23 patients, cholecystocholedochal in 11, cholecystohepatic in 4, cholecystogastric in 1, cholecystoduodeno-colic in1, and cholecystocholedochalcolic in1. In five patients we did not classify the fistula because we did not disect the fistulous tract (gallstone ileus). In the 36 remaining patients without a gallstone ileus picture, the surgical techniques were: cholecystectomy and fistula repair in all cases, also adding: sphincteroplasty in 13, choledochojejunostomy in 3, choledochoduodenostomy in 2, and T-tube in 5. In 16 of these patients (44.4%) we found choledocholithiasis. Of the spontaneous resolution and in the 10 remaining we carried out enterolithotomy (in 3 of these we performed cholecystectomy and fistula repair and on one only a cholecystostomy). The postoperative mortal-morbidity was 34%.

CONCLUSIONS: Confirmatory diagnosis is mainly at operation. In spite of the high incidence of postoperative complication, with correct surgical management the mortality is low and the results are good.

### P182

SPONTANEOUS INTERNAL BILIARY FISTULAS

S. Sekulić, A. Tomanović, B. Daković, M. Filipović, P. Simic, D. Zlta, J. Mladenović

Surgical clinic, KBC, Medical Faculty, Priština, Yugoslavia

The largest number of internal biliary fistulas are a consequennce of penetration of gall calculuses (87,0%) in biliary system, duonenum, stomach or colon. They are formed spontaneously and we can find them very seldom. They are usually discovered by solving biliary calculuses, and go together with clinical description of apsces, icterus, holangitis or sepsis. They ar solved operatively, by reconstruction of biliary pass. At the Surgical Clinic, KBC (Clinical and Hospital Centre), of Medical Faculty in Priština during the period of 1990-1994 there were 10288 operations from which 827 or 8,03% on biliary tract, 131 or 15,8% were male and 696 or 84,2% female. The calculuses of gall-bladder without complications appeared in 529 cases or 63,9%, icterus with holedoholitiasis 74 cases or 8,9%; gangrene of gall-bladder with complications in 80 cases or 9,7%; empiem and hydrops of gall-bladder in 100 cases or 12,1%; holecistopancreatitis in 24 cases or 2,4%; tumour of gall-bladder and pancreas which involved opstruction in 8 cases or 0,97% and internal biliary fistulas in 12 cases or 1,5% (holecisto-colic 1 case or 0,12%; holecisto-gastric 2 cases or 0.24% and holecisto-duodenal 6 cases or 0,72%; biliobiliary 3 cases or 0.36%). During operations on biliary-tract at our clinic died 11 persons or 1,3%. Biliodigestive fistulas were solved as an accessory discovery during the operative treatment of gall calculosis and its complications and plastic of biliary pass, it was made the sewing of duodenum, stomach and colon transversum. By an abdominal drainage, nasogastric suction and corresponding antibiotics and reanimation therapy, thepost-operative course was regular.

### P183

***HORMONAL ASPECTS OF GALLBLADDER STONES***

***Ahmet ÇOKER, Ümit Bayol*, Yıldıray YÜZER, Ali MENTEŞ***

***The HPB Surgery Unit, AegeanUniversity Faculty of Medicine and Department of Pathology*SSK Tepecik Hospital, Izmir, TURKEY***

Thirty-trec patients with gallbladder stones were evaluated prospectively in respect of their hormonal status. Eight male, 25 female patients were included into this study. Serum cholesterol, estradiol and testosteron levels were detected preoperatively in all patients. After a cholecystectomy, pathologic specimes were prepared from the apex(f), body(c) and neck(i) of the gallbladder. Estrogen and progesteron receptor status were determined in all specimens at all three locations stated above. Male and female patients were compared to each other in respect of serum estradiol / serum cholesterol ratios and the reseptor status. Serum estradiol / cholesterol ratio in female and male patients were 0.23 ± 0.09 and 0.34 ± 0.04, respectively. There was no statistical difference between the two group of patients. Estrogen respetor levels were 0.17±0.06(f), 0.17±0.09(c) and 0.28 ± 0.09(I) in females and 0.35±0.10(f), 0.37±0.08(c) and 0.52±0.19(I) in males while progesteron reseptor levels were 0% in all patients. There was a statistically significant difference between these two groups in respect of the estrogen reseptor status ($p<0.05$). We conclude that, a decrease in the sensibility to estrogen reseptors may be responsible for the tendency to cholelithiasis in males.

### P184

**THE INFLUENCE OF TUMOUR TYPE AND DIFFERENTIATION TO THE RESULTS OF CYTOLOGY OF MALIGNANT BILIARY STRICTURES**

S Ahmed, V Distante, TR Kurzawinski, A Deery, JS Dooley, BR Davidson
Hepatobiliary Unit, Royal Free Hospital, London

Exfoliative bile and brush cytology has greatly improved our ability to determine the nature of biliary tract strictures but the sensitivity of these techniques is rarely over 60%**(1)**. This study has analysed the effect of tumour type and differentiation to the results of biliary cytology.
Data was analysed on 79 patients (50 Male, median age 65 years, range 19-85) who had both biliary cytology (92 samples taken at ERCP) and tissue available for routine histopathology. Cytology was reported as positive or negative for malignant cells. Tumour type and differentiation was obtained from histology of resected specimens (n=30), percutaneous or intra-operative biopsy (n=45) or post mortem examination (n=4).
Twenty three patients had pancreatic, 29 bile duct, 20 ampullary and 6 gallbladder cancers. In one case histology showed no evidence of cancer despite a positive cytology. Tumour differentiation was well (n=20), moderate (n=27) and poor (n=21)(1 Ca in situ, 9 differentiation not known). The overall sensitivity of cytology was 55% (43/78) and the positive predictive value of the test was 98%. There was no significant difference in the sensitivity of cytology by tumour differentiation (well 13/20(65%) moderate 14/27(52%) and poor 10/21(48%)). There was, however, a significant difference in the sensitivity of cytology by tumour type being highest for ampullary and bile duct cancers (59 and 80% respectively) and lowest for pancreatic and gallbladder (30 and 50%).
This study has clearly demonstrated the influence of tumour type to the results of biliary cytology but that this is not related to tumour differentiation.
* $p<0.05$, $X^2$ test

1) Kurzawinski T, Davidson BR : Br J Surg 1993 ; 80 : 414-21

### P185

### ROLE OF HORMONES IN CHANGE OF EXOCRINE LIVER FUNCTION AFTER CHOLECYSTECTOMY

R.A.Ivanchenkova, E.P.Gitel
Moscow Medical Academy, Russia

The exocrine liver function after cholecystectomy changes in increasing choleresis (85,7 %) through both bile-acid-dependent and bile-acid-independent fractions. A considerable increase of bile volume has been observed in biliary desease and has been connection with increasing of the secretion in ductules and canalicules.

To investigate the change of bile secretion after cholecystectomy, an attempt has been made to analyze the participation of most known gastrointestinal hormones (glucogon, bombesin, VIP, somatostatin) in regulation of bile-acid-independent fraction. (30 patients with cholelithiasis before and after cholecystectomy, 10 healthy). HCl-stimulator and secretin has been used.

Glucogon, VIP, bombesin influence stimulatory on bile flow of healthy patients. A linear relationship of a high an average correlation has been observed between the blood hormone concentration and the stimulated bile flow. Somatostatin inhibits bile flow. Patients with cholelithiasis have some changes in hormonal regulation: an increase of basal level of bile stimulators (glucogon by 85,8 %, VIP - 27,6 %, bombesin - 33,9 %), a change of character and degree of correlation between the blood hormone concentration and bile flow as well as the character of interactions between hormones. After cholecystectomy they remained. But considering the similar hormone kinetics in patients before and after operation one can assume, that a considerable increase of choleresis after cholecystectomy is determined by local factors (infection, mechanical factor etc.), which change the sensitivity of cells to some hormone action.

### P186

### TRUNCAL VAGOTOMY WITH GASTROJEJUNOSTOMY AFFECTS GALLBLADDER EMPTYING

*Xynos E, Pechlivanides G, Fountos A, Zoras O, Tzovaras G, Chrysos E, Petrakis I, Epanomeritakis E, Koutsoubi E, Vassilakis JS*

Department of General Surgery, University Hospital of Heraklion, Greece

***Background:*** Gallbladder emptying has been studied by scintigraphy after a variety of antiulcer gastric operations, including highly selective vagotomy, truncal vagotomy with pyloroplasty and Billroth I and II gastrectomy.
***The aim*** of the present study was to estimate gallbladder emptying by scintigraphy in patientts with truncal vagotomy and gastrojejunostomy
***Patients-Method:*** In 8 patients after truncal vagotomy with gastrojejunostomy (TV-GJ), gallbladder emptying was estimated by HIDA-scintigraphy. The results were compared to those of 28 healthy controls. Thirty min after i.v. injection of 2 mCi of $^{99m}$Tc-HIDA, an initial abdominal scan was obtain, and then the subjects drank 300 ml of fresh milk (lipids: 4%). Thereafter serial scans of 60 sec, every 5 min and for one hour were taken. By plotting gallbladder radioactivity (measured at all time points and expressed as percentage over the initial count) against time, emptying curves were obtain. From those curves the duration of lag phase, the ejection fraction (peak to least activity) and the pattern of gallbladder emptying were estimated.
***Results:*** In 2 controls radioactivity partitioned into the gallbladder over the abdomen was <25% at the initial view, a phenomenon attributed to spontaneous gallbladder emptying. These subjects were excluded from further assessment. TV-GJ significantly increased the lag phase duration (8±3.5SDmin) and reduced the ejection fraction (50±6SD%) as compared to controls (1.5±4SDmin, p<0.0001 and 81±8SD%, p<0.0001 respectively). All controls exhibited a type I pattern (exponential curve) of gallbaldder emptying. On the contrary, 3 out of the 8 patients after TV-GJ (p<0.03) exhibited a type II pattern (multiple emptying and refilling events) of gallbaldder emptying.
***Conclusions:*** Truncal vagotomy with gastrojejunostomy significantly affects gallbladder motility, by delaying the duaration of lag phase, reducing the extent and altering the pattern of emptying. This could be attributed to the fact that emptying gastric contents bypass the duodenum, after gastrojejunostomy, resulting thus to reduced release of cholecystokinin during the intestinal phase.

### P187

### PREOPERATIVE TRANSHEPATIC DRAINAGE FOR OBSTRUCTIVE JAUNDICE - A PROSPECTIVE STUDY

Wig JD,Gupta NM,Suri S, Kumar H,Khanna SK
Department of Surgery & Radiodiagnosis
Postgraduate Institute of Medical
Education & Research,Chandigarh-160012,India.

Surgery for patients with obstructive jaundice carries formidable morbidity and mortality rates. The role of preoperative percutaneous transhepatic biliary drainage (PTBD) was evaluated in a randomized trial. A total of 40 patients were assigned to either PTBD followed by surgery (Group A,n=20) or elective surgery (Group B, n=20). Mean duration of jaundice in PTBD was 54.5 days and 49.5 days in Group B. PTBD was performed under ultra-sound guidance. The mean duration of drainage was 42.5 days. Both the group were similarly prepared for surgery.
Results : After PTBD significant reduction of hyperbilirubinaemia was observed. Serum bilirubin decreased from 22.24 ± 8.3 mg/dl to 6.75 ± 5.0 mg/dl ($p < 0.05$). Postoperative complications occurred in 5 patients (25%) in PTBD group and in 11 patients (55%) in group B ($p > 0.05$). One patient in PTBD group (5%) and 4 patients in Group B (20%) died (significant at 5% level with probability 0.2). There was significant relief from itching and increase in appetite following drainage. No major complications related to PTBD occurred.
Conclusions : Our observations suggest that preoperative biliary drainage results in significant improvement in liver functions, marked relief from pruritus and reduction in postoperative morbidity and mortality rates.

### P188

### GALLBLADDER MOTILITY IN THE POSTGASTRECTOMY PATIENTS ESTIMATED BY INFUSION CHOLESCINTIGRAPHY;

M.Petrović, V.Artiko, M.Milićević, V.Obradović and K.Kostić

Institute of Nucl. Med., School of Medicine and Clinical Center, Beograd - YU

The aim of the study is the assessment of the gallbladder (GB) contractile function in 8 controls (Cs), and in the postgastrectomy patients: 10 early (EPG) and 10 late (LPG) after total gastrectomy as well as 4 after Billroth I (partial) resection (Bl).

The study was performed during (3 h,10 ml/h) infusion of 150 MBq, 0.25 mg/ml 99m-Tc-EHIDA, preceeded by a loading dose, with two eggs given in 120. min.

In CS, emptying time (ET) was X=37.8min +/- 11.4), ejection fraction (EF) X=76.7% +/- 17.9) and ejection rate (ER) X=2.3%/min+/-0.8. In EPG, ET (X=27.3min +/- 10.9) didn't differ from CS and LPG, while EF (X= 28.7% +/- 10.9) and ER (X= 1.1%/min+/-0.5) were decreased (p< 0.01). However, in LPG, ET (X=39.6min +/-20.0), EF (X= 73.3% +/- 11.3) and ER (X=1.8%/min+/-0.6) didn't differ (p> 0.05) from CS. Also, in Bl, ET (38min+/-8.5), EF (73.7%+/-10.7) and ER (2.02%/min+/-0.22) didn't differ from CS and LPG.

In conclusion, after total gastrectomy with vagotomy and excluded duodenal transit, impairement of the GB motility early and recovery of the physiological contractile function late after operation might be attributed to the establishment of the hormonal mechanisms (CCK). In the patients with partial gastrectomy, without vagal denervation and preserved duodenal tranzit, GB motility remains undisturbed.

### P189

THE DRAINAGE PROCEDURES FOR BENIGN BILIARY TRACT OBSTRUCTION

Michael E.Kontes, P.Gousis,S.Pinis,Ch.Kitsou,G.Androulakis.
4th Surgical Department University of Athens,Greece.

This study deals with the Common bile duct drainage procedures due to benign biliary tract diseases.
We studied 64 patients,30 males,34 females whose ages were ranging from 24 to 91 years (Mean age was 72 years). These patients were treated in the last five years (July 1989-November 1994) in our department.
The first group consisted of 26 patients who underwent an elective operation.The second group consisted of 38 patients who had an urgent operation.
In the first group the indications for operation were: Common bile duct stones, left hepatic duct stones,retaining stones of the Common bile duct and pancreatic pseudocyst.
In the second group the indications for operation were: Obstructive jaundice, suppurative cholangitis,hydrops of the gallbladder and acute cholecystitis.
Fifty of both group patients had a side to side choledohoduodenostomy and the remaining 14 had a Roux en Y choledohojejunostomy.
One patient of the second group had also a transverse colectomy.Three of them had bile leakage and underwent a reoperation.Two patients presented postoperatively evisceration, while 2 more died.As a conclusion drainage procedures of the biliary tract are common and safe.
Morbidity and mortality is dramatically increased when we operate on an emergency basis.

### P190

GALLBLADDER EMPTYING IN PREGNANCY: AN ULTRASONOGRAPHIC STUDY.

S. Bourkov
Sechenov Moscow Medical Academy, Russia

Although the pathogenic role of pregnancy and gallbladder (GB) motility and biliary stasis in gallstone formation has assumed increasing importance only a few studies have analysed GB emptying in pregnant women. The aim of this study was to investigate the GB emptying in pregnancy. GB fasting volume (FV) was measured ultrasonographycaly in 48 healthy nonpregnant women, 120 healthy pregnant women and 146 pregnant women with cholecystities.

The FV was larger in pregnant women with cholecystities in the 3 trimester of pregnancy (54,4 $\pm$ 5,6 ml), then in healthy pregnant women in the 3 trimester (33,7 $\pm$ 4,0 ml) and healthy nonpregnant women (17,2 $\pm$ 5,2 ml). The emptying of GB in pregnant women after fatty meal was retarded in comparison with nonpregnant women, the residual volume increased also, and the percentage of emptying lowered significantly. In parallel with GB contraction the levels of blood progesterone were mesuared and it was seen the straight correlation.

Thus, this data show that pregnancy itself impair GB contractility, and this fact can be a potential pathophysiological risk for gallstone formation in women.

### P191

REGRESSION OF RADIOLOGIC ABNORMALITIES OF PRIMARY SCLEROSING CHOLANGITIS (PSC) IN A YOUNG WOMAN AFTER URSODEOXYCHOLIC ACID THERAPY.

N. Frangiadakis, I.A. Mouzas, P. Skordilis, E. Kouroumalis, M. Koulentaki, O.N. Manousos
Gastroenterology Department, University Hospital, Heraklion, Greece.

Ursodeoxycholic acid (UDCA) has been proven useful in the therapy of PSC, improving symptoms and biochemical abnormalities. There are only few reports about regression of cholangiographic changes (1,2), which are considered irreversible. We report a case in which a 12 month therapy of UDCA resulted in significant regression of radiologic abnormalities.
A 17-yr-old woman presented in June 1992 with severe epigastric pain during the last month. The liver function tests revealed high levels of transaminases (2-4x), alkaline phosphatase (4x) and γ-GT (8x), with normal bilirubin. WBC was elevated (15.500) as well as CRP (3x). On US and CT scan there were no abnormalities. An initial ERCP showed multiple strictures of the intra and extrahepatic biliary ducts with a beaded appearance, consistent with PSC. Oral UDCA at a dosage of 500 mg a day was initiated. The patient was symptom free and the hepatic function tests were normal after one month. On September 1992 an ERCP was performed, which showed a remarkable improvement of the abnormalities of intra- and extrahepatic ducts. Therapy with UDCA was continued for the next 10 months, when the patient, who was now a student, discontinued the treatment. On July 1993 a third ERCP was performed, which showed a complete resolution of the abnormalities in the common bile duct and only slight changes of the intrahepatic ducts. The patient feels well, liver tests remain normal after an aditional 16 months of follow up.
It is concluded that almost total regression of the cholangiographic changes of PSC is possible, at an initial stage, with UDCA therapy.

(1) Lebovics E, Salama M, Elhesseiny A, Rosenthal WS. Resolution of radiographic abnormalities with ursodeoxycholic acid therapy of primary sclerosing cholangitis. Gastroenterology 1992; 102:2143-2147
(2) Springer DJ, Gaing AA, Siegel JH. Radiologic regression of primary sclerosing cholangitis following combination therapy with an endoprosthesis and ursodeoxycholic acid. Am J Gastroenterol 1993;88:1957-1959

### P192

BENIGN OBSTRUCTIVE JAUNDICE

Wig JD,Gupta NM, Katariya RN, Behera A
Department of Surgery
Postgraduate Institute of Medical
Education & Research,Chandigarh-160012,India.

Benign obstructive causes of bile duct other than stone disease or stricture are uncommon. We present our experience with varied aetiologies - Choledochal cyst (n=7), tuberculosis (n=3), fungal granulomas (n=2), intrabiliary hydatid disease (n=8), chronic pancreatitis (n=4), Mirizzis syndrome (n=5), villous adenoma of ampulla (n=2) and idiopathic papillary stenosis in portal hypertension (n=1). Their ages ranged from 15 to 72 years and all were jaundiced at the time of presentation. The various modes of imaging modalities were US,CT,PTC AND ERCP. Preoperative biliary drainage (PTBD) or endoscopic) was instituted in 8 patients. All the patients underwent surgery and an appropriate biliary drainage procedure was performed depending on the pathology. Excision of choledochal cyst was performed in 6 and a cyst-enterostomy in one. Choledochoduodenostomy was performed in patients with common bile duct tuberculosis. Local ampullary excision was performed in patients with ampullary tumours with reconstruction. Three patients (9%) died - two with chronic pancreatitis and one with intrabiliary hydatid disease; the others are alive and well on follow-up. We conclude that these uncommon aetiologies should be kept in mind in managing patients with surgical jaundice and the surgical procedure appropriately planned.

### P193

### THE FOLLOW-UP RESULTS OF CLINICAL COURSE OF PRIMARY SCLEROSING CHOLANGITIS IN ULCERATIVE COLITIS AND CROHN'S DISEASE

G.A.Grigoreva, I.S.Kulazhenkova, D.A.Rudenko
Sechenov Medical Academy, Moscow, Russia

In the series of long-term investigations (1975-1994), carried out at Moscow Medical Academy, in a group of patients with ulcerative colitis (UC-164) and Crohn's disease (CD-97), primary sclerosing cholangitis (PSC) was found in 25 (15.2%) and 13 (13.4%) of patients accordingly, that makes up 14.6% from the total number of patients with inflammatory bowel diseases (IBD). In 10 cases (US-4, CD-6) PSC was diagnosed at the early stage without clinical manifestations of the disease according to biochemical indices proving the initial symptoms of cholestasis. During this observation progressiv PSC has been found in 4 of these patients followed by 2 lethal cases (one patient has got cirrhosis of the liver, the other has developed cholangiocarcinoma). After diagnosing PSC in 28 patients (UC-21, CD-7) they have demonstrated both biochemical indices disordes and pronounced symptoms of the above mentioned disease. By the end of observation 6 patients had died of hepatic failure resulting from PSC followed by cirrhosis of the liver and 1 patient - of cholangiocarcinoma. 9 patients developed slow progressing PSC and transplantation of the liver was performed for two of them; 4 patients have been enlisted and waiting for transplantation. By the end of investigation clinical symptoms of PSC and biochemical indices remained stable in 8 patients. It was impossible to detect distant results in 4 cases.
Thus, in 20 of 38 (53%) cases PSC has been in progress, won the first place in the clinical picture of associated diseases and defined their future prognosis. The results of our investigations are confirming the priority development of PSC in medio-severe and severe forms of UC with total affection of large intestine and chronic continuous inflammatory process there, as well as in permanently active large intestinal forms of CD.

### P194

### ANTIBIOTIC PROPHYLAXIS IN BILIARY SURGERY

L. Papastamatiou, A. Tsaroucha, V. Skountzos, S. Nikolaides, G. Tzagarakis
2nd Dpt of Surgery "Apostle Paul" Hosp-KAT. ATHENS-HELLAS

Although antibiotic prophylaxis is effective in reducing postoperative infections following biliary surgery, there is a controversy on patient selection, antibiotic choice or duration and timing of administration.

This prospective study is based on 1098 consecutive operations for benign biliary diseases. Definition of risk factors for septic complications were determined as follows: age over 65, obesity in relation with diabetes, bile duct exploration and acute cholecystitis. The clinical material was classified in 3 groups: GI:no risk factor (292 cases), GII:1-2 risk factors (469 cases), GIII: 3-4 risk factors (267 cases). Antibiotics used were 2nd generation cephalosporin.

No antibiotic administration to the patients of GI. Perioperative administration of one(preoperative) or three doses to the patients of GII. Administration for 72 hours postoperatively to the patients of GIII. In all cases of acute cholecystitis 3 doses or 3 days administration was applied, in relation to operative findings.

Septic complication rate was 1,7% for GI, 3,2% for GII and 9,4 for GIII.

The data of these series permit the following suggestions:

1) In contrast to recent data, no prophylaxis for GI is needed.
2) Minimal prophylaxis of one single dose, 1 -2 hours prior to surgery is suitable for GII.
3) Maximal 3-days administration for GIII.
4) Acute cholecystitis obliges to 3 doses or 3 days antibiotic prophylaxis. Antibiotic of choice: 2nd generation cephalosporin and only in the presence of inflammatory pancreatic reaction, use of 3rd generation.

It is concluded that this approach to controversial recommendations is hoped to be of medical and economic benefit in biliary surgery.

### P195

### INDICATIONS FOR PERIOPERATIVE CHEMOPROPHYLAXIS IN HEPATOBILLIARY SURGERY

N.Djordjević,G.Stanojević,D.Stanković-Dordević

Interventious on the billiary tract are followed by high frequency postoperative infectious complications,expecialy in patientes with bacteria present in the bile.The incidence of septical complications is reduced by antibiotic prophylaxis in billiary surgery,but it is useful only in patientes with contaminated bile.Intraoperativ gram staining the bile is easy and useful method for determination the presence of bacteria in the bile and for antimicrobial drugs selection for sistemic chemoprophylaxis.This action avoid unnecessary antibiotic aplication,give a chance to make a regular antibiotic choice,reduce the frequence of postoperative septic complications. We avoid unnecessary antibiotic aplication in 52% cases.
Perioperative chemoprophylaxis chould be carried out in the following cases:acute inflamatory pro cesses on billiary tract,early reinterventions, patients with icterus and calculosis of billiary duct and patientes with high risk factors.
By the application of perioperative chemoprophylaxis total frequency of postoperative infectious complications is reduced from 8,91% to 4,91%. In the patients on which billiary-digestive anastomosis is done,this frequency has been reduced from 13,81% to 7,14%.

### P196

### THE STUDY ON THE INTERRELATED ELEMENT CONTENT IN SERUM AND URINE BEFORE AND AFTER OPERATON FOR PATIENTS WITH OBSTRUCTIVE JAUNDICE

Li jin ming,Cheng xiang min,Gao xu chao

Fifth Hospital Zhengzhou,China

The level of Cu,Zn,Fe in serum and urine before and after operation were detected by flame atomic absorption method in 35 case of obstructive jaundice patients which be verified by clinic and operation.The result shouted that:the level of Cu in serum before operation were significantly higher than that after operation($P< 0.001$).The content of Cu in urine there was no obviously difference before and after operation.The level Zn,Fe in serum and urine before operation were noticely lower than that after operation($P< 0.001$).It is suggested that:biliary obstruction,infection the content rised of bilirubine,can lead to abnormal metabolism of Cu,Zn,Fe in serum and urine.But the abnormal metabolism of Cu,Zn,Fe in body can be corrected by operation of biliary drain.This index which be dynamics observed,has a important role in tratment and judged the prognosis for obstrctive jaundice patients.

### P197

Perioperative antibiotic one-shot prophylaxis in laparoscopic cholecystectomy.

Ch.Alexiev; J.Rechner; W.Asperger; M.Meyer

We have performed a prospectiv and consecutiv study. Totally, 560 patients, subjected to laparoscopic cholecystectomy, were allocated into three prophylactic groups. The first group - 400 patients - was prophylactively treatet with Ceftrioxon. In the second group - 90 laparoscopic cholecystectomy - the perioperative prophylaxis was carried out using the antibiotic Cefuroxim. The last serie included 70 patients without any antibiotic prophylaxis.

We analyzed the primar and intraoperative stage, clinical follow-up and the complications of all patients into the three groups. The most seriously inflammation complications were to observe in the group without any perioperativ antibiotic treatment in laparoscopic cholecystectomy.

### P198

**CAN WE USE A NEPHROSCOPE INSTEAD OF A CHOLEDOCHOSCOPE?**

**O. Yagmur, H. Sonmez, A. Alparslan, S. Zeren***
**Dept. of General Surgery and Urology*, Cukurova University School of Medicine, Adana-TURKEY**

**A 35-year old male was admitted our hospital for epigastric and right-upper quadrant pain, fever and jaundice. In his past history, he had a antrectomy and vagotomy operation for duodenal ulcus 8 years ago. His WBC was 13.400/mm$^3$, total bilirubin:2.4mg/dl, direct bilirubin:0.8mg/dl, SGOT:147 iu, SGPT:52 iu, blood amylase:185u, urine amylase:1305u, alkalane phosphatase:545 u. Ultrasonography and CT scan were showed acute cholecystitis with stone and acute pancreatitis. On hospital day 3, he was undergone emergency operation for acute cholangitis. Cholecystectomy and choledochotomy were performed, infected bile and stones were evacuated. Only T-tube was inserted because of severe infection.**
**At the 10$^{th}$ day postoperatively, T-tube cholangiogram showed retained stone into the choledochus. Follow up cholangiogram showed stone again. Postoperatively 45$^{th}$ day, T-tube pulled out and nephroscope inserted into the choledochus via the drain tract under scope guide. Stone was shown and during the manuplation stone passed from oddi sphincter spontaneously.**
**At conclusion, we believe that nephroscope is used to for choledochal exploration effectively as well as a flexible choledochoscope.**

### P199

INTESTINUM OBSTRUCTION FROM GALLSTONE

A.Koussidis, M.Apsokardou, G.Koussidis
Department of Surgery, General Hospital of Chios, Greece.

The aim of this announcement is to present the intestinum obstruction from gallstone because even today it endangers a patient's life due to the at ypical abdominal symptomatology which it presents. Diagnosis is based mainly on clinical symptomatol ogy and on the simple abdominal X-ray after taking a flacon gastrographin.

PATIENTS AND METHODS

In 1993 we operated on a 78 year old male patent for obstruction of the small intestine from a gal lstone. The ultrasount of the gall bladder showed chololithiasis, while the simple abdominal X-ray (in upright position, after taking a flacon gastro graphin) showed obstruction of the small intestine with hydroaero levels. An emergency laparotomy enterotomy was performed as well as the removal of the stone from the small intestine. The enterotomy was closed in two layers. An incision of the gall bladder was done and the stones were removed from the gall bladder. Cholecystostomy was also pe rformed. There was a cholecystoduodenal fistula which was left as it was. The post operative progress was very good. A cholecysto-cholangiography was done through the cholecystostomy-tube on the 20th postoperative day, with a good reproduction of intra and extra hepatic billiary tree. Dischage was given on the 12th postoperative day and the cholecystostomy tube was removed on the 25th post operative day.

CONCLUSION

Intestinum obstruction from gallstone constitutes a rare cause of mechanical obstruction of the small intestine. Diagnosis and indication for surgic therapy must be put forth as soon as possible aft er considering the clinical and labor-findings.

### P200

A GIANT CHOLEDOCHAL CYST CAUSING OBSTRUCTIVE JAUNDICE IN ADULT.

Kafadar Y, Yüceyar S, (*) Aktuğlu Y, Ertürk S.

Department of Surgery, (*) Department of Internal Medicine, I.U. Cerrahpaşa Medical Faculty, Istanbul-TURKEY

Choledochal cyst is a rare congenital malformation of pancreato-biliary ductal system.The incidence ranges from 1 in 13000 to 1 in 2 million live births. Although, it is seen typically in paediatric patients (80 %),the initial clinical presentation in adulthood ( age > 16 years ) occurs in less than 20 % of all patients.

A 17 years old young female patient was admitted to our hospital in September 1994 with a complaint of abdominal pain , mass in the right upper quadrant and jaundice one month after the delivery at 28 th gestational week with severe postpartum bleeding . Preoperative abdominal US demonstrated a Hydatid cyst originated from right lobe of the liver. Serologic tests were negative . In the peroperative exploration,a bile filled cystic lesion in the diameter of 18 x 22 cm. , localized between the right lobe of the liver and duodenum was found. The gallbladder was not identified as well. The nature of the cystic lesion was not related to the hydatid disease. The cyst was extended from bifurcation of right and left hepatic duct to retroduodenal portion of common bile duct and considered to be giant type-1 choledochal cyst according to Todani classification system. The cyst was excised from Hepatic duct bifurcation to retroduodenal portion of choledochus where cyst wall in a diameter of 3 x 3 cm. remained in place . Omega shaped end to side hepaticojejunostomy + side to side jejunojejunostomy was applied to restore the biliary-enteric continuity. The patient has been periodically controlled with uneventfull course.

### P201

ALGORITHM OF A SPECIAL EXAMINATION OF PATIENTS WITH BILIARY TRACT DISEASES

V.Gostischev, A.Vorotyntsev, V.Misnik

Moscow Medical Academy, Moscow, Russia

The present investigation has been conducted to study diagnostic potentialities of combined application of echography, dynamic hepatobiliscintigraphy, ERC for early diagnostics of gallbladder and biliary ducts diseases and to develop a rational surgical treatment strategy. 374 patients have been examined, 90% of them having calculous cholecystitis and its complications. The greatest informativeness of echography in gallbladder calculuses detection and its organic lesions was determined. Gamma-scintigraphy technique is more informative in diagnostics of acute cholecystitis, biliary tract patency disturbance in early, anicteric period. Noninvasive methods permit to determine timely indications for ERC, its accuracy in diagnostics of the cause and the level of biliary tract obturation being 100%. The combined use of the aforementioned techniques made it possible to develop a rational strategy of treating patients.

### P202

CHOLEDOCHAL CYSTS

C. BARGIGGIA, P. MOTTOLA, A. DE MEDICI;

Second Surg. Clin. IRCCS Hosp. S. Matteo

PAVIA ITALY.

Choledochal cysts are uncommon anomalies of the biliary system and probably they are originally congenital. The widespread availability of ultrasound imaging has led to more frequent recognition of this kind of disease. They usually consist in cystic dilatation of the extra biliary tract. This localization and their appearing as cystic or focal dilatation consent to difference them from the post-amoebic or pyogenic abscess which are more frequently intra hepatic. A great part of information about this patology is coming in the last years from Japan, where its incidence is much higher than inoccidental countries. It seems most likely that choledochal cysts are the result of pancreatic reflux into the biliary tree from an anomalous junction of the main pancreatic duct with the common bile duct. The clinical manifestation is most frequently abdominal pain followed by jaundice, abdominal mass and episodes of pancreatitis which are often espression of associated diseases. The syntoms are often expression of associated diseases which are cholangitis or lithiasis in a great part of the cases at the moment of the surgical approach. The surgical treatment generally is performed in the thirth decades of life. Cyst excision and choledocho-enterostomy is the most succesful approach. Endoscopic cyst incision and drainage or transduodenal cyst excision and sphinteroplasty is frequently carried on in some cases. Excellent long term results are provided by reconstruction with Roux-en-y hep-jejunostomy with minimal complications. THe incidence of complications in patients in whom endoscopic incision and drainage is performed is very low. Our experience.

### P203

**GALLBLADDER HISTOLOGIC FINDINGS IN 221 CONSECUTIVE ELECTIVE CHOLECYSTECTOMY**

A.L. Montagnini, J. Jukemura, P.T.H. Gianini, E.E. Abdo, J.E. Monteiro da Cunha, M.C.C. Machado, H.W. Pinotti
Dept. of Gastroenterology São Paulo University Medical School BRAZIL

Cholecystectomy is a very safe procedure and is regarded as the definitive treatment for symptomatic cholelithiasis. Some authors indicate conservative treatment for "high risk" or low symptomatic patients, but these methods leave a diseased gallbladder in place and expose patients to the risk of gallbladder cancer.

The aim of this study is review the type, incidence and distribution of epithelial changes in gallbladder under routine histologic examination.

We reviewed the records of 221 patients submitted to conventional cholecystectomy between march 1987 and march 1992.

RESULTS:

One hundred sixty one patients (72,9%) were women and 60 (27,1%) were men, the median age was 51,5 years (13 to 86) and 48 patients were over 65 years.

| | PATIENTS | | |
|---|---|---|---|
| HISTOLOGY | **AGE < 65** | **AGE ≥ 65** | **TOTAL** |
| **CHRONIC CHOLECISTITIS** | 74 % | 64,6 % | 72 % |
| **ANTRAL TYPE METAPLASIA** | 19,1 % | 16,7 % | 18,5 % |
| **INTESTINAL METAPLASIA** | 2,3 % | 8,3 % | 3,6 % |
| **CARCINOMA** | 0 | 8,3 % | 1,8 % |
| **OTHER** | 4,6 % | 2,1 % | 4,1 % |

DISCUSSION:

Incidence of antral type and intestinal metaplasia were very lower than other reports. We believe it is due to sampling and technique error because histological examinations were not carried out with special mapping technique. As other authors, the global incidence of carcinoma was about 2% and it was present only in patients over 65 yrs.

CONCLUSION: Because of the potentially harmful epithelial lesion found on gallbladder when cholecystolithiasis is present, specially in older patients, conservative treatment should be reserved only for patients who are at high operative risk.

### P204

*LAPAROSCOPIC CHOLECYSTECTOMY ABOLISHES THE POSTOPERATIVE CATABOLIC STRESS RESPONSE.*

*H Heindorff, H. Glerup, A. Flyvbjerg, S. Lindkjær Jensen, H. Vilstrup.*
*Dept. of Medicine V and Surgery L, University Hospital of Aarhus, and Dept. of Surgery C, Rigshospitalet, Denmark.*

*INTRODUCTION: Laparoscopic surgery reduces postop. pulmonary complication, reduces postop. fatigue, and shortens hospital stay. However, the metabolic consequences of lap. surgery are largely unknown. Following open cholecystectomy the efficiency of the liver to convert amino acids to urea is doubled, indicating that liver plays a primary role for postop. loss of body nitrogen. This phenomenon is for the major part mediated by changes in glucagon and cortisol.*
*AIM: To measure the hepatic efficiency of urea synthesis by the Functional Hepatic Nitrogen Clearance (FHNC), and to determine changes in glucagon and cortisol before and after lap. cholecystectomy.*
*PROTOCOL: 8 ptt. undergoing elective lap. cholecystectomy were compared to a historical matched group of 16 patients undergoing open cholecystectomy. Both groups were investigated before surgery and on the first postop. day.*
*RESULTS: Lap. cholecystectomy did not change FHNC (8.8 - 10.7 ml/s, pre/postop., NS), whereas open cholecystectomy doubled it (8.9 - 17.9 ml/s, pre/postop., $p<0.05$). Glucagon and cortisol remained unchanged after lap. cholecystectomy, but increased by 50% ($p<0.05$) and 75% ($p<0.05$) respectively after open cholecystectomy.*
*CONCLUSIONS: Lap. cholecystectomy ameliorates postop. hepatic contribution to postop. N-loss. This is probably due to amelioration of the increase in glucagon and cortisol normally seen after surgery.*

### P205

**CHOLELITHIASIS AND PRECANCEROSIS OR CANCER OF GALLBLADDER**

E.C.Tsimoyiannis, P. Siakas, G. Floras, A. Tassis, M. Tzabarin, M. Handes.
Department of Surgery, G. Hatzikosta General Hospital, Ioannina, Greece.

Intestinal metaplasia and especially dysplasia are predisposing factors of cancer. We study retrospectively the incidence of intestinal metaplasia and dysplasia as well as cancer of the gallbladder in 1416 cholecystectomies for cholelithiasis during the last seven years. In 15 patients (1%) cancer of the gallbladder was found. Mean age of these patients 69.2 ± 7.63 (range 53-79). The incidence of this cancer from 0.87% during the 6th decade of life increases to 4% during the 8th decade (P<0.01). Intestinal metaplasia and/or dysplasia were observed in 74 patients (5.2%) of mean age 60.1 ± 14.8 (range 13-86). The incidence of these predisposing factors of cancer increases from 2.2% during the 4th decade of life to 9.7% during the 8th decade (P<0.005). The total incidence of cancer plus predisposing factors of cancer during the 8th decade of life is 13.7%, while during the 7th and 6th decades is 8.6% and 5.8% respectively (P<0.1, P<0.005 respectively versus 8th decade).
In conclusion, the cholelithiasis coexists with predisposing factors of cancer and cancer of the gallbladder; the incidence of these combinations is rised in the advance of age. This suggests that the early cholecystectomy in patients with cholelithiasis is the treatment of choice to prevent cancer and to treat radically the early cancer as well.

### P206

**CARCINOMA OF THE GALLBLADDER**

B. Paizis, D. Panoussopoulos, B. Pararas, K. Karli, S. Papadopoulos, K. Toutouzas
A' Propaedeutic Surgical Clinic of Athens University.

31 patients with carcinoma of the gallbladder, are presented. All patients were operated in our Clinic, and represent 0,74% of the total number of 4153 cholecystectomies, which performed during the period of 1989-1994.
The female to male ratio, was 22/31 and 9/31 respectively. None of these patients had a preoperative diagnosis of carcinoma of the gall bladder. In 23/31 (74,2%) the carcinoma was found and confirmed intraoperatively, whereas in the rest 8/31 (25,8%) the diagnosis was an incidental finding at histology.
Co-existed cholelithiasis was in 24/31 (77,4%) pts. The surgical procedure which performed, was in 24 (77,4%) cholecystectomy, in 1 (3,2%) atypical Rt hepatic lobectomy, in 3 (9,7%) wedge excision of the gallbladder's bed. "T" tube was placed in 2 (6,4%) and in 1 (3,2%) only exploratory laparotomy.
In 9/31 (29,03%) patients additional operations were performed, namely: gastroenterostomy and Roux-en-Y anastomosis in 5 pts, Rt colectomy in 1, partial small bowel excision in 1 and oophorectomy-omentectomy in 2 pts. The histology of specimens were reported: tubular adenocarcinoma in situ in 1 (3,2%). Regarding the differentiation, in 11 (35,48%) was of low grade, in 16 (51,6%) of moderate and in 3 (9,7%) of high grade. There is no perioperative mortality. The survival figures, were as follows: 22/31 (70,96%) <1 year, 5/31 (16,12%) was 1-2 years, 3/31 (9,7%) was 2-3 years and only one patient 1/31 (3,2%) exceeded 3 years.
In conclusion, carcinoma of the gallbladder is a rare nosological entity with limitations for radical surgical excision (<10%) resulting poor prognosis.

### P207

GALL-BLADDER BENIGN TUMORS

P. Petričević

The Surgical Ward, General Hospital, Yugoslavia

Up to a few years ago we met gall-bladder benign tumors rather rarely because the preoperative diagnostics was not an a high level with us. Since 1990 six patients (3 men and 3 women, whose average age was 57 years) were operated on for gall-bladder polyps. Five patients diagnoscated before the operation, one was operated on as an urgent case, because gall-bladder perforation threatened. Before the operation the diagnosis was made by ultrasonography. None of the patients had gall-bladder calculi. Preoperative discomforts lasted from one month to three years. By gall-bladder exploration the number of polyps varied from 1-3. One of the patients had papiloma with metaplasm, other patients had cholesterol polyps. One woman patient had adenomatous polyp. With all the patients cholecystectomy has been done, but we were careful not to distroy polyps with gall-bladder forceps. Conclusion: thanks to ultrasonographic diagnostics and the doctors experience we can discover gall-bladder tumors, which we remove surgecally to prevent from turning into maligne dysplasia.

### P208

SURGICAL PALLIATION FOR ADVANCED GALLBLADDER CARCINOMA

Gr.Kouraklis,E.Misiakos,G.Eleftheriou,G.Karatzas.
2nd Department of Propedeutic Surgery,Athens University Medical School, Greece.

Primary carcinoma of the gallbladder has a poor prognosis due to its non-specific clinical symptomatology which produces a considerable delay in diagnosis. The records of 38 patients with gallbladder carcinoma operated on between 1971 and 1994 were retrospectively reviewed.There were 26 women and 12 men,with an average age of 66.3 years (range 47-82 years).Clinical symptomatology consisted of abdominal pain, jaundice, nausea, vomiting and weight loss Ultrasonography and computerized tomography were most helpfulf in defining preoperative diagnosis and staging.
In 24 cases (63.2%) associated cholelithiasis was present. The majority of cases were stage III and IV according to TNM system. Surgical procedures included cholecystectomy alone (10 patients), cholecystostomy (5 patients),chlolecystectomy with T-tube insertion (4 patients),chlolecystectomy with hepatic wedge resection (one patient),biliary-enteric bypass with or without gastroenteroanastomosis (6 patients) and exploration with biopsy (12 patients). Operative mortality rate within one month was 21%(8:38). No patient lived more than 2 years; mean survival in our series was calculated as 9.3 months.In conclusion surgical palliation in advanced gallbladder carcinoma has the potential to improve quality of life but offers no significant improvement in survival.

### P209

**HISTOGENESIS OF GALLBLADDER CARCINOMA FROM INVESTIGATION OF METAPLASTIC MUCOSA**

I.Stamenković, V.Katić, A.Nagorni, M.Stojanović

Faculty of Medicine, Niš, Yugoslavia

The histogenesis of gallbladder carcinoma was investigated in association with metaplastic changes in 20 carcinoma.
The microscopical sections from surgical tissue biopsies were stained with:histological (H&E) stain and histochemical (PAS,HID-AB,pH= 2,5) stains.
The gallbladder carcinoma was surrounded by: intestinal,gastric,squamous and pancreatic type metaplasia (98%).The combined metaplasia was observed the most frequent (47%).
The remaining gallbladder carcinoma showed no metaplasia in the surrounding mucosa.
The incidence of metaplasia is in correlation with long-history,type of calculi and multiple cholelithiasis.
From these data it is concluded that every microscopical type of gallbladder carcinoma origined from specific kind of metaplasia.

### P210

**NON FUNCTIONING INSULINOMA OF THE PANCREAS**

G.Belli, A. D'Agostino, I. Marano*, A. Iannelli, G. Mennella, F. Pastena.

Department of General Surgery and Organs Trasplantations.

*Department of Radiologic Sciences.

University of Naples "Federico II" Medical School, Italy.

INTRODUCTION

During the last decade non functioning tumours of the pancreas islet cells have been reported with increasing frequency. These tumours often are discovered because of the "mass effect" that they cause on the near organs. Consequently, when first discovered, they have already reached a considerable size. Here is described the case of a young woman with a non secreting islet cell tumours of the pancreas.

CASE REPORT

A 27 year old woman following a diffuse orticarioid rush was found to have highly platelets count (714000) and reported to the Hematology Department of this University. Here the patient underwent abdominal ultrasonography (US) which revealed the presence of a mass in the head of the pancreas. The patient came to our attention for further investigation. Family history displayed that the patient had 2 sisters. One died of acute cerebrovascular accident during an hypertensive crisis when 10 year old. The other is affected by a surrenalic pheocromocitoma. The physical examination of the patient displayed a mass occuping the right upper quadrant of the abdomen. Routine laboratory tests were normal. Computed Tomography (C T) and Nuclear Magnetic Resonance revealed a solid mass (diam. max 5 cm) in the head of the pancreas. Celiac arteriography demonstrated the presence in the same place of a highly vascularized lesion supplied by the gastroduodenal artery. The mass pushed aside the aorta on the right and the portal and splenic veins upward. Cytologic examination of the specimen obtained through fine needle aspiration biopsy stronghly suggested the presence of a neuroendocrine cell tumour. Serum and urine testing for routine endocrine hormones (including serotonin and 5-hydroxy-indolacetic acid) was normal. The patient underwent a duodenocephalopancreasectomy according to Whipple's procedure. Pathologic examination demonstrated the presence of an insular carcinoma. Immunohystochemical staining was positive for cytocheratine and neuronal specific enolase. No lymphonodal metastases were present. The patient was discharged from the hospital 12 days after operation. A follow up C.T. scan, 6 months after operation, showed no evidence of progressive disease.

Neuroendocrine non secreting tumours are very rare (< 1/ 100000 individuals), but have a better prognosis than their more frequent exocrine counterparts to which they resemble. The attempt to differentiate these two classes of cancer is mandatory. An endocrine tumour would justify an aggressive surgical treatment including a repeted debulking.

### P211

**CYSTIC TUMOURS OF THE PANCREAS: THE IMPORTANCE OF AN ACCURATE DIFFERENTIAL DIAGNOSIS.**

*J.Van de Stadt, M.Delhaye, F.Rickaert, D.Van Gansbeke, M.Cremer, M.Gelin.* Departments of Digestive Surgery, Gastroenterology, Pathology and Radiology, Erasme hospital, Brussels, Belgium.

When the majority of cystic lesions of the pancreas are inflammatory pseudocysts, 10 to 20% consists of tumours with a cystic appearance, that must be correctly diagnosed in order to propose the adequate therapy. Forty-two patients with cystic tumours have been recently followed in our department: serous cystadenoma 4, mucinous cystadenoma or cystadenocarcinoma 12, intraductal mucin-hypersecreting neoplasms (IDMHN) 26. The mean age was 61 years (23-82) and the sex ratio M/F=2/1. Signs and symptomatology were poorly specific. Serum amylase and lipase were usually normal for serous and mucinous tumours, but often increased in IDMHN. CT-scan and ERCP were the most contributive investigations: they precised the general architecture of the tumour, the cyst content and the aspect of pancreatic ducts. Cyst content aspiration during ERCP may be helpful. Thirty-three patients underwent a surgical resection. Radical surgery (Whipple or pylorus-preserving pancreatico-duodenal resection, caudal resection) was performed in all cases of suspected IDMHN and mucinous cystadenocarcinoma. Local or segmental resections were reserved for serous and mucinous cystadenomas. Palliative surgery was the only choice in 3 cases of extended carcinoma. Serous tumours were always benign. Mucinous tumours and IDMHN respectively presented low grade dysplasia in 2 and 8, high grade dysplasia in 2 and 4, in situ malignancy in 0 and 2 and invasive malignancy in 8 and 3. The 1-year survival rate in malignant lesions is 54% (7/13).

*Conclusions:* Cystic tumours of the pancreas are not that rare. Serous cystadenoma may always be considered as benign: it may be treated expectantly. Mucinous cystic tumours and IDMHN are malignant in respectively 50 to 80% and 25 to 50%: radical surgical resection is therefore highly recommended.

### P212

**A RARE NEUROENDOCRINIC TOUMOR OF PANCREAS: PANCREATIC GLUCAGONOMA ( A case report )**

Kakoutis E., Stratis J., Chatzichristou A.,Rafail S., Makrantonakis N.

1st SURGICAL CLINIC, G.R. HOSPITAL "G. PAPANIKOLAOU", THESSALONIKI, GREECE.

Glucagonoma is a solitary and usually large tumor of the pancreas which develops in the alpha cells of the islets of Langerhans and secretes excessive amounts of Glucagon. It causes a distinct syndrome characterized by dermatitis (necrolytic migratory erythema) glucose intolerance, weight loss and anemia.

We report a case of a large glucagonoma (diameter 7cm) in the head of the pancreas in a 52 years old woman without the characteristic necrolytic migratory erythema. The symptoms were epigastric and back pain, moderate diabetes mellitus, weight loss, anemia and duodenal obstruction. The final diagnosis of Pancreatic Glucagonoma was confirmed only after the surgicai resection of the tumor (pancreatoduodenectomy) and the immmunochemical study of the tumor. We discuss our results 32 months after the surgical resection of this neuroendocrine tumor of pancreas.

### P213

### ADENOMA OF THE PANCREATIC DUCT PAPILLA

M.Sever, V.Pegan
Department of Gastroenterological Surgery,
University Medical Center, Ljubljana, Slovenia

In 1994, a 65-year old man was operated on for adenoma of the pancreatic duct papilla, which was diagnosed by ERCP; bioptic and histopathologic diagnosis was: adenoma tubulare gradus III-IV. The patient had semicircural pains in the upper abdomen.
On surgery we found a papillary duct tumor 14x10 mm of size, originating from the pancreatic duct papilla; transduodenal excision of adenoma was done.
Six months later the patient is free of pain, but shows a minor increase in serum amylase.
In the literature we found one article describing villous adenoma excised from the pancreatic duct. Our patient had adenoma located at the pancreatic duct papilla, 15 mm below the papilla Vateri. The authors emphasize the importance of preoperative and intraoperative biopsy with histologic examination to perform adequate surgical procedure.

### P214

### PANCREATICO-DUODENAL ENDOCRINE TUMORS WITH LIVER INVOLVEMENT.

C. Pasquali, C. Sperti, ^ G. Liessi, § P. Gasperoni, S. Pedrazzoli.
Semeiotica Chirurgica - University of Padua, ^ Radiology and § 2nd Dept. of Medicine - Castelfranco V. Hospital, Italy.

From 1970 to 1994, in our Department were observed 128 patients with diagnosis of endocrine tumor of the pancreatico -duodenal area. According to the type of tumor or syndrome at time of presentation the patients were : 63 insulinomas, 51 gastrinomas, 2 glucagonomas, 2 PP-omas, 1 Somatostatinoma, 5 "Non-functioning" and 4 Carcinoids.
Seventeen patients ( 13.3 %) had a liver involvement at time of diagnosis or during the follow-up: 4/ 63 insulinomas (6.3 %), 7/51 gastrinomas (13.7 %), 2 glucagonomas, 2 NF tumors, 1 PP-oma and 1 Carcinoid. Three out of 17 patients with liver tumor had a MEN 1 syndrome (18.7 % of all MEN 1 observed). Two more patients had a multiple hormones secreting tumor that caused an evident second syndrome due to a different active peptide (Insulin + Gastrin and Glucagon than Gastrin respectively). In 5 /17 patients the liver mass was single and the site of the primary was known in 11 cases; in 5 ZES patients the site of the primary was unknown but 2 had diffuse peripancreatic lymphnodes involvement. Two patients had the liver mass detected but the nature (thought to be benign) unrecognized for 3 and 6 years respectively, and 2 patients had liver metastases mistaken for secondary from pancreatic cancer for 1 and 2 years respectively; 3 had distant metastases, outside the abdomen, at time of diagnosis. Six/17 patients underwent resective surgical treatment: 2 right epatectomy and 1 left bisegmetectomy, 2 liver atypical resections + left pancreatectomy; the last patient after DTIC chemotherapy had a left pancreatectomy. Seven out of 17 patients died within 1 year, but 6 lived > 5 years ( range 8-13) after the detection of the liver tumor. No insulin- secreting tumor lived > 18 months. ZES patients showed longer survival (5/7 > 3 yrs.); those who underwent typical liver resection are still living 3, 9 and 10 years after surgery despite recurrence 1,1 and 5 years later. DTIC chemotherapy was effective to control cancer growth in 3 patients who survived 2, 3.5 and 8 years. Due to the slow growing tumor, survival > 5 years is not uncommon. Aggressive surgery helps to improve survival in patients with liver tumor from pancreatico-duodenal apudoma.
Study supported by Italian Ministry of University (MURST) 60%.

### P215

### CYSTIC PANCREATIC TUMOURS

**H.Demiryürek, H.Sönmez, EU.Erkoçak, Ö.Yagmur, O.Demircan, Ö.Alabaz**

**University of Çukurova, Faculty of Medicine, Department of Surgery, ADANA, TÜRKiYE.**

**The purpose of this communication is to present our experience in cystic pancreatic tumours. Seven patients (five women, two men) aged 19-60 years have been studied. Abdominal pain was the main complaint, present in all cases. Diagnostic methods included abdominal US in all cases and CT in six patients. Distal pancreatectomies were performed in all cases. Histologic examination demonstrated one cystadenoma, three cystadenocarcinomas, two serous cystadenomas and one cystic islet cell tumor. A malignant recurrence developed in one case of cystadenocarcinomas, which caused his death 2 years later. Six patients have been remaining well following operation. Where, possible, cystic neoplasm of the pancreas should be completly removed in patients who can be suitable such resection.**

### P216

### TREATMENT OF PANCREATIC CANCER

Emm. Bandouvas, M. Paraskevopoulou, J. Alexandridis, A. Alexandridi, I. Parharidou, A. Tagalos, V. Kolia, Ch. Kountouris, E. Androulakakis, E. Kokinakis, D. Tsabrinou
"Helena Venizelou" Hospital, Athens, Greece

Since 1973 to 1987 patients with pancreatic carcinoma have undergone surgery in our Clinics. Of these, 105 patients resections were performed on 32 (32%); 25 (25%) of whom underwent a curative resection based on macroscopic evidence. Four of whom underwent macroscopic curative resection survived for 6 years, giving a 6-year survival rate 16.5% and 13.5%. Seventy three patients underwent a laparotomy, including biopsy only (n=26). Biliary bypass (n=28), gastric bypass (n=1), biliary and gastric bypass (n=15). There were three treatment groups as treatment policies evolved in both categories (resected and not resected). Initially, patients were observed after surgery without adjuvant treatment (Group 1 in resection category 10 patients and Group 1a in palliative surgery category 23 patients). Patients were offered adjuvant radiation therapy. Postoperatively (Group 2, 10 patients and Group 2a, 23 patients) and Group 3, 10 patients and 3a, 24 patients, received radiotherapy and 5-FU as an in bolus on the first 3 days of the first and fifth weeks of treatment. So, 33 patients were treated with chemosensitized radiation therapy following surgery using 96-hour 5FU infusion during the first and fifth weeks of treatment. There were 5 postoperative deaths which are excluded from the analysis. Among evaluable patients of Groups 1, 2 and 3, local recurrences occurred in 9 of the patients in Group 1, 5 of the patients in Group 2 and 2 in Group 3. The 2-year survival was 33% in Group 1 (3 patients), 40% in Group 2 (4 patients) and 50% (5 patients) in Group 3. Patients with involved surgical margins had a poor survivals only 2 of these 15 patients survived longer than 18 months. Among patients with negative margins, the 2-year survival was 40% in Group 1, 50% in Group 2 and 60% in Group 3. Although the number of patients is small, the 3-year survival was 20% in Group 1, 30% in Group 2 and 50% in Group 3. In palliative surgical treatment Groups 1a, 2a and 3a the median survival was 4 months, 6 months and 10 months.
Conclusion: The results do not support routine prophylactic use of gastrojejunostomy at the time of biliary bypass for patients with unresectable carcinoma of the pancreas. Survival following pancreatic resection is substantially improved with the addition of adjuvant chemosensitized radiation therapy. Our results agree with the international bibliography.

### P217

### P218

### CA 19-9 AND PREDICTION OF UNRESECTABILITY OF PANCREATIC CANCER: COMPARISON WITH CT-SCAN.

C. Sperti, C. Pasquali, ^ R. Polverosi, § C. Lo Giudice, S. Pedrazzoli.
Semeiotica Chirurgica - University of Padua, ^ Radiology - Montebelluna Hosp., § 2nd Dept. Medicine - Castelfranco V. Hosp., Italy.

A correct preoperative staging of pancreatic cancer is needed to avoid unnecessary surgical. exploration. CT scan is the most widely imaging technique used to evaluate tumor's extension. In this study we evaluated the utility of serum CA19-9 assay in combination with CT for staging in 123 patients with histologically proven pancreatic carcinoma, observed from 1986 to 1992. Thirty-five patients were not operated and 88 underwent surgery: 19 radical resections, 21 non-radical resections, 45 biliary and/or digestive by-pass, and 3 exploratory laparotomy. In 15 patients (12 %), CA 19-9 values were < 37 U/ml (cut-off level). In 8 of them CT showed a resectable tumor: 4 patients underwent radical resection and 4 biliary by-pass. In 7 patients CT showed an unresectable tumor:1 had a non-radical resection, 3 by-pass, and 3 were not operated. Thirty patients (24 %) had CA 19-9 values between 38 and 200 U/ml. CT showed a resectable tumor in 22: 12 patients had a radical resetion, 4 a non-radical resection, and 6 by-pass surgery. In the remaining 8 cases, CT showed unresectable tumors: 1 patient had by-pass surgery and 7 were not operated. Seventy-eight patients (63 %) had CA 19-9 levels > 200 U/ml. In 34 cases CT showed a resectable tumor:only 3 patients had radical resection, 13 non-radical resection, 18 by-pass surgery. In the remaining 44 cases CTshowed unresectable tumors: 3 patients had non-radical resection, 16 by-pass surgery, and 25 were not operated.These data strongly suggest that high levels of CA 19-9 (>200 U/ml) are predictive for unresectable pancreatic cancer, and improve CT findings of unresectability.If resection is possible, the tumor is likely to be in advanced stage.

Study supported by Italian National Research Council (CNR), project nr. 93.02236.PF39

### P219

### ENCOURAGING RESULTS FOR PANCREATIC AND PERIAMPULLARY CANCER SURGERY

D. Gakis, S. Rowley, D. Mayer, P. McMaster, J A C Buckels
Liver Unit, Queen Elizabeth Hospital, Birmingham, UK

In the past the results of pancreatic surgery for malignant disease, in nonspecialized centres in the West Midlands, have been poor in terms of perioperative mortality and long term survival.

We report the results of pancreatic surgery in 75 consecutive patients with pancreatic and periampullary malignancy who have had operations in the last 8 years at this unit. Fifty-six (74.6%) of these patients had adenocarcinomas of either the head of the pancreas or ampulla, the remaining had other types of malignant tumours including neuroendocrine tumours and cholangiocarcinomas.

Postoperative 30 day mortality was 2.7% and morbidity was 32%. Reoperation was necessary in 11 patients (14.7%). Actuarial five year survival was 30.6%. There was no difference in 5 year survival between the patients with adenocarcinomas of the head of the pancreas and those with periampullary tumours ($p=0.14$, log rank test). However patients with tumours other than adenocarcinomas had a better outcome ($p=0.039$). Lymph node spread and degree of differentiation were significant determinants of survival ($p<0.05$). The size of tumour, age of the patient and presence of portal vein infiltration had no effect on the outcome. The infiltrated portal vein was replaced with a cryopreserved venous graft in two patients.

We believe that long term results, in a specialized centre, are very encouraging and justify an aggressive approach in a selected group of patients with pancreatic and periampullary tumours. Postoperative 30 day mortality is low but morbidity is still considerable.

### P220

### BILIARY TRACT IN PATIENTS WITH CARCINOMA OF THE HEAD OF THE PANCREAS

*A.Nagomi, T.Tasić, D.Mitrović, S.Petrović-Nagomi, J.Milanović, B.Bajagić, V.Pejčić.*

*Clinic for gastroenterology and Clinic for surgery Faculty of medicine Niš, Yugoslavia*

***

The aim of this study was to evaluate cholangiographic findings in patients with carcinoma of the head of the pancreas. In 32 patients with carcinoma of the head of the pancreas (histologically proved), before surgery ERCP were done. There were 24 male and 8 female, aged 35 to 72, mean age 58.6 years. Biliary tract was visualised in all patients. The common bile duct was dilated in 20 patients (62.5%) with diameter of 10 to 24 mm, mean 16.4 mm. Stenosis of distal common bile duct with dilation in proximal part were obtained in 8 patients (25%), four patients (12.5%) had irregular margins of distal common bile duct with proximal dilation too. In 6 patients (18.7%) stones of the gallbladder were discovered, with common bile duct stones in two patients. Intrahepatic bile ducts were dilated in 28 patients (87.5%) and 3 patients had cholagiographic signs of abscessing.

**Conclusion:** *Cholangiography (ERCP) before surgery for carcinoma of the head of the pancreas routinely would be perform to identify changes in the biliary tract which diagnosed in high percentage.*

### P221

CARCINOMA OF THE AMPULLA OF VATER: SONOGRAPHIC AND CT DIAGNOSIS

C. Schulze
Department of Surgery, University of Brussels, Belgium

Twenty patients with carcinoma of the ampulla of Vater were studied with sonography (n=9) or both sonography and CT (n=11). The tumour was shown by sonography in 16 patients (80%) as a small, round or oval, fairly well delineated mass in between the dilated distal common bile duct and duodenum which was delineated due to luminal fluid or gas (n=13); or as a polypoid mass within the dilated distal common bile duct, resulting in abrupt obstruction (n=3). In then remaining four patients, the mass was not delineated. Bile ducts were dilated down to the level of mass or ampullary region in all cases (100%), while the pancreatic duct was dilated in five cases (45%). We believe that sonography is the technique of initial choice in the diagnosis of carcinoma of the ampulla of Vater as it identifies the mass at the distal end of the dilated common bile duct and/or pancreatic duct.

### P222

**PALLIATIVE GASTROENTEROSTOMY FOR PANCREATIC CANCER**

J.E.M. CUNHA, M. MACHADO, T. BACCHELLA, S. PENTEADO, E.E. ABDO, J. JUKEMURA, H.W. PINOTTI.
Department of Surgery - University of São Paulo, Brazil.

Adenocarcinoma of the pancreas has a very poor prognosis. In approximately 90% of patients palliative treatment is all that can be offered to patients with biliary and/or gastric outlet obstruction. The need for gastric bypass is not as obvious as the need to perform a biliary bypass. The records of 85 patients with unresectable pancreatic cancer treated between 1981-1990 were reviewed to determine whether gastroenterostomy (GE) should be performed profilatically at initial intervention or on a therapeutical basis. Forty-six patients underwent biliary bypass (BBP) alone and on 39 patients a GE was associated with the BBP procedure. There were no statistically significant differences between the two groups as far as age, disease stage and clinical presentation are concerned. The addition of GE to the biliary bypass did not significantly increase perioperative mortality (0% Vs 6.5% in bilioenteric diversion alone), morbidity (58.9% Vs 47.8%) nor length of hospital stay (14.8 Vs 12.8 days). The most common complication of the GE patients was delayed gastric emptying (28.2%). Although the incidence of chronic vomiting was similar in both groups (11.6% Vs 10.2%), no secondary gastroenterostomy was needed in patients submitted to GE as opposed to 9.3% in the biliary bypass group. These results recommend the simultaneous gastroenterostomy at initial intervention because it does not increase morbidity, mortality and length of hospital stay and helps avoiding secondary gastroenterostomy.

### P223

Local resection for the carcinoma of ampulla of Vater

Dianchang Wang ,Tao Sun

Department of Abdominal Oncology,Tianjin Cancer Hospital

There have been 11 cases of carcinoma of ampulla of Vater undergone local resection during May 1985 to May 1989 in Tianjin cancer Hospital.

8 cases have been followed up over 5 years .

Even if the number of the cases is too less to be analysed statistically but there have been 3 cases living more than 5 years without cancer.

The results of these cases supports the conclusion of that local resection in elderly patients with a small tumor is resonable alternative operation to pancreaticoduodenectomy .

### P224

**RADICAL RESECTION OF PANCREATIC ENDOCRINE TUMORS IN PATIENTS WITH MEN1 SYNDROME**

**B. Dousset, O Saint-Marc, O Soubrane, A Louvel, Y Chapuis.**
**(Department of Surgery, Hôpital Cochin, Paris, France)**

The treatment of pancreatic endocrine tumors (PET) in case of multiple endocrine neoplasia type 1 (MEN1) remains controversial, because of multicentric tumors and frequent recurrence following surgery. We reviewed our experience of 6 MEN1-patients who underwent radical resection of PET.

***Methods.*** Between 1973 and 1993, 2 males and 4 females with MEN1 aged 20 to 39 years were referred for PET. There were 4 Zollinger-Ellison syndromes, 1 insulinoma and one non-functional apudoma. Associated endocrine disorders were hyperparathyroidism (n=5), pituitary adenoma (n=4) and adrenal adenoma (n=3). Hormonal measurements demonstrated hypergastrinemia (n=5), hyperinsulinemia (n=1) and normal hormonal profile (n=1). Imaging studies included ultrasonography (n=6), computed tomography (n=6), and endoscopic ultrasonography in the last three patients. Indications for surgery were a tumor > 15 mm (n=3), uncontrolled Zollinger-Ellison syndrome (n=2) and severe hypoglycemia (n=1).

***Results.*** Surgical exploration disclosed 4 to 9 PET ranging from 4 to 30 mm. Preoperative imaging work-up underestimated the number of lesions in all instances. The removal of all macroscopic tumors led to left (n=3), subtotal (n=2) or total (n=1) pancreatectomy. There was no mortality. Complications included one post-operative diabetes (after total pancreatectomy) and one splenic infarct. Immunohistochemical study showed : multiple gastrinomas (n=4) associated with duodenal microgastrinoma and lymph node metastases in one case, insulinoma (n=1) associated with malignant gastrinoma (positive nodes), non-functional apudoma (n=1). In both cases of malignant tumor, the largest gastrinoma was less than 15 mm in size. Four patients including the two with lymph node metastases had no evidence of tumor or hormonal recurrence 1 to 13 years after surgery. 2 patients developed hypergastrinemia (1 recurrence, 1 de novo) 1 and 3 years after surgery without detectable tumor on imaging studies. Both are alive with stable clinical condition.

***Conclusions.*** 1) Radical pancreatic resection in MEN1-patients can achieve a prolonged disease-free survival with a low incidence of post-operative diabetes. 2) The low accuracy of preoperative imaging studies and the risk of malignancy even in small tumors gives further support to an aggressive surgical approach.

### P225

### FACTORS ACCOUNTING FOR PROGNOSTIC DIFFERENCES IN PERIAMPULLARY TUMORS

J.D. Roder, P.M. Schneider, H.J. Stein, J.R. Siewert.
Department of Surgery, Technische Universität München, Germany

Despite a close spatial relationship, the various types of adenocarcinoma of the periampullary region (i.e. carcinoma of the pancreatic head, papilla vateri, or distal bile duct) have a distinctly different prognosis after surgical resection. We attempted to identify factors which may account for these prognostic differences.
Patients and Methods: Prospectively documented tumor and patient dependent factors were analyzed in a total of 194 patients who had a partial duodeno-pancreatectomy for adenocarcinoma of the pancreatic head (N=90), papilla vateri (N=66) or distal bile duct (N=38) at our institution between 1983 and 1994. Median follow up is 42 months.
Results: Age and sex distribution, postoperative mortality, and tumor grading was not different between the patient groups. There were, however, marked differences in tumor size, rate of perineural invasion, the rate of node negative patients (pN0), UICC stage distribution, rate of complete tumor removal (R0-resection) and survival (see table).

| *Location of the Carcinoma* | *Pancreatic Head* | *Distal Bile Duct* | *Papilla Vateri* | *p-value* |
|---|---|---|---|---|
| Mean Tumor Size (mm) | 34.8±11.0 | 22.0±13.6 | 27.4±19.3 | 0.05 |
| Perineural Invasion | 43.3% | 34.2% | 16.7% | 0.002 |
| pN0 | 31.1% | 42.0% | 57.6% | 0.05 |
| UICC Stages I/II | 32.2% | 13.2% | 57.5% | 0.001 |
| R0-resections | 43.3% | 68.0% | 92.4% | 0.05 |
| Median Survival | 12 months | 13 months | 41 months | 0.001 |
| 5-Year Survival | 9.6% | 17.1% | 34.6% | 0.001 |

Conclusion: Compared to patients with adenocarcinoma of the pancreatic head or distal bile duct, patients with carcinoma of the papilla vateri undergoing resection have a lower rate of perineural invasion, a higher node negative rate, and are diagnosed at an earlier stage. This results in a higher rate of complete tumor resections (R0-resections) and a markedly better survival.

### P226

### ADENOCARCINOMA OF THE PANCREAS: LONG-TERM SURVIVORS.

C. Sperti, C. Pasquali, C. Militello, S. Pedrazzoli.
Semeiotica Chirurgica - University of Padua, Padova, Italy.

Long-term survival in patients with a proven histologically diagnosis of pancreatic carcinoma is virtually confined to those undergoing tumor resection. However, there is some doubt as to the number of long-term cures that result. The purpose of this study was to analyze the patients who survived 5 years or longer after operation for exocrine pancreatic cancer, and to determine factors that may have influenced the favorable outcome. From 1970 to 1992 a total of 536 patients with carcinoma of the pancreas were seen in our Department. There were 11 putative 5-year survivors: 2 after by-pass surgery and 9 after radical resection. Pathologic review confirmed primary carcinoma of the pancreas in 9 patients (all those resected) with a real 5-year survival rate of 1.6% (8% of 111 resected patients). The histologically proven survivors included 8 ductal and 1 acinar cell carcinoma. Four tumors were located in the head, 3 in the tail, and 2 in the whole pancreas. Only 3 tumors were < 2 cm in diameter. In 3 cases, the tumor involved the duodenal wall, and 1 tumor extended into peripancreatic fat. Histologically 7 were well,1 moderately and 1 poorly differentiated adenocarcinoma. Six showed lymphatic invasion, which was perineural in 3 cases.No patient had lymph node metastases. Two patients died of their disease 7 and 8 years after surgery, respectively, with local and/or hepatic recurrence. Seven patients are alive and disease-free from 6 to 15 years. Six long-term survivors were operated until 1980, and 3 after this period.
In conclusion, long-term survival in histologically confirmed pancreatic carcinoma is a rare event. Only few patients without lymphnode metastases are suitable for a favorable prognosis. Late tumor relapse is a possible event; so, 5-year survival is not a guarantee of cure for pancreatic cancer.

Study supported by Italian National Research Council (CNR), project nr. 93.02236.PF39.

### P227

### CARCINOMA OF PANCREAS SURGICAL TREATMENT

Z.Wajda, Z.Gruca, Z.Sledziński, T.Wysocki

II Department of Surgery, Medical University of Gdańsk

Poland

185 patients suffering from pancreatic cancer were treated in our institution from 1970-1993. The radical operation rate achieved 18,3%, 34 curative procedures were performed in 22 patients with malignancy localised in the head of pancreas; in 9 with the tumour in periampullary region, and in 3 patients with the cancer of the body of pancreas. Indication for radical surgery were local resectability of lesion and regional lymph nodes free from metastases proved by histology. The paliative operation were carried out in 107 patients; in 44 explorative laparotomies only were performed. The perioperative mortality rate due to radical treatment revealed to be 14,7%; 13% of paliative treatment and 5,6% as consequences of the explorative laparotomy. late results of surgical treatment of pancreatic cancer were as follows; laparotomy - median survival time 2,5 months; paliative procedures 6 months but after radical treatment survival was significantly better more than 17 months and differs due to the tumour localisation. Long term survival was better in periampullary cancer 22 months comparing to the carcinoma of pancreas - 13 months. The longest survival /7,5 years/ was observed in one patient with periampular cancer. Conclusion: results of surgical treatment of pancreatic cancer are still unsatisfactory mainly due to the advenced stage which excludes radical procedure.

### P228

### PANCREATIC NEOPLASMS AND TUMOR MARKERS. BRAZILIAN EXPERIENCE

A.L. Montagnini, S.R.B. Natal, L. Simon, M.S. Kubrusly, S. Penteado, J. Jukemura, M.C.C. Machado
Dept. of Gastroenterology São Paulo University Medical School BRAZIL

Because of absence of specific symptoms in early stages of the disease, pancreatic cancer diagnosis is done more often when ressecability rates are about 10 to 20%. Tumor markers for pancreatic cancer could help in early diagnosis.
This study was carried out to review the sensitivity and specificity of tumor markers (CEA, CA 19-9 and CA 72-4) for pancreatic cancer diagnosis in our institution.
Sixty patients with pancreatic cancer and 33 patients with benign disease were studied. Tumor markers were measured by radioimmunoassay (CIS bio international).
RESULTS
In pancreatic cancer group, 36 were men and 24 were women, the mean age was 61,5 years (39 to 83), curative resection were possible in only 11%. In benign disease group 23 were men and 10 were women and the mean age was 47,3 years (20 to 87).

| | CEA | | CA 19-9 | | CA 72-4 | |
|---|---|---|---|---|---|---|
| | ≤10ng/ml | >10ng/ml | ≤36 U/ml | >36 U/ml | ≤ 3 U/ml | > 3 U/ml |
| CANCER GROUP | 34 / 56 | 22 / 56 | 11 / 57 | 46 / 57 | 33 / 53 | 20 / 53 |
| BENIGN DISEASE | 32 / 32 | 0 | 24 / 32 | 8 / 32 | 31 / 32 | 1 / 32 |

If we change the cut-off level for CA 19-9 to 100 U/ml, sensitivity drops from 81% to 69 % but specificity raises from 85% to 97,8%.
If we consider all markers together and at least one positive, sensibility is 78% and specificity is 97,8%.

CONCLUSION: Association of these 3 tumor markers and cut-off level for CA 19-9 at 100 U/ml allow good sensitivity and excellent specificity, and should be used when pancreatic tumor is suspected.

### P229

SURGICAL MANAGEMENT OF TUMORS OF THE AMPULLA OF VATER

B. Chareton, G. Spiliopoulos, JP. Campion, J. Terblanche, B. Launois
Department of Digestive Surgery and Transplant Unit, Hopital Pontchaillou, CHU de Rennes 35033, France

**MATERIALS AND METHODS**

Between 1970 and 1992, 63 patients underwent surgery for ampullary tumors. The group comprised 33 males and 30 females with a mean age of 64.8 ± 9.8 years. Surgical procedures included subtotal duodenopancreatectomies (n = 40), total pancreatectomies (n = 3), ampullectomies (n = 8) and surgical bypass or exploratory laparotomies (n = 12). Resectability was 68%. Pathology included 53 adenocarcinomas, 1 undifferentiated lesion and 9 benign lesions. According to the MARTIN staging criteria tumors were classed as follows: stage I = 7, stage II = 11, stage III = 14, stage IV = 21. All patients with stage I, II and III tumors underwent resection. Among the stage IV patients, 11 were resected and 10 had bypass procedures.

**RESULTS**

Mean hospital stay was 20.6 days. For the patients having undergone subtotal duodenopancreatectomies mean time of stay was 24.8 days (16.5 days when the postoperative course was uncomplicated). Overall operative mortality was 12.7%, and 7.5% after subtotal duodenopancreatectomy. Five-year survival for the entire group was 40%. Five-year survival for stage I through IV tumors was 85%, 65%, 44%, and 8% respectively. For stage I, II, and III lesions, survival was significantly better following subtotal duodenopancreatectomy than after ampullectomy. For stage IV lesions, 1 and 2-year survival following subtotal duodenopancreatectomy and surgical bypass was 70% and 25%, 20% and 0% respectively. We now consider subtotal duodenopancreatectomy rather than amullectomy as the treatment of choice for benign ampullary lesions, having reoperated on two patients with stage IV tumors who had undergone ampullectomy for benign lesions, 4 and 22 years previously.

**CONCLUSIONS**

Subtotal duodenopancreatectomy is the treatment of choice for ampullary tumors, even when these are benign.

### P230

THE SURGICAL PALIATION IN PANCREATIC TUMORS : SURVIVAL AVERAGE

Turienzo S.,E; González Martínez, J.

Servicio de Cirugia I-A ; Hosp. Central Asturias- España

The pancreatic cancer is nowadays a disease which treatment is not effective in the majority of the cases, so surgical techniques are neccesary in order to make better the life s quality of the patients;these tecniques can, also, increase the time of life.

With the intention of knowing the time of survival in the patients with pancreatic cancer who underwent the surgical paliation, we have done a analysis about our patients.

Between 1.973-1.993, we have attended 559 patients. Only the 14 % underwent the surgical exeresis.

In 42 cases (7.4 %) that were undergone surgery it was not possible doing anything and the survival average was 64 days.

In 325 cases (59 %) it was only done surgical paliation. The post-operative mortality in this group was 23 % . Among the rest of patients of this group, we have analized 100 of them whose date of death was known.The survival average was 172 days. At the six and twelve months lived the 48.3 % and 12.2 % . At the two years all the patients were died.

CONCLUSIONS: The surgical paliation increases the time of life in 3.6 months respect to those patients in which it was not possible the surgery.

### P231

SURGERY OF PANCREATIC AND PERIAMPULLARY CARCİNOMA

E. Sözüer, N. Akyürek, Z. Yılmaz, M. Akpınar, Y. Yeşilkaya

Üniversity of Erciyes, Medical School

Turkey.

Between 1980 and 1994, 100 patients had operation for adenocarcinoma of the head of the pancreas(n:94), ampulla(n:5) and papillary adenom(n:1). 69 patients were men, and 31 were women, and the mean age was 59.8 (range:30-78). The patients were divided into two groups on the basis of two different time periods: those operated on from 1980 to 1990 (n:55) and those operated on from 1991 to 1994 (n:45). The rates of resection in the first and second group were % 3 and % 19 respectively. Hospital morbidity rate was 11%. Hospital mortality rate was 66% operated on during the first period and were 16.6% operated on during the second period. We had no patients who survived for five years. However, the importance of Whipple procedure reveals itself when the results of the second group are examined.

### P232

CURATIVE SURGICAL TREATMENT OF HEPATIC METASTASIS FROM COLORECTAL CANCER.

J Carles, G Reboul, P Bernard, E Rullier, M Caudry, J Saric.

Chirurgie Viscérale, Hopital Pellegrin, Bordeaux, France.

We studied different factors predicting long term survival.

From October 1985 to December 1993, 133 curative hepatectomies for hepatic metastasis from colorectal cancer were performed. It was an adenocarcinoma in all cases. The surgical procedures consisted in 98 major hepatectomies, 12 minor hepatectomies and 39 tumorectomies. The metastasis was unique in 73 cases and more than 3 in 13 cases. They were synchronous in 37 cases and metachronous in 89 cases. The average time of appearance was 18 months ( 0 to 120 months ). The diameter of lesions was less than 50 mm in 73 cases. Fifty four patients were treated with chemotherapy based on FUFOL (48 in the last 3 years ) .

The postoperative mortality was 1,58 % ( 2 patients ). Sixty nine patients were alive with an average follow up of 23,5 months. The actuarial survival rate is respectively at 1, 3 and 5 years of 84,5 %, 50,7 % and 25,3 %. Seventy three patients presented a recurrence in wich 49 in the liver with an average time appareance of 12 months. Forty nine patients were alive without any recurrence.

Among the factors predicting long term survival ( CEA, Dukes stage, minor or major hepatectomy, number and diameter of metastasis and resection margin more than 10 mm ), the only one which was statistically significant was the major hepatectomy.

Nevertheless, a survival rate of 25 % at 5 years confirms that a complete surgical resection , when possible, is the treatment of choice for hepatic metastasis from colorectal cancer.

### P233

### COLORECTAL CARCINOMA METASTASIS TO THE CAUDATE LOBE OF THE LIVER - REPORT OF TWO CASES -

E. M. Gadžijev, D. Stanisavljević, M. Matič
Department of Gastroenterologic Surgery, University Medical Center, Ljubljana, Slovenia

Background. In patient with colorectal carcinoma liver metastases are expected to develop in 40 - 50%. Metastases to the first liver segment (Caudate lobe) are rare, their finding and location is possible using CT or MR investigation but surgical treatment is challenging and demanding.
Methods and results. Two patients with metastases in caudate lobe are presented. The history of the disease, diagnostic procedures and treatment are described. The first patient with colorectal metastases to the liver had been operated with right hepatectomy 20 month prior to the second operation when the caudate lobe was resected. In the second patient the metastasis to the first liver segment was found three years after resection of rectosygmoid colon and the caudate lobe removed. Both patient had been given chemotherapy after resection of the primary cancer, but before and after liver resection no chemotherapy was used. No blood replacement was needed and in both cases the postoperative course was uneventful.
Conclusions. With careful follow of the patient operated for colorectal carcinoma, metastases to the liver could be discovered soon enough sometimes to perform radical liver resection. Resections of the caudate lobe are still challenging procedures demanding excellent knowledge of liver anatomy.

### P234

### SURGERY OF LIVER TUMOURS WITH SPECIAL REFERENCE TO MAJOR RESECTIONS

Kupcsulik,P.K., L.Harsányi, K.Darvas,K. Pinkola, L.Flautner
1st Dept. of Surgery, Semmelweis Univ. of Medicine, Budapest

From 1989 to 1994, 401 patients underwent surgery for liver tumour. 172 interventions were performed for liver malignancy,partly because of metastatic origine. Types of surgical procedures are listed on table see below. Out of 68 cases with progressive liver malignancy only explorative laparotomy or biopsy was performed, and 21 of them were lost postoperatively. 160 surgical intervention resulted in removal of tumorous mass. Lethality of this group was 3.1 percent, respectively. Liver cirrhosis represents premalignant condition, therefore malignant tumors of the cirrhotic organ require special interest. 12 patients with liver cirrhosis and liver cell carcinoma underwent hepatic resection. One death occurred as a consequence of liver insufficiency.
The use of ultrasonic dissector, or its combination with partial or total liver exclusion diminishes intraoperative blood loss, and promotes extended resection of the liver.

| | malignant | + | benign | + |
|---|---|---|---|---|
| trisegmentectomy | 32 | 2 | 5 | 0 |
| right lobectomy | 18 | 1 | 6 | 0 |
| left lobectomy | 26 | 0 | 15 | 0 |
| segmentectomy | 84 | 2 | 112 | 0 |
| expl.lap. | 68 | 2 | 118 | 0 |
| fenestration | - | - | 17 | 0 |

### P235

### EXTRAHEPATIC DISEASE AS BAD PROGNOSTIC FACTOR AFTER RESECTION OF COLORECTAL LIVER METASTASIS.

Murio, J.E., Balsells, J, Charco, R., Lazaro, J.L., Bilbao, I., Gifre, E., Ruiz, C., Margarit, C.
Liver Transplant Unit. Hospital General Universitario Vall d'Hebron. Barcelona, SPAIN.

Between 1987 and 1993 we have operated on 28 patients with liver metastasis from colorectal cancer. Patients' mean age was 58 years (15 males, 13 females). Primary tumor was located in colon in 17 cases and in rectum in 11; local Duke's stage was 1A, 7B, and 20 C. Secondary tumor was synchronic in 12 cases and metachronic in 16. Nineteen major and 9 minor hepatectomies were performed.In 9 cases extrahepatic disease was found during the operative procedure (32%). Adjuvant chemotherapy was administered to 13 patients. The 1, 2 and 3 years survival rate after liver resection was 72%, 44% and 22% respectively. One patient died during postoperatory, 16 for tumor recurrence, 2 for unknown cause and 1 for brain seizure.
We analyzed the following risk factors: age, sex, primary site, free interval, number of metastases, size of metastases, CEA, type of hepatectomy, adjuvant chemotherapy and extrahepatic disease (hepatic lymph nodes, local recurrence and parietal infiltration). In our serie only the presence of extrahepatic disease was a statistically significat survival prognostic factor after liver resection for colorectal metastasis ($p< 0.02$). There was a trend to poor prognosis when primary tumor was advanced. Adjuvant chemotherapy seemed to improve the disease free survival without effect on overall survival. Eventually we should comment that in this serie survival was affected by the high incidence of extrahepatic disease, particularly when compared with other reported series.
In summary, extrahepatic disease is associated with bad survival after resection of colorectal liver metastases and preoperatory work-up should be directed to exclude this condition.

### P236

### SYSTEMIC CHEMOTHERAPY IN METASTATIC COLORECTAL CANCER.

Ciferri E., Gazzaniga G.M., Aschele C.*
1st Surgery Dept. - S. Martino Hosp.- Genoa - Italy
*1st Dept. of Medical Oncology IST - Genoa - Italy

On the basis of recent demonstrations in vitro of two possible mechanisms of action and of induced resistance depending on the dosage and schedule of FUra administration, we submitted twelve patients with multiple liver metastases from colorectal cancer and all considered unresectable and without any sign of recurrence to chemotherapy, in collaboration with the 1st Dept. of Medical Oncology IST of Genoa, from August 1993 to September 1994. According to this rationale we began a phase II trial of schedule-oriented biochemical modulation of FUra bolus by MTX and β Interferon, and FUra continuous infusion by Leucovorin. In particular, the treatment schedule provided a hybrid regimen of two biweekly administrations of 600 mg/sqm of FUra bolus, which had to be preceded the day before by 200 mg/sqm of methotrexate, and had to be followed, the same day and the next day, by two administrations of 3 x $10^6$ β-Interferon i. m.; after an interval of two weeks, the cycle arried on with three weeks of continuous infusion of 200 mg/sqm per day of FUra, which was preceded every first day of the week by an administration of 20mg/sqm of Leucoverin bolus.The entire cycle was repeated after a week of rest, having first evaluated the lesions through US/CT/MNR scans and plotted the percent change of total measured tumour mass and dosed tumour markers.All the twelve patients, with no pre-chemotherapy, had the primary colorectal tumor mass resected for necessity and their livers were considered unresectable for the characteristics of the hepatic metastases: their number, dimensions, contiguity/continuity with important vascular structures didn't allow a radical operation.Their Performance Status was 0 and average age 63 (range 54 - 82).Eleven patients have completed at least one cycle of the treatment and have been re-evaluated, while one patient has just been included in the study; we have obtained two complete responses, after six months of chemotherapy, and, at the moment, also seven partial responses (75% of all); two patients died after 8 months because of the advancement of their illness.We have had one death due to toxicity after the first administration of FUra, MTX and β Interferon in the first cycle, and two cases of III grade toxicity (diarrhoea and mucositis).The two patients that had a complete response to the chemotherapy was submitted to a second surgical look, during which two hepatic metastasis, that had reached dimensions of 2 cm in diameter, were resected in each one, after a previous ultrasonography controll.

### P237

### INTRA-ARTERIAL CHEMOTHERAPY FOR IRRESECTABLE HEPATIC TUMORS

L.J.A. STROBBE, E.D.M. BRUGGINK, J. VAN TURNHOUT
Depts of Surgery and Medical Oncology, Canisius Wilhelmina Ziekenhuis, Nijmegen, The Netherlands

**purpose**: Identify which patients with irresectable liver tumors can benefit from intra-arterial chemotherapy and what complications can be expected.
**methods**: Twenty-eight patients underwent operative installation of an intra-arterial port device for regional liver perfusion. Twenty patients had metastases from colorectal primaries, three adenocarcinoma of the breast, one carcinoid, one apudoma, one leiomyosarcoma and two primary liver tumors. All tumors were irresectable. All patients received celiac and mesenteric angiography; 25% showed relevant arterial variations.
**results**: The carcinoid patient has been "cured" with 6 5-Fluoro-Uracil infusions, she is now 8 years disease-free. The overall response rate is 78%; response rate for the metastases from colorectal origin is 90% and 0% for the hepatic primaries. When the colorectal primaries are subdivided according to the Astler-Coller-Dukes classification, median survival after detection of liver metastases is 19,2 months for the B II-stage tumors and 12 months for the C II-stage tumors. A relation between alkaline phosphatase (AF) level and survival as well as between tumor differentiation and survival are demonstrated.
We did not find a relation between the extent of liver tumor replacement and survival. Median remission duration as demonstrated by sonography, AF and carcinoembryonal antigen is 8,5 months for B II-stage tumors, 2,8 months for C II-stage tumors. Catheter-related problems occured eight times, necessitating operative revision in four cases.In five patients we had to discontinue perfusion because of systemic toxicity.
**conclusions**: The palliation we intend to achieve is worth the effort in the patient subgroup whose primary tumor was a B II colo-rectal carcinoma. Carcinoid tumor metastatic to the liver can be palliated for a prolonged time using regional liver perfusion therapy.

### P238

### HIGH PREOPERATIVE SERUM AMINOTRANSFERASE LEVEL INCREASES THE RISK OF RESECTION OF HEPATOCELLULAR CARCINOMA (HCC) IN CIRRHOTIC PATIENTS WITH PRESERVED HEPATIC FUNCTION.

P. Jagot, R. Noun, A. Sauvanet, J. Belghiti.
Department of Digestive Surgery, Hôpital Beaujon, F - 92110 Clichy.

Surgical resection of HCC in cirrhotic patients is associated with an operative mortality above 5%, even in those with a preserved hepatic function.The aim of this study was to define in an homogenous group of cirrhotics with good hepatic function (i.e Child-Pugh A) undergoing resection for HCC, the factors correlated with operative morbidity and mortality.
Patients and Methods : From 1981 to 1994, among 152 patients resected for HCC, 108 were Child-Pugh A. There were 88 men and 20 women with a mean age of 58±11 years. Preoperatively, mean Prothrombin Time (PT) was 81±11%, mean albuminemia level was 43±7g/L and mean bilirubinemia level 15±9µmol/L. In 31 (29%) patients, the preoperative serum aminotransferase ALT level was above 2N (N<40IU/L). Forty-three (39%) patients underwent major resection. Portal triad clamping was performed in 85 (79%) patients with a mean duration of 30±14 min. Fifty-seven (53%) patients did not require any operative blood tranfusion, 28 received 1 to 5 pack red cells units (PRCU) and 23 more than 5 PRCU.
Results : Major complications occurred in 32 (29%) patients and 9 (8.3%) patients died postoperatively. In a multivariate analysis (Logistic regression), operative mortality was correlated with preoperative ALT serum level (p=0.005) and with the number of PRCU operatively transfused (p=0.002). There was a significant raise in mortality and morbidity if preoperative ALT was above 2N.

| | Death | Ascitis | Renal failure | PT <40% at day 1 |
|---|---|---|---|---|
| | n=9 | n=57 | n=5 | n=34 |
| ALT≤2N (n=77) | 2(1,2%) | 36(47%) | 0 | 25(32%) |
| ALT>2N (n=31) | 7(22%) | 2(68%) | 5(16%) | 9(29%) |

Conclusion : Operative risk of Child-Pugh A cirrhotic patients is lower if preoperative ALT is below 2N. When ALT is above 2N, these results suggest that the surgical procedure should be delayed.

### P239

### PORTAL PERFUSION ASSESSMENT IN CIRRHOSIS AND LIVER TUMOURS BY HEPATIC RADIONUCLIDE ANGIOGRAPHY

V. Artiko, K.Kostić, M.Perišić-Savić and S. Janošević. Institute of Nuclear Medicine, Medical School and Clinical Center, Beograd - YU

The aim of the study is the examination of the relative portal blood flow, by assessment of the hepatic perfusion index (HPI) in different degrees of hemodynamic alterations related to liver cirrhosis and some focal liver diseases. Hepatic radionuclide angiography (HRA) was performed with bolus injection of 740 MBq- 99m-Tc-pertechnetate, during one minut (1f/sec), using ROTA scintillation camera and Micro Delta computer (Siemens). HPI was estimated using Sarper's method of slope analysis.
In 10 controls (C), HPI was 0.68+/-0.06; it was significantly decreased (p<0.01) in 5 patients with chronic active hepatitis (HAH, 0.57+/-0.03), 13 with liver cirrhosis without (LC, X=0.49 +/-0.13) and 18 with esophageal varices (LCEV, X=0.32+/-0.19), as well as in 4 patients with LC and sclerosated esophageal varices (LCSEV, X=0.16+/-0.11). Comparing to HAH and LC (HAH-LC, p>0.05), HPI values were significantly lower in LCEV (p<0.01) and LCSEV (p<0.05), while the values between the last two groups didn't differ (p> 0.05).
In 22 patients with liver hemangiomas (LH, X= 0.64 +/-0.08) HPI values were physiological (C-LH, p>0.05). However, in 4 patients with hepatocellular carcinoma (H, X=0.26+/-0.20), and 8 with liver metastases (LM, X=0.40 +/-0.28), HPI values were significantly decreased (p<0.01), but they didn't differ between themselves (H-LM, p>0.05).
Portal liver perfusion decreases in respect to the portal hypertension and collateral circulation development, while after sclerotherapy, it remains very low. Considering the HPI values obtained in liver tumours, HRA is an useful method for the differential diagnosis of hemangiomas and primary liver carcinomas, together with ultrasonography and blood pool scintigraphy.

### P240

### SURGICAL TREATMENT OF HEPATIC METASTASES FROM COLORECTAL CANCER : A COMPARATIVE STUDY BETWEEN "CONVENTIONAL" AND POSTERIOR APROACH TECHNIQUE - PRELIMINARY REPORT

G. SPILIOPOULOS, B. MEUNIER, M.A.C. MACHADO, C. STASIK, F. GONZALEZ, B. LAUNOIS.
Department of Surgery and Transplantation Unit. University of Rennes - France.

Surgical resection still represents the best chance of improving survival for some patients with hepatic metastases of colorectal origin.
The aim of this study is to compare the outcome of hepatic resection for metastases of colorectal cancer in two similar groups of patients using two different techniques of hepatectomy.
The group I was constituted of 25 patients (15 men and 10 women, with mean age of 63.1 years - range, 32 to 80 years). The surgical procedure was hepatic resection employing the posterior approach of the hepatic hilum technique with intermitent clamping of glissonian sheeths.
The group II was constituted of 23 patients (12 men and 11 women, with mean age of 63.8 years - range, 40 to 73 years). The surgical procedure was hepatic resection employing the conventional technique with in mass continous clamping of the hepatic pedicule.
There was no statistical difference between the two groups concerning sex, age and number of metastases. The total lenght of ischemia was superior in the posterior approach patients (group I) with a mean of 84.2 minutes against 37.5 minutes of the group II (p < 0.0001). There was no influence of the ischemia time in the postoperative hepatic function, postoperative course or recovering time.
The survival rate was superior in the group of the posterior approach technique: 778.8 ± 410.2 days (group I) vs 572.5 ± 349.7 days (group II). Although this difference, it was not statistically significant (p = 0.14).
We concluded that the posterior approach procedure is a feasible and safe technique allowing to perform segmentary and subsegmentary anatomical resection. This new technique seems to improve the survival of patients with hepatic metastases of colorectal cancer. This results still has to be confirmed by subsequent series with greater number of patients.

### P241

SURGICAL TREATMENT OF MALIGNANT LIVER TUMORS. ANALYSIS OF 124 CASES.

E.Hadjiyannakis, S.Drakopoulos, A.Papakonstantinou, N.Georgopoulos, S.Mathioulakis, N.Nikitakis, A.Poultsidi, H.Harisis, F.Filippidou, K.Anastasakou.

1st Surgical department and Transplant Unit - "Evagelismos" Hospital of Athens.

During the last 20 years (1975 - November 1994) we treated 135 cases with liver neoplasms, 124 malignatn, in different hospitals.

Resectability rale was 67,75% (84 cases) while 40 cases (32,25%) seemed inoperable.

122 cases were operated upon electively; 2 cases under emergency conditions due to rupture of the tumors into the abdminal cavity. In 45 cases the tumor was rightsided and in 37 cases leftsided. In 2 cases it extended in both. lobes. In these 2 cases we perfurmed liver transplantation Right lobectomy was performed in 13 cases right extended hepatectomy in 21 cases left lobectomy in 13 cases, left extended hepatectomy. In 11 cases and segmental ressection in 24 cases.

Bathological examination revealed hepatocellular carcinoma in 74,4% (92 patients).

Perioperative mortality was 3,22% we maintain that liver. Surgery plasmas very satiofactory results when both the Surgical principles and resectability criteria are being well considered and applied.

### P242

PRE-OPERATIVE DIAGNOSIS OF HEPATIC ADENOMA AND FOCAL NODULAR HYPERPLASIA. IS IT RELIABLE?

P. Herman, V. Pugliese, M.Z. Salem, M.A.C. Machado, T. Bacchella, M.C.C. Machado, H.W. Pinotti.
Department of Gastroenterology, Hospital das Clínicas-University of São Paulo Medical School; Brazil.

Benign liver tumors as hepatic adenoma (HA) and focal nodular hyperplasia (FNH) are uncommon, generally affect young women, but their pathogeny and outcome are totally different. The pre-operative diagnosis of this lesions is often difficult. Surgical resection of HA is advocated based on the incidence of bleeding complications, in some instances lifethreating. Moreover, neoplastic degeneration of HA have also been reported. In contrast, FNH is often an incidental finding and there is no proven cases of hemorrhage or malignant degeneration. For this reason it must be conservatively treated.
The aim of this study was to evaluate 17 young female patients with benign liver tumors (10 FNH, 7 HA), trying to establish a pre-operative criteria for the differential diagnosis, avoiding therefore a laparotomy and eventual hepatic resection. In the present study, all patients were submitted to a surgical biopsy or to a hepatic resection.
Based on clinical and laboratorial data we were not able to distinguish HA from FNH. Even with the development of imaging methods that were used in combination (ultrasound, computed tomography, scintigraphy with HIDA and sulfur-coloid, magnetic ressonance and angiography) the differentiation was not possible in most cases.
Surgical biopsy is the only safe alternative for the differential diagnosis between HA and FNH and when in doubt, a hepatic resection can be safely performed.

### P243

RESULTS OF A LARGE HEPATOCELLULAR CARCINOMA TREATED BY GIVING CHEMOTHERAPY THROUGH SWAN-GANZ CATHETER

Shu-Yuan Han, Zhi-Qiang Huang

Dept HB Surgery of General Hos of PLA CHINA

Patient is a 51 years old farmer who had pain in right abdomen for 2 months in 1986, at age 42. CT scan taken and showed a parenchymal focus of 11.8cm in right liver lobe. He was admitted for hepatocarcinoma on Feb 24, 1986. Examination revealed a palpable liver 4cm below the right costal margin. HBSAG(+), AFP 4000ng/ml, US detected a clear edge focus in right liver lobe.Operation upon on Mar 6, 1986 because the tumor was too big to resect at that time. So the hepatic artery was ligated and Swan-Ganz cannula was inserted into the portal vein. 5 days postoperation portal vein catheterization was carried out, chemotherapy with MMC was bigan. Swan-Ganz catheter was taken out at Apr 15,1986.

After treatment CT US and AFP reexamined and follow up over and again. Up to the present, follow up is now more than 9 years, proved the patient was good health. CT scan was only a calcified focus of 2.5cm,US shows it is clear edge while colour doppler failed to show any sign of blood flow in it, AFP 99ng/ml.
The method has used for far advanced large hepatic carcinoma. The curative effect was marked, successful and satisfactory in this case. We consider it is through ligation of hepatic artery and chemotherapy through portal vein catheter and regular stop the portal venous flow, oxygindificit ployed a chief role.

### P244

DIAGNOSIS OF PRIMARY MALIGNANT LIVER TUMOURS BY ULTRASOUND-GUIDED ASPIRATION CYTOLOGY AND BIOPTIC HISTOLOGY

C.Gheorghe,G.Aposteanu,L.S.Gheorghe,G.Jovin,Al. Oproiu
Center of Gastroenterology,Fundeni Hospital, Bucharest,Romania

We evaluated the concordance of ultrasonically-guided aspiration cytology(AC)and biopsy histology(BH)in a study employing 46 patients with one or more intrahepatic masses.We aimed to correlate the concordance of AC(performed with Chiba 22 gauge fine needles)and BH(performed with Menghini and TruCut needles)with tumor's parameters: size(3 cm,3-5 cm,>5 cm-graded);histology(low,moderate and well differentiated tumor);and with the statement of the hepatic parenchyma:cirrhosis(31),chronic active hepatitis(4),normal(11). The concordance AC-BH was 35%,52%,79% in the case of lesions of 3 cm,3-5 cm,and > 5 cm and 24%; 48%,86% in well,moderate and poor differentiated tumors,respectively.We conclude that the concordance AC-BH is low in the case of small tumours and significantly increases with the tumor'size; is low in the case of well differentiated tumors (low accuracy of AC);did not varied with the underground state of diagnosis in the hepatic parenchyma($p<0.2$).The accuracy of diagnosis significantly increased when the AC and BH are performed together when primary liver neoplasms are to be diagnosed: AC 53%; BH 76%; AC and BH 87%.

### P245

### Usefulness of a New Convex Type Puncture Probe in Early Diagnosis of Small Liver Cancer

Hiroshi Kasugai, Tsugio Kitamra, Masaharu Tatsuta, Itaru Kaji, Atsuo Inoue, Toru Otani, Shigeru Okuda (The Center for Adult Diseases, Osaka, Japan), Yasuo Ishikawwa, Hiroshi Sato (Hitachi Medical Corporation, Chiba, Japan)

Accurate puncture is very important for the diagnosis of small lesions in the liver, pancreas and so on by ultrasonically guided fine needle biopsy. We developed a new convex type puncture probe which was devised so as to push out the needle from the center of the transducer to three directions at the angle of 0, 18, 30 degree. This new convex type puncture probe made it possible to perform the biopsy in any part of the liver including subdiaphragmatic area through intercostal apace. Therefore, it also became possible to reach where it had been difficult to aim at for PEIT to treat small liver tumors by the previous probe. The clinical usefulness was investigated in 489 cases with small liver tumors admitted to our hospital from July 1990 to December 1993. Among them, indication for ultrasonically guided fine needle biopsy was confined to negative or indefinite cases by imaging diagnosis such as CT, MR or angiography. Fine needle biopsy using convex type probe showed higher diagnostic results than imaging diagnosis, especially for the nodules smaller than 2.0 cm in diameter or the well-differentiated type. Consequently, PEIT became possible after the liver biopsy for every lesion. It became also possible to pierce the lesions on the surface of the liver which was difficult until now. The newly developed convex type puncture probe is very useful as it has a wide indication of the area of the biopsy and is easy to operate.

### P246

### FIBROLAMELLAR CARCINOMA. AN ANALYSIS OF FIVE PATIENTS.

A. López Labrador, A. Alvarado, C. Loinaz, C. Jiménez, M. Abradelo, M. Marcello, D. Rodriguez Romano, E. Moreno González.
General and Digestive Surgery Dept. "12 de Octubre" Hospital. Ctra. Andalucia Km. 5.5. Madrid. Spain.

INTRODUCTION.- Fibrolamellar carcinoma (FLC) is a liver tumour that can be differentiated from common hepatocellular carcinoma (HCC). There are still many doubts about whether it is or not a better prognosis tumour.
MATERIALS AND METHODS.- From 1985 to 1994, 134 patients with diagnosis of HCC were operated on in our department. Only 5 of them (3.7%) were FLC. Three males, 2 females, with a mean age of 23.8 years (range 19-30).
RESULTS.- The 5 FLC were treated by hepatic resection with tumour free margin of approximately 2 centimeters. All were non cirrhotics livers. There was no perioperatively mortality.

| Pts | Tumour Size (cm.) | Type Resection | Lymph Node Invas. | Follow-up months | Recidive | Outcome |
|---|---|---|---|---|---|---|
| I | 12 x 12 | R.Triseg. | + | 9 | + | D |
| II | 8 x 8 | L.Triseg. | + | 46 | + | D |
| III | 6 x 8 | IV,V,VI Seg. | - | 29 | - | A |
| IV | 9x9x6 | V, VI Seg. | + | 17 | + | A |
| | | | | (15 m.& reinterv) | | |
| V | 16x16x11 | IV,V,VI Seg. and partially II,III,VII,VIII | - | 5 | - | A |

CONCLUSIONS.- Similarly to other reports we find that FLC appears in young people with non cirrhotic liver, being often resectable. Lymph node metastasis seems to be a highly valuable prognostic index.

### P247

### HEPATIC RESECTION COMBINED WITH PRE- AND POSTOPERATIVE CHEMOEMBOLIZATION (CE) IN THE TREATMENT OF HEPATIC MALIGNANCIES

A.M.Granov, D.A.Granov
Department of Surgery, St.Petersburg Research Institute of Roentgenology and Radiation Therapy, Russia

The aim of this study was to assess the results of surgical resection plus preoperative or adjuvant postoperative CE for the treatment of malignant liver tumors. Between 1986 and 1993, 50 liver resections (35 hepatectomies or tri-segmentectomies, and 15 wedge resections) were performed in 46 patients with hepatocellular carcinoma (HCC, 20 cases), gallbladder carcinoma (2), and liver metastases (Mts) from colorectal (18), carcinoid (3), renal (2) and gastric (1) cancer. Okuda stage I HCC was in 14 pts and stage II in 6. Gennari stage I, II, and III liver Mts were in 7, 14, and 3 pts, respectively. We used 15-20 mg of liposoluble cytostatic Dioxadet in iodized oil for CE. CE was made in 11 pts 2 to 5 weeks prior to surgery. One to four adjuvant CEs through the hepatic artery and/or portal vein was performed in 39 patients including 4 with preoperative CE. The treatment was beginning as early as 1 to 6 weeks after the surgery and was repeated every 6 months. Only one postoperative death occured. Complication of CE was acute cholecystitis in 2 pts. The 1-2-3yr survival was 100%,64%,43% for Okuda stage I HCC and 88%, 50%,17% for stage II. In liver Mts, the 1-2-3yr survival was 100%,86%,43% for Gennari stage I, 78%,43%,21% for stage II, and 67%,33%,33% for stage III. Both pre- and adjuvant postoperative CE may be useful for surgical treatment of malignant liver tumors.

### P248

### LIVER RESECTION IN ADVANCED AGE

G. Nuzzo, I. Giovannini, F. Giuliante, G. Boldrini, C. Chiarla, G. Tebala, M. Vellone
Dept of Surgery (Chirurgia Geriatrica), Catholic University School of Medicine, Rome, Italy

The risk due to advanced age has motivated for many years exclusion of old patients from resective surgery of the liver. More recently, improved surgical techniques, minimization of bleeding and optimization of postoperative care have contributed to extend the indications for liver resection to elderly pts. We report the results obtained in 38 pts older than 65 yrs, undergoing liver resection for primary tumors (10 pts, 9 with cirrhosis), secondary tumors (16 pts), biliary cancer (6 pts), other lesions (6 pts). ASA score was 1 in 13 pts, 2 in 3 pts, 3 in 21 pts, 4 in 1 pt. Thirteen pts had major resections, 18 pts resections of 2 segments, 7 pts smaller resections. Two patients died postoperatively (1 ruptured aortic aneurism, 1 liver failure and sepsis). Minor complications (respiratory) occurred in 7 pts, hepatic insufficiency in 2 pts, bowel occlusion in 1 pt: these complications occurred in 10 "ASA 3" pts and in 2 "ASA 1" pts (3 of these were cirrhotic). Comparison with the results of more than 100 resections performed in younger pts during the same period did not show relevant differences. These data support the concept that old age in itself, in the absence of associated diseases, is not a controindication for resective surgery of the liver.

### P249

### HEPATIC RESECTION FOR MALIGNANT DISEASE-RISK ASSESSMENT FOR PRIMARY TUMORS AND METASTASES

T.U.Cohnert ,H.G. Rau, E. Buttler, C. Reuter, K.W. Jauch, F.W. Schildberg
Department of Surgery, Klinikum Grosshadern, University of Munich, Germany

Liver resection for patients with primary or secondary malignancies of the liver became an important curative therapy. To improve patient survival a preoperative risk assessment can help to find the optimal regimen for primary resection or interventional pretreatment.
276 patients (pts) underwent consecutive hepatic resections between Nov.90 and Oct.94 (146 male, 130 female, mean age 56,7±13,8yrs, range 19-85 yrs.) with a mortality of 3,6% (10/276 pts.). Data collection was retrospective through March 94 , and prospective from April 94. Besides clinical and operative data a C14-Aminopyrine-Breath Test and a MegX Test was performed. The Parenchymal Hepatic Resection Rate (PHRR) and the Liver Resection Index (LRI) were compared by Mann-Whitney U Test.
189 pts. were operated because of malignant disease: primary hepatobiliary malignomas in 52 pts and liver metastases in 137 pts. Operative mortality was 7,7% in primary tumors (4/52 pts) and 4,5%(6/137 pts) in metastases. Aminopyrine Breath Test and MegX-Test did not show significant differences between survivors and non-survivors.

| | PHRR (%) | | | LRI | | |
|---|---|---|---|---|---|---|
| pts | n | survivors | non-surv. | n | surv. | non-surv. |
| primary tumors | 20 | 13,0+17,1 | 38,8+17,5 | 18 | 1,1+1,6 | 0,5+0,7 |
| metastas | 43 | 15,7+17,4 | 41,8+22,9 * | 39 | 11,6+18 ,9 | 0,2+0,3 ** |
| | | | * p<0,05 | | | **p<0,01 |

One of the main prognostic factors in the extent of the intended hepatic resection that can be quantified by the Parenchymal Hepatic Resection Rate. Improvement of prediction can be reached by calculating the Liver Resection Index especially in patients with hepatic metastases.

### P250

### HEPATIC RESECTIVE SURGERY IN THE ELDERLY

Mazziotti A., Morganti M., Jovine E., Grazi G.L., Principe A., Gallucci A., Gruttadauria S., Gozzetti G.
2°Department of Surgery, Policlinico S.Orsola, University of Bologna, Italy

**INTRODUCTION**: The progressive increase of the average length of life and improved diagnostic tests lead more often to the detection of liver tumors in the elderly patients thus increasing the indication to hepatic resective surgery. Out of a total of 604 hepatic resections performed in the last 13 years, 81 were carried out in patients over 69 years of age and are reported on in this retrospective study. **MATERIAL AND METHODS**: The age of the patients ranged between 69 and 82 years (mean 71.5). Fiftyfive (67.9%) were male and 26 (32%) female. In 29 cases, cirrhosis was present (35.8%). The indications to surgery were 46 hepatocellular carcinomas (HCC), of which 28 were in cirrhotic livers, 25 metastases of which 21 originated from colorectal cancer, 4 cholangiocarcinomas, 1 ruptured hemangioma and 6 cases for other diseases. The preoperative evaluation consisted of extensive assessment of liver function which included the lidocaine test (MEGX). Respiratory and cardiovascular functions were also evaluated. Thirteen right hepatectomies, 4 left hepatectomies, 2 extended right hepatectomies, 1 extended left hepatectomy, 46 segmentectomies (1 to 3 segments), and 15 wedge resections were carried out. Statistical correlation of the variables was performed by means of $X^2$ test comparing two groups of patients respectively over and under 69 years of age. The actuarial survival rate was evaluated with the Kaplan Meier method. **RESULTS**: In elderly patients, a statistically significant (p<0.0001) higher incidence of associated diseases was found compared to younger patients. In particular, cardiovascular (33.3%) and respiratory (22.2%) diseases. Fortyfive patients did not require a blood transfusion (55.5%). Post-operative mortality was 3.7%, similar to that in the younger age group (2.8%). Two out of the three post-operative deaths were cirrhotic patients. Overall, the post-operative complication rate was 25.9% in the elderly group and 21.3% in the group under 69 years of age. The complication rates were higher in both groups when cirrhosis was present (31% vs 31.4%), expecially in regards to the incidence of jaundice and ascites. The mean post-operative hospital stay was similar to that of the general population (16 days). The actuarial survival rate at 1, 3 and 5 years found in HCC, HCC with cirrhosis and in colorectal metastases, was similar to that of the younger population and is reported in the table.

Survival in elderly patients operated on for hepatic tumors

| | 1 year (%) | 3 years (%) | 5 years (%) |
|---|---|---|---|
| HCC | 78.1 | 54.5 | 54.5 |
| HCC on cirrhosis | 70.5 | 50.0 | 50.0 |
| Colo-rectal metastasis | 87.5 | 56.7 | 40.5 |

**CONCLUSIONS**: The results of elective hepatic resective surgery in the elderly are overlapping with those found in the rest of the population. In order to achieve better results, it is important to perform a thorough evaluation of hepatic, cardiovascular and respiratory functions. Anatomical resections with limited use of blood transfusions allow a safe surgery with acceptable morbidity and mortality outcomes.

### P251

### ULTRASOUND APPEARANCE OF HEPATOCELLULAR CARCINOMA

T.Pavlidis, I.Spathopoulos, D.Kouvelas, S.M.Janković
Second Department of Surgery, Agios Pavlos Hospital, Thessaloniki, Greece

Modern imaging techniques in combination to α-feto-protein measurements made possible the early diagnosis of hepatocellular carcinoma(HCC) leading to effective surgical treatment and reliable follow-up. Ultrasound(US) appearance might be variable depending on location and stage of the tumor.In order to evaluate this range we studied retrospectively the notes of 28 patients with HCC confirmed by tissue diagnosis, irrespective of way of treatment. We compared closely the US findings with the pathology reports to find out any relationship. Our study indicated that: (a) Tumors less than 5cm in diameter(n=6, small HCC), which are considered as the most curable, are seen on US as homogenous hypo echoic lesions. However, a distal non-echoic halo could be occasionally distinguished. The pathology revealed compact mass; (b) Tumors between 5 and 8cm in diameter(n=14) were seen on US as hyper echoic lesions, mainly. However, a mixed echoicity,or a distal hypo echoic halo could be found,too. The pathology confirmed fatty degeneration and dilatation of sinusoids and bile ducts; (c) Tumors more than 8cm in diameter(n=8) appeared mainly with mixed echoicity, and sometimes with homogenous hyperechoicity. The pathology revealed combination of compact mass with dilatation of sinusoids and bile ducts, and necrotic areas with cavitation. In conclusion, it seems that the appearance of HCC on US correlates well with size and structure of the tumor.

### P252

### EXPERIENCE WITH 146 LIVER RESECTIONS: INDICATIONS, TECHNIQUES AND RESULTS

E.Gifre, J.Balsells, R.Charco, JL.Lázaro, E.Murio, I.Bilbao, C.Margarit.

During the period of 1988-94, 146 liver resections in 143 patients were performed in our Unit. Mean age of patients was 52.2 (range:14-81 years), 97 were males and 49 females. Malignant hepatic tumors (115 cases), particularly hepatocellular carcinoma in cirrhosis (58 cases) and liver metastasis from colorectal cancer (46 cases) have been the principal indications. Four hepatectomies were performed for traumatic hepatic injuries and 31 for benign diseases: hemangioma and hydatic cysts were the main indications. Almost half of the patients submitted to hepatic resection had liver cirrhosis. Only those cirrhotics with good liver function, groups A and B according to Child-Pugh criteria were considered for resection.
Techniques: Anatomical resections following the lobar or segmental division of the liver were more frequently done in cases of tumors appearing in normal livers whereas ultrasound guided radical non-anatomical resections was indicated in cirrhotic patients. In 44 cases a Pringle manouver was used during the hepatic parenchimal division. Frequently another type of operations were performed at the same time.
Results: operative mortality has been of 8.2 %, most of patients died from liver failure (4.1%). Postoperative complications were: biliary leaks (7.5%), hemorrhage (4.1%) and infections (10%).
Risk factor for morbi-mortality in our experience have been liver cirrhosis. cholestasis and major associated surgical tecniques.

### P253

**EXPERIENCE WITH FIBROLAMELLAR HEPATOCELLULAR CARCINOMA**

L.Lara, MA., Margarit, C., Charco, R., Balsells. J., Lázaro, JL., Murio, J.E., Fortuño, A., Bilbao, I., Bonnin, J.

Liver Transplantation Unit. Hospital General Universitario Vall d'Hebron. Barcelona, SPAIN.

Fibrolamellar hepatocellular carcinoma(FLHC) is a uncomon histologic subtype of hepatocellular carcinoma (HCC). It has a low incidence, it usually occurs in young people and it is not associated with cirrhosis. Serum alpha-fetoprotein (AFP) is usually normal.
From 1970 to 1994, 306 HCC were recorded in our institution. Four tumors were diagnosed as a FLHC. Two were weman and two were man. Mean age was 32.1 years ( range: 22 to 49 ).Clinical symptoms were: back-pain and fever in 1 patient, ascites and toxic syndrome in 1, right subcostal pain in 1 and no symptoms in 1.
Laboratory tests showed a normal AFP and negative HBsAg in all patiens. Computed tomography revealed a large mass in right lobe in two patients. Kidney and diaphragm were involving in one, and celiac lymph nodes and left lobe in other. In the remaining two patients tumour invaded both lobes.
Treatment: Right hepatectomy and nefrectomy, and partial diaphragm resection was perfomed in one patient. Total hepatectomy and duodenopancreatectomy follow "split" liver transplant was perfomed in other. The remaining two patients underwent laparotomy, although no resection was perfomed.
Follow range in patients underwent resection was 12 to 24 months. No mortality was found in the follow-up, but tumour recurrence was detected in all resected patients.
In conclusion, FLHC has a more favourable prognosis than the usual HCC, with a better average survival and resectability rate and distinguishing this histological subtype is important for surgical management and survival prognosis.

### P254

**MAJOR LIVER RESECTION FOR CANCER IN PATIENTS WITH OBSTRUCTIVE JAUNDICE**

D. Cherqui, R. Alon, N. Rotman, M. Julien, P.L. Fagniez. Department of Surgery. Université Paris XII-Hôpital Henri Mondor, Créteil, France

It has been suggested that obstructive jaundice increases the risk of liver resections because of reduced tolerance to ischemia and regneration capacities, increased operative bleeding and general effects associated with jaundice. Therefore, some authors recommend preoperative percutaneous biliary drainage. However, biliary drainage may lead to sepsis and tumor seeding along the catheter tract. From 1989 to 1993, 60 major liver resections (≥ 3 segments) were performed. Eight patients (13%) had obstructive jaundice and underwent resection without previous biliary drainage. Mean serum bilirubin was 215±108 µmol/L (80-439). They included 3 hilar cholangiocarinomas, 3 intrahepatic cholangiocarcinomas extended to the hilus and 2 tumors with thrombus extension in the biliary tract. These cases were compared with 52 major resections in patients without jaundice.

| mean values | jaundice + | jaundice - | p |
|---|---|---|---|
| N | 8 | 52 | |
| extended resection | 8 (100%) | 8 (15%) | < 0.001 |
| vascular exclusion | 6 (75%) | 16 (30%) | < 0.05 |
| clamping time | 39 min | 38 min | |
| transfusions | 4.6 units | 3.6 unités | NS |
| operative time | 5.9 h | 3.6 h | < 0,01 |
| AST peak | 543 | 311 | NS |
| minimal PT | 54 | 53 | |
| biliary fistula | 50 % | 6 % | < 0,01 |
| mortality | 1 (12,5%)* | 1 (2%) | NS |
| hospital stay | 28 days | 15 days | < 0,01 |

* preop serum bilirubin = 439 µmol/L, hypoalbuminemia, renal failure

These results suggest that present liver resection techniques are applicable to most patients with obstructive jaundice without previous biliary drainage. In these patients, semi-urgent operation is required because of ongoing jaundice, a long and complex procedure is necessary and the incidence biliary fistula is high. Intraoperative bleeding, tolerance to ischemia and regeneration were not affected by jaundice in this series. Preoperative drainage may be indicated in selected cases (bilirubin > 300, prolonged jaundice, hypoalbuminemia, renal failure).

### P255

**ADJUVANT HEPATIC ARTERIAL CHEMOTHERAPY AFTER CURATIVE RESECTION OF HEPATIC METASTASES - INFLUENCE ON SURVIVAL?**

K.-P. Riesener, M. Höfer, G. Winkeltau, V. Schumpelick
Department of Surgery, Medical Faculty, Rhenish Westfalian University of Technology Aachen, Germany

Radical resection has proven to be the only effective therapy in hepatic metastization. Approximately half of the patients will develop hepatic recurrences during follow-up. In this non-randomized study the influence of adjuvant regional chemotherapy on outcome after hepatic resection was evaluated in our patients.

**Methods:** From 1.1.1986 to 31.12.1993 radical hepatic resection was performed in 91 patients after colorectal (n = 77) or other (n= 14) primaries. 42 patients received an implantable port system for regional chemotherapy. Although implantation of the port was not predetermined, the groups with and without chemo-therapy were comparable regarding tumour stage and extent of resection. Our chemotherapy protocol consisted of six courses of Mitomycin C (first day) and 5-FU (five days) with intervals of one month.

**Results:** Due to several reasons only 30 patients were treated with three or more chemotherapeutic courses. Short term survival of this group was significantly better compared with untreated patients (1-year-survival 93 vs 75%), but follow-up resulted in similar survival rates after three years (40 vs. 47%). Extrahepatic metastization was frequently observed in either group prior to death.

**Conclusions:** Adjuvant hepatic artery infusion chemotherapy did not improve long-term survival in patients following radical hepatic resection for metastases.

### P256

**MULTIFOCALITY OF HEPATOCELLULAR CARCINOMA IN CIRRHOSIS: A CLINICO-PATHOLOGIC STUDY**

M Schwartz MD, I Fiel MD, S Emre MD, P Sheiner MD, S Guy MD, C Miller MD The Mount Sinai School of Medicine, NY, NY USA

Hepatocellular carcinoma (HCC) is a known sequela of cirrhosis, particularly in the setting of underlying viral hepatitis; multifocality is recognized to occur, but its incidence is not well-defined. This study was undertaken to better define the incidence of multifocality of HCC in cirrhosis. MATERIALS AND METHODS: The livers of all patients with cirrhosis and HCC who underwent liver transplantation at The Mount Sinai Hospital between 9/88 and 9/94 were studied. The nature of the underlying liver disease. and whether or not the tumor had been noted preoperatively, were noted. Livers were "bread-loafed" in 1 cm slices, and the presence, size. and number of tumors were recorded. Prior to 10/91, patients with known HCC > 5cm were excluded from transplantation; thereafter, such patients were included in a multimodality protocol. RESULTS: Sixty patients with cirrhosis and HCC were transplanted. Underlying diagnoses were: hepatitis C- 37, cryptogenic- 7, hepatitis B- 6, EtOH- 5, biliary cirrhosis- 5. Findings, broken down by size (< or ≥ 5cm) and whether or not HCC was diagnosed before transplant, were as follows:

| Group (n) | Diameter of largest (mean ± sd) | # Lesions 1 | 2 | >2 |
|---|---|---|---|---|
| HCC < 5cm | | | | |
| unknown preop (33) | 2.2±0.9 cm | 20 | 6 | 7 |
| known preop (8) | 2.7±1.0 cm | 5 | 0 | 3 |
| HCC ≥ 5cm | | | | |
| unknown preop (6) | 7.4±2.7cm | 2 | 0 | 4 |
| protocol (13) | 9.1±5.2 cm | 2 | 4 | 7 |

When stratified for tumor size, the incidence of multifocality was similar whether or not HCC was recognized pretransplant. In patients with HCC ≥ 5cm, multifocality was found in 15/19 (79%); in cases with HCC < 5cm, the incidence of multifocality was 16/41 (39%). Of the 31 livers with multifocal tumors, 20 demonstrated bilobar involvement. including 10 cases with HCC < 5cm. Multifocal tumors were present in 21/43 patients with viral hepatitis vs. 10/17 patients with non-viral etiology. CONCLUSION: HCC in the setting of cirrhosis is commonly multifocal. even when tumor diameter is small. Recognition of this fact has important implications in the choice of therapy

### P257

### SPONTANEUS RUPTURE OF THE HEPATOCELLULER CARCINOMA

Okay NAZLI, Atilla ÇÖKMEZ, Serhat GÜR, Bilek TAŞKIN, Orkan KARABAĞ, Sümer DENİZ

First Department of Surgery, Atatürk State Hospital, İZMİR TURKEY

In hepatocelluler carcinoma (HCC) patients who are candidates of the surgical treatment,regardless of the operative mortality, five year survival is 6-51% in different series. The HCC patients whose diagnose is spontaneus rupture is very rare and after surgical treatment their survival are short.

In this case report, a patient surviving for three years with spontaneus HCC rupture will be presented.

Male patient, 68 years old, admitted to emergency unit with acute abdomen, hypovolemic schock. Urgent laparotomy was undertaken. In exploration, spontaneus rupture in the right hepatic lobe was diagnosed and irregular right hepatectomy was performed including tumor mass.

In pathologic study HCC was diagnosed, and, postoperatively no residual tumor mass is traced in CT. At the second postoperative year recurrence is diagnosed in the right hepatic lobe, and right regular hepatectomy was performed. No recurrence is traced at the third postoperative year.

We support that when spontaneus HCC rupture is encountered either in emergency, resection with safe margins must be carried without any hesitation.

### P258

### THE PERITONEAL AND SYSTEMIC CYTOKINE RESPONSE TO PANCREATIC SURGERY

JM Badia, SA Whawell, DM Scott-Coombes, RCN Williamson, JN Thompson.
Department of Surgery, Royal Postgraduate Medical School, Hammersmith Hospital. Du Cane Road, London, United Kingdom.

Cytokines are products of activated leukocytes that mediate the inflammatory postoperative changes within the peritoneal cavity and may also be partly responsible for the systemic acute phase response to surgery.

*Aims.* To investigate the peritoneal and systemic cytokine response to elective pancreatic surgery during the first 72 hours after operation.

*Methods.* Six patients undergoing pancreatic surgery were studied (1 cholangiocarcinoma, 2 chronic pancreatitis, 3 adenocarcinoma). Peritoneal fluid was sampled through abdominal silastic drains and venous blood was taken from a central line. A blood sample was taken preoperatively. Samples of blood and peritoneal fluid were taken at 6, 8, 10, 12, 36, 48 and 72 hours after the beginning of the operation. They were centrifuged at 2500 g for 10 minutes at 4° C and the supernatant stored at -80° C until assay. Interleukin-1 beta (IL-1), Interleukin-6 (IL-6) and Tumour Necrosis Factor (TNF) were measured in plasma and peritoneal fluid using immunoassays.

*Results.* Operative procedures were 5 pylorus-preserving proximal pancreatectomy and 1 choledoco-jejunostomy. Mean operative time was (mean ± 1 standard error) 5.3 ± 0.3 hours. There was no postoperative complication. All peritoneal fluid samples had detectable TNF, IL-1 and IL-6, with maximum values: TNF 298 ± 140 pg/mL at 8 hours after beginning of operation; IL-6 244 ± 59 ng/mL at 12 hours; and IL-1 372 ± 142 pg/mL at 12 hours. Plasma IL-1 and TNF concentrations were very low or undetectable (< 10 pg/mL). Plasma IL-6 levels were 300 fold lower than peritoneal levels, with maximum value of 836 ± 548 pg/mL at 8 hours.

*Conclusions.* There is a high level peritoneal cytokine response to pancreatic surgery which is responsible for local inflammatory changes and probably also produces the secondary increases in systemic cytokine concentrations observed after operation.

Dr.Badia had the grant FISS 94/5024 (Ministry of Health of Spain).

### P259

### CAVERNOUS HEMANGIOMA OF THE PANCREAS: A CASE REPORT.

L. Harsányi, J.Sebők*, I. Ádler**
1st Department of Surgery, *Department of Pathology, **2nd Department of Internal Medicine
Semmelweis University of Medicine, Budapest, Hungary

Authors report the case of pancreatic hemangioma in a 38 year old female patient. The patient had been admitted to hospital with blunt, girdle-like upper abdominal pain where ultrasonography and CT scan revealed a solid tumor in the pancreatic body and cholecystolithiasis. Cholecystectomy, segmental resection of the gland was performed with end-to-side pancreaticogastrostomy. The histological work-up of the specimen verified cavernous hemangioma of the pancreas. As far as authors know, this is the first ever report of this entity.

### P260

### THE EFFECT OF FATTY EMULSIONS ON EXOCRINE PANCREATIC FUNCTION IN EARLY POSTOPERATIVE PERIOD

K.Witkowski, M.Pardela, A.Kozłowski, J.Piecuch, A.Urbaniak, W.Orkisz
Dept. of General and Vascular Surgery, Silesian Medical School, Zabrze, Poland

The administration of fatty emulsions during total parenteral nutrition (TPN) and its influence on exocrine pancreatic function has been recently discussed. To determine whether such influence exist and how it affects the postoperative pancreatic function was the goal of this study. The subject was group of 28 patients with pancreatic head cancer who underwent pancreato-duodenectomy. 15 of them were treated postoperativly with TPN (group I) and the other 13 were given limited i.v. nutrition without fatty emulsions (group II).

The pancreatic juice was diverted from Wirsung duct by nasopancreatic catheter and collected in six-hour fractions. This juice was analysed for volume, protein, bicarbonate and enzyms like amylase and chymotrypsin.

Slow increase of these values during the first three days after operation has been found in both groups. This rise of measured values, was slightly faster in group I starting from the fourth day and became steady after 6 days. We found that juice volume was almost the same in both groups. Other values were a little higher in group I (especially protein and amylase level), but the differences were not signifficant. According to the above data the two ways of parenteral nutrition seem to be of the almost the same value for pancreatic exocrine function.

### P261

### PSEUDOLYMPHOMA OF THE PANCREAS: A RARE ENTITY.

E.Hatzitheoklitos, C.Dervenis, H.Friess, W.Mohr, M.Büchler

Departments of Surgery and Pathology, University of Berne, 1st Department of Surgery, Hippokration Hospital, Thessaloniki.

Pseudolymphoma has been described as occurring in a wide variety of sites including the lung, small intestine, stomach and gall bladder. We present a case of pseudolymphoma of the pancreatic body and tail. A 68-year old woman presented with weight loss, recurrent abdominal pain, accompanied by an elevation of serum pancreatic enzymes. Ultrasonographically there was a suspicion of pancreatic tumor in the body and tail. In CT scanning there was an enlargement of the pancreatic body with obstructed pancreatic duct accompanied by dilatation of the pancreatic duct in the tail region. ERCP showed normal duct in the pancreatic head. In the pancreatic body there was a filiform stenosis followed by massive dilatation of the Wirsung duct within the pancreatic tail. Preoperative diagnosis was tumor localized in the pancreatic body with obstructive lesion in the pancreatic tail. During surgical exploration pancreatic body and tail were altered macroscopically comparable with a suspicion for malignancy. A pancreatic left resection was carried out. Histology revealed massive follicular lympatic hyperplasia based on the presence of hyperplastic follicles with germinal center and mixed infiltration of plasma cells and mature lymphocytes with no significant cytologic atypia. This is the 2nd case of pseudolymphoma of the pancreas in world literature (Hum Pathol 22:724-6;1991). Pseudolymphoma of the pancreas seems to be a benign lesion which develops on the basis of chronic inflammation. Occuring at other GI locations in moderate frequency it obviously represents a rare entity in the pancreas.

### P262

### MANAGING PANCREATIC TRAUMA

A.Agorogiannis and E. Naoum

Surgical Unit.District General Hospital Of Larisa-Greece

Pancreatic trauma presents a number of difficalt problems to the Surgeon.The aim of this study is to present and to discuss these problems and to find out the best treatment. The last 16 years we operated on 614 patients with multiple abdominal trauma.42 patients had had trauma to the pancreas.The 30 patients were men and 12were women.The mean age of all thse patients was 44 years.The 34 cases were due to blund abdominal trauma,,1 case to penetrating injury,3 cases to gan shot wounds,2 cases to horse kick and 4 cases to falling by trees.All the patients had associated injuries of the head 15 cases,liver trauma 10 cases,spleen rupture 10 cases,blund trauma of thethorax and multiple bone fractures.In 90% of pancreatic truma there were associated injuries.In our cases the injuries were classified as class I include contusions (28 cases),class 2 severe contusions involving parenchymal destruction (10 cases), class 3destruction of the major duct (2 cases) and class 4 involve injuries of the duedenum and the pancreas (4 cases) The contusions need only drainage of the aerea,the class 2 thrama may need some sutures of the pancreatic parenchyma the class 3 needs resuture of the duct or more complicated operations and the class 4 may need pancreatoduodenectomy.Conclusions:The pancreas is injured in 3% to 12%.Most of the blund trauma is usually the result of steering wheel or seat belt injuries from motor vehicle accidents.Usually there is an even distribution of injuries to the head,body,and tail of the pancreas.The head injury is associated with injury to the common bile duct,duodenum,liver,right kidney,and colon.These injuries are associated with highest mortality.The patient may complain of epigastric pain but is often asymptomatic.ERCP define major duct injury.The most reliable is laparatomy.The treatment consists of arrest of hemorrhage and contain contamination from the gastrointestinal tract.Drainage,jejunostomy,duct repair,resection, with Roux-en-Y anastomosis are the usual operations.The complications are pancreatitis,fistula,pseudocyst,endocrine insuficiency and sepsis.

### P263

### SURGICAL MANAGEMENT OF PANCREATIC TRAUMA : STUDY OF 65 CASES

M.A.C. MACHADO, P. VOLPE, A.L. SOUZA, R.S. POGGETTI, P.D. BRANCO, D. BIROLINI.

Department of Surgery - University of São Paulo - Brazil.

With the aim of aiding the accurate diagnosis and treatment of patients with pancreatic injuries, we reviewed the medical records of sixty-five patients, treated for traumatic pancreatic lesions at the Department of Surgery University of São Paulo School of Medicine in the 5-year period from 1989 through 1993.

Records, including operative and pathology reports, were reviewed to study the location of the pancreatic injury, associated intra-abdominal injuries, type of injury, trauma scores, treatment, complications and mortality rates.

There were 58 male and seven female patients with a mean age of 28.3 years (range, 2-77 years). Of the 65 pancreatic injuries, 45 (69.2%) were caused by penetrating wounds and twenty by blunt trauma. The most frequent site of lesion was the head of the pancreas (38.5%). Associated injuries were found in all but five of the patients. In the 65 patients, 170 intra-abdominal injuries were found or 2.6 per patient. Twenty-eight of the 65 patients (43.1%) had liver lacerations. Lacerations of major abdominal vessels (27 patients), gastric lacerations (25 patients) and colorectal lacerations (17 patients) were the next most commonly seen injuries. Fifteen of the twenty deceased patients died within two days after the accident of severe concomitant injuries. Simple drainage were performed in 33 patients, distal pancreatectomy in 17 and duodeno-pancreatectomy in six patients. Pancreas-related complications occured in 20 (30.7%) of 57 patients who survived the initial operation.

We concluded that the type of repair employed in our series was related to the class of injury and clinical conditions (based on trauma scores). Therefore, whenever possible, conservative management (no pancreatic resection) was employed in patients sustained class I and II injuries and pancreatic resection in class III and IV injuries.

### P264

### IMMUNOHISTOCHEMICAL STUDY OF N-CADHERIN IN DEVELOPING HUMAN PANCREAS

D.D.Zhernossekov, Yu.A.Gaydar, A.Ye.Tikhomirov, G.Sheiketova, L.I.Gaydar, L.N.Moseychuk

Dniepropetrovsk State University, Ukranian Institute of Gastroenterology

Fetal pancreas were studied at 26 embryos received from legal abortion on the development stage of 6, 10 and 14 weeks. In present work avidin-biotin immunohistochemical method was followed by imagine analysis and statistical investigation.
N-cadherin antibodies were a generous gift by Prof.E.Bock ( Protein Lab.Copenhagen ). Ca-dependent cell adhesion protein N-cadherin was observed at eatch investigated stages of development in cell endocrine granules of tubular structures and on the cell surface of ganglia cells in fetal pancreas. Langerhans islets cells which were clearly distinguished after 6 week of embryos development also have N-cadherin.The amount of N-cadherin in these cells was increased according to the development stage.
So N-cadherin could be a good marker for developing endocrine part of pancreas and probably this molecule take part into histogenesis of neuroendocrine complex in this organ.The data could be taken into consideration at the pathological development of pancreas, particulary for undestanding of pancreatic neuroendocrine histogenesis.

### P265

### DISCONNETCTION OF ANASTOMOSIS WITH OVERSEWING THE PANCREATIC STUMP IN THE MANAGEMENT OF DISRUPTED PANCREATICOJEJUNOSTOMY AFTER WHIPPLE'S OPERATION

Yeong-Cheng Huang, Don-Ho Hu, Dah-Cheng Yeh, Hurng-Sheng Wu, Mei-Due Yang, Cheng-Chung Wu, Tse-Jia Liu, and F.K. P'eng, Department of Surgery, Taichung Veterans General Hospital, Taichung, Taiwan, R.O.C.

Dehiscence of pancreaticojejunostomy is a rare but dismal complication after Whipple's operation (pancreaticoduodenectomy (PD)). A completion pancreatectomy has been suggested as the treatment of choice, however, the results have revealed some controversy regarding this technique. Twelve patients developed a disrupted pancreaticojejunostomy after PD were treated by disconnection of the anastomosis, oversewing of the pancreatic stump with a continuous shuttling suture and decompression enterostomy. Although a high morbidity rate (75%) occurred after this procedure, ten patients survived reoperation. No recurrent pancreatic fistula or evidence of diabetes mellitus were noted among the survivors. We recommended this procedure as an alternative method for treating severe pancreatic leakage after PD, without the need for resection of the residual pancreas.

### P266

### TREATMENT OF BILIARY AND PANCREATIC FISTULAS WITH FIBRIN SEALANT.

V. Costantino , C. Sperti, A. Alfano D'andrea, C. Pasquali, S. Pedrazzoli.
Semeiotica Chirurgica , Padua University, Italy

Pancreatic and Biliary surgery are someting complicated by fistulas. We think that their treatment must include an adeguate drainage , the functional suppression of secretions, a careful evaluation of all nutritional parameters and surgical treatment in select cases.We performed all the above coservative techniques in order to achieve a good healing of the fistulas we observed. In addition we used a human fibrin sealnt to fill their tracts. Our overall experience in the treatment of fistulas with fibrin sealant includes 25 enteric, vaginal, pancreatic and biliary fistulas.In the last then years,13 pancreatic fistulas underwend fibrin sealing: 6 followed a pancretico-duodenectomy (2 for cancer, 2 for papillary carcinoma, 2 for endocrine tumors); 2 after left pancreatectomy ( 1 for chronic pancretitis ,1 for cistoadenoma); 1 followed pancreaticojejunostomy for chronic pancretitis; 1 after excision of an insulinoma in the pancreatic head; 3 after a surgical procedure due to an acute pancreatitis (1 necrosectomy and drenage, 1 percutaneous drenage of pseudocyst, 1 cysto-jejunostomy); 2 biliary fistulas of liver after atypical hepatectomy due to echinococcosis. All our patients reseived an adeguate nutritional support and had their secretions reduced by pharmacological treatment.Morever ,they were all submitted to repeated X-ray controls in order to position an accurate and proper drainage. As soon as a regular tract and a low outflow were achieved, the patients underwend the sealing treatment. We used a double lumen catheter under X-ray control which permitted a selective injection of sealant at the origin of the fistulas up to the skin. The tract was thereby completely filled.Of pancreatic fistulas in 11 cases we obtained a good healing with a single injection, 2 patients required 2 treatments; in all 2 cases of biliary fistulas was required to repeteat three treatments. The sealant is self-shaping and its pressure prevents the out-flow of secretions through the fistulas, diverting them into their natural channels. Finally, the components of the components of the sealant support the growth of a good scar tissue. The results we obtained by this techique can be cosidered satisfactory as some patients recovered without any surgical treatment whic would have been otherwise required.

### P267

### TREATMENT OF EXTERNAL PANCREATIC FISTULAS WITH TOTAL PARENTERAL NUTRITION AND OCTREOTIDE

V.Alivizatos, Ch.Kyrkos, V. Athanasopoulos
Department of Surgery and Nutrition Unit, "St.Andrews" General Hospital of Patras, Greece

This study was performed to assess the effect of Total Parenteral Nutrition (TPN) alone or in combination with Octreotide in the management of patients suffering from external pancreatic fistulas During a 6 year period (88-93), 4 patients, mean age 53,7 years, with postoperative external pancreatic fistulas were treated in our Department. There were 3 low-output fistulas (developed as a result of external drainage of pancreatic pseudocyst in 2 patients, and pancreatic injury due to abdominal surgery in one patient), and one high-output fistula as a result of surgical treatment of necrotizing pancreatitis. All the patients had moderate to severe malnutrition at the time of presenting of the fistula. Those with low-output fistulas were treated with TPN, resulting in complete closure of the fistulas within 12-21 days from the beginning of the treatment, respectively. The patient with high-output fistula was treated with TPN and subcutaneous injections of Octreotide (o,lmg every 8 hours).This form of treatment resulted in the decrease of the fistula volume ranging from 30% to 60% of the initial output within the first five days of treatment,whereas complete closure was noted after 18 days from the beginning of the treatment. There were no side effects from the use of TPN and Octreotide. The results of our study suggest that TPN is essential in the treatment of external pancreatic fistulas, whereas Octreotide seems to be a useful adjuvant agent especially in the management of the high-output ones.

### P268

### SOMATOSTATIN-ANALOGUE OCTREOTIDE IN THE CONSERVATIVE TREATMENT OF THE HIGH OUTPUT PANCREATIC AND BILLIARY FISTULAS

C.Dervenis, E.Hatzitheoklitos,M.Avaraki,C.Labrakis,G.Kalafatis
First Department of Surgery, "Agia Olga" Hospital, Athens, Greece

**We performed this study to assess the efficacy of the, Somatostatin-analogue, octreotide, in the conservative treatment of high output (> 500 ml/day) pancreatic and billiary fistulas.**
**During the last 4 years, we treated 17 patients with fistulas, 9 with TPN alone and 8 with TPN and octreotide. The mean closure time was 18 days for the TPN group, and 10 days for the TPN plus octreotide group. The average cost was $ 760 and $ 661 respectively.**
**We conclude that, the use of octreotide in the conservative treatment of the high output pancreatic and billiary fistulas reduses significantly the mean closure time and is a cost effective modality.**

### P269

A PROSPECTIVE RANDOMIZED STUDY FOR PREVENTIG FISTULAS IN PANCREATIC SURGERY WITH THE FIBRIN SEALANT.

V.Costantino, A.Alfano D'Andrea , C. Pasquali , C.Sperti , S.Pedrazzoli .
Semeiotica Chirurgica, Padua University, Italy

Some Authors have suggested the use of Human Fibrin Sealant in pancreatic surgery to prevent fistulas. We performed a prospective randomized study in our institution including 97 patients , 34 females and 63 males. 46 were affected by pancreatic inflammatory diseases and 51 had pancreatic or peripancreatic neoplastic diseases..All the pathients were managed by the same surgical staff.Surgical treatment included 30 pancreaticoduodenectomy (PD), 40 pancreatico-jejunostomy(PJ) ,23 left pancreatic resections (LP) and 4 tumor excision(TE). The patients were randomized at the moment of the surgical treatment ,they were chosen and divided into 2 different groups: Group A , including 43 subjects who had intaoperative fibrin sealing in the anastomosis or pancreatic stump; Group B , including 54 patients who had not fibrin sealing during the surgical treatment. We considered only radiologically assessed fistulas.After surgery were observed 12 (12.7%) fistulas . 6 fistulas were found in group A and 6 in group B . Five fistulas (16.1%) accurred in patients with pancreatic cancer(3 Group A,2 Group B) , 6 (13%) in patients with pancreatitis (3 Group A, 3 Group B); one occurred in a patient ( Group B) with an endocrine tumor . According to the surgical procedure we observed 5 fistulas (16.6%) in cases of PD (4 A,1 B),5 (12.5%) after PJ ( 2 A, 3 B ) ,1 patient ( B) after LP and 1 (B) after TE. Our results don't show any statistically significant difference between the patients treated whit fibrin sealant and the controll group.The higest incidence of fistulas was observed in the patients with pancreatic cancer of group A (18.7%) and in the patients who undervend PD in Group A (25%).

### P271

SOLITARY, UNILOCULAR, TRUE CYST OF THE PANCREAS - CASE REPORT

C. Katsios, D. Nastos, D. Stefanou, D. Cassioumis
Department of Surgery, University of Ioannina, Ioannina Greece

True, unilocular cysts of the pancreas are very rare. Therefore, little is known about their natural history, clinical characteristics, and treatment. To the few described cases we add a new one.
The patient, a 39-yr-old woman, had a 2 years history of epigastric pain, radiated to the right abdomen. On ultrasound and CT scanning a cystic lesion of the neck of the pancreas, with a diameter fo 2.8 cm, was found. The patient looked to be healthy and was put on medical observation. In the meantime endoscopy of the stomach and a barium enema showed normal upper GI tract and colon. Finally the pain aggravated seriously and nausea, vomit and weight loss were added, so a laparotomy was desided. At the operation the pancreatic cyst was enucleated, without difficulty. The pancreas was sutured. Besides the enucleation of the cyst a cholecystectomy was performed. The postoperative course was uneventful, aside from a transient hyperamylasemia. The histology of the cyst showed that was unilocular, and lined with cuboidal epithelium. The gallbladder was normal. The patient is asymptomatic two years after the operation.
Our case shows that such cysts can cause symptoms, and excision seems to be the treatment of choice.

### P270

PANCREATIC PSEUDOCYSTS:PATHOGENESIS AND POSSIBLE LOCATION

A.Agorogiannis and E.Naoum
Surgical department.District General Hospital of Larisa
Larisa-Greece

The aim of this studyis to verify the pathogenesis of pancreatic pseudocysts (PP) and its possible locations.The last 16 years we operated on 4,150 patients with pancreatobiliary deseases.Of these patients 14 had PP.10 were men and 4 women.The mean ageof the patients was 39 years.The aetiology of the PP was alcohol abuse in 4 patients,biliary deseases in 8 patients and blund abdominal trauma in 2 cases.Formation of the cyst propably follows pancreatic ductal obstruction be surrounding edematous parenchyme.The swollen duct ruptures,allowing pancreatic juice (with its proteolytic enzymes) to escape,ultimately through the orga ns capsule,in the surrounding tissue.PP are more commonly associated with inflammatory of the pancreas,usually caused by alcholic abuse or biliary deseases.Many PP occur in the setting of chronic pancratitis from duct obstruction and pancreatic fibrosis,but they may also result in the aftermath of acute pancreatitis fron a process of autodige stion.Most PP are located within the omental bursa.The organs bounding the lesser peritoneal sac,coated by inflammatory dabris and fibrotic material,compose the pseudocyst wall.Although the epiploic foramen is usually sealed by the lesions fibrotic capsule,PP may dissect into the retro perotoneal space,the pelvis,or the thorax.Because of the risk of secontary complications,such as haemorrhage,infection or spontaneous rupture we recommend drainage when a pseudocyst persists after 6-8 weeks of conservative treatment and its wall at this time is thick enough to allow intarnal drainage.In nerarly all cases an internal drainage with a Roux-Y-limb was performed. Our follow-up examination showed that the majority of the patients had done well or satisfactorily postoperatively,with impovement in their general condition and a return to work.Before surge ry,patients with chronic pancreatitis also, need a ERCP to asses the pancreatic ductal system and to identify tho se patients who are canditates for more detaiuled treatme

### P272

*EARLY AND LATE RESULTS OF OPERATIONS FOR PANCREATIC PSEUDOCYSTS*

A.Magyar, L.Flautner, L.Harsányi, I.Pulay,T.Tihanyi
1st Surgical Departement of Semmelweis Medical University, Budapest, Hungary

The authors performed 991 operations for pancreatic pseudocysts in 850 patients during a five years period (01.01.1987 - 31.12.1991) at the 1st Surgical Departement of Semmelweis Medical University.
The pseudocysts were of acute type in 46% and of chronic type in 54%.
The surgical treatment of the pseudocysts was internal drainage in 60%, external drainage in 30% and resection in 10% of all cases. In 50% (499/991) of all operations they performed combined procedures. The indication of combined procedure was either multifocal appearence of pseudocysts (185/499) or coexisting complications of chronic pancreatitis (314/499).
The early postoperative complication rate was 19% and the postoperative mortality was 2,1%. Complications and subsequenting reoperations were significantly ($p<0.01$) more frequent after operations for acute type of pancreatic pseudocysts.
Among the late complications they experienced recurrent or residual pancreatic pseudocyst in 21%, pancreatic fistula in 8%.
Cumulative late death rate was 15,5% till 31.12.1993.
In a 87% follow up rate till 31.03.1994 the mean follow up period was 44 months (ranging from 23 to 84). Based upon a five degrees scale authors classified the results as 23% excellent, 34% good, 30% acceptable, and only 10% poor or 1% bad.

### P273

### SURGICAL TREATMENT OF CHRONIC PANCREATITIS

M.C.C. MACHADO, J.E.M. CUNHA, M.A.C. MACHADO, J. JUKEMURA, A.L. MONTAGNINI, E.E. ABDO, S. PENTEADO, T. BACCHELLA, H.W. PINOTTI.
Department of Surgery - University of São Paulo, Brazil.

Chronic pancreatitis differs from acute or obstructive pancreatitis in that it is difficult or impossible to halt its progression. The aims of surgical treatment are to relieve pain, treat complications and preserve pancreatic function. The appropriate surgical procedure to achieve these ends must be carefully chosen.

The aim of this study is to describe the indications and results of surgical treatment of chronic pancreatitis. We report our findings in 220 patients with complications resulting from chronic pancreatitis. 211 patients were men and nine were women. The main indication was persistent pain (54%) followed by pancreatic pseudocyst (9%), pancreatic ascitis (8.6%) and obstructive jaundice (7.2%). The surgical treatment was established according a preoperative protocol with the following principles: pain relief, ductal obstruction removal, minimal resection of pancreatic parenchyma and return of pancreatic enzymes back to the digestive system. Pancreaticojejunostomy was performed in 111 patients, internal derivation of pseudocyst in 59 patients, external drainage of pseudocyst in 25 patients, drainage of pancreatic abscess in 18 patients, bile-enteric anastomose in 15 patients and pancreatic resection in 15 patients. The operative mortality of pancreaticojejunostomy was 1.8% with postoperative morbidity of 10.9%. Late complications were persistent pain in 9% and reoperation in 7.2%. There was no operative mortality in patients operated on for pseudocyst (86 cases). There was no morbidity among patients that underwent internal derivation (59 cases) and pancreatic resection (2 cases). From the 25 patients that underwent external derivation, 7 presented persistent pancreatic fistula that needed reoperation. The global results were good in 74% of patients.

The goal of surgical treatment is not to cure, but to reduce pain, overcome associated obstruction of the bile duct or duodenum, and to treat pancreatic duct disruptions including pseudocysts and internal pancreatic fistulas. Because continuing deterioration of pancreatic function is to be expected in chronic pancreatitis, maximum conservation of pancreatic tissue by avoiding resectional procedures is advisable.

### P274

### PANCREATIC DUCT MORPHOLOGY CORRELATES WITH EXOCRINE FUNCTION IN CHRONIC PANCREATITIS - RESULTS OF A PROSPECTIVE STUDY

U. Braun, G. Lux, T. Bozkurt. Department of Internal Medicine and Gastroenterology, Community Hospital Solingen, Germany

Pancreatic duct morphology and exocrine function was compared in 48 pts. Transabdominal ultrasound (US), computed tomography (CT), endoscopic retrograde pancreatography (ERP), and a secretin-caerulein test (SCT) were performed in all pts. Findings of US, CT and pancreatogram were based on Cambridge classification. In 10 pts no pancreatic duct changes were found. Equivocal (Cambridge I), mild to moderate (Cambridge II), and considerable ductal changes (Cambridge III) were detected in 10, 12 and 16 pts, respectively. CT and US findings were found to correlate in 40-50%, 67%, and 94-100% of pts with Cambridge I, II, and III abnormal duct morphology, respectively. All pts with normal pancreatogram were without functional impairment. 70% of pts with equivocal pancreatic duct changes had dissociated, and 30% global, pancreatic insufficiency, while 50% of those with mild to moderate abnormal duct morphology manifested dissociated, and 50% global functional impairment. All pts with considerable pancreatic duct changes had global pancreatic insuffiency. Thus, the results of this study confirm that normal morphological ERP findings and Cambridge III ductal changes correlate well with normal pancreatic function and advanced functional insufficiency, respectively. Only in Cambridge III pancreatitis, US and CT, as diagnostic tools, are comparably sensitive as pancreatogram.

### P275

### PANCREATICOPLEURAL FISTULA

Lamesch P, Witzigmann H, [1]Keim V, Otto M, Sierig G,[1]Mössner J, Hauss J

Klinik für Abdominal-,Transplantations- und Gefäßchirurgie

[1]Medizinische Klinik und Poliklinik II - Universität Leipzig, Leipzig - FRG

**INTRODUCTION**
Pancreaticopleural fistulae are serious complications in the evolution of a chronic pancreatitis. The incidence ranges between 1 and 4% of all patients presenting a pancreatic pseudocyst. The course of a case with pancreaticopleural fistula is presented, the therapeutic approach discussed.

**CASE REPORT**
37 year old man was admitted to the department of medecine with the clinical signs of dyspnea and painful right hemithorax. From the past medical history a chronic pancreatitis complicated by a pseodocyst was known since 2 years. The diagnostic work up ruled out a chronic pancreatitis of the head of the pancreas with a right sided pancreaticopleural fistula. Conservative treatment including pleural drainage, stenting of the pancreatic duct and octeotride treatment was unsuccessful after 2 months. The patient finally underwent surgical treatment by means of a Whipple's operation with excision of the pancreaticopleural fistula. The postoperative course was uneventful, the patient was discharged within 3 weeks after operation.

**DISCUSSION**
Chronic pancreatitis is complicated by pseudocyst formation in almost 10% of the cases. Out of these 1-4% develop a pancreaticopleural fistula. Pancreaticopleural fistulae to the right hemithorax do occur in 20% of the cases only, 80% drain to the left side, bilateral fistulae are rare. 50% of pancreaticopleural fistulae can be treated successfully by non surgical procedures. The remainder do require surgery. However, at least 50% of the non surgically treated patients do require an operative intervention later, in most cases due to symptomatic pseudocysts. In the presence of a pseudocyst an operative treatment should be performed after a short trial of conservative treatment. Fistulae without pseudocyst may be better candidates for conservative treatment.

### P276

### PERCUTANEOUS TREATMENT OF PANCREATIC PSEUDOCYST

V. Ivshin, A. Yakunin, Yu. Makarov, I. Kuranov
Tula Regional Hospital, Tula Center of Liver Surgery, Russia

The purpose of this investigation was to evaluate the efficiency of non-surgical treatment of patient with pancreatic pseudocysts. 117 patients (82 men, 35 women) at the age from 26 to 76 years old got percutaneous therapeutic procedur with ultrasound guaidance. 110 patients had cysts caused by pancreatitis and 6 patients had cysts caused by trauma of pancreas. Sizes, localization, the condition of cyst walls were investigated ultrasonically. 55 patients with cysts less than 3-4 cm have been treated by punctures with the aspiration of fluid. 37 of them underwent multiple punctures. The cysts of a lager size were drainaged. All patients underwent biochemical, bacteriological, cytological inves tigations and cystography. 67 patients who had the cysts of the size lager then 3-4 cm got percutaneous drainage with the use of original technique. The duration of drainage was from 14 to 62 days. 99 patients (85.3%) were treated successfully. 18 of all patients required surgery operation (4 - because of the later developed complications, 3 - because of malignization cysts, 11 - because of communication of cysts with pancreatic duct). 2 patients got percutaneous pseudocystogasrostomy. We claim that percutaneous puncture and/or drainage is a safe and effective method for the treatment of pancreatic pseudocysts.

### P277

PANCREATIC HEAD PARTIAL RESSECTION WITH LONGITUDINAL PANCREATOJEJUNOSTOMY IN CHRONIC PANCREATITIS

F. Callejas Neto, J.C. Pareja, V.F. Pilla
Department of Digestive Diseases Surgery, State University of Campinas, Campinas-SP, Brazil

This video shows technical aspects of the partial ressection of the head of the pancreas associated to longitudinal pancreatojejunostomy in chronic pancreatitis. This procedure is specially useful in treating major pancreatic head complications. The head of the pancreas is cored out leaving a rim of pancreatic tissue along the inner aspect of the duodenal loop. The intrapancreatic common bile duct is dissected from the surrounding fibrosis relieving it from the constriction. The main pancreatic duct is fully opened and drained into a Roux-in-Y loop.
This surgery provides an excelent postoperative outcome in regard to clinical as well metabolic data, thus rendering an excelent alternative to ressection procedures (as the Whipple procedure with or without pylorus preservation).

### P278

**CYSTADENOMA MUCINOUS** OF PANCREAS AS ACUTE PANCREATITIS CAUSE

F. Martin Vieira, I. Moreno, R. Garcia, A. Lopez, A. Calvo, L. Lozano, J. Guadarrama, M. Garcia, M. Lomas, F. Botella.
Servicio Cirugia Digestivo(Prof. M.R. Vilariño).Hospital Universitario "Doce de Octubre". Madrid. ESPAÑA.

Pancreatic cystadenoma are rare neoplasms that represent 10% of all benign cystic lesions of the gland.
A case is presented of mucinous cystadenoma of the head of the pancreas, perhaps the least described in the litera ture, that debuted as an acute pancreatitis. On ultrasound examination is found a dilation of the Wirsung duct and the ERCP showed the dilation of all pancreatic duct and an image on his cephalic portion simulating a malignant neoplasm.Needle citology guided by CT was negative for malignant cells.
On the operation is found an encapsulated tumor of 1 cm of diameter with external surface smooth and glistening. The tumor was treated with total excision by means of a plane of separation between the normal pancreatic tissue and the neoplasm.
The cholecistectomy was realized and the intraoperative colangiographie showed the disappearance the previous image of tumor on the pancreatic duct.
The study pathologic showed a tumor cystic with mucus and an epithelium with tall columnar cells mucin-producing.
After one year, the patient is asymptomatic and the study radiologic by CT is normal.

### P279

**HISTOLOGIC FINDINGS AND SURGICAL TREATMENT OF CYSTIC NEOPLASMS OF THE PANCREAS.**

S. Rguez. Dapena, J.D. Sánchez, J.M. Ramia, R. Villeta, J.M. Alcalde, A. Ibarra, C. Morales, A. Abad, D. Ballesteros.
General and Digestive Surgery Service "A". Doce de Octubre Hospital. Madrid. Spain.

**Introduction**: More than 80% of the cysts found in the pancreas are pseudocysts; the rest are true cysts (congenital or acquired) and only a low percentage of these last ones are neoplasms. Cystic neoplasms include a spectrum of lesions from benign to malignant forms depending on their serous or mucinous component: cystadenocarcinomas are malignant, mucinous cystadenomas tend to malignancy and only serous cystadenomas can be considered as benign forms. There is no way to identify which cysts are from each form; image techniques (US, CT scan, MR) can inform about cystic lesions; fine needle aspiration only is conclusive when it's positive for malignancy and only the microscopic exam of all the lesion can define the true diagnosis. This makes that resective surgery should be the elective treatment (also for serous forms because of their high risk of recurrences).
**Material and methods:** We have revised the casuistry from 1986 to 1994 in our hospital: 8 cases. All of them were women from 17 to 56 years (x= 38.8). The head of pancreas was the location in 50%, tail in 37.5% and body in 12.5%. Caudal pancreatectomy with splenectomy was done in all the caudal cysts, enucleation in 25% (one in the head and one in the body) and duodenopancreatectomy in 25% (in the head). The histopatologic findings were: 75% mucinous cystadenomas and 25% cystadenocarcinomas (there was no serous forms). There was only one case with postoperative complications icluding sepsis and death.
**Conclusions:** 1.- Cystic neoplasms in the pancreas are very unfrequent (less than 10% of all the pancreatic cysts). 2.- All of them must be resected because of the impossibility to obtain a true diagnosis without the exam of all the piece and because of the only benign form (serous cystadenoma) tends to recurrence. 3.- Drainage and marsupialization should not be performed. 4.- The location of the lesion determine the surgical procedure (enucleation can be done if it is possible).

### P280

MANAGEMENT TACTICS OF THE NONCOMPLICATED PANCREATIC PSEUDOCYSTS

A.Perejaslov, S.Chooklin
Department of Surgery, Lviv Medical Institute, Ukraine

During 1980-1994 58 patients with pancreatic pseudocysts were treated. Roentgenography, laparoscopy, ultrasonography and computed tomography were used for the diagnostic pseudocysts. Roentgenological investigation and laparoscopy lacked diagnostic accuracy. Diagnostic accuracy of ultrasonography and computed tomography was 95,8%.
In 14 (24,1%) patients uncomplicated immature pseudocysts with the diameter less than 5 cm resolved spontaneously after therapeutic treatment. 20 (34,5%) patients (12 - with immature and 8 - with mature pseudocysts) underwent transcutaneous laparoscopic or ultrasonographic guided drainage. Among the patients with immature pseudocysts we had only one case of the pseudocyst relapse. Any patients with mature pseudocyst did not recovered and all of them have been operated on. 33 (56,9%) patients (5 - with immature and 28 - with mature pseudocysts) underwent surgical intervention. In 12 cases external drainage was used, including marsupialization, and in 21 - internal drainage. In the postoperative period in 8 (66,7%) patients external pancreatic fistula and in 2 (16,7%) - acute profuse bleeding were noticed. Among the patients with internal drainage only one (4,8%) had bleeding in the early postoperative period and this patient died. Other complications were not observed. Internal drainage was performed if the complications were absent and from pseudocyst's formation period notless than 3,5 month. Depending on pseudocyst's localization different kinds of internal drainage were used.
Thus, laparoscopic or ultrasonographic guided drainage can be used in the treatment of noncomplicated immature pseudocysts. The operation of choice in the treatment mature noncomplicated pseudocysts is internal drainage.

### P281

EFFECT OF PROSTENON ON THE PAIN OF CHRONIC PANCREATITIS

Nikolai Budagov, Alexander Yagoda.
Stavropol Medical Institute, Stavropol, Russia.

Pain relief is a primari objective in the treatment of chronic pancreatitis (CP), but one that often difficult to achieve besides on sufficient choice of drugs. The aim of this study was to assess the effect of Prostenon (P), PGE derivate and Baralgin (B), non-narcotic analgetic on the pain of CP: Fourty-four patients with mild manifestation of CP recurrence were studied. Prostenon was administered i.v. at dose 0,04mkg/kg/min. Baralgin was administered i.m. at dose 5ml twice a day.

Results: Effect of P and B in two groups are presented in the table.

| Duration of treatment (days) | Prostenon | | Baralgin | |
|---|---|---|---|---|
| | Patients (n=19) | % | Patients (n=25) | % |
| 1-5 | 5 | 25 | 1 | 4 |
| 6-10 | 5 | 25 | 3 | 12 |
| 11-15 | 5 | 25 | 5 | 20 |
| 16-20 | 3 | 15 | 10 | 40 |
| >20 | 1 | 5 | 6 | 24 |

Results of this study indicate that analgetic effect of was significantly higher when compared to B. We have previously shown that i.vi given P has as stimulatory effect on water and bicarbonate secretion as relaxatory properties of smooth muscles verified by ultrasonografic examination.

Conclusion: Thus properties of P to relieve pain, stimulate directly water and bicarbonate production and relaxate smooth muscles of pancreatic ducts may be helpful in treatment of CP patients.

### P282

SURGICAL THERAPY IN CHRONIC PANCREATITIS

M.Otto, H.Witzigmann, P.Lamesch, K.Kohlhaw, J.Mössner*, J.Hauss

Klinik für Abdominal-, Transplantations- und Gefäßchirurgie

*Medizinische Klinik und Poliklinik II Universität Leipzig, Leipzig, FRG

Introduction:
Indications for surgical intervention in chronic pancreatitis (30-40%) are intractable pain, complications related to adjacent organs and the inability to exclude carcinoma. The surgical approach depends on the pathomorphological parameters and complications. Indications for surgical treatment will be discussed on our own patients.

Patients:
From 1.5.93 to 30.11.94 20 patients underwent surgical intervention for chronic pancreatitis. Of these, 19 suffered from intractable pain (out of them in 9 patients pain was the only sign, in other 10 pain occured in combination with compression of sourrounding organs and suspicion of carcinoma). Important for surgical approach was CT (in 4 cases enlargement of the head, in 4 cases dilatation of the pancreatic duct, and pseudocysts in 11 patients). ERCP was succesfully performed in 8 patients (irregular pancreatic duct in 5 cases, total stenosis in 3 patients, pseudocyst once). 5 patients underwent Whipple's operation, 4 longitudinal Pancreaticojejunostomy, 11 Pseudocystojejunostomy. An emergency drainage was performed in one patient.

Results:
3 surgical complications (severe wound infection, non-sufficient drainage of a cyst, insufficiency of anastomosis). 17 patients reported substantial relief of symptoms. 2 patients deteriorated (recurrency of carcinoma, extreme alkohol abuse). Average short-term follow-up lasts: 5.5 months.

Conclusions:
Resecting and draining techniques are not competitive but completing one to another.Indications for resection are malignancy (1-2%), inflammation located in the head and cauda, combination of complications, unsuccessful draining procedures and specialties (pancreaticopleural fistula). Draining procedures should be used in case of obstruction followed by dilatation of the pancreatic duct. Operations in cases of emergency are extremely rare. As in our own patients (17/20) as in statistics (about 80%) short-term follow-up results are satisfying in both resecting and draining techniques.

### P283

ACUTE GALLSTONE PANCREATITIS:BEST TIMING FOR BILLIARY SURGERY

A.SOUMELAS,A.NOUSIAS,S.BETSIS,K.KALLIGIANNI,A.LIAKOS
1st SURGICAL DEPT.,GENERAL HOSPITAL "ELPIS",ATHENS

After alcoholism,cholelithiasis is the most common cause of acute pancreatitis.Passage of stones through the ampulla of Vater is possibly an important aetiology.To prevent recurrence of pancreatitis from this cause,removal of stones is accepted practice,but the timing of surgery remain open to debate.In the last ten years,82 patients, with mean age of 54 years,were admitted for acute gallstone pancreatitis,for which an operation was performed. Immediate operation(in suspicion of necrotizing pancreatitis,acute cholecystitis and acute cholangitis with jaundice)was undertaken in 21 patients,early or late operation in the remaining 61 patients.The main operation performed was cholecystectomy with common bile duct exploration.Complications occured in 9 patients,mainly in the group who had an immediate operation.Our conclusion is that when stones have been proved in the billiary system, operation should be performed within a few days of the serum amylase returning to normal,and certainly during the same hospital admission.

### P284

SURGICAL TREATMENT OF NECROTIZING PANCREATITIS (NP) COMPLICATIONS:RESULTS OF LONG-TERM (15 YEAR) INVESTIGATION

I.Bondarenko,N.Bondarenko,Ja.Bereznitsky
Digestive Organs Surg.Dept.,Inst.of Gastroenterology;Dept.of Surg.,Medical Academy, Dniepropetrovsk,Ukraine

Acute severe NP is assosiated with serious complications and significant mortality.The goal of our study was to asses the efficiency of re-operations with the aim of post-operative problems correction.

Patients and Methods:At First Aid Hospital 960 patients have been operated on due to NP.There were 84(8,75%) cases of re-operations.

Results:The re-operations were undertaken to treat pancreatic or retroperitoneal abscesses in 60 of the 84 cases,small bowel obstruction (SBO)-in 10,peritonitis-in 9, other complications-in 5.Our repeated surgical efforts were based on wide-raning necrosectomy ,combined with widespread continuous washing and suction drainage.We also applied long intestinal decompression tube in patients with SBO,lavage with antibiotic solution and laparostomy in case of peritonitis.Post-operative mortality rate within re-operated patients was 25%.

Conclusion:We consider that in spite of high risk this surgical strategy is the only possibility to heal re-operated patients after NP.

### P285

OCTREOTIDE IN THE TREATMENT OF ACUTE PANCREATITIS

A. Dovas, G.Papaioannou and N.Tamourides
2[ed] Department of Internal medicine of General Hospital of Larissa, Greece

Acute pancreatitis is associated with significant mortality. To date,no specific treatment has been found for acute pancreatitis.
The purpose of our prospective case-controlled study was to assess the efficiancy of high doses of octreotide in the treatment of patients with acute pancreatitis.
Material and Methonds: 20 patients(10 males and 10 females) 28-75 yrs of age(mean age 52 yrs),were studied. In 15 patients the cause of pancreatitis was biliary and in 5 patients alcohol abuse was determined. The mean Ranson score was 4(range 1-10).Octreotide was begun with the first day of admission in dosage 3x200 mg intravenously per day for a period of 10 days.For comparison a control group consisting of 20 patients with acute pancreatitis, who had not been treated with octreotide, was used. The two groups were comparable with regard to sex, age and the severity of the acute pancreatitis.
Results: 1)In the octreotide group relief of pain was more rapid and the use of analgetics less than in control group of patients 2)The mean duration of Hospitalization in octreotide group was 10±2 days in comparison with the control group where the mean duration of Hospitalization was 15±3 days. 3)Local complications of acute pancreatitis (pseudocyst formation, abscesses and necrosis)were found only in two patients in octreotide group in comparison with control group where local complications were found in 5 patients ($p<0.01$).4) Mortality within 15 days was 15% (3 of 20) in the octreotide group and 25% (5 Of 20) in the control group ($p<0.05$).
Conclusion: The results of our study showed a beneficial effect of octreotide in patients with acute pancreatitis, and should be validated in more prospective, double-blind, well controlled studies in a larger patient population.

### P286

COURSE OF THE ACUTE PANCREATITIS WITH RESPECT TO THE ETIOLOGY.

J. Špičák, V. Dufek, J. Lindner, A. Aunická, J. Martínek, F. Závada

Ist Internal Clinic, Ist Medical Faculty, Praha, Czech Republic

Aim of this study was to specify whether the course of the attacks of pancreatitis is dependent on their origin and to evaluate the differences with respect to the etiology. 112 patients were admitted for the attacks of pancreatitis during two years. The average age of 31 patients with alcoholic pancreatitis (group 1) was 42.2 years, hospitalization length 19.6 days, bilirubin level 28.1 (norm. 18), male/female ratio 28/3, 16 patients underwent surgery and 6 died. The average age of 41 patients with biliary origin (group 2) was 61.4 years, hospitalization length 21.6 days, bilirubin level 45.3, male/female ratio 8/33, 15 patients underwent surgery, and 5 died. In 40 patients with other causes of pancreatitis (group 3) the average age was 48.8 years, hospitalization length 19.3 days, bilirubin level 26.1, male/female ratio 19/21, 9 underwent surgery and 4 died. Conclusions: Course of the attacks of pancreatitis is similar irrespective to their cause. The three groups differ neither in the length of hospitalization nor in mortality. Need for surgery was significantly lower in the group 3. Groups of alcoholic and biliary pancreatitis differ significantly in age, bilirubin level and male/female ratio. From those 15 patients who died 13 underwent surgical treatment. No patient with biliary pancreatitis died after the endoscopic treatment.

### P287

APACHE III SYSTEM AS AN EARLY PREDICTIVE INDEX IN ACUTE PANCREATITIS

C. Agelopoulos, N. Mavroidis, A. Touliatou, B. Papanastasopoulos
Second Department. Pathological, General Hospital of Athens, Greece

The APACHE III system has been created from APACHE II in order to predict better the outcome of severely ill patients. It is a score system that estimates the acute illness, age, and chronic health of the patients. The main advantage of the system is that it can be calculated on the first day of the patients entrance in the hospital.
The aim of this study is to investigate the ability of APACHE III to predict hospital mortality in patients with acute pancreatitis. For this purpose we prospectively studied 59 patients with acute pancreatitis (32 male and 27 females) mean age 61.62 years (36-84). All the patients were admitted to our department during the last three years. All had high levels of serum amylase at least 5-fold the normal value and the CT or US showed swollen pancreas. There were 37 lithiasic, 16 idiopathic and 7 alcoholic pancreatitis. APACHE III score was calculated in all patients and the group of patients without complications was compared with the group with complications and deaths. The Mann-Whitney test was used. The sensitivity (se) and specificity (sp) of the method was also evaluated. 8 patients (4M and 4F) died (11.8%) and 12 patients had major complications (6 respiratory infections, 1 Acute Renal Failure and 5 intraabdominal collections). In the group with complications the APACHE score was 21.38±11.77, and in the group of patients who died the score was 54.5±13.4. If we take the two groups as a whole, then the APACHE score was 43.8±13.4. The comparison between them, based on Man-Whitney test, was 4.981, p=0.000. If the cut off sign of APACHE III score was ≥ 43 the sp of the method in predicting death was 92% and the se was 75%. If the cut off sign was ≥ 40, then the se was 100% and the sp 86% and if it was ≥ 50, the se was 50% and the sp 98%. Using Ranson system and if the cut off sign was ≥ 4, then the se and sp of the method was 37.5% and 92% respectively.
In conclusion the APACHE III system is a valuable early prognostic index and in acute pancreatitis if the score is ≥ 43 the sp and se of the method in predicting death was 92% and 75% respectively. The best se was found when the score was ≥ 39 and the best sp when the score was ≥ 50.

### P288

COMMON BILE DUCT STONES IN MILD AND SEVERE BILIARY PANCREATITIS : AN ERCP REPORT

B. De Waele, T. Peterson, L. Smekens*, G. Willems
Depts. of Surgery and Gastroenterology*, VUB University Hospital, Brussels, Belgium

The systematic use of preoperative ERCP with sphincterotomy in acute biliary pancreatitis (ABP) remains controversial. Some authors advocate that only patients with predicted severe pancreatitis should undergo ERCP, partly because the yield of common bile duct (CBD) stones might be higher in this subgroup. In the present study we looked for a correlation between the severity of pancreatitis and the subsequent finding of CBD stones at ERCP.
In 189 patients with ABP, ERCP with successful cholangiography was available. The severity of pancreatitis was determined by the modified Glasgow scoring system. The presence of gallstones in the CBD was recorded, as well as the time interval between admission and ERCP examination.
In 53 of the 189 patients CBD stones were found (overall incidence of 28%). There were 153 patients with mild pancreatitis (score 0-2) and 36 patients with severe pancreatitis (score 3-8). The incidence of CBD stones in these subgroups was 43/153 (28%) and 10/36 (27%) respectively (NS). The highest incidence of CBD stones was noted in the first 2 days (42%), while it decreased to 20% on the 7th and the 8th day. The rate of disappearance of CBD stones was statistically not different in the two groups.
Conclusion. The incidence of CBD stones and their natural transit time through the CBD was comparable in the mild and severe forms of biliary pancreatitis. The severity of ABP is not a predictive factor for the finding of CBD stones at ERCP.

### P289

DIAGNOSTIC VALUE OF LIPASE/AMYLASE (L/A) RATIO IN ACUTE ALCOHOLIC PANCREATITIS

D.Štimac, M.Rubinić, T.Lenac

Interal Clinic, Gastroenterology division, Clinical Hospital Center Rijeka, University of Rijeka, Croatia

This study was undertaken to confirm the value of L/A ratio in differentiating alcoholic from non alcoholic acute pancreatitis.

Consecutive 129 patients (75 females and 54 males) with diagnosis of acute pancreatitis who had serum lipase and amylase measured on admission were entered into the study. Patients were divided into group A (alcoholic etiology)and group NA (non alcoholic etiology). Group NA was consisted of patients with biliary (B group) and non alcoholic, non biliary (NANB group) pancreatitis.

The L/A ratio was computed by dividing the serum lipase and amylase level by the upper limit of normal in each case. As recommended in previous studies we undertook ratio > 2 in consideration of an alcoholic etiology.

Following our results serum amylase level in alcoholic pancreatitis is lower than in other forms of pancreatitis, while there were no significant differences in serum lipase values between groups. Patients with alcoholic pancreatitis have significantly higher L/A ratio ($p \leq 0,01$) than patients with non alcoholic pancreatitis.

Calculating of L/A ratio is easy method that can help us in early differentation of pancreatitis etiology.

### P290

ACUTE NECROTIZING PANCREATITIS INDUCED BY PROOXIDANT AGENT T-BUTYL HYDROPEROXIDE

Z.Sledziński,M.Woźniak,A.Brunelli,E.Lezoche, E.Bertoli,Z.Wajda
II Department of Surgery,Medical University of Gdańsk, POland, Institut Biochemical Medical University of Gdańsk,Poland and Universita di Ancona,INRCA, Italy

The purpose of this study was to evaluate the possibility of inducing experimentally acute pancreatitis giving into pancreatic duct t-butyl hydroproxide /Bu OOH/ which is well known prooxidant agent. Twenty six animals with pancreatis fistule were randomly divided into four groups Group 1 /n=6/ common bile duct /CBD/ was injected with physiologic saline 1 ml/5 minutes for 3 hours and Group 2 /n=6/ for 6 hours /control groups/, Group 3 /n=7/ CBD was injected with 160 mM Bu OOH 1 ml/5 minutes for 3 hours, Group 4 /n=7/ CBD was injected with 160 mM Bu OOH 1 ml /5 minutes for 6 hours. Results: The rats injected with physiological saline had normal bile pancreatic juice secretionmean volume 1,3 ml/3 h + 0,2 and normal morfology. In the rats injected with Bu OOH, bile pancreatic juice secretion was dramatically reduced to 0,2 ml +o,01 after 3 hours and to 0,5 ml + 0,05 after 6 hours observations period. The light microscopic examination revealed interstitial edema, focal vacuolization of acinar cells and diffuse multifocal necrosis of pancreatic cells with leukocytes infiltration of interstitium of the gland. These changes were more pronunced after 6 hours. Profund intracellular structures damage in pancreatic tissue has been shown in electron microscopic examination. Cinclusion: Infusion of prooxidants agent t-butyl hydroperoxide into pancreatic ducts gives in consequence acute necrotizing pancreatitis which confirms crucial role of free radicals in pathology of this disease.

### P291

HOW TO PREDICT MORTALITY IN ACUTE PANCREATITIS

**M.A. Secchi,E. Tagliaferri,W.Sanchi,L.Scoco,C.R.Gimenez, G.Raimundo.Departments of Surgery, Intensive Care and Radiology.Hospital Italiano and Hospital Provincial. Rosario.Argentina.**

**INTRODUCTION: The aim of this paper is to analized mortality predictive parameters in severe acute pancreatitis (A.P.). METHODS: Two hundred and twenty one patients with A.P. were prospectively studied from 1987 to 1994.Thirty (13.66%)were considered severe:seven patients died (GroupI)and 23 survived(GroupII).The different parameters included in the prognostic systems were analized.1-Original prognostic index that combines 9 parameters(pain,ileus,shock ascitis, creatininemia,glycemia,leukocytosis,high bilirubin level, calcemia)(non-diabetic patients). 2-McMahon (included in our index).3-Apache II. 4-Baltazar-Ranson. The differences between the GroupI(G-I)(non-survivors) and Group II(G-II)( survivors) were statiscally compared by Student's T Test. RESULTS: Creatinine was over 2 mg/ml in 100% of G-I and only in 48% G-II(p<0.001); glycemia was above 180 grams/% in 83% of G-I and only in 48% in G-II(p<0.05). The original prognostic index was higher in G-I:0.55±0.11 than in G-II: 0.45±0.6(NS)/ Ascitic fluid test using McMahon,showed blood or methalbumin in 57% of G-I and 33% of G-II(NS). Apache Score was significantly higher in G-I:21.3±3.5 than to G-II 12.8±4.5(p<0.01).Baltazar-Ranson score by CT scan was slightly higher in G-I:6.0±2.6 than in G-II:4.5±2.0(NS). The presence of pancreatic necrosis was seen in 72% of G-I and in 55% of G-II(NS).CONCLUSION: Variables that proved to be the most sensitive to predict mortality in our patients were creatininemia (included in our Index as well as in the Apache II score) and glycemia.**

### P292

THE EFFICACY OF MULTIFACTORIAL PROGNOSTIC CRITERIA IN DETECTING SEVERE ACUTE PANCREATITIS

A. USLU, A. GÜRKAN, M.TİRELİ
SSK TEPECİK HOSPITAL, THIRD DEPARTMENT OF SURGERY, İzmir Turkey

Early dianosis has great importance in the therapy and prognosis df severe acute pancreatitis. During the past six years, 120 patients who have had 122 acute pancreatitis attacks were reviewed using the RANSON, IMRIE, BANK and AGARWAL's criteria in evaluating the severity and prgnosis of pancreatitis. 79 of the 120 patients were women, and 43 were men. 21 of the 122 attacks were severe and 101 of them mild. The results using the criteria are stated below:

| | Sensitivity | Specivity | Positive predictive value |
|---|---|---|---|
| RANSON | %85.7 | %86.1 | %56.2 |
| IMRIE | %76.2 | %84.1 | %50.0 |
| BANK | %66.7 | %80.2 | %41.2 |
| AGARWAL | %71.4 | %82.2 | %45.4 |

As a result, the efficacy in detecting the severity and prognosis of pancreatitis from these scoring systems do not show remarkable differences; although Ranson and Imrie could be classified as a little more sensitive.

### P293

**PIRENZEPINE TREATMENT EFFICIENCY IN PATIENTS WITH ACUTE PANCREATITIS**

V. Vdovitchenko, V. Vashtchuk

Lviv Medical Institute, Ukraine

It is known a wide spectrum of pirenzepine action on gut: inhibition of gastric and pancreatic secretion, weakening of Oddy sphincter tonus etc. This study was performed to assess the efficiency of this medicine in complex treatment of patients with acute pancreatitis. We observed 26 patients (males 19, females 7, mean age 46,8 years) with a swelling (16 persons) and a destructive (10) form of acute pancreatitis. All patients received in the first 5 days fluid replacement, strong analgetics, diuretics and pirenzepine (Gastrozepine, Boehringer Ingelheim Pharma) 10 mg i.v. and i.m. every 6-8 hours. Patients of control group (corresponding to quantity, sex, age and the severity of acute pancreatitis) receivd the same drugs but atropine sulphate (0,5 mg on injection) instead of pirenzepine. In patients with swelling form of acute pancreatitis for pirenzepine administration the pain syndrom decreased for 1,7±0,1 days and urine amylase normolized for 3,8±0,2 days versus 3,5±0,5 and 5,8±0,3 days in control group. But we didn't reveal any effect of pirenzepine on clinical and bichemical tests in patients with a destructive form of acute pancreatitis. 4 patients of basis group died versus 3 patients of control group. So, application of pirenzepine is effective only in patients with swelling form of acute pancreatitis.

### P294

MYOCARDIAL INFARCTION - SYSTEMIC COMPLICATION OF SEVERE ACUTE PANCREATITIS

V.Živković, V.Katić, T.Stanković, T.Jocić, A.Nagorni

Faculty of Medicine in Niš, Niš, Yugoslavia

The ultimate pathogenetic process in acute pancreatitis is the proteolysis,lipolysis and haemorrhage resulting from the destructive effect of pancreatic enzymes released from acinar cells.
We report two victims with severe abdominal pain in the epigastrium radiating to the back and to the heart, dead from "myocardial infarct, proved by clinical methods.
Autopsy findings: in addition to acute haemorrhagic pancreatitis,accompanied by diffuse fat necrosis,the following changes in heart were observed:
disseminated intracapilar coagulation with multifocal myocitolysis and coagulative necrosis(in the heart).
necrosis( in the heart ).
Both release of toxic enzymes (proteases,lipases and elastase) into systemic circulation and their role in pancreatic and systemic lesions are the most important events in pathogenesis of acute pancreatitis.

### P295

DUCT-TO-MUCOSA PANCREATOJEJUNOSTOMY AND SINGLE LOOP RECONSTRUCTION AFTER PANCREATICODUODENECTOMY

J.C. Pareja, F. Callejas Neto, V.F. Pilla, S.L.S. Vaz, L.S. Leonardi
Department of Digestive Diseases Surgery, State University of Campinas, Campinas-SP, Brazil

The propensity for leakage at the site of pancreatojejunostomy is a major reason for morbidity and mortality after pancreaticoduodenectomy. We report the results of our Institution with single loop reconstruction and duct-to-mucosa pancreaticojejunostomy. From January 1991 to September 1994, 33 patients were submitted to pancreaticoduodenectomy with this kind of reconstruction, nineteen patients (57,5%) were men and fourteen (42,5%) were women. The mean age was 54 years (range from 30 to 75 years). The ressection was performed for ampullary carcinoma (19 patients - 57,5%), pancreatic carcinoma (11 patients - 33%), biliary duct carcinoma (2 patients - 6%) and pancreatic non-functioning endocrine tumor (1 patient - 3%). We reformed pancreaticoduodenectomy with linphadenectomy (R2) and single-loop reconstruction (biliary-pancreatic-gastric). There was neither operative mortality, nor pancreatic fistula. The morbidity was related to pneumonia (1 patient), pleural effusion (1 patient), wound infection (2 patients), gastrojejunostomy hemorrhage (1 patient), biliary fistula (2 patients). The average postoperative stay in the Hospital was 10 days. Radiological control of the pancreatojejunostomy was performed in 20 patients after 6 months showing patency in all but one patient (main pancreatic duct contrastation or elevated amylase levels inside the jejunal loop).
The results confirm that duct-to-mucosa pancreato jejunostomy is a safe procedure drainage after pancreaticoduodenectomy.

### P296

PANCREATICO-JEJUNAL ANASTOMOSIS: A NEW METHOD IMPROVING THE RESULTS OF PANCREATODUODENECTOMY.

Onopriev, A.Manuilov, M.Rogal
Republican Centre of Surgical Gastroenterology, Krasnodar, Russia

Materials of 169 pancreaticoduodenectomies (PD)performed upon patients with periampullary adenocarcinoma were studied.The purpose of this work is the formation of a safe pancreatico-jejunal anastomosis for the friable stump of the pancreas after PD.Experience in pancreatic surgery shows that formation of pancreatico-jejunal anastomosis when the tumor mass impinges on the major pancreatic duct is not difficult. The formation of the anastomosis is not difficult in case of fibrosis of the pancreatic tissure and dilatation of the major pancreatic duct up to 4-5 mm. In case the tumor is located at a distance from a normal Wirsung's duct with a diameter of 2-3 mm we use an early external drainage of the pancreatic stump,due to fear of anastomotic leakage.We introduce a new pancreatico-jejunal anastomosis.The pancreatic stump is mobilized in a distance of 3-4 cm below the margines of dissection. Hemostasis is carried out by the precise suture of vessels of the pancreatic stump. The pancreatic stump is delivered through the mesenteric opening and a loop of the jejunum is wound around it.The submucosal layer of the jejunum around the pancreatic stump is widely exposed by dissection.The wound surface of the pancreatic stump is buried in submucosal layer.The isolated pancreatic duct is implanted into the bowel lumen.The pancreatic duct is drained externally using Imanaga's method.Forty-seven PD were carried out. Early postoperative complications were not observed.

### P297

THE USAGE OF METALS WITH THE MEMORY OF FORM IN THE TREATMENT OF PANCREATIC DISEASE

T.B.Komkova,G.T.Dambayev

Siberian Medical Univercity,Tomsk,Russia

The problem of surgical treatment of pancreatic diseases has not been solved yet,as the existing methods do not lead to the desired results.
Experimental studies of animals proved the possibilite of usage of metal staples with the memory of form for pancreatic resections, after that the methods was applied in the clinic in cases of chronic pancreatitis.
Metal staple with the memory of form causes sclerosis of pancrestic parenchima and closes the pancreatic duct,which leads to the decrease of painful syndrome and prevents the development of pancreatic fistula.
The usage of metal staples with the memory of form makes it possible to decrease the painful syndrome,to preserve endocrinic function of the pancreas and to avoid the formation of pancreatic fistula.

### P298

POSTOPERATIVE COMPLICATIONS AFTER PANCREATODUODENECTOMY

Th Polymeropoulos,E Yettimis,P Vachliotis,
H Tsipras,E Kalokerinos,J Maroulas,G Limatzakis
1st Dep. of Surgery,Athen's General Hospital
Greece

We present our experience in the treatment of postoperative complications after pancreatoduodenectomy (PDM) for cancer in 18 pts since 1988.Of them 10p. had pancreatic cancer,1p duodenum cancer,and 1p malignant somatostatinoma.Fifteen p. underwent PDM with pyloric preservation in 7p, 2p distal pancr/my and 1p total pancr/my.In all cases the main pancreatic duct was ligated.The operation was thought therapeutic in 13p.One pt died in the immediate postoperative period because of hemorrhagic pancreatitis.The main post. complications seen were:small bile leakage(1p),subhepatic collection (1p),pancreatic fistula for less than 30 days (3p), and cyst of the pancr. tail(1p).Long standing pancreatic fistulas (1-3mo) were seen in 2pts.Reoperation was performed for treatment of the the subhepatic collection, the pancr.cyst and the longstanding fistulas.Five pts survived 8-31mo (mean survival 17,8 mo).The remaining 12p are still alive 2-66 mo after PDM.Conclusion:(a)Best post. results depent on strict selection of pts undergoing PDM and fine surgical skill,technic and experience (b)Long standing post. fistula may easily managed with anastomose of the fistulous tract to the intestine.

### P299

OUR EXPERIENCE ON PANCREATICODUODENECTOMY (PD): PROCEDURE BY PANCREATIC-GASTRIC ANASTOMOSIS END-TO-SIDE AND BY NASOPANCREATIC DRAINAGE

M. Vajo, F.V. Contino, L. Rabagliati, M. Fusaro, A. Bruno, G. Malandrino, R. Suriani*.
Chirurgia Generale, Ospedale Civile, Rivoli (Torino),ITALIA
*Servizio di Endoscopia Digestiva, Ospedale Civile, Rivoli

The Authors report their experience on a surgical technique used in the last seven cases in PD performed for pancreatic head cancer and ampulluma. By this alternative surgical approach the most common complications, i.e. fistula or pancreatic cysts formation, have been prevented.
After the resection of the duodenum, of the head of the pancreas, of the first jejunal segment, by "decrossage" under mesenteric root and piloryc conservation (Traverso-Longmire procedure), we apply this type of reconstruction: pancreatic -gastric anastomosis on posterior wall end to side, by interrupted suture of nonabsorbable monothread; insertion into Wirsung of a nasopancreatic catheter (WC 7 fr); end-to-end piloryjejuno mechanical anastomosis; end-to-side choledocojejuno anastomosis by interrupted suture of nonabsorbable monothread; insertion of 2 underhepatic and parahepatic drainages.
The postoperatory period has been normal for all patients. They didn't show any complications as well as fistula or wound infection.
Conclusion: our date show that the PD procedure py pancreatic-gastric anastomosis associated with nasopancreatic drainage, even if in a small series, is safe and well tolerated. From a surgical vieuwpoint, the procedure can be performed without any supplementary difficulties compared to the standard procedure.

### P300

OXYGEN DELIVERY/OXYGEN CONSUMPTION (DO2 /VO2) ALTERATIONS DUE TO DOBUTAMINE , IN POLYTRAUMA HPB SEPTIC ICU PATIENTS.

E.Pavlou , A.Kalogerometros , M. Stavropoulou , H.Ioannidou
ICU Departement of KAT General Hospital , Athens , Greece

We studied the hemodynamic effects and the oxygen delivery / oxygen consumption (DO2/VO2) relationship alterations of dobutamine administration in 13 polytrauma , HPB ,septic , ICU patients , 9 male and 4 female , mean age 43 years( 22-69) All of them were admitted to the ICU because of acute respiratory failure due to polytrauma and HPB surgery . All of them were sedated , and mechanically ventilated with PEEP. When the study was performed , they all were septic according to the sepsis criteria. No inotropes were administered before . The hemodynamic profile was studied with a Swan-Ganz catheter of continuous SVO2 monitoring (oximetrix-ABBOTT) . We administered dobutamine 5 and 10 μg/Kg/min with an interval of 60 min . Calculations were performed before and after the dobutamine administration . Statistical analysis was done with ANOVA test .
**Results** : With 5 μg/Kg/min of dobutamine we had an increase in DO2/VO2 . DO2 increased by 25% (p<0,01) , while VO2 by 13% (p<0,05) . With 10 μg/Kg/min of dobutamine we had an additional increase of 7% in DO2 ( total DO2 increase by 32%) while no additional change was seen in VO2 . Heart rate increased by 14% (p<0,05) while the increase in cardiac index was 20% (p<0,01)
**Conclusions** : Dobutamine administration in ICU polytrauma , HPB septic patients had favourable results in tissue oxygenation , because of the oxygen transport (DO2) and oxygen consumption (VO2) increase . The best dose related results were seen in 5 μg/Kg/min .

### P301

ULTRASONICALLY GUIDED PERCUTANEOUS DRAINAGE ABDOMINAL ABSCESSES- 81 patients

N.Ivaniš,Glavaš R,Včev A,Perić R,Šustić A.

Medical Centre-Rijeka,CROATIA

Interventional ultrasound is today an established method in the diagnostics and therapy of a numerous gastroenterological diseases.

In the course of the past five years,between 1989 and 1994,more than thousand ultrasonic interventions had been performed (punctures,drainage). In this group of patients thera were 81 patients with abdominal abscesses,which were by percutaneous ultrasonically guided aspiration and drainage treated. Various drainage sets and puncture needles of Angiomed had been used(LADS, GADS,OTTO,SUMP).

Only 6 of 81 patients wich were treated by ultrasonically guided multiple aspirations and drainage required surgical intervention.

So 92,6% patients with abdominal abscesses were successfuly treated by percutaneous aspiration or drainage,and only 7,4% required surgical treatment.

Conclusion is that ultrasonically guided percutaneous treatment of abdominal abscesses is the method of first choice in management of most cases. Surgical treatmentremains only alternative method for a little number of patients where percutaneous drainage was not successful.

Our methodology,results and conclusions will be detaily described on the poster.

### P302

SKIN LESIONS IN DIABETIC PATIENTS

Lj.Spalevic, M.Spalevic, V.Katic, Lj.Nikolic, D.Jovanovic

Clinic for Skin and Veneral diseases - Clinical Centre Nis, Department for Dermatoveneorology, Medical Faculty - University of Nis, Nis, Yugoslavia.

The relation between poor glycemic control and development of diabetes mellitus has long been recognized. Association of large-vessel disease and microangiopathy is also well known. However, the role of duration of diabetes and fasting-blood glucose levels on the development of the various skin lesions, permanent theme of research, has caused our study, too. In 50 dermatological diabetics with the duration of disease between 5 and 10 years, and with various blood glucose levels, as well as in 20 autopsied diabetics, the skin lesions were studied. The results demonstrated the various degree of atopic skin lesions, complicated by infections, the ulcerous and gangrenous changes were localized predominantly on legs. The positive correlation was found more frequent between the severity of skin lesions and the blood glucose levels than between the duration of diabetes and observed lesions. In addition, positive correlation was found between nepropathy and skin lesions (autopsied cases).

### P303

IMMUNOHISTOCHEMICAL STUDY OF N-CAM IN HUMAN FETAL GASTROENTEROPANCREATIC SYSTEM

Yu.A.Gaydar, M.Witt, E.A.Lepekhin, G.A.Sheiketova, L.I.Gaydar
Ukranian Institute of Gastroenterology, Dresden University, Dniepropetrovsk State University

The N-CAM is a cell surface glycoprotein involved in direct cell-cell adhesion. The purpose of the study was to investigate the N-CAM distribution in the human fetal gastroenteropancreatic system (GEPS). The samples from 30 fetuses ( 6 - 14 weeks of gestation ) were collected at legal abortions. The tissues were fixed in Bouin's solution for light microscopy study.

Thin paraplast sections were stained by a rabbit polyclonal antibody to N-CAM followed by avidin-biotin-peroxidase staining technique.

N-CAM was found at the surface of neuronal cells in intramural neuronal apparate of GEPS.

N-CAM positive endocrine cells contained gastrin in both stomach and small intestine and vasoactive intestinal peptide in fetal islets of Langerhance.

N-CAM may used as a neuroendocrine markers of fetal GEPS. We suppose that soluble form of N-CAM is necessary for normal histogenesis of GEPS.

### P304

MODIFIED MINI-CHOLECYSTECTOMY: A minimally invasive procedure.

Hamdi Ibrahim Ali, Abdussalam A. Khamage, Jumma M. Faitori.
Dept. of Surgery, Hawari Hosp., Arab Medical Univ.,Benghazi -Libya.

Minimally invasive surgery is being increasingly employed and extended to various procedures, it reduces hospital stay and shortens recovery interval, with excellent cosmetic results, and is subsequently preferred by patients. Short incisions tend to be associated with less postoperative pain. Tissue destruction is minimised and the risk of wound complications is probably diminished as a result.

We report the results of modified mini-cholecystectomy performed on 148 patients (127 women, 21 men). The mean age of the patients was 37 years, (range 18-65 years), while the mean weight was 79.9 kgm (range 61-135 kgm). 88.5% of patients were overweight (5-98% in excess of standard chart based on height and weight).

The incisions used were 2 to 4 cm long, and the procedure was carried out employing selected laparoscopic instruments. Sixteen patients had mucocele and 7 had empymatous gall bladders. The incision had to be extended in 11 patients due to obscured anatomy (5 patients) or for unanticipated exploration of the common bile duct (6 patients). Nasogastric tubes were not employed, peritoneal drainage was instituted for cases with infected gall bladder. All patients were allowed oral intake after 6 hours from operation. The mean period of hospital stay was 2.08 days (range 1-5 days). The operative time ranged from 25-75 minutes, generally tending to get shorter towards the end of the study period, presumably a reflection of the learning-curve effect. No major postoperative complications were encountered in any of our patients during the follow-up period 1-21 months.

We conclude that modified mini-cholecystectomy is a simple and safe procedure. In our estimate the operation is applicable to over 90% of patients scheduled for elective open cholecystectomy and in whom preoperative ultrasonography reveals a normal biliary tree. Details regarding preoperative cholangiography, results and operative technique will be presented.

### P305

### SEGMENTAL SPLENECTOMY FOR SPLENIC CYSTS.

Hamdi Ibrahim Ali, Abdussalam A. Khamage, Jumma M. Faitori.
Consultant General Surgeons, Hawari Hosp., Arab Med. University, Benghazi-Libya.

Post-splenectomy sequelae are now well recognized, and conservative splenic surgery is widely advocated. Splenic cysts are uncommon, Non-parasitic splenic cysts have generally been categorised as either true epidermoid or false post-traumatic pseudocysts. Meanwhile, in areas of endemic hydatid disease, the spleen is not a rare site of parasitic larval infestation.

We present five patients with splenic cysts; epidermoid, pseudocyst and infected hydatid cyst, treated successfully by segmental splenectomy. Patients had unremarkable post-operative period and were discharged after 7-10 days of surgery. During a 2-year follow-up by doppler ultrasonography, intact splenopedal blood flow was confirmed in all patients. No recurrence has been noted.

Recognition of the biological importance of the spleen, together with the advancement in imaging modalities and operative surgical techniques should initiate a strategic change in the management of splenic cysts.

### P306

### HEPATIC ASPARTATE AMINO TRANSFERASE (mAST) ISOENZYME ACTIVITY IN CHRONIC C HEPATITIS

Teodora Macchia, Rosanna Mancinelli, Oriana Leri*, Edgardo Lugaresi, Pierluigi Russo**, Stefano Gentili, Anna Paggi**
Department, of Clinical Biochemistry, Istituto Superiore di Sanità, *Department of Infectious Diseases , **II Internal Medicine, University "La Sapienza", Rome, Italy.

In liver 'and in serum Aspartate AminoTransferase (tAST) activity is dependent on two isoenzymes, which are mitochondrial (mAST) and cytosolic (cAST). In order to verify if tAST levels could be correlated to mAST, or if the mAST activity could be a precocious and sensitive marker of chronic HCV hepatitis, we have studied 21 patients (13 F, 8 M); none was alcoholist. Nine of them (6 F, 3 M, mean age 67,33±16,48) had persistently normal liver function indexes, were positive for HCV antibodies (ELISA 1st and 2nd generation tests), while HCV-RNA, performed by Polymerase Chain Reaction (PCR) according to Chomczynsky and N. Sacchi, was positive in eight. Twelve patients (7 F, 5 M, mean age 61,92±5,63) affected by chronic C hepatitis were also studied. All had persistently increased enzymatic activities and HCV-RNA positivity. In those last patients liver biopsy scoring (Knodell) was assessed; it showed periportal necrosis, lobular and portal inflammation according to chronic active hepatitis. The mAST separation was performed by an immunochemical procedure with dryed blood cells from sheep which have antihuman antibodies soluble AST isoenzymes attached on their surface (mGOT-TEST immunoassisted, Poli Diagnostici, Italy). After incubation and centrifugation, residual AST activity corresponding to the mitochondrial fraction was determined by a standard spectrophotometric procedure (AST-MONOTEST acc. to IFCC, Boehringer Mannheim, Germany). Statistical analysis was carried out by the Spearman's correlation, evaluating mAST/tAST ratio. There was no correlation between mAST/tAST ratio in both groups (r=0.302, and 0.480, respectively), irrespective of the type of the disease. Our data differ from the ratio obtained in patients affected by alcoholic steatosis.

